# Indole Compounds in Oncology: Therapeutic Potential and Mechanistic Insights

**DOI:** 10.3390/ph17070922

**Published:** 2024-07-10

**Authors:** Sara M. Hassan, Alyaa Farid, Siva S. Panda, Mohamed S. Bekheit, Holden Dinkins, Walid Fayad, Adel S. Girgis

**Affiliations:** 1 Biotechnology Department, Faculty of Science, Cairo University, Giza 12613, Egypt; 2 Department of Chemistry and Biochemistry, Augusta University, Augusta, GA 30912, USA; 3 Department of Biochemistry and Molecular Biology, Augusta University, Augusta, GA 30912, USA; 4 Department of Pesticide Chemistry, National Research Centre, Dokki, Giza 12622, Egypt; m_bekheit@yahoo.com; 5 Drug Bioassay-Cell Culture Laboratory, Pharmacognosy Department, National Research Centre, Dokki, Giza 12622, Egypt

**Keywords:** indole, cancer, antiproliferation, synthesis, mode of action

## Abstract

Cancer remains a formidable global health challenge, with current treatment modalities such as chemotherapy, radiotherapy, surgery, and targeted therapy often hindered by low efficacy and adverse side effects. The indole scaffold, a prominent heterocyclic structure, has emerged as a promising candidate in the fight against cancer. This review consolidates recent advancements in developing natural and synthetic indolyl analogs, highlighting their antiproliferative activities against various cancer types over the past five years. These analogs are categorized based on their efficacy against common cancer types, supported by biochemical assays demonstrating their antiproliferative properties. In this review, emphasis is placed on elucidating the mechanisms of action of these compounds. Given the limitations of conventional cancer therapies, developing targeted therapeutics with enhanced selectivity and reduced side effects remains a critical focus in oncological research.

## 1. Introduction

Cancer is one of the biggest health challenges to mankind, considered the second most deadly disease, trailing cardiovascular disease [1,2,3,4]. Due to its invasive and aggressive proliferation, cancer may spread into other tissues, causing metastatic capability [5,6]. Despite several tools, therapeutics, and strategies currently developed and applied to manage the disease, many cancer patients are vulnerable to drug resistance, which reduces the efficacy of different therapies [7,8,9]. In this context, the search for safe anticancer agents with high potency, selectivity, and minimal off-target effects is an urgent demand. Paying attention to novel therapeutics such as gene [10], immune [11], and photodynamic [12] therapies is also a noticeable trend to attain effective approaches for combating diverse cancer types, especially in the advanced phases.

Indole analogs are widely distributed as natural compounds in animals, plants, and microorganisms [13,14,15]. Many indole analogs were reported with potential biological properties, among them anti-SARS-CoV-2 (severe acute respiratory syndrome coronavirus-2) [16,17,18,19,20,21,22], anti-malarial [23,24], antimicrobial [25,26], and anti-inflammatory [27,28,29], in addition to approved drugs for the treatment of several diseases [30,31,32,33,34,35,36,37,38,39,40,41] (Table 1).

Cell death is crucial and fundamental for maintaining tissue balance and eliminating potentially harmful cells in multicellular organisms. Accidental cell death (ACD) is typically caused by unintentional injury, while regulated cell death (RCD) is programmed cell death controlled by signaling pathways necessary for an organism’s development and/or tissue renewal [42]. Autophagy, necrosis, and apoptosis are significant types of RCD. They are potent approaches against cancer progression and metastasis and are important for developing potential anticancer agents [43,44].

Indole analogs have been recognized as potent anticancer agents targeting RCD and related signaling pathways [45,46]. So, they may control cancer cell progression via various biological targets, including tubulin polymerization, DNA topoisomerases, tumor vascularization, histone deacetylase (HDAC), and sirtuins [46,47,48]. Moreover, efficacy towards drug sensitivity and resistance in vitro and in vivo were also reported [49].

**Figure 1:**
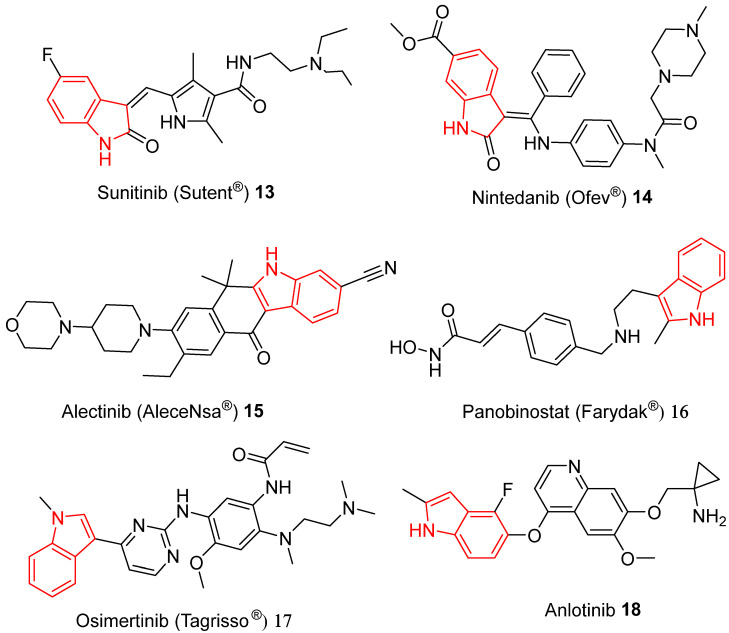
Clinically approved indole-containing drugs **13**–**18** as anticancer agents.

Sunitinib (Sutent^®^) **13** (Figure 1) is a famous clinically approved drug by the FDA against imatinib-resistant gastrointestinal, pancreatic, and high-risk renal cancer in adults. Sunitinib inhibits cellular signaling/multi-target tyrosine kinases related to tumor growth, angiogenesis, and metastatic progression. The antitumor activity of sunitinib is attributed to PDGFR and VEGFR (platelet-derived and vascular endothelial growth factor receptors, respectively) inhibition that reduces tumor vascularization and size [50,51]. Nintedanib (Ofev^®^) **14** is an indolinone-derived intracellular tyrosine kinase inhibitor drug awarded FDA approval against NSCLC (non-small cell lung cancer) with potential anti-angiogenesis properties and inhibitory activity against PDGFR-α, -β; VEGFR-1, -2, -3; and FGFR-1, -2, -3 (fibroblast growth factor receptor) [52,53,54,55,56,57]. Alectinib (AleceNsa^®^) **15** is usable against NSCLC [58,59,60,61], panobinostat (FarydaK^®^) **16** against multiple myeloma [62], osimertinib (Tagrisso^®^) **17** against NSCLC [63], and anlotinib **18** against NSCLC as well as metastatic colon cancer [64]. They are also indolyl-containing drugs approved by the FDA (except anlotinib, which is approved by the National Medical Products Administration (NMPA) of China). 

The current study summarizes the recently reported indolyl analogs, either naturally isolated or synthetically prepared, with potential antiproliferative activity against different cancer types within the last five years, utilizing different search engines (Scopus, ScienceDirect, and Pubmed) and specific keywords (indole; cancer; antiproliferation; synthesis; mode of action). The study adopts the classification of potential indole-containing compounds against the most common cancer types. The mode of action mentioned for the reported analogs is one of the main concerns of this study. 

## 2. Natural Indoles with Potential Antiproliferation Properties

Natural compounds from different resources (plants, animals, or microorganisms) have significantly revealed therapeutic possibilities for treating different diseases. Many natural compounds can be used directly or give inspiration for designing/optimizing potent agents/therapeutics [65]. Despite the obstacles in natural product drug-based discovery, including the limitation of chemical structure diversity accessed for different diseases and the low supply relative to the needs [66], continuous progress in technical screening, isolation, and characterization may increase the number of natural compounds accessible as potential therapeutical candidates. The potential of natural indole-containing compounds as promising candidates for cancer treatment, with some structural modifications or in their monomeric forms, is an inspiring and motivating prospect for the future of cancer research [67,68].

**Figure 2:**
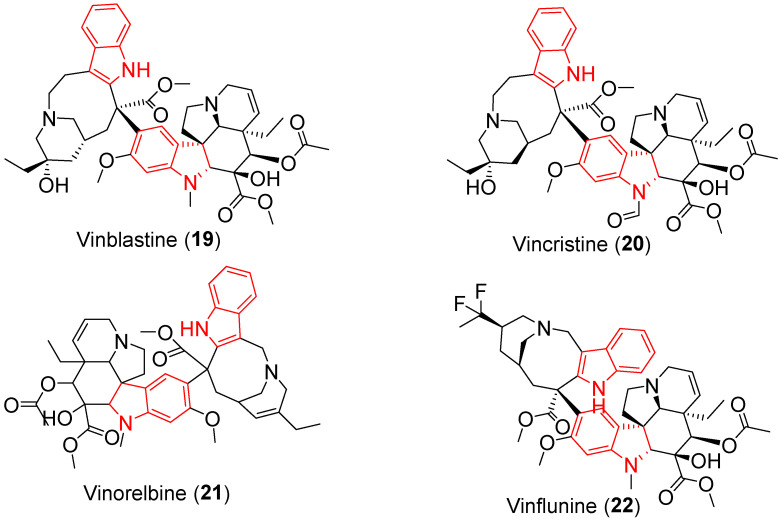
Chemical structure of vinca alkaloids (vinblastine **19**, vincristine **20**, vinorelbine **21**, and vinflunine **22**).

Indole is an essential branch of alkaloids widely presented in many natural resources and other alkaloid subsets with various biological properties [14,42,69]. It has been reported that indole alkaloids can control cell death by regulating signal pathways responsible for the death mechanism, thus exerting promising anticancer efficacy [45]. Vinca alkaloids have shown broad-spectrum antitumor properties alone or in combination with other agents. Vinblastine **19**, vincristine **20**, vinorelbine **21**, and vinflunine **22** are capable of interfering with microtubule function, inhibiting angiogenesis, and causing cell cycle arrest and cell death [45] (Figure 2). Vinblastine (Velban) (against lymphoma, testicular, and breast cancers) and vincristine (against lymphoma and neuroblastoma cancers) are tubulin polymerase inhibitors that have been clinically approved as antitumor drugs [47,70,71,72,73,74].

### 2.1. Breast Cancer

Breast cancer is one of the most common causes of death among women’s cancer types globally. It is categorized into receptor-positive and triple-negative types [75]. Treatment options include surgery, radiotherapy, chemotherapy, hormone therapy, and immunotherapy [76,77]. Metastasis poses another challenge: the disease can spread to vital organs such as the lungs and bones or lead to lymphoma [77].

**Figure 3:**
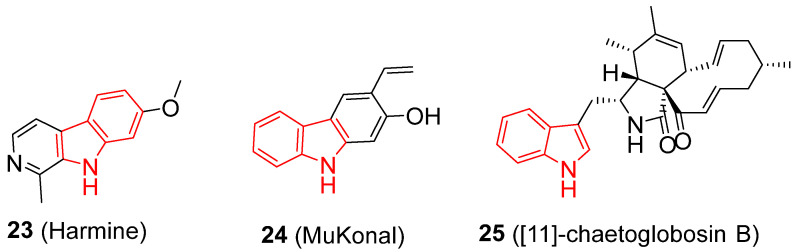
Chemical structure of indole alkaloids **23**–**25** with antiproliferation properties against breast cancer.

Harmine **23** (Figure 3) is an apoptosis-inducing indolyl analog isolated from the seeds of *Peganum harmala*. The antiproliferation and control of the migration of breast cancer cells (MDA-MB-231 “triple-negative” and MCF-7) by harmine were reported. Its capability for controlling/downregulating the overexpression of TAZ (PDZ binding motif) was also mentioned. Additionally, inhibition of proteins including p-Erk (phosphorylated extracellular signal-regulated kinase), p-Akt (protein kinase B), and Bcl-2 (B-cell lymphoma 2) was reported [78].

Mukonal **24** (obtained from *Murraya koenigii*) (Figure 3) exhibits potential antiproliferation properties against SK-BR-3 and MDA-MB-231 breast cancer cell lines with an IC_50_ value of 7.5 μM (MTT “3-(4,5-dimethylthiazol-2-yl)-2,5-diphenyl-tetrazolium bromide” assay) and safety behavior against normal breast cells (MB-157). The antitumor effect was attributed to its apoptosis capability, which was supported by its role in the enhancement of the cleavage of PARP and caspase-3, as well as controlling the Bcl-2 level. Enhancement of the expression of autophagy proteins (Beclin-1, LC3-I, and LC3-II) also emphasizes/justifies the anti-breast cancer properties. The in vivo study (xenografted mouse models) demonstrated that mukonal significantly decreased tumor weight and volume [79]. 

[11]-Chaetoglobosin B **25** (isolated from the fermentation of *Pseudeurotium bakeri* fungus) (Figure 3) exhibits promising cytotoxic activity against the MCF-7 cell line relative to that of doxorubicin hydrochloride (IC_50_ = 6.2 and 1.2 μM, respectively). Arrest of the cell cycle at G2/M was achieved via flow cytometric assay. Moreover, the apoptotic activity was supported due to the increment of the Bax and CyT-c levels, the cleavage of caspase-3 and PARP, and the decrease in Bcl-2 expression (Western blotting technique) [80].

### 2.2. Lung Cancer

Lung cancer is a leading cause of worldwide mortality. Many environmental risk factors, along with smoking, are associated with lung cancer [81,82,83]. NSCLC is an aggressive type [81]. Surgery and chemotherapy are preferred options for early-stage patients, but detecting the disease early is challenging. Prevention through dietary changes and avoiding tobacco smoking is important [84].

**Figure 4:**
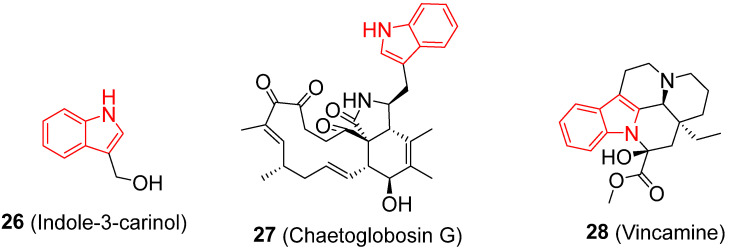
Chemical structure of indole alkaloids **26**–**28** with antiproliferation properties against lung cancer.

Indole-3-carbinol **26** (Figure 4) (found at high levels in *Cruciferous* vegetables) displays anticancer activity against H1299 lung (NSCLC) cancer cell with IC_50_ = 449.5 μM (MTT assay) and safe behavior against CCD-18Co, a normal cell. It also increases the expression of ROS (reactive oxygen species) and activates apoptosis-related signals. Furthermore, it enhances pro-apoptosis expression and blocks anti-apoptosis proteins (FOXO3/Bim/Bax and Bcl-2/Bcl-xL, respectively) [84].

Chaetoglobosin G **27** (Figure 4) is a secondary metabolite in the *Chaetomium globosum* fungus. It possesses antiproliferation activity against lung (NSCLC) cancer A549 cells (MTT assay). The mechanistic study revealed that it enhances the autophagic effect via inhibition of p-EGFR, p-MEK, and p-ERK proteins and incrementally increases the LC3-II protein level. Flow cytometry supports its ability for apoptosis induction and cell cycle arrest at the G2/M phase. Controlling/downregulating cyclin B1 protein and enhancing p21 protein are also reported [85].

Vincamine **28** (Figure 4), isolated from the Vinca minor leaves and used as a diet for aging combat, was reported as an apoptosis inducer. Its antiproliferation properties against the A549 cell line (IC_50_ = 309.7 μM) were mentioned (MTT assay). In addition to the potential change in mitochondrial membrane potential, the potential activity towards ROS and caspase-3 was the mode of action mentioned that supported the anticancer activity revealed [86].

### 2.3. Gastric Cancer

The fifth most common cancer in the world is gastric cancer, which is also known as stomach cancer [87]. Usually, surgery and chemotherapy are the options considered for diagnosed patients with stomach cancer [88].

**Figure 5:**
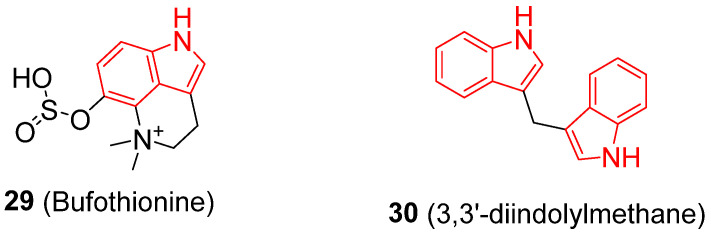
Chemical structure of indole alkaloids **29** and **30** with antiproliferation properties against gastric cancer.

Bufothionine **29** (Figure 5) isolated from the toad *Bufo bufogargarizans* reveals inhibition of the gastric cancer cell lines MKN28 and AGS (CCK-8 assay) with apoptosis induction (supported by flow cytometric analysis). It facilitates caspase-3/8/9 apoptosis in both cell lines in addition to upregulating Bcl-2 and downregulating Bax proteins. In vivo, a gastric cancer xenograft mouse model supported its ability to suppress tumor growth and weight [89]. 

3,3′-Diindolylmethane **30** (Figure 5) obtained from *Cruciferous* plants has been demonstrated to induce ferroptosis in BGC-823 gastric cancer cells through the upregulation of lipid-ROS levels and a decrease in GSH generation [90].

### 2.4. Colorectal Cancer 

The second most frequent cancer-related cause of death in the US and the third one globally is colorectal cancer [91,92]. Recurrence and metastasis reduce the survival rate for this disease [92]. It has been reported that colon polyps are the main cause of the disease, in addition to heredity/family history and colitis [93]. Surgery is the first option for the disease; meanwhile, chemotherapy is appropriate for metastasis [94].

**Figure 6:**
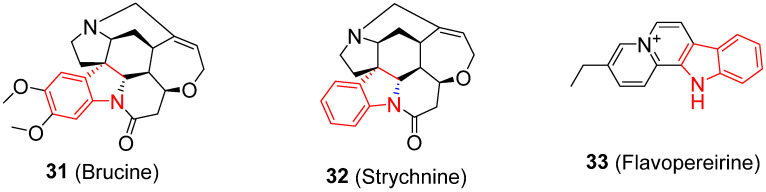
Chemical structure of indole alkaloids **31**–**33** with antiproliferation properties against colorectal cancer.

Brucine **31** and strychnine **32** (Figure 6) were obtained from the seeds of *Strychnos nux-vomica* L., used as a traditional medication for tumor treatment. Brucine and strychnine exhibit inhibitory effects on the growth of human colorectal cancer cells DLD1, SW480, and Lovo (MTT assay). The Wnt/β-catenin singling pathway is involved in the activity since both induce an apoptosis effect through DKK1 and APC expression and downregulate the β-catenin, c-Myc, and p-LRP6 levels. In vivo studies (nude mice) support their effect/suppression of DLD1 tumors [95].

Flavopereirine **33** (Figure 6) is a β-carboline alkaloid extracted from *Geissospermum vellosii*. It affects the viability of different malignant stages of colorectal cell lines (SW480, SW620, DLD1, HCT116, and HT29, with IC_50_ = 15.33, 10.52, 10.76, 8.15, and 9.58 μM, respectively). Its activation of p53 and p21 protein expression justifies the growth suppression and apoptotic cell death of colorectal cancer [96].

### 2.5. Pancreatic Cancer 

Worldwide, pancreatic cancer ranks as the 12th most common male cancer and the 11th most common female cancer [97]. Pancreatic cancer is classified into two categories based on its origin: exocrine or neuroendocrine; the latter is less common but more accessible in prognosis [98].

**Figure 7:**
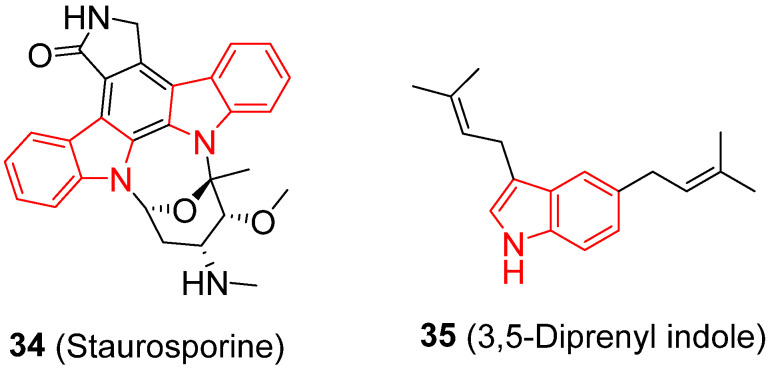
Chemical structure of indole alkaloids **34** and **35** with antiproliferation properties against pancreatic cancer.

Staurosporine **34** (Figure 7), an alkaloid obtained from *Streptomyces staurosporeus*, can induce apoptosis in pancreatic cancer cells (PaTu 8988t and Panc-1). Activation of caspase-9 in both cells was reported (Western blotting analysis). Additionally, both Bcl-2 and Bad expression were mentioned in PaTu 8988T cells [99].

Indole-based alkaloids were obtained from *Ravenia spectabilis Engl.* (leaf extract), revealing noticeable antiproliferation properties against various cancer cell lines, including HeLa, A549, and MIA PaCa-2, with a safety index against the normal cell line WI-38. 3,5-Diprenyl indole **35** (Figure 7) is the most promising cytotoxic agent observed against MIA PaCa-2 (a human pancreatic adenocarcinoma cancer cell line) with an IC_50_ = 9.5 ± 2.2 μM, comparable to the positive drug/control gemcitabine 0.6 ± 0.4 μM (MTT assay) [100].

### 2.6. Liver Cancer

It is the third-most deadly cause of mortality among many cancer types. The chance of its diagnosis is almost three times higher for men than for women [101]. Although surgical resection is an appropriate option for liver cancer patients, its accessibility is limited due to many serious factors, including easy recurrence and metastasis. Chemotherapy is also an important clinical pathway with or without surgery against this disease [102].

**Figure 8:**
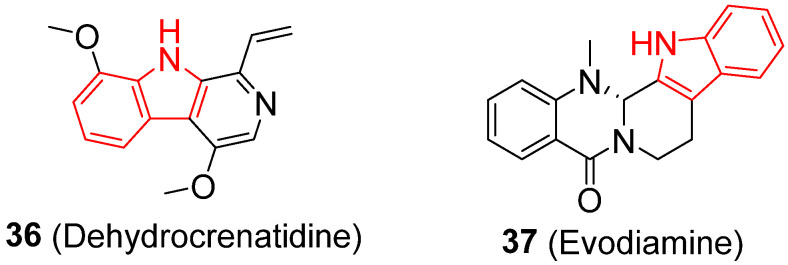
Chemical structure of indole alkaloids **36** and **37** with antiproliferation properties against liver cancer.

Dehydrocrenatidine **36** (Figure 8) is a β-carboline alkaloid isolated from the stem of *Picrasma quassioides*. It exhibits promising growth inhibitory effects against hepatocellular carcinoma in vitro and in vivo, with potent antiproliferation properties (MTT assay) against HepG2 and Hep3B cell lines (IC_50_ = 3.5 and 5.87 μM, respectively). Effects on apoptosis-related proteins such as Bax and Bcl-xl, mitochondrial dysfunction, and a decrease in the mitochondrial membrane were reported to cause apoptosis induction in hepatocellular cancer cells [103].

Evodiamine **37** (Figure 8), obtained from fructus Evodiae, exhibits antiproliferation activity against liver cancer cell lines HepG2 and SMMC-7721 (IC_50_ ≈ 1 μM for both cell lines). Evodiae arrests the cell cycle at G2/M (flow cytometric analysis) and induces apoptosis via upregulation of p53 and Bax, decreasing the Bcl-2, CyclinB1, and cdc2 protein levels. Furthermore, it enhances apoptosis through NOD1 signaling suppression [104]. 

### 2.7. Cervical Cancer

It is one of the most severe cancer diseases in women. It is usually caused by the infection of a specific type(s) of human papillomavirus (HPV) [105,106]. Two types of cervical cancer were identified: ectocervix and endocervix, which are the outer and inner parts of the cervix, respectively [107]. 

**Figure 9:**
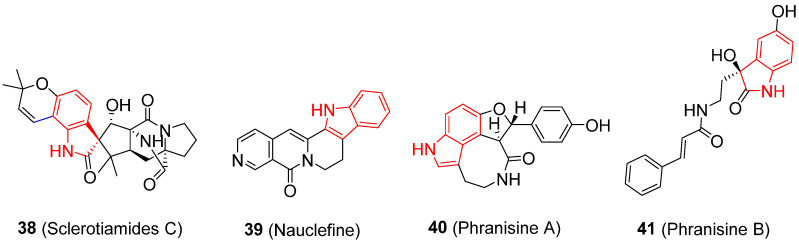
Chemical structure of indole alkaloids **38**–**41** with antiproliferation properties against cervical cancer.

Sclerotiamides C **38** (Figure 9) is a notoamide-type alkaloid obtained from the marine fungus *Aspergillus sclerotiorum*. It has been demonstrated to stop cell division and trigger cell death in HeLa cells via elevation of the phosphorylation of JNK, ERK, and p38. Sclerotiamides C can potentially stimulate the activation of apoptosis-associated proteins, including Cyt-c, Bax, and p53. Demonstrating the MAPK pathway is also mentioned as influencing cell growth and death in HeLa cells [108,109]. 

Nauclefine **39** (Figure 9) is an indolyl alkaloid analog obtained from the bark of *Nauclea subdita* with potent cytotoxicity against HeLa cells (IC_50_ < 10 nM). Additionally, in HeLa cells, nauclefine triggers the PDE3A-SLFN12-dependent (phosphodiesterase family member) pathway, inducing apoptosis [110].

Phranisine A **40** and phranisine B **41** (Figure 9) are natural indolyl alkaloids isolated from the roots of *Phragmites australis*. Both exhibit moderate cytotoxicity against Hela cancer cells, with phranisine A having lower efficacy (IC_50_ = 54 μM) than that of phranisine B (IC_50_ = 19 μM) [111].

### 2.8. Ovarian Cancer 

**Figure 10:**
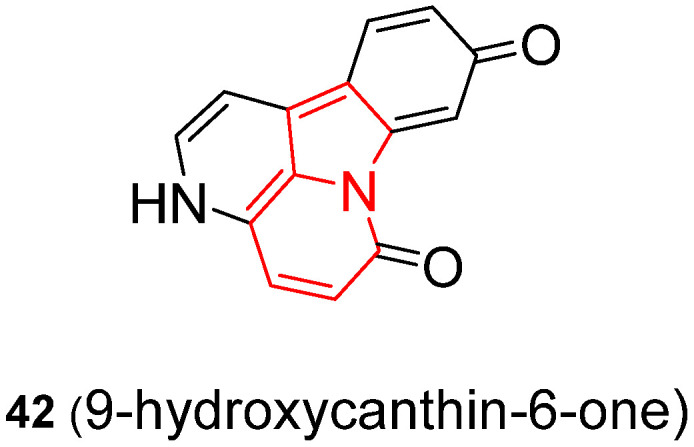
Chemical structure of 9-hydroxycanthin-6-one **42** with antiproliferation properties against ovarian cancer.

The eighth most frequent cancer type in women and the 18th most frequent cancer overall is ovarian cancer [112]. 9-Hydroxycanthin-6-one **42** (Figure 10) is a natural β-carboline alkaloid (isolated from the stem bark of *Ailanthus altissima*), revealing promising antiproliferation properties (MTT assay) against three ovarian cancer cells, including A2780, SKOV3, and OVCAR-3 (IC_50_ = 17.4 ± 1.1, 13.8 ± 0.6, and 18.8 ± 0.7 μM, respectively). It triggers apoptosis by activating caspase-3, -8, and -9, increasing the intercellular ROS-dependent level [113]. 

### 2.9. Leukemia 

Leukemia is one of the most prevalent diseases in children (less than 15 years old) and usually affects elderly individuals [114,115]. Based on the affected white blood cell type, leukemia is divided into two categories/classes: lymphocytic/lymphoid and myeloid, which may be either acute or chronic [116,117]. 

**Figure 11:**
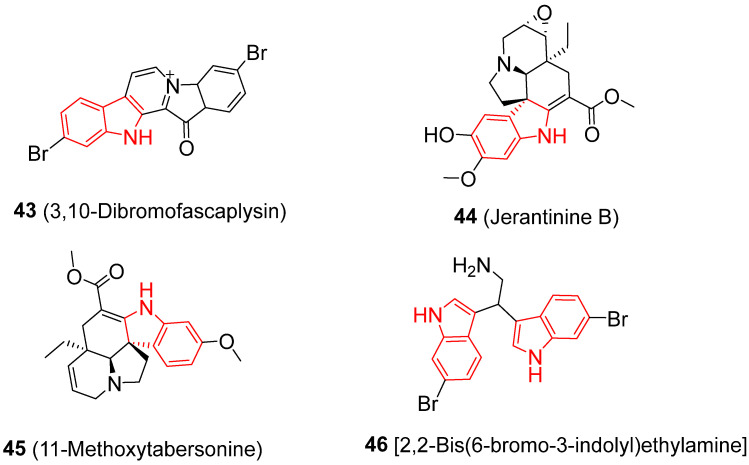
Chemical structure of indole alkaloids **43**–**46** with antiproliferation properties against leukemia.

The marine alkaloid 3,10-dibromofascaplysin **43** (Figure 11) (obtained from *Fascaplysinopsis reticulate*) exerts anticancer activity on several myeloid leukemia cells (K562, THP-1, MV4-11, and U937; IC_50_ = 318.2, 329.6, 233.8, and 318.1 nM, respectively). It induces apoptosis by upregulating the expression of genes encoding the leukemia cell survival proteins, such as E2F1, and by downregulating the expression of FLT3 genes. It can arrest the S and G2 cell cycle phases (9-hydroxycanthin-6-one flow cytometry study) [118].

Jerantinine B **44** (Figure 11) extracted from the *Tabernaemontana corymbosa* leaf reveals potential antiproliferation properties (IC_50_ = 0.3, 0.4, and 0.8 μM against MV4-11, HL-60, and KG1a cells, respectively) and apoptosis in acute myelocytic leukemia cells with activation of the c-Jun/JNK pathway [119]. 

11-Methoxytabersonine **45** (Figure 11), extracted from *Melodinus cochinchinensis,* displays promising antiproliferation properties against acute lymphoblastic leukemia (MOLT-4) and pro-myeloid leukemia (HL-60) cells (IC_50_ = 0.71 and 1.10 μM, respectively). Its antiproliferation properties were attributed to cell death via ROS accumulation and calcium level increases by inhibiting the PI3K/Akt/mTOR pathway in MOLT-4 cells [120].

2,2-Bis(6-bromo-3-indolyl) ethylamine **46** (Figure 11) is found in both Didemnum candidum and the New Caledonian sponge Orina. It induces apoptosis in U937 (human myelomonocytic lymphoma cells) by inhibiting Bcl-2 and Bcl-xL and elevating Bax protein levels [121].

## 3. Synthesized Indoles with Potential Antiproliferation Properties

Synthesized compounds/heterocycles are uniquely positioned in drug discovery programs, providing potent agents and clinically accessible drugs. Many of the synthesized analogs developed are inspired by natural compounds due to the considerable bio-observations revealed. Different medicinal chemical techniques are accessible for designing the targeted hits/leads in addition to the various computational methods, including QSAR, pharmacophoric analysis, docking, and molecular dynamic simulation [122,123,124,125,126,127,128]. 

### 3.1. Breast Cancer

**Scheme 1:**
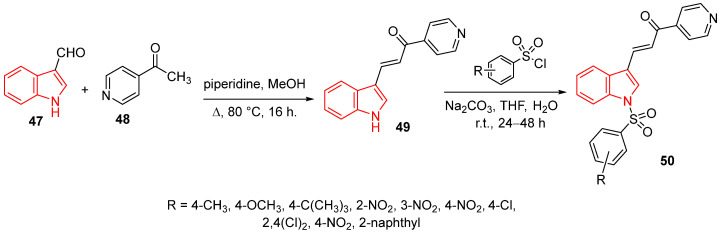
Synthetic route towards pyridyl-indole-based chalcones incorporated in sulfonamide group **50**.

A series of pyridyl-indolyl-based chalcones incorporating the sulfonamide group were synthesized through Knoevenagel condensation of indol-3-carboxaldehyde **47** with 4-acetylpyridine **48** in the presence of piperidine (refluxing MeOH), giving the corresponding chalcone **49**. Treatment of chalcone **49** with sulfonyl chlorides in THF/H_2_O (50%) containing Na_2_CO_3_ (stirring at room temperature) produced the corresponding sulfonamide analogues **50** (Scheme 1). The antiproliferation properties of chalcones **50** were determined against MCF-7 (breast), HepG-2 (hepatoma), and HEK293 (embryonic kidney) cancer cell lines (MTT assay). Among the synthesized agents, two conjugates with R = 2,4-Cl_2_ and 4-NO_2_ possess effective properties against the MCF-7 cancer cell line (IC_50_ = 12.2 and 14.5 μM, respectively), which is more potent than that of the reference drug doxorubicin (IC_50_ = 20.2 μM). These analogs revealed promising antiproliferation properties (IC_50_ = 14.8 and 18.3 μM, respectively) against HepG2 relative to the standard drug, doxorubicin (IC_50_ = 18.7 μM). Significant induced apoptosis in the MCF-7 cancer cell line was reported during the apoptosis assay study. No considerable antiproliferation properties against the HEK293 cell line were noticed by the synthesized agents (IC_50_ > 150 μM). Inhibitory properties against human carbonic anhydrases (hCA IX, hCA II) were experimentally supported as the mode of action of the constructed agents (Supplementary Figure S1). Molecular modeling (Autodock 4.2 software) utilizing PDB ID: 3IAI was considered for explaining the observed enzymatic inhibitory properties [129].

**Scheme 2:**
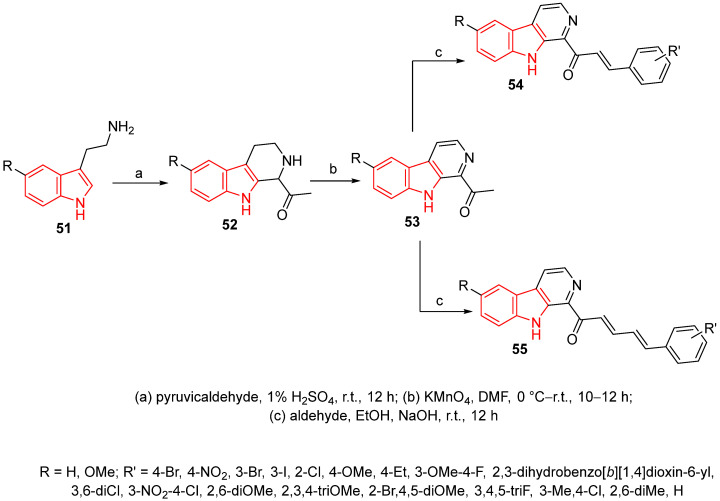
Synthetic route towards harmine–chalcone conjugates **54** and **55**.

Harmine is a natural compound called “9*H*-pyrido[3,4-*b*] indole analog” with potential antitumor properties; however, its clinical accessibility is hindered due to the associated toxicological effects. Conjugation of harmine with chalcone scaffolds was considered for enhancement of antitumor properties and toxicity reduction. The targeted agents **54** and **55** were obtained via condensation of the appropriate aldehyde with the corresponding harmine-based analog **53** in the presence of ethanolic NaOH at room temperature (Scheme 2). Considerable antiproliferation properties of the targeted agents **54** and **55** were investigated against MCF-7, MDA-MB-231 (breast), HepG2 (liver), HT29 (colorectal), A549 (lung), and PC-3 (pancreatic) cancer cell lines and compared with L02 (normal cell line) utilizing the MTT assay (Supplementary Figure S2). The most potent agent observed was **54** (R = H, R’ = 3-NO_2_-4-Cl; IC_50_ = 0.34, 0.98, 1.61, 0.57, 2.02, 1.17, and 9.61 μM, respectively). Induction of apoptosis against MCF7 (breast cancer) was attributed to its ability to decrease Bcl-2 and increase Bax, PARP, and phosphorylated Bim proteins. Additionally, suppression and migration of the breast cancer cell (MCF7) due to downregulation of the MMP-2 protein were mentioned. Inhibition of topoisomerase I was supported and justified as the mode of action against cancer. Molecular docking was used to explain the estimated mode of action relative to that of camptothecin (a co-crystallized ligand of PDB ID: 1T8I, Discovery Studio 2016 software) [130].

**Scheme 3:**
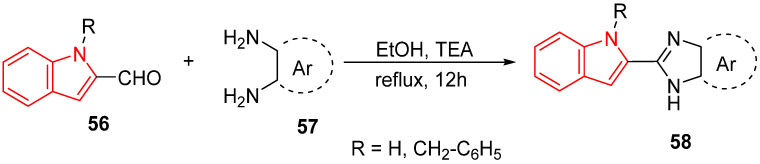
Synthetic route towards indole–benzimidazole derivatives **58**.

A set of indole-benzimidazole conjugates **58** was synthesized as selective estrogen receptor modulators. The targeted compounds were obtained by the cyclocondensation reaction of 1*H*-indole-2-carbaldehyde **56** with different ortho-diamines **57** (EtOH/TEA) [131] (Scheme 3).

**Figure 12:**
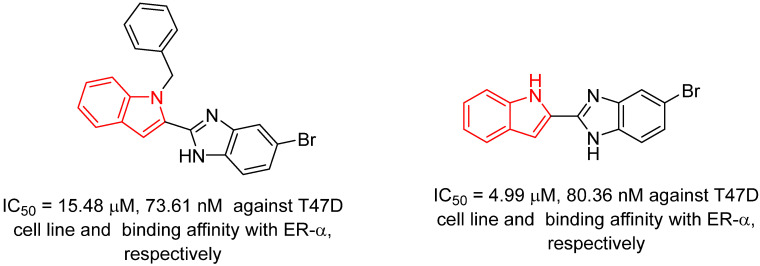
Promising antiproliferative indole–benzimidazole conjugates **58**.

Amongst all the synthesized agents, two bromo-substituted analogs possess promising antiproliferation properties against the estrogen-sensitive breast cancer (T47D) cell line (Figure 12, Supplementary Figure S3). Both conjugates were found to decrease mRNA and ER-α (estrogen receptor-α) activity. The binding activity of both conjugates towards ER-α (PDB ID: 4XI3) was reported to be in the same way as bazedoxifene (an FDA-approved drug to treat osteoporosis and breast cancer, Maestro 9.6 software) [131].

**Scheme 4:**
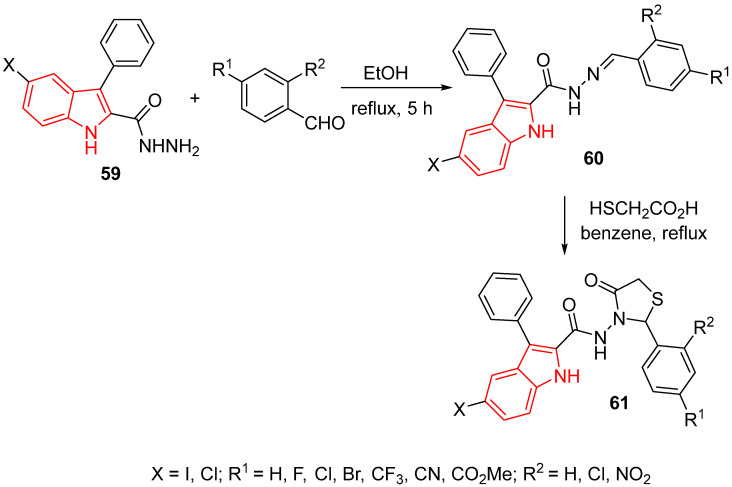
Synthetic route towards indole-2-carbohydrazides **60** and thiazolidines **61**.

Indole-2-carbohydrazones **60** were obtained through a reaction of indole-2-carbohydrazides **59** with the appropriate aromatic aldehyde. The reaction **60** with thioglycolic acid in refluxing benzene afforded the corresponding thiazolidines **61** (Scheme 4). Some of the synthesized hydrazones **60** (X = Cl, R^1^ = CF_3_, R^2^ = H) and (X = Cl, R^1^ = CN, R^2^ = H) showed good antiproliferation properties against the MCF-7 cell line (IC_50_ = 0.42 ± 0.06 and 0.17 ± 0.02 µM, respectively; SRB “sulforhodamine B” assay), relative to the reference standard, combretastatin A-4 (IC_50_ = 0.016 ± 0.003 µM). The tubulin polymerization inhibition revealed by the promising agents discovered (IC_50_ = 1.7 ± 0.6 and 1.4 ± 0.02 μM, respectively) is close to that of the reference standard, combretastatin A-4 (IC_50_ = 1.2 ± 0.08 μM) [132].

**Scheme 5:**
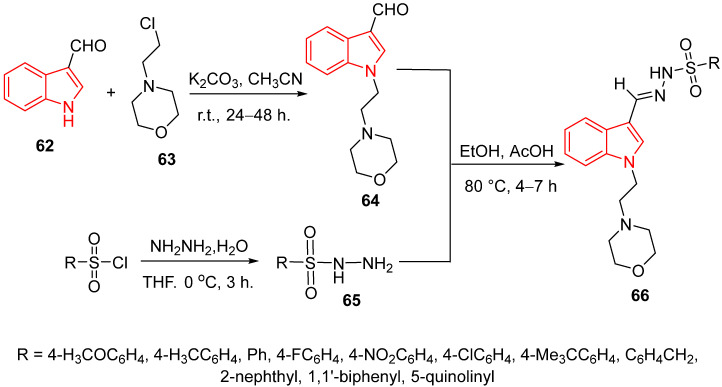
Synthetic route towards indolyl sulfonohydrazones **66**.

Indolyl sulfonohydrazones **66** bearing morpholinyl scaffold were synthesized through a condensation reaction (EtOH/AcOH, 80 °C) of sulfonyl hydrazides **65** with 3-indolecarboxaldehyde **64** (obtained from the reaction of **62** with chloroethyl morpholine **63** in the presence of K_2_CO_3_/CH_3_CN at room temperature) (Scheme 5). Antiproliferative properties were investigated (MTT assay) against MCF7 (estrogen receptor-positive) and MDA-MB-468 (triple-negative) breast cancer cell lines. Some of the synthesized agents revealed considerable anti-breast cancer properties, of which the p-chlorophenyl-containing analog (R = 4-ClC_6_H_4_) showed promising properties (IC_50_ = 13.2 and 8.2 μM against MCF-7 and MDA-MB-468, respectively) compared with doxorubicin (positive drug control, IC_50_ = 0.06 and 0.08 μM, respectively). All the tested compounds behaved safely toward HEK 293, a non-cancer cell, in concentrations up to 100 μM [133] (Supplementary Figure S4).

**Scheme 6:**
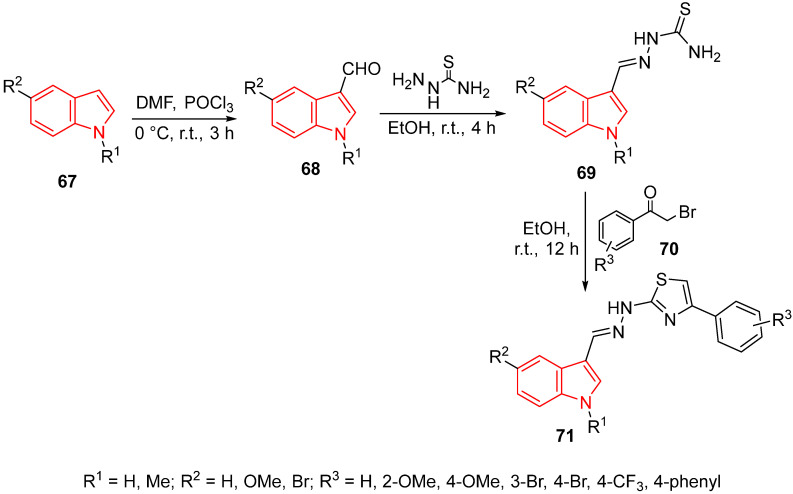
Synthetic route towards thiazolyl hydrazones linked to indolyl scaffold **71**.

Various thiazolyl hydrazones linked to indolyl scaffold **71** were synthesized by reacting the appropriate 3-indolecarboxaldehyde **68** with thiosemicarbazide (EtOH, room temperature). The reaction of the resulting thiosemicarbazones **69** with the appropriate phenacyl bromide **70** produced the targeted hydrazones **71** (Scheme 6). The antiproliferation and tubulin polymerization inhibitory properties of the synthesized agents were studied (Supplementary Figure S5). The most promising agent observed is that of R^1^ = H, R^2^ = OMe, and R^3^ = 3-Br (IC_50_ = 0.46, 0.21, and 0.32 μM against MCF-7 (breast), A549 (lung), and Hela (cervical) cell lines, respectively; with tubulin polymerization inhibitory properties IC_50_ = 1.68 μM) relative to colchicine and combretastatin A-4 “CA-4” (IC_50_ = 0.75, 0.68, and 0.72; 0.52, 0.24, and 0.48 μM against MCF-7, A549, and Hela cell lines; with tubulin polymerization inhibitory properties IC_50_ = 3.28 and 2.12 μM, respectively). Its ability to induce apoptosis and arrest the cell cycle at the G2/M phase was supported by flow cytometric analysis/study. Docking studies (PDB ID: 1SA0, Discovery Studio 3.5 software) were utilized to explain the mode of action considered [134].

**Figure 13:**
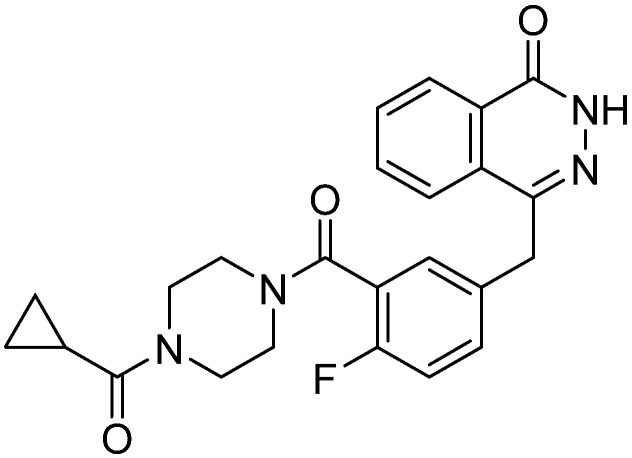
Chemical structure of olaparib (standard drug against PARP-1).

**Scheme 7:**
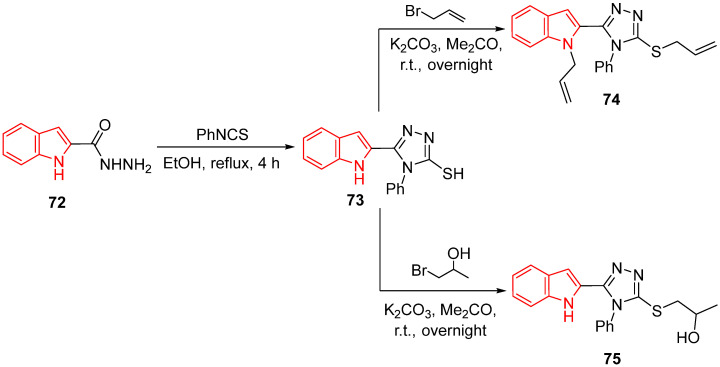
Synthetic route towards indole-triazol congugates **74** and **75**.

Indole-triazole conjugates **74** and **75** were obtained through the reaction of indolyl-triazolethione **73** with allyl bromide and 1-bromopropan-2-ol (stirring in dry Me_2_CO containing K_2_CO_3_ at room temperature overnight), respectively (Scheme 7). Conjugate **75** reveals better activity/inhibitory properties than that of **74** against PARP-1 “poly(ADP-ribose) polymerase-1” (IC_50_ = 0.35 ± 0.05 and 0.33 ± 0.10 μM ± SD, respectively) relative to olaparib (standard reference/drug IC_50_ = 1.8 × 10^−3^ ± 0.0001 μM) (Figure 13). PARP-1 is a key enzyme in DNA repair. It represents an important target in combating oncology in breast cancer cells and is safe against normal cells with lethal mode selectivity [135].

**Scheme 8:**
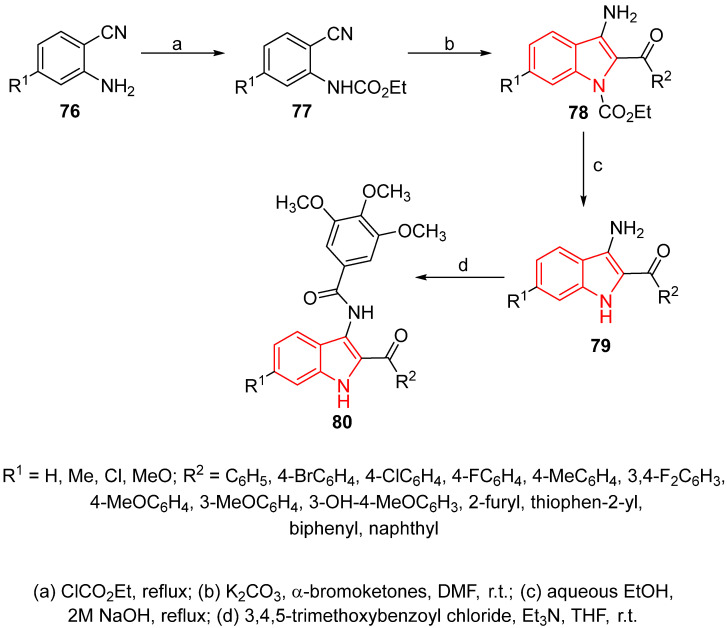
Synthetic route towards 3-amidoindoles **80**.

A short library of 3-amido indoles **80** was synthesized via hydrolysis (NaOH in refluxing aqueous EtOH) of the corresponding 1-ethyl carbonyl indoles **78,** giving the *N*-unsubstituted indoles **79**, followed by acylation with 3,4,5-trimethoxybenzoyl chloride in anhydrous THF containing TEA (triethylamine) at room temperature (Scheme 8). Some of the synthesized agents revealed considerable antiproliferation properties (MTT assay) against breast cancer cell lines MCF-7, MDA-MB-231, BT549, T47D, MDA-MB-468, and HS578T. The most promising is that with R^1^ = Cl, R^2^ = 4-ClC_6_H_4_ displays considerable activity with tubulin polymerization inhibitory properties (IC_50_ = 10.87, 6.43, 3.17, 0.04, and 7.92 μM against MCF-7, MDA-MB-231, BT549, T47D, and MDA-MB-468, respectively; IC_50_ = 9.5 μM against tubulin polymerization) relative to combretastatin A-4 (CA-4, reference agent, IC_50_ = 3.00, 3.17, 1.71, 1.89, and 1.55 nM against MCF-7, MDA-MB-231, BT549, T47D, and MDA-MB-468, respectively; IC_50_ = 4.22 μM against tubulin polymerization) (Supplementary Figure S6). Its flow cytometric studies evidenced the cell cycle arrest at the G2/M phase. Molecular docking studies (PDB ID: 5lyj; SURFLEX module of SYBYL 7.3) revealed its interaction in the colchicine binding active site [136].

**Scheme 9:**
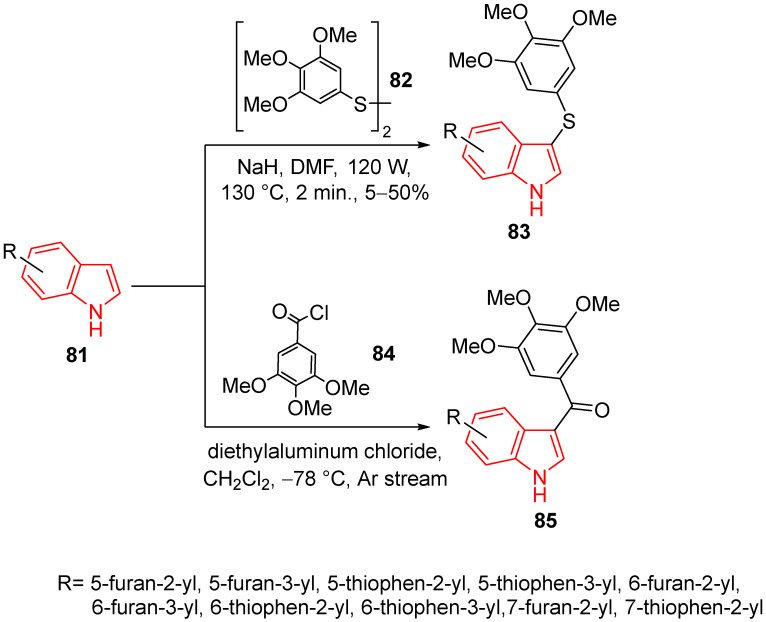
Synthetic route towards 3-arylthio- **83** and 3-aroyl-1*H*-indoles **85**.

3-Arylthio-1*H*-indoles **83** bearing heterocyclic rings at positions 5, 6, or 7 of the indolyl nucleus were synthesized through the reaction of the appropriate indole **81** with bis(3,4,5-trimethoxyphenyl)disulfide **82** in anhydrous DMF containing NaH (microwave “MW” radiation, 120 W, 130 °C) (Scheme 9). Potent antiproliferative properties against MCF-7 (a non-metastatic breast cancer cell line, MTT assay) were exhibited (IC_50_ in nanomolar value). Compounds **83,** where R = 6-thiophen-3-yl and 7-thiophen-2-yl, are the most potent agents revealed (IC_50_ = 4.5 and 29 nM, respectively) relative to the reference drug CA-4 (IC_50_ = 13 nM). Additionally, tubulin polymerization inhibition is promising (IC_50_ = 0.58 and 0.57 μM, respectively) compared to CA-4 (IC_50_ = 1.0 μM).

The role of the sulfur bridging atom was studied by constructing an **85**-containing carbonyl function. The 3-aroyl-1*H*-indoles **85** were obtained through a reaction of the appropriate indole **81** with 3,4,5-trimethoxybenzoyl chloride **84** in the presence of diethylaluminum chloride in CH_2_Cl_2_ (inert atmosphere at −78 °C). Although promising antiproliferation properties were observed by some of the synthesized agents against the MCF-7 cell line, a dramatic drop was exhibited due to the analogs with sulfur bridging mentioned upon utilizing carbonyl function (IC_50_ = 18 and 550 nM for R = 6-thiophen-3-yl and 7-thiophen-2-yl, respectively) (Supplementary Figure S7). Molecular docking studies (PDB ID: 1SA0) were considered for compounds with potent tubulin polymerization inhibition for understanding and explaining the mode of action shown [137].

**Scheme 10:**
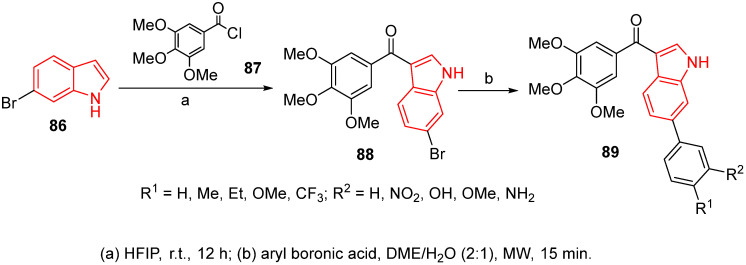
Synthesitic route towards 6-aryl-3-aroyl-indoles **89**.

Friedel-Craft acylation of 6-bromoindole **86** using 3,4,5-trimethoxylbenzoyl chloride **87** afforded the corresponding 3-aroyl indole **88** (HFIP (hexafluoroisopropanol) at room temperature is an adequate condition for inter- and intramolecular Friedel-Craft acylation) [138,139]. The Suzuki coupling reaction of **88** with various aryl boronic acids produced the targeted 6-aryl indoles **89** in DME (dimethoxyethane)/H_2_O under microwave irradiation conditions [138] (Scheme 10). Antiproliferation properties (SRB assay) and inhibitory tubulin polymerization against breast cancer cell lines (MCF-7 and MDA-MB-231) were observed for the targeted agents **89** relative to those of CA-4 (Supplementary Figure S8). The most promising analog (R^1^ = H, R^2^ = OH) discovered can arrest the cell cycle at the G2/M phase in the MDA-MB-231 cell (flow cytometry), disrupt the microtubule structure, and inhibit cell migration. Molecular docking studies revealed valuable insights regarding key interactions towards the colchicine site (PDB ID: 1SA0, Discovery Studio 4.5 software) [138].

**Scheme 11:**
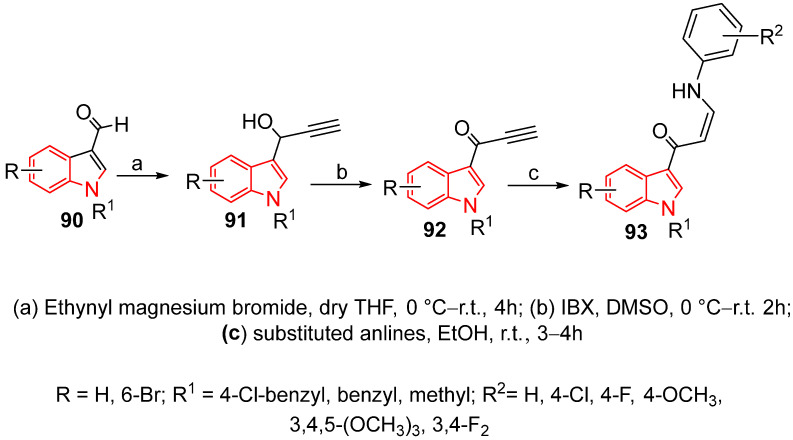
Synthetic route towards indolyl-arylaminopropenone conjugates **93**.

Molecular conjugation is an important and famous approach intensively used in medical chemistry for designing/optimizing highly promising hits/leads against different diseases. This usually takes place by connecting biologically active functional group(s) and/or scaffold(s) to each other with or without a linker [140,141,142,143,144]. Indolyl-arylaminopropenone conjugates **93** were prepared by reacting indole-3-carboxaldehydes **90** with ethynyl magnesium bromide, producing the corresponding arylprop-2-yn-1-ols **91**. Oxidation of the latter alcohols using 2-iodoxybenzoic acid (IBX) in DMSO yielded the corresponding alkynes **92**, which were subjected to reaction with various anilines (EtOH, r.t.), giving the targeted conjugates indolyl-arylaminopropenones **93** (Scheme 11). The antiproliferation properties (MTT assay) of **93** were determined against MCF-7, HeLa, A549, and DU145 (breast, cervical, lung, and prostate cell lines, respectively). Among them, synthesized conjugates [R = H, R^1^ = 4-chlorobenzyl, R^2^ = 3,4,5-(OMe)_3_] and [R = H, R^1^ = benzyl, R^2^ = 3,4,5-(OMe)_3_] exhibited considerable properties against the MCF-7 cell line (IC_50_ = 2.3 and 1.9 μM, respectively) relative to doxorubicin (IC_50_ = 0.8 μM) (Supplementary Figure S9). Both compounds showed cell cycle arrest at G0/G1 (flow cytometry) and induction of cell death apoptosis. Molecular docking (PDB ID: 4AQ3, Schrodinger suite 2014–3) observations of the most promising agents discovered support the Bcl-2 protein (anti-apoptotic protein) interactions and bio-properties revealed [145].

**Scheme 12:**
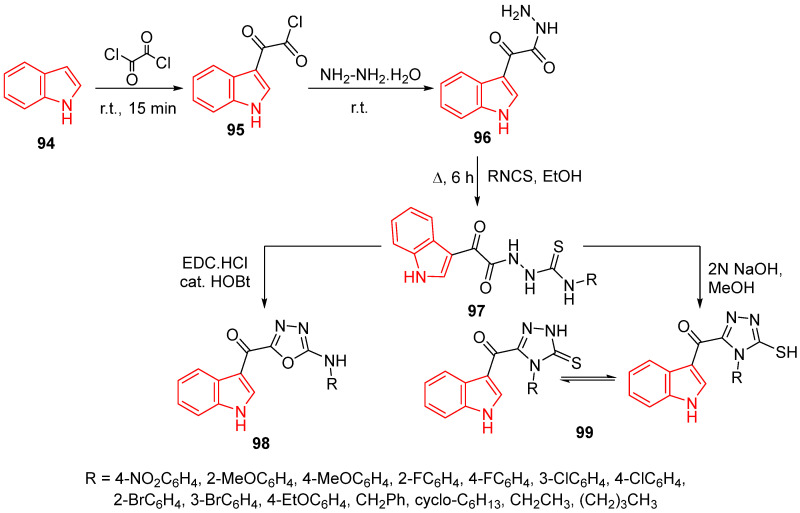
Synthetic route towards 1,3,4-oxadiazole-indole **98** and 1,3,4-triazole-indole conjugates **99**.

**Figure 14:**
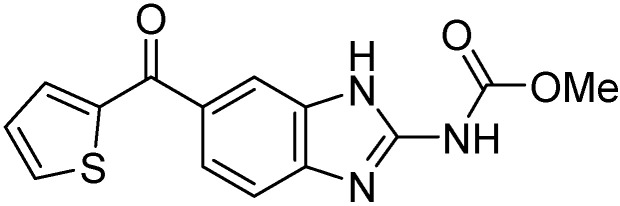
Chemical structure of nocodazole, an antineoplastic agent that exerts its activity by interfering with the polymerization of microtubules.

1,3,4-Oxadiazole-indole **98** and 1,3,4-triazole-indole conjugates **99** were synthesized in a multi-step reaction sequence. 3-Indolyl-2-oxoacetyl chloride **95** was obtained from the reaction of indole **94** and oxalyl chloride, which was further subjected to the reaction with hydrazine hydrate, yielding the corresponding oxoacetohydrazide **96**. Refluxing the hydrazide **96** with isothiocyanates produced the corresponding thiosemicarbazides **97**. Cyclization of the latter with either EDC.HCl (*N*-ethyl-*N’*-(3-dimethylaminopropyl)carbodiimide hydrochloride) or HOBt (hydroxybenzotriazole) produced the 1,3,4-oxadiazole-indole conjugates **98**. However, cyclization of **97** with 2N NaOH afforded the corresponding 1,3,4-triazole-indole conjugates **99** (Scheme 12). Antiproliferation properties (MTT assay) revealed the promising anti-MCF-7 activity of some synthesized oxadiazole **98** (R = 4-NO_2_C_6_H_4_, 2-FC_6_H_4_, and 3-ClC_6_H_4_; IC_50_ = 5.98, 2.42, and 8.11 μM, respectively) and triazole conjugates **99** (R = 4-FC_6_H_4_ and 3-BrC_6_H_4_; IC_50_ = 3.06 and 3.30 μM, respectively) relative to doxorubicin and CA-4 (IC_50_ = 6.31 and 2.16 μM, respectively). Furthermore, the potent synthesized oxadiazole hybrid **98** (R = 2-FC_6_H_4_) shows cell cycle arrest in the G0/G1 phase (flow cytometry), disruption of the mitochondrial membrane, and reduction in cell migration. Additionally, tubulin polymerization inhibitory properties (IC_50_ = 3.89 μM) relative to those of nocodazole (IC_50_ = 2.49 μM) (Figure 14) were supported by in vitro studies. Molecular modeling studies (PDB ID: 1SA0) were utilized to explain the β-tubulin and antiproliferation properties [146].

**Figure 15:**
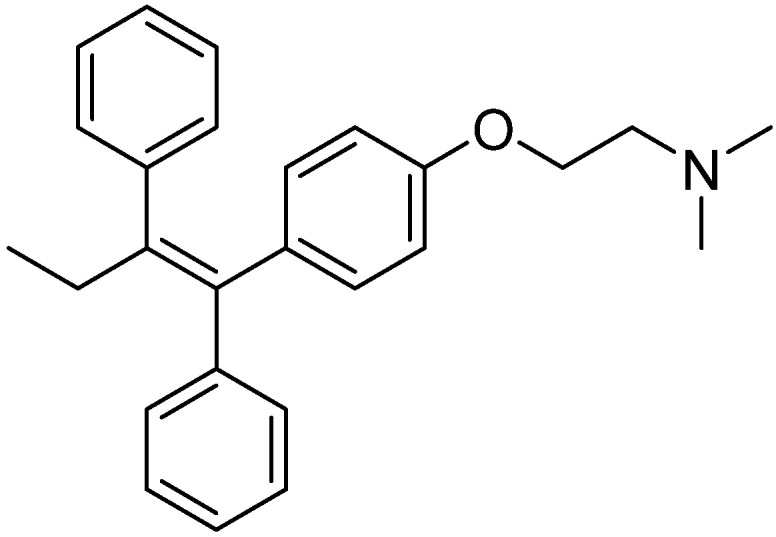
Tamoxifen (an approved drug for breast cancer).

**Scheme 13:**
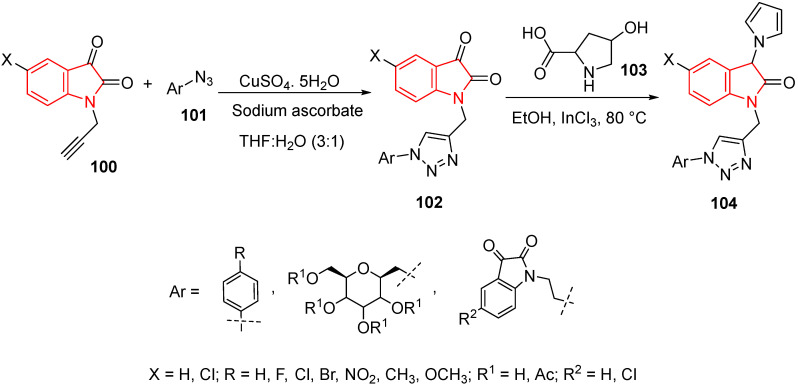
Synthetic route towards 3-pyrrolylisatin-triazole conjugates **104**.

A set of 3-pyrrolylisatin-triazole conjugates **104** was obtained through the reaction of 4-hydroxyproline **103** with 1,2,3-triazole-isatine analogs **102** (obtained from the click reaction of *N*-indole alkynes **100** with substituted azides **101**) in EtOH (80 °C) containing InCl_3_ (indium (III) chloride) as a Lewis acid catalyst (Scheme 13). Antiproliferative properties (MTT assay) of the targeted agents **104** against breast cancer (MCF-7 and MDA-MB-231) cell lines demonstrated that some of them have more potent activity than that of tamoxifen (an approved drug for breast cancer treatment, Figure 15), with similar behavior against the normal cell line HEK-293 (human embryonic) (Supplementary Figure S10). Molecular docking studies have evidenced the potential binding interaction of the potent agents synthesized and tamoxifen with topoisomerase II (PDB ID: 1ZXM) [147].

**Scheme 14:**
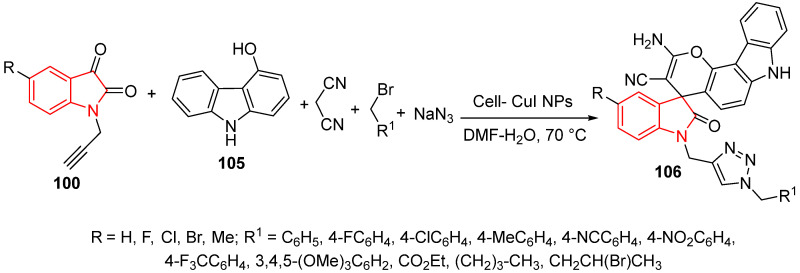
Synthetic route towards spirochromenocarbazols linked to 1,2,3-triazole **106**.

Spirochromenocarbazols linked to 1,2,3-triazole **106** were obtained through a multi-component click reaction of *N*-propargyl isatin **100**, malononitrile, 4-hydroxycarbazole **105**, sodium azide, and alkyl bromides using Cell-CuI NPs (cellulose-supported CuI nanoparticles) catalysis in DMF-H_2_O (1:2 *v*/*v*) at 70 °C (Scheme 14). The antiproliferation properties (MTT assay) were determined against MCF7, MDA-MB-231 (breast), HeLa (cervical), A549 (lung), PANC-1 (pancreatic), and THP-1 (leukemia) cell lines (Supplementary Figure S11). Some synthesized spiro-analogs showed promising antiproliferative properties against MCF-7, MDA-MB-231, and HeLa cancer cells. The most effective agents are those with R = H and R^1^ = 4-NO_2_C_6_H_5_ (IC_50_ = 2.13 µM), revealing more enhanced properties than those of doxorubicin (IC_50_ = 4.63 µM) against MCF7, with a satisfied safety profile towards HUVEC (umbilical vein endothelial/non-cancerous cell). Apoptotic cell death was suggested to be the leading cause of the reduced proliferation of breast cancer cells, which was supported by AO (acridine orange)/EtBrz (ethidium bromide) stains and fluorescence microscopy [148].

**Scheme 15:**
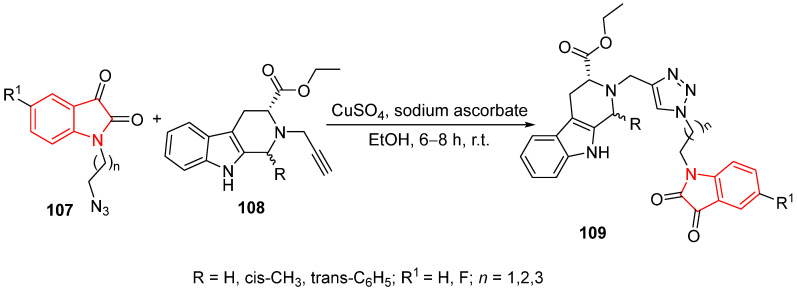
Synthetic route towards 1*H*-1,2,3-triazole connecting tetrahydro-β-carboline and isatin scaffolds **109**.

A series comprising tetrahydro-β-carboline and isatin scaffolds connected by 1*H*-1,2,3-triazolyl heterocycle **109** was synthesized through click cycloaddition of the azide-alkyne isatins **107** and the corresponding carboline **108** in the presence of CuSO_4_/sodium ascorbate in EtOH at room temperature (Scheme 15). The antiproliferation properties (MTT) of **109** were studied against MCF-7 and MDA-MB-231 cell lines (Supplementary Figure S12). Few of the synthesized agents revealed promising antitumor properties against MCF7. The most promising is that with R = R^1^ = H, *n* = 2 (IC_50_ = 37.42 μM) relative to peganumine A (β-carboline analog, obtained from *Peganum harmala*) and tamoxifen (IC_50_ = 38.5 and 50 μM, respectively). The docking study (PDB ID: 3ERT, Autodock Vina software, V 1.5.6) explained the bio-properties exhibited [149].

**Scheme 16:**
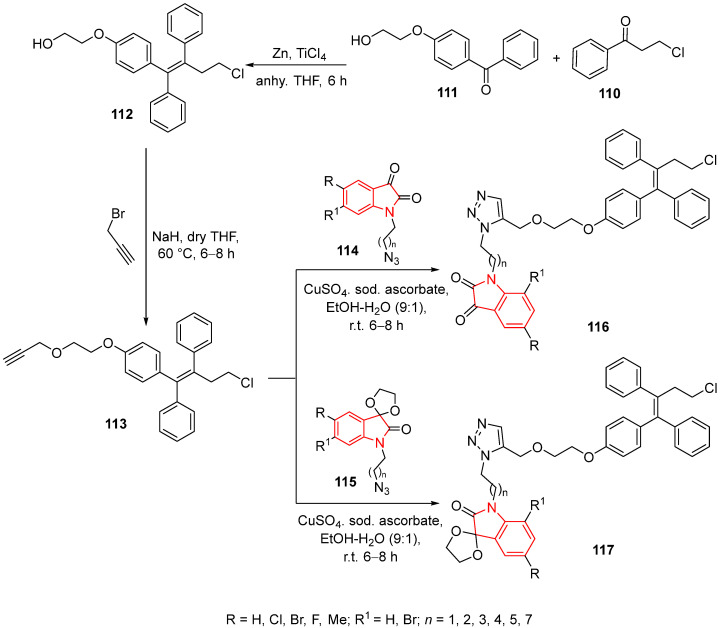
Synthetic route towards ospemifene-isatin/ospemfene-spiroisatin **116**/**117**, linked through 1*H*-1,2,3-triazole, respectively.

A group of ospemifene-isatins **116** and ospemifene-spiroisatins **117** conjugates linked through a 1*H*-1,2,3-triazolyl heterocycle was synthesized via click cycloaddition (CuSO_4_, sodium ascorbate in EtOH/H_2_O) of the appropriate azide-containing indoles **114**/**115** with alkynes containing ospemifene **113** (Scheme 16). Antiproliferation properties (MTT assay) were studied against breast cancer (MCF-7 and MDA-MB-231) cell lines. Some of the synthesized conjugates revealed considerable anti-MCF7 properties. The most promising is the conjugate **116** (R = R^1^ = Br, *n* = 1; IC_50_ = 1.56 µM) relative to that of the standard references (IC_50_ = 55 and 50 μM of ospemifene and tamoxifen, respectively). It has been noticed that when a more extended spacer/alkyl group was considered (*n* = 2 or 3), the anti-MCF-7 properties were drastically reduced (IC_50_ = 16.54 and 10.99 µM, respectively) (Supplementary Figure S13). Molecular docking studies (PDB ID: 3ERT, ERα active site, Autodock Vina software V 1.5.6) explained the biological properties exhibited [150].

**Scheme 17:**
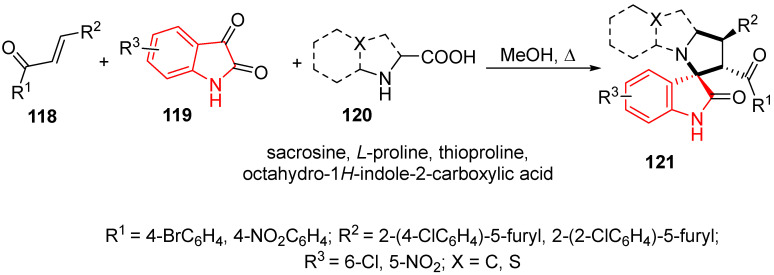
Synthetic route towards spirooxindoles **121**.

**Figure 16:**
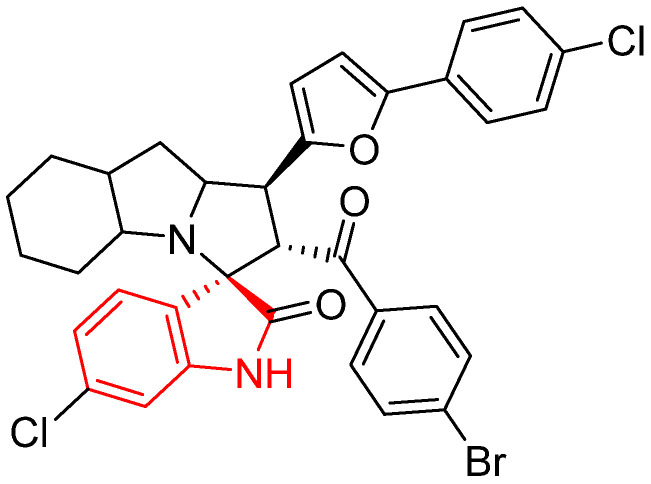
Most potent spiroxindole **121** aganist the MCF7 cell line.

A series of spiroxindoles bearing 2-furanyl heterocycle **121**, prepared from the azomethine ylide reaction (obtained from isatins **119** and amino acids **120**) with furanyl-containing chalcones **118** in refluxing MeOH (Scheme 17), showed promising results. The antiproliferation properties (MTT technique) of **121** were assessed against the MCF7 cell line. Amongst all, the analog derived from chalcone with R^1^ = 4-BrC_6_H_4_, R^2^ = 2-(4-ClC_6_H_4_)-5-furyl, 6-chloroisatin, and octahydro-1*H*-indole-2-carboxylic acid (Figure 16) exhibited potent activity (IC_50_ = 4.3 μM/mL) compared with the standard staurosporine (IC_50_ = 17.8 μM/mL) (Supplementary Figure S14). The molecular modeling of the potent agent suggested a dual mode of action against EGFR and CDK-2 (PDB ID: 1M17 and 2A4L, respectively; AutoDock Vina software V 1.5.6) [151], indicating the potential for further development.

**Scheme 18:**
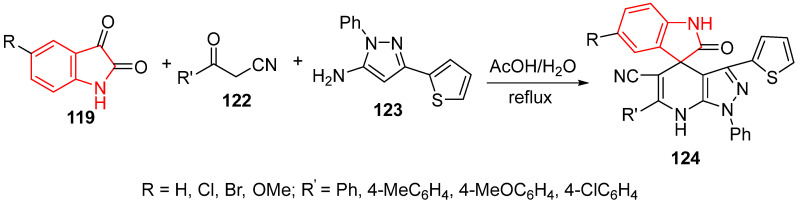
Synthetic route towards spiroxindoles **124**.

Spiroxindoles **124** were obtained through a multi-component condensation reaction of isatins **119**, aroylacetonitriles **122**, and 5-aminopyrazole **123** (Scheme 18). Some targeted agents **124** exhibited mild antiproliferation properties (MTT assay) against the MDA-MB-231 cell line (Supplementary Figure S15). The most promising are those with R/R’ = H/Ph, Cl/Ph, and Br/Ph (IC_50_ = 6.70, 6.40, and 6.70 μM, respectively) relative to doxorubicin (adriamycin, IC_50_ = 0.12 μM). Safety behavior against WI-38 (lung normal cell) was evidenced for the effective agents discovered (IC_50_ = 78.1, 43.2, and 39.3 μM for compounds **124** with R/R’ = H/Ph, Cl/Ph, and Br/Ph, respectively). Upregulation of Bax and downregulation of Bcl-2 proteins in addition to elevation of caspase-3 levels evidenced the induction of apoptosis of the effective agents discovered (effect = 405.5, 353.7, and 0.80; 0.3958, 0.7449, and 2.692; 0.3501, 0.4058, and 0.0111 pg/mL for compounds **124** with R/R’ = H/Ph and Cl/Ph against Bax, Bcl-2, and caspase-3, respectively). Inhibition of EGFR was reported as the mode of action for the promising agents discovered relative to erlotinib [152].

**Scheme 19:**
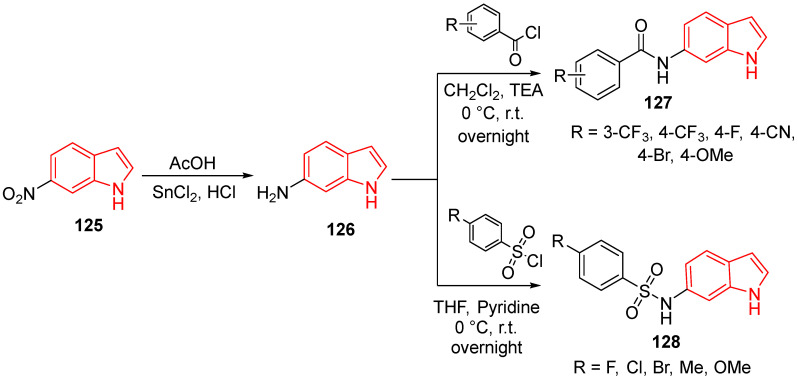
Synthetic route towards *N*-(1*H*-indole-6-yl)benzamides/benzene sulfonamides **127** and **128**.

*N*-(1*H*-indole-6-yl) benzamides **127** and their benzene sulfonamide analogs **128** were obtained through acylation/sulfonylation of 6-aminoindole **126**. The latter was synthesized via reduction (SnCl_2_/HCl/AcOH) of the corresponding 6-nitroindole **125** (Scheme 19). Cell viability assays of the synthesized compounds against breast cancer cell lines (MCF7 and T47D) were studied (Supplementary Figure S16). The most promising was **127**, where R = 3-CF_3_ (IC_50_ = 28.23 and 30.63 μM) relative to tamoxifen (IC_50_ = 34.42 and 42.40 μM) against the T47D and MCF7 cell lines, respectively. A reduction in tumor size in Ehrlich ascites carcinoma (EAC)-bearing mice was observed by compounds **127** (R = 3-CF_3_) and **128** (R = F), supporting their potential necrosis effect and decrease in ER-α expression in tumor sections [153].

**Scheme 20:**
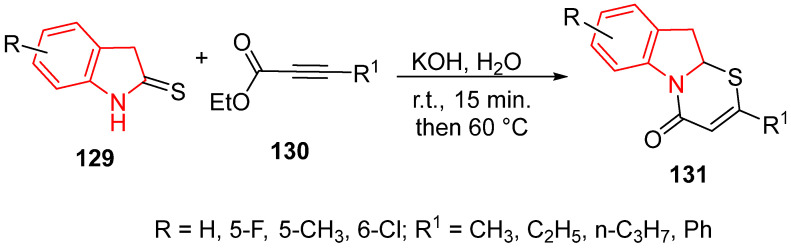
Synthetic route towards 4*H*-[1,3]thiazino[3,2-*a*]indol-4-ones **131**.

[1,3]Thiazino[3,2-*a*]indol-4-ones **131** were obtained from the reaction of indoline-2-thiones **129** and propiolic acid esters **130** in aqueous medium by KOH/H_2_O (Scheme 20). The antiproliferative properties (MTT assay) against the MDA 231 and MDA 468 cell lines were studied (Supplementary Figure S17). Two of the synthesized agents (R/R^1^ = H/CH_3_ and 5-CH_3_/n-C_3_H_7_, IC_50_ = 302 and 116; 330 and 97 μM against MDA-231 and MDA-468, respectively) displayed considerable antiproliferation properties [154].

**Scheme 21:**
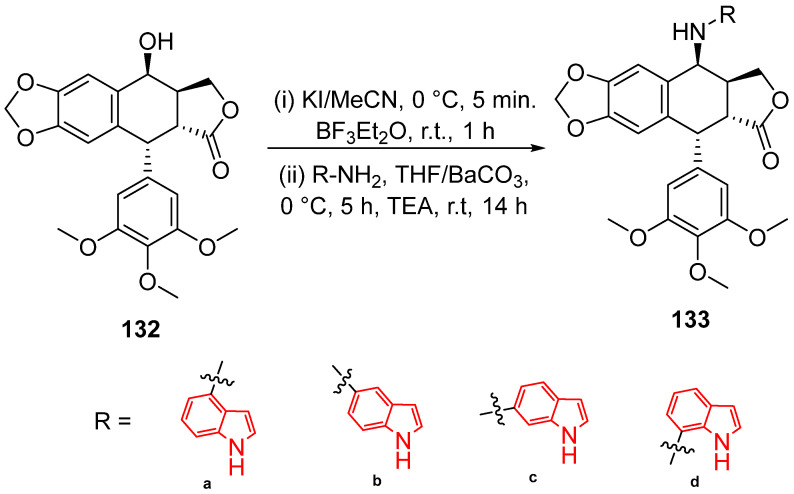
Synthetic route towards indole-podophyllotoxin conjugates **133**.

Podophyllotoxin **132** is an important agent with antiproliferation properties against diverse cancer cell lines, exhibiting affinity at the colchicine binding site and identifying tubulin polymerization inhibitory properties. A series of indole-podophyllotoxin conjugates **133** was developed via the halogenation reaction of **132** using KI and BF_3_OEt_2_ in MeCN, affording the 4β-iodopodophyllotoxin, which was subjected to nucleophilic attack of the indolyl derivative using BaCO_3_ and triethylamine (TEA) in tetrahydrofuran (THF), affording the targeted conjugates **133** (Scheme 21). Potent tubulin polymerization inhibition was revealed by **133c** (GI_50_ < 0.1 μM). Moreover, **133c** displayed outstanding antiproliferation properties (MTT method) against HepG-2, HeLa, A549, and MCF-7 cell lines (IC_50_ = 0.07–0.1 μM) relative to nocodazole (IC_50_ = 0.2–0.4 μM) (Supplementary Figure S18). In vivo studies demonstrated that **133c** reduced tumor volume in the nude mouse xenograft MCF-7 cell model, supporting the idea that it can be considered a promising viable anticancer agent with tubulin polymerization inhibitory properties. Molecular docking studies (PDB ID: 5JCB, Discovery Studio software) were considered to explain the observed mode of action [155].

### 3.2. Lung Cancer

**Scheme 22:**
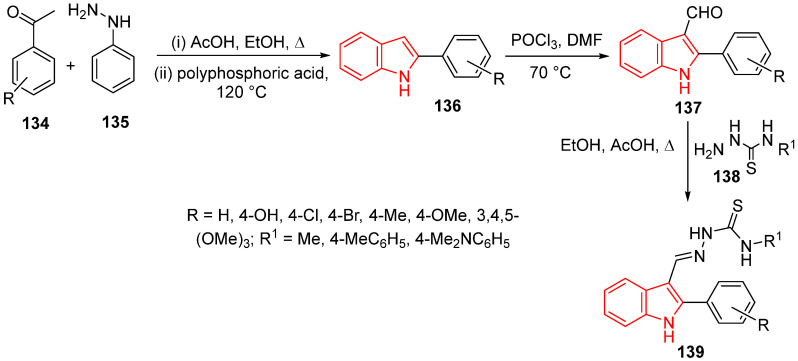
Synthetic route towards indolylthiosemicarbazones **139**.

Indolylthiosemicarbazones **139** were obtained through condensation of indole-3-carboxaldehydes **137** (obtained from the Fischer reaction of acetophenones **134** with phenyl hydrazine **135** followed by the Vilsmeier formylation reaction) with the appropriate thiosemicarbazides **138** (Scheme 22). One of the synthesized agents **139** (R = 4-OMe, R^1^ = Me) revealed potent antiproliferation properties (MTT method) against the lung A549 cell line (IC_50_ = 12.50 μM), i.e., about three-fold more potency than the reference drug etoposide (IC_50_ = 34.25 μM) (Supplementary Figure S19). Apoptosis induction was reported for the potent agent discovered based on morphological and flow cytometric studies. Molecular modeling studies (PDB ID: 1S0 and 1ZXM, Discovery Studio 4.1 software) were considered for assigning the tubulin polymerization and topoisomerase II inhibitory properties, respectively [156].

**Scheme 23:**
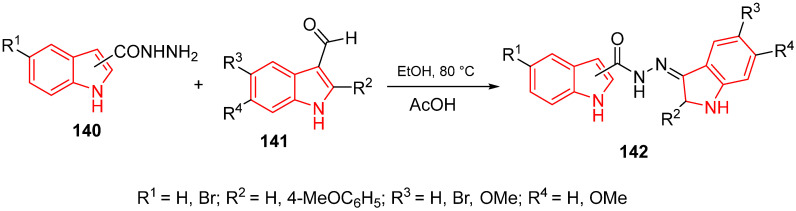
Synthetic route towards bis(indolyl)hydrazide-hydrazones **142**.

Microtubule assembly plays a crucial role in cellular division. For this reason, anti-tubulin/microtubule polymerization inhibition is one of the most effective approaches for combating many cancer types. Bis(indolyl)hydrazide-hydrazones **142** as tubulin polymerization inhibitors were designed. The targeted agents were obtained by refluxing a mixture of indolylcarboxylic acid hydrazides **140** with indole-3-carboxaldehydes **141** in EtOH containing a catalytic amount of AcOH (Scheme 23). The antiproliferation properties (MTT method) of **142** were evaluated against the lung cancer (A549) cell line, revealing that the compound with R^1^ = R^2^ = R^4^ = H and R^3^ = OMe was the most effective analog relative to colchicine (IC_50_ = 2 and 0.02 μM, respectively) in arresting the cell cycle at the G2/M phase (flow cytometry) and tubulin polymerization inhibition (IC_50_ ∼ 7.5 μM) [157] (Supplementary Figure S20).

**Scheme 24:**
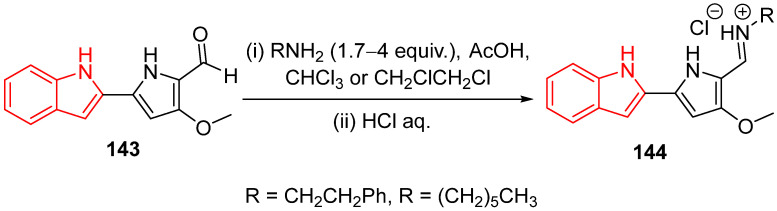
Synthetic route towards indole-based tambjamine analogs **144**.

Tambjamine is a natural compound obtained from marine invertebrates with the ability to compromise cell survival. Indole-based tambjamine analogs **144** were synthesized as natural-based antitumor active agents by condensing the aldehydic analog **143** with the appropriate amine (Scheme 24). A potent inhibitory effect of the synthesized analogs against lung cancer cell lines relative to cisplatin was observed (Supplementary Figure S21). The synthesized compounds introduced several gene expressions demonstrating induced cell death/apoptosis in addition to ROS (reactive oxygen species)-induced cellular stress [158]. It has also been mentioned that **144** with R = (CH_2_)_5_CH_3_ can block Janus kinase/signal transducers, supported by a reduction in survivin protein levels and confirming the potential anti-lung efficacy through STAT3 inhibition [159].

**Scheme 25:**
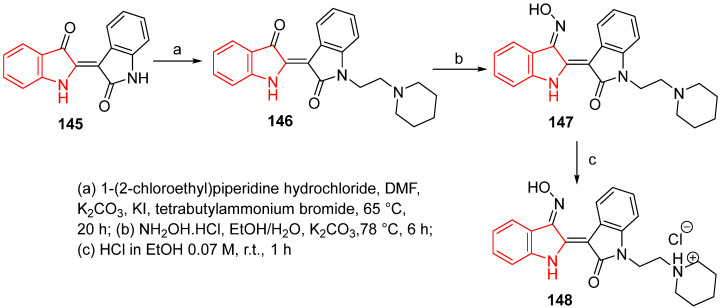
Synthetic route towards indirubin-piperidine conjugate **146** and its HCl salt **147**.

Indirubin **145** is a natural compound with potential anti-leukemia activity in many plants and some protein kinase (CDK and GSK-3β) inhibitory properties. Indirubin-piperidine conjugate **147** was synthesized via alkylation of **145** with 1-(2-chloroethyl)piperidine HCl, followed by condensation with NH_2_OH-HCl. The HCl salt **148** was formed by the effect of EtOH/HCl on **147** (Scheme 25). Promising antiproliferation properties were revealed by the synthesized conjugate **147** and its HCl salt **148** against SW480, A549, HepG2, and B16F10 (colorectal, lung, liver, and melanoma cell lines, respectively; MTT technique) relative to bortezomib (Supplementary Figure S22). A better or more enhanced tumor reduction was exhibited through the in vivo testing (mouse model with skin cancer) of **148** compared to the standard (bortezomib) [160].

**Figure 17:**
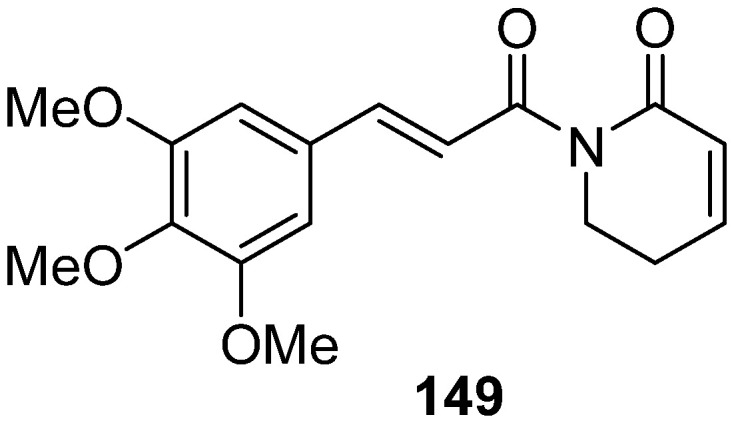
Chemical strucure of piperlongumine **149**.

**Scheme 26:**
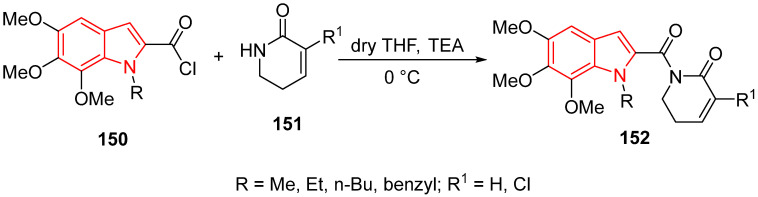
Synthetic route towards indole-piperlongumine conjugates **152**.

Piperlongumine **149** is a natural alkaloid found in *Piper longum* L. with various biological properties (Figure 17). Conjugation of indolyl scaffold with the pharmacophoric unit of piperlongumine was considered for assigning promising antitumor active agents. The reaction of acyl chlorides **150** (obtained from the action of oxalyl chloride on the corresponding carboxylic acids) with lactams **151** (dry THF, TEA, 0 °C) produced the targeted conjugates **152** (Scheme 26) [161]. In vitro, cytotoxicity against A54, HCT116, ZR-75-30, and MDAMB-231 (lung, colon, breast ductal, and breast carcinoma, respectively) in addition to MRC-5 (normal) cell lines was studied (Supplementary Figure S23). Enhanced antiproliferation properties (MTT method) were noticed by the synthesized analogs **152** relative to the precursor piperlongumine **149,** with safe behavior against the normal lung cell line (MRC-5). The most promising agents synthesized are R = Me and R^1^ = Cl, which exhibit induced apoptosis against the lung (A549) cancer cell line (flow cytometry), arresting the cell cycle at the G2/M phase. Furthermore, in vivo studies (BALB/C mice with lung cancer, A549 cells) of the promising agent (2 mg/kg/day, i.p., 14 days) revealed inhibition of tumor growth/volume (54.6%) compared with the parent piperlongumine **149** and doxorubicin (38.3 and 53.3%, utilizing 2 and 10 mg/kg/day for **149** and doxorubicin, respectively) [161].

**Scheme 27:**
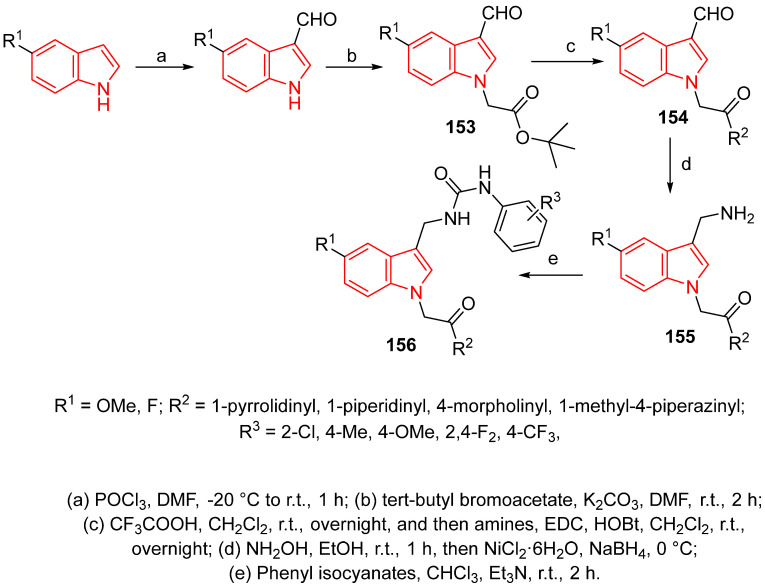
Synthetic route towards indoleyl analogs linked to urea function **156**.

Discoidin domain receptors (DDRs), like many tyrosine kinases (TKs), have a unique place in cancer chemotherapy due to their role in cellular proliferation/differentiation. Inhibition of DDRs is an effective pathway for controlling many diseases, including cancer. A group of indole-containing compounds linked to urea function **156** was designed as inhibitors of DDRs employing virtual screening (molecular docking, PDB ID: 4CKR). The targeted agents **156** were prepared in a multi-step reaction sequence. The 3-formyl-1-indole acetate **153** was allowed to react with the appropriate amine in the presence of EDC [*N*-ethyl-*N*-(3-dimethylaminopropyl)carbodiimide] and HOBt (hydroxybenzotriazole), affording the corresponding 3-formyl-1-(2-amino-2-oxo-ethyl)-1*H*-indoles **154**. The reaction of the latter with NH_2_OH (EtOH/H_2_O) then NiCl_2_·6H_2_O was added, followed by NaBH_4_ producing the 2-[3-(aminomethyl)-1*H*-indol-1-yl)ethan-1-ones **155,** which were subjected to the reaction with the appropriate phenyl isocyanate (CHCl_3_, TEA, room temperature), affording the targeted **156** (Scheme 27). Some of the synthesized conjugates revealed considerable DDR1/2 and TK-A/-B/-C inhibitory properties (Supplementary Figure S24). The most promising agent observed was that with R^1^ = F, R^2^ = 1-methyl-4-piperazinyl, and R^3^ = 2,4-F_2_, which was subjected to an antiproliferation properties investigation against lung (A549, SPC-A-1, and H1975) cancer cell lines relative to that of dasatinib (IC_50_ = 1.84, 3.51, and 1.87; 2.55, 2.46, and 1.26 μM, respectively). Additionally, the in vivo testing (30 mg/kg dose, mouse model) evidenced its capability for inhibition of bleomycin-induced lung injury [162].

**Figure 18:**
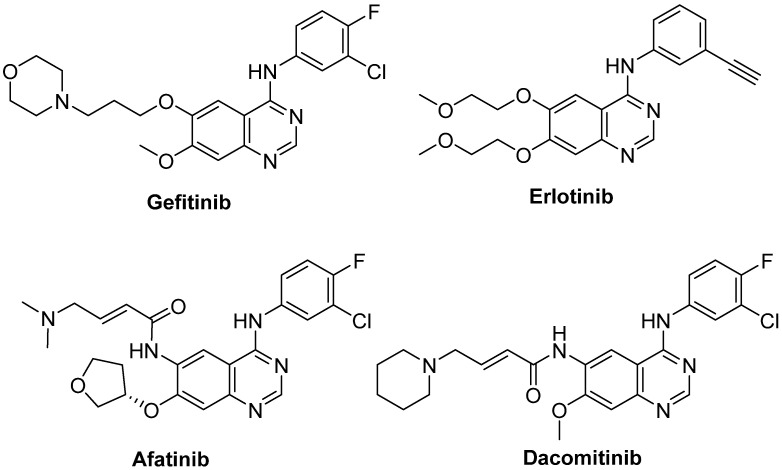
Clinically approved quinazoline-containing compounds with EGFR inhibitory properties.

**Scheme 28:**
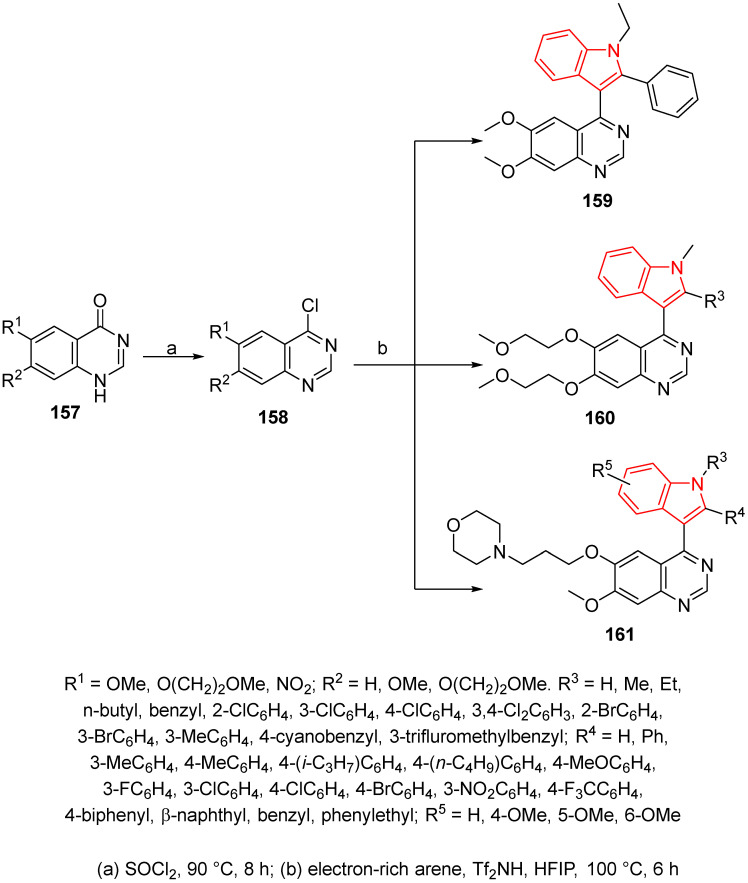
Synthetic route towards quinazoline-indole conjugates **159**–**161**.

EGFR (epidermal growth factor receptor) is an important category of tyrosine kinases, occupying a unique place in cancer chemotherapy. Overexpression of the EGFR is associated with cellular proliferation and many other activities. Many agents have been identified as EGFR inhibitors, and some of them have been chemotherapeutically approved against various cancer types. Several quinazoline-containing compounds were developed with EGFR inhibitory properties and approved against different types of cancers (Figure 18). Conjugation of quinazoline with indole scaffolds was considered for attaining potential EGFR inhibitors. The reaction of 4-chloroquinazolines **158** (obtained through chlorination “thionyl chloride, 90 °C” of the corresponding quinazolinones **157**) with the appropriate indoles using HFIP (hexafluoroisopropanol) and Tf_2_NH [bis(trifluoromethane sulfonimide)] in a sealed tube at 100 °C produced the corresponding conjugates **159–161** (Scheme 28). Enzymatic inhibitory properties of the synthesized conjugates were assayed against the EGFR [L858R] (Supplementary Figure S25). The most promising was **161** with R^3^ = Et, R^4^ = Ph, and R^5^ = H, revealing potent EGFR inhibitory activity [IC_50_ = 5.2, 9.6, and 1.9 nM, against EGFR(WT), EGFR(d746-750), and EGFR(L858R), respectively], antiproliferation properties (IC_50_ = 4.1, 0.5, and 2.1 μM against A549, PC-9, and A431, respectively), arresting the cell cycle at the G0/G1 phases (flow cytometry), and apoptosis induction, in addition to tumor growth suppression evidenced by in vivo testing (BALB/c nude mouse model, oral administration) [163].

**Scheme 29:**
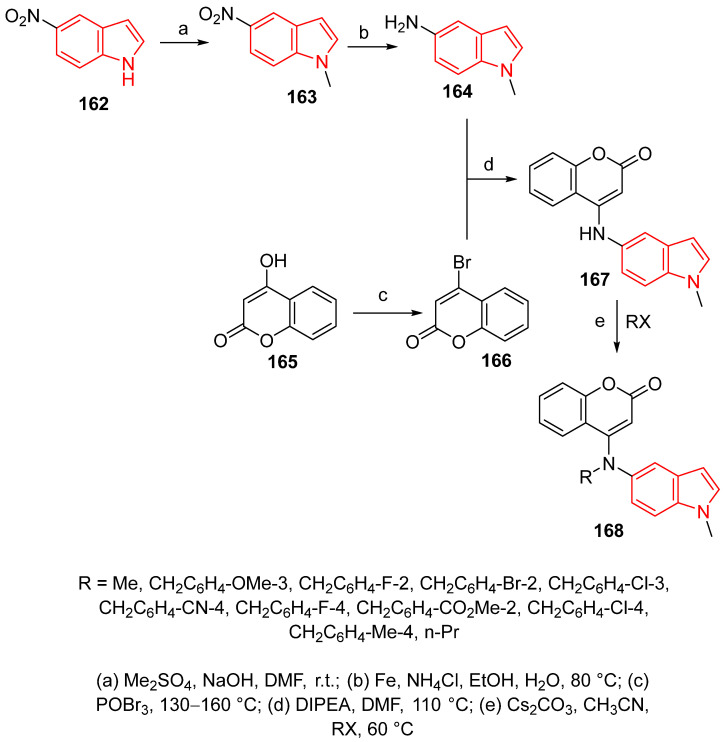
Synthetic route towards coumarin-indole conjugates **168**.

A series of coumarin-indole conjugates **168** was synthesized through dehydrohalogenation (DMF containing DIPEA “*N*,*N*-diisopropylethylamine”, 110 °C) of 5-amino-1-methylindole **164** (obtained from alkylation of 5-nitroindole **162,** followed by a reduction in the nitro group) with 4-bromocoumarin **166** (formed from bromination of coumarin **165**), followed by alkylation (Scheme 29). The antiproliferation properties of **168** were studied against A549, HepG2, and MCF7 cell lines. The most promising agent discovered was that with R = Me against lung cancer cell line A549 (IC_50_ = 1.79 × 10^−3^ μM) relative to that of cisplatin and colchicine (IC_50_ = 5.62 and 0.01 μM, respectively) (Supplementary Figure S26). The most promising agent discovered revealed cell cycle arrest of A549 at the G2/M phase with induction of apoptosis and presumed tubulin polymerization inhibition, as evidenced by molecular docking studies (PDB ID: 1SA0, Autodock Vina software) [164].

### 3.3. Gastric Cancer

**Scheme 30:**
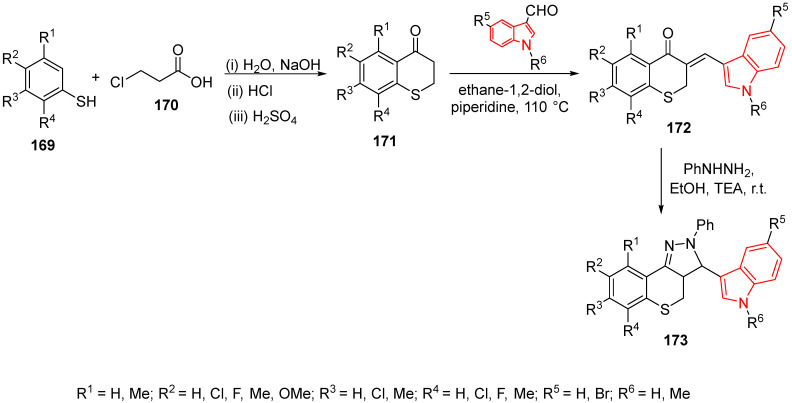
Synthetic route towards thiochromeno[4,3-*c*]pyrazole-indole conjugates **173**.

**Figure 19:**
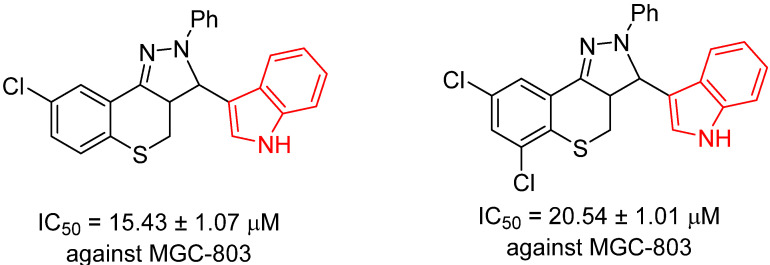
The promising agents of **173** observed against the MGC-803 (gasteric) cancer cell line.

A series of thiochromeno[4,3-*c*]pyrazole-indole conjugates **173** were obtained through Aldol condensation of thiochroman-4-ones **171** with indole-3-carbaldehydes (ethane-1,2-diol, piperidine, 110 °C), followed by cyclocondensation with phenyl hydrazine (EtOH, TEA, room temperature) (Scheme 30). Antripliferation properties (MTT methodology) against MGC-803, Hela, MCF-7, Bel-7404 (gastric, cervical, breast, and liver cancer), and L929 (normal) cell lines were studied (Supplementary Figure S27). Some synthesized hybrids revealed considerable bio-properties relative to etoposide and cisplatin (standard references). The most promising against MGC-803 were those exhibited in Figure 19, which were subjected to a topoisomerase I/II inhibitory assay, revealing selective inhibition against topoisomerase II and no efficacy against topoisomerase I until 100 μM. This behavior was supported by docking studies (PDB ID: 5GWK, Glide XP of Maestro software). They also showed cell cycle arrest (MGC-803 cell) at the G2/M phase [165].

**Scheme 31:**
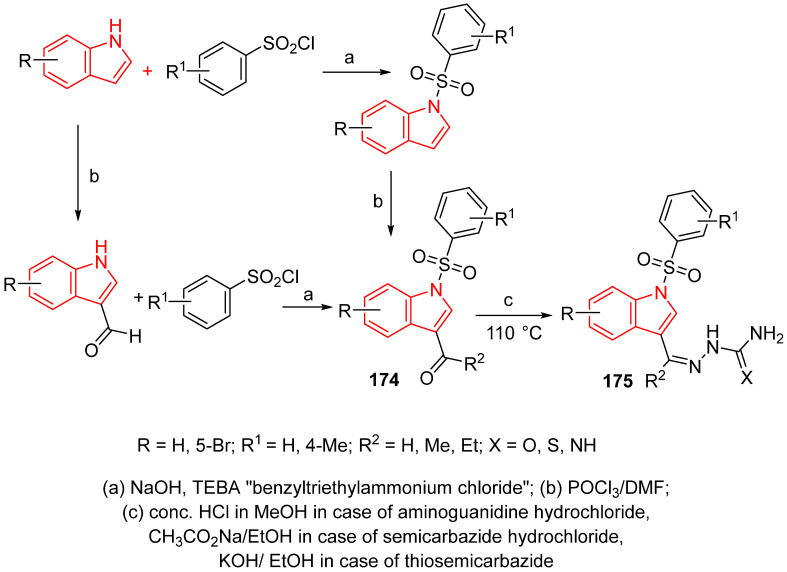
Synthetic route towards *N*-arylsulfonylindoles **175**.

A series of *N*-arylsulfonylindoles **175** was obtained through condensation of the appropriate 3-aldehydic/ketonic indoles **174** with aminoguanidine, semicarbazide, or thiosemicarbazide (Scheme 31). Some of the synthesized indolylhydrazine-1-carboximidamides **175** (X = NH) displayed considerable antiproliferation properties against SGC7901 and A590 (gastric and lung) cancer cell lines (Supplementary Figure S28). The most promising was that with R = 5-Br, R^1^ = 4-Me, and R^2^ = Me, with safe behavior against the normal HEK 293T cell line (IC_50_ = 1.51, 4.44, and 56.39 μM, against SGC7901, A590, and HEK 293T, respectively) [166].

### 3.4. Colorectal Cancer

**Scheme 32:**
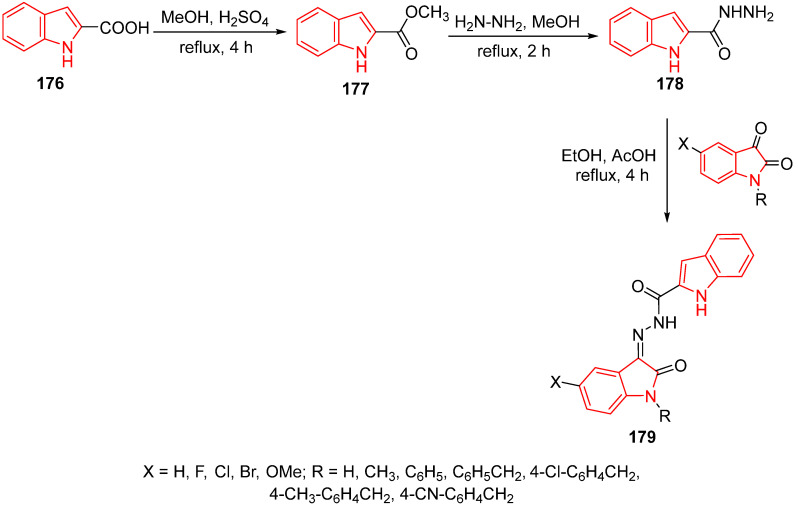
Synthetic route towards isatin–indole conjugates **179**.

A variety of 2-oxo-3-indolylidene-2-indolecarbohydrazones **179** was prepared through condensation (refluxing EtOH containing AcOH in a catalytic amount) of the appropriate isatin with 3-indolecarhydrazides 178 (Scheme 32). The antiproliferative properties of the prepared hydrazones **179** against HT-29, ZR-75, and A-549 (colon, breast, and lung) cancer cell lines were studied (Supplementary Figure S29). The most promising agent is X = Cl and R = CH_2_C_6_H_5_, comparable to sunitinib (IC_50_ = 2.02, 0.74, and 0.76; 10.14, 8.31, and 5.87 μM, respectively). It was also noted that the most promising agent discovered arrested cell cycle at the G1 and G2 phases of the A549 testing cell. Western blot studies revealed the enhancement of BTG1, cdc-2, BAX (B cell translocation gene 1, cyclin-dependent kinase 1, and Bcl-2-associated X protein, respectively), and caspase-3 proteins [167].

**Scheme 33:**
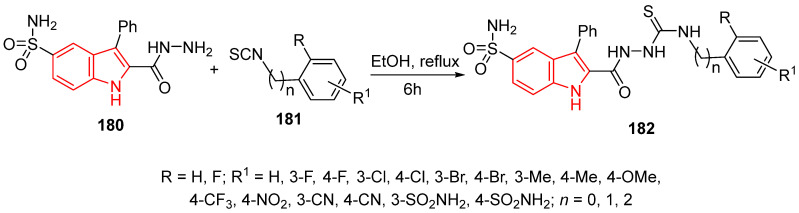
Synthetic route towards 1-(indole-2-carbonyl)thiosemicarbazides **182**.

A series of 1-(indole-2-carbonyl)thiosemicarbazides collaborating with a sulfonamide group **182** was obtained via a reaction of the 2-indolocarbazole **180** with the appropriate isothiocyanate **181** in refluxing ethanol (Scheme 33). A few of the synthesized agents showed mild to considerable antiproliferation properties against HT-29 (colorectal) and skin normal (CCD-86Sk) cell lines (MTT method). The most promising is that with R = H, R^1^ = 4-F, and *n* = 0 (IC_50_ = 53.32 and 74.64 μM, respectively) relative to doxorubicin (IC_50_ = 17.20 and 0.17, respectively). Carbonic anhydrase inhibitory properties against hCA I, hCA II, hCA IX, and hCA XII exhibited the high potency of some of the synthesized agents. The most effective agents are R = H/H, R^1^ = 3-SO_2_NH_2_/4-SO_2_NH_2_, and *n* = 0 (*k*i = 78.7/75.9, 38.0/19.5, 2.1/1.4, and 0.69/0.87 nM, respectively) relative to acetazolamide (reference standard, *k*i = 250.0, 12.5, 25.0, and 5.7, respectively) (Supplementary Figure S30). Molecular docking (Maestro software v2022-3, PDB ID: 3B4F) and molecular dynamic studies were considered to explain the inhibitory behavior against carbonic anhydrases of the promising agents observed [168].

**Scheme 34:**
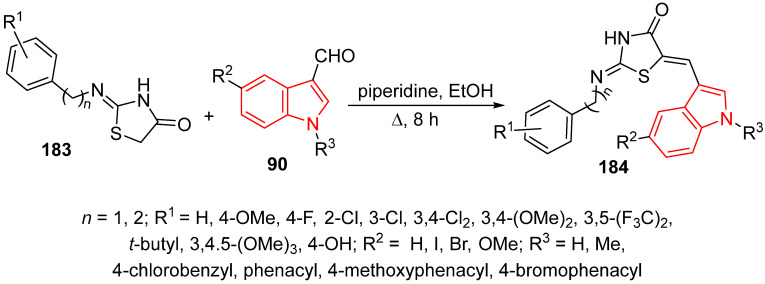
Synthetic route towards thiazolidinone-indole hybrids **184**.

Thiazolidinone-indoles 184 were synthesized through a base-catalyzed condition (refluxing EtOH in the presence of piperidine) of thiazolidinediones **183** with indole-3-carboxaldehyde **90** (Scheme 34). Some synthesized hybrids revealed considerable antiproliferation properties (MTT method, A549, NCI-H460, lung; HCT-29, HCT-15, colon; and MDA-MB-231, breast cancer cell lines). The most promising is that with *n* = 2, R^1^ = 4-OMe, R^2^ = Br, and R^3^ = H relative to podophyllotoxin with safe behavior against normal lung cell L132 (IC_50_ = 0.92 and 0.029; 10.84 and 0.021 μM, against HCT-15 and L132, respectively) (Supplementary Figure S31). Tubulin polymerization inhibition was the molecular target for the most promising agent discovered (IC_50_ = 2.92 μM), with cell cycle arrest at the sub-G1 and G2/M phases. Furthermore, a decrease in mitochondrial membrane potential was observed with an increased intracellular ROS level [169].

**Scheme 35:**
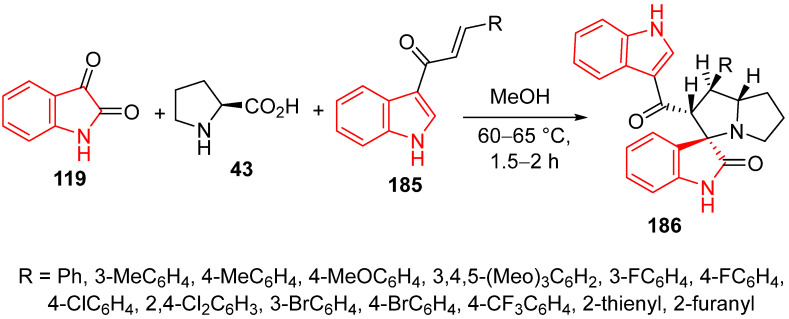
Synthetic route towards spiro[indoline-3,3′-pyrrolizin]-2-ones **186**.

Spiro[indoline-3,3′-pyrrolizin]-2-ones **186** were obtained in diastereoselectivity through a catalyst-free cycloaddition reaction of isatin **119**, *L*-proline **43**, and indolyl-bearing chalcones **185** in boiling MeOH (Scheme 35). Some analogs synthesized displayed promising activity (MTT method) against the HCT116 (colon) cancer cell line, of which R = 3-MeC_6_H_4_, 3-BrC_6_H_4_, 4-CF_3_C_6_H_4_, and 2,4-Cl_2_C_6_H_3_ relative to cisplatin (IC_50_ = 7.0, 9.0, 9.0, 9.0, and 12.5 μM, respectively) (Supplementary Figure S32). Phosphodiesterase 1 (PD-1) inhibitory properties were observed by one of the promising agents observed (R = 2,4-Cl_2_C_6_H_3_), revealing activity at 2 µM with 74.2%, which is explained by molecular docking (PDB ID:1NOP, OpenEye software version 4.1.2) studies [170].

**Figure 20:**
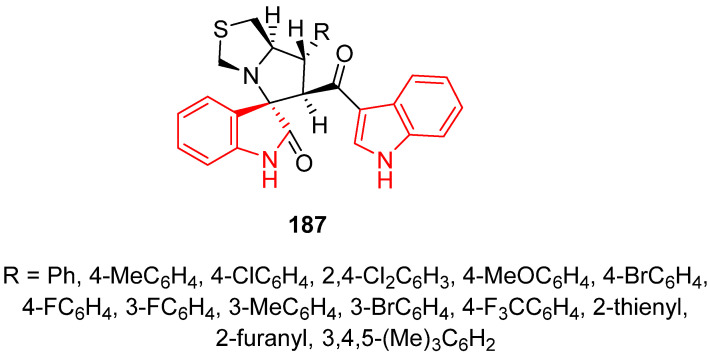
Chemical strucure of the synthesized spiroindoles **187**.

Spiroindoles **187** were similarly obtained upon utilizing *L*-thioproline instead of *L*-proline (Figure 20). A few synthesized compounds showed considerable antiproliferation properties. The most promising is that with R = 4-F_3_CC_6_H_4_ compared to cisplatin (IC_50_ = 7.0, 5.5, and 6.0; 12.6, 5.5, and 5.0 μM against HCT116, HepG2, and PC-3, respectively) (Supplementary Figure S33). Inhibition of the MDM2-P53 interaction was mentioned as the mode of action of the synthesized agents based on theoretical/computational studies (molecular docking, PDB ID: 5law, OpenEye software version 2.2.5) [171].

### 3.5. Pancreatic Cancer

**Figure 21:**
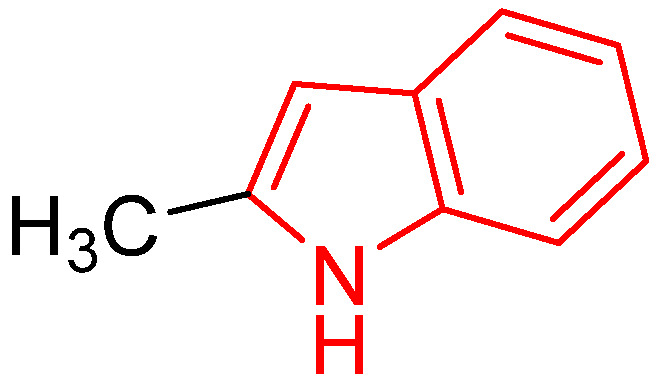
Chemical strucure of 2-methylindole **188**.

Qin et al. reported the efficacy of 2-methylindole **188** against pancreatic cancer, revealing apoptosis and exhibiting antiproliferation properties (Figure 21). Suppression of capan-1, aspc-1, and MIApaCa-2 was mentioned as the apoptotic mode of action. Downregulation of ZFX led to the deactivation of P13K, and AKT phosphorylation was also mentioned [172].

**Scheme 36:**
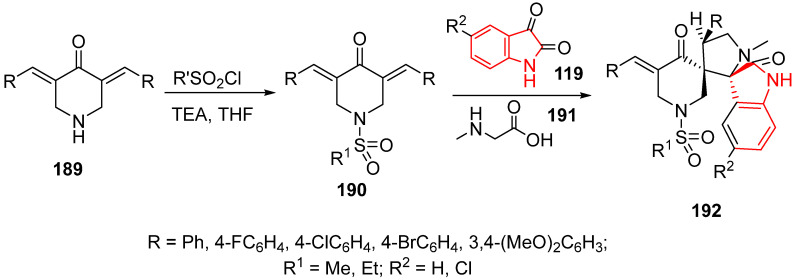
Synthetic routes towards spiroindoles **192**.

Spiroindoles **192** were synthesized through a one-pot reaction of 3,5-diylidene-4-piperidones attached to sulfonyl function **190** with isatins **119** and sarcosine **191** (azomethine cycloaddition) (Scheme 36). The antiproliferation properties (MTT method) of **192** were assessed against PaCa2, MCF7, HCT116, and A431 (pancreatic, breast, colon, and skin) cancer cell lines. Promising properties, relative to the standard drugs (sunitinib and 5-fluorouracil) with inhibitory properties (western blotting study), were observed against VEGFR-2 and the EGFR (Supplementary Figure S34). The most promising against PaCa2 is that with R = 4-BrC_6_H_4_, R^1^ = Me, and R^2^ = H (IC_50_ = 12.500 μM), which is more potent than sunitinib (an FDA-approved drug against pancreatic cancer) (IC_50_ = 16.91 μM). The safety index of **192** was assigned by studying the cytotoxicity against the normal RPE1 cell line [20].

**Scheme 37:**
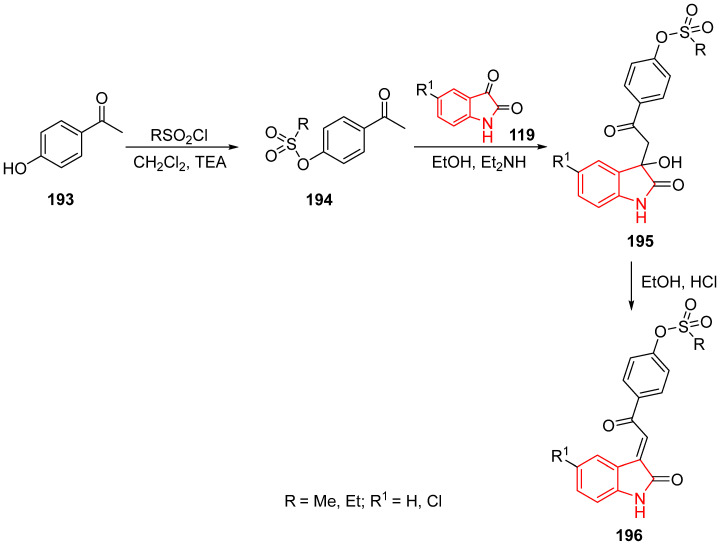
Synthetic route towards 3-alkenyl-2-oxindoles **196**.

**Scheme 38:**
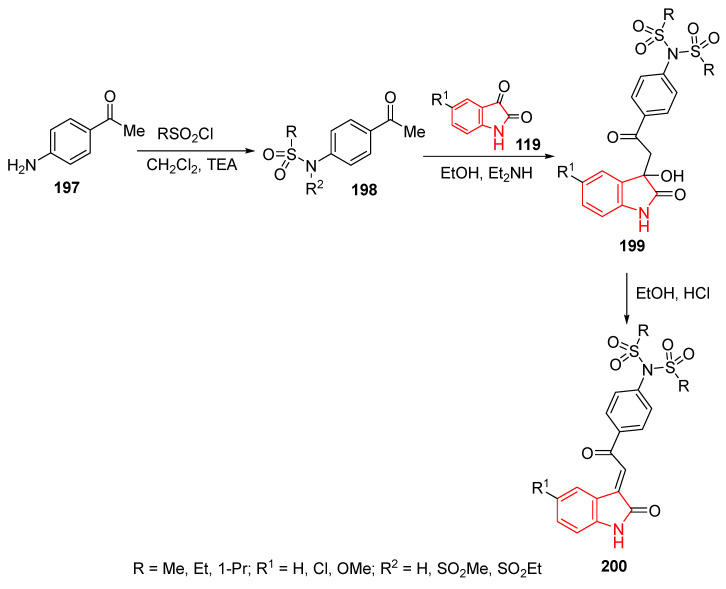
Synthetic route towards 3-alkenyl-2-oxindoles **200**.

The reaction of sulfonated acetophenones **194** with isatins **119** (EtOH/Et_2_NH) produced the corresponding 3-hydroxy-2-oxoindolines **195**. Acid dehydration (EtOH/HCl, room temperature) of **195** afforded the targeted 3-alkenyl-2-oxindoles bearing the sulfonate group **196** (Scheme 37). Applying similar reaction sequences/conditions, 3-alkenyl-2-oxindoles bearing the sulfonamide group **200** were obtained (Scheme 38). 3-Alkenyl-2-oxindoles **196** (R = Et, R^1^ = Cl) and **200** (R = Et, R^1^ = H) are the most promising antiproliferative agents, displaying efficiency against PaCa2 of about 3.4 and 3.3 folds to that of sunitinib (IC_50_ = 4.99, 5.08, and 16.91 µM, respectively). Anti-angiogenic capabilities close to those of sunitinib were supported by CAM (chick chorioallantoic membrane) experiments revealing qualitative and quantitative reductions in blood vessels. Considerable properties were also noticed against MCF7 and HCT116. Inhibitory properties of kinases (VEGFR-2 and c-kit) were noticed by the targeted agents, supporting their mode of action as multi-targeted inhibitors [21] (Supplementary Figure S35).

**Scheme 39:**
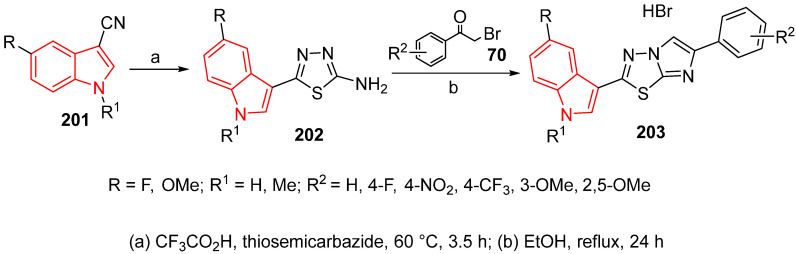
Synthetic route towards indole linked to imidazo[2,1-*b*][1,3,4]thiadiazoles **203**.

Indole linked to imidazo[2,1-*b*][1,3,4]thiadiazoles **203** was obtained through the reaction of indole-3-carbonitriles **201** with thiosemicarbazide in CF_3_CO_2_H at 60 °C, producing the corresponding 2-aminothiadiazols **202**. The reaction of phenacyl bromides **70** with **202** (refluxing EtOH) yielded the targeted agents **203** as hydrobromide salts (Scheme 39). A few of the synthesized **203** exhibited cytotoxic properties against pancreatic cancer cell lines (SUIT-2, Capan-1, and Panc-1; SRB method) (Supplementary Figure S36). A decrease in the tested cell migration in the scratch wound-healing assay was also observed [173].

### 3.6. Liver Cancer

**Scheme 40:**
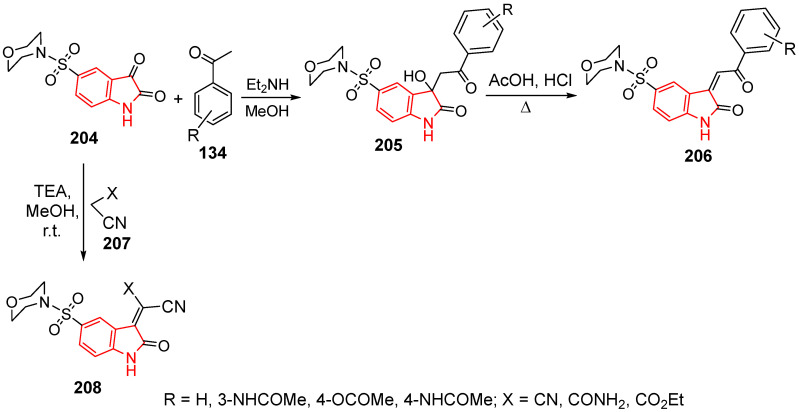
Synthetic route towards 5-(morpholinosulfonyl)-2-indolinones **206** and ylidenes **208**.

The reaction of 5-morphilinosulfonylisatin **204** with the appropriate acetophenone **134** under basic conditions (MeOH, Et_2_NH) followed by acidic dehydration (AcOH, HCl, reflux) produced the corresponding 5-(morpholinosulfonyl)-2-indoline **206** (Scheme 40). Two of the synthesized **206** (R = 3-NHCOCH_3_ and 4-OCOCH_3_) exhibited promising antiproliferation properties against MCF-7, HepG-2, and HCT-116 cell lines (SRB method) relative to doxorubicin. Considerable EGFR inhibitory properties of **206** (R = 3-NHCOCH_3_) were noticed relative to lapatinib (IC_50_ = 0.0191 and 0.0283 µM, respectively). Additionally, condensation of the isatin analog **204** with active methylenes **207** (MeOH, TEA, r.t.) produced the corresponding ylidenes **208**, which also revealed considerable antiproliferation and EGFR inhibitory properties (Supplementary Figure S37). Molecular docking (PDB ID: 1M17, MOE software 10.2008) was considered for explaining the EGFR inhibitory observations [174].

**Scheme 41:**
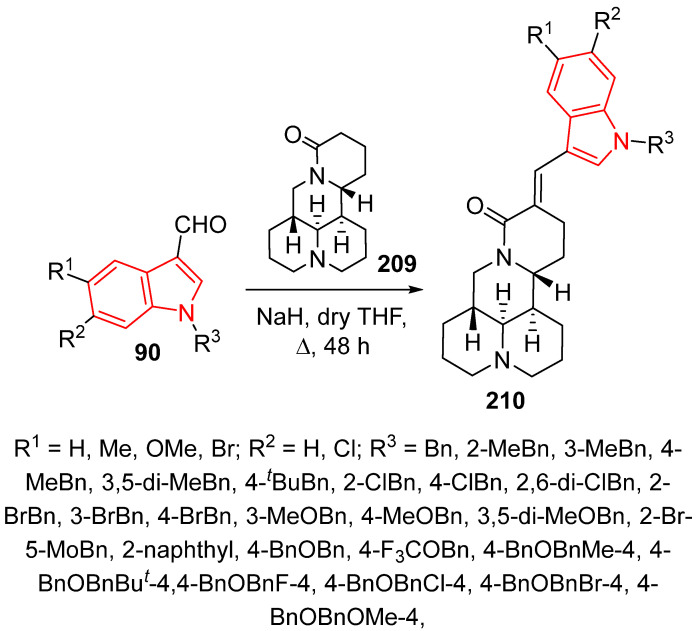
Synthetic route towards sophoridine-indole conjugates **210**.

Sophoridine **209** is a traditional Chinese medication useful for combating a few cancer types (lung, liver, and gastric) in combination with other chemotherapeutics. Sophoridine-indole conjugates **210** were obtained by the Aldol condensation reaction of **209** with the appropriate indole-3-carboxyaldehyde **90** (NaH, dry THF, reflux, 48 h) (Scheme 41). Noticeable antiproliferation properties (MTT method) against HepG2 were observed by **210** relative to sophoridine and camptothecin. The most promising anti-HepG2 agent discovered was R^1^ = OMe, R^2^ = H, and R^3^ = 4-BnOBn (IC_50_ = 1.96, 4670, and 6.08 μM for the potent agents discovered, sophoridine **209** and camptothecin “CPT, natural origin topoisomerase inhibitor”, respectively). Moreover, promising properties were also noticed by this analog against hepatocellular (SMMC-7721), cervical (Hela, CNE1, CNE2), and breast (MCF7) carcinoma cell lines (Supplementary Figure S38). Apoptosis induction of the promising agent discovered was supported by the biochemical observations due to activation of caspase-3, increment/upregulation of the cleaved caspase-3 and Bax, and downregulation/decreasing of Bcl (i.e., reduction in the Bcl-2/Bax ratio). Molecular docking revealed its ability to inhibit topoisomerase I (PDB ID: 1k4t, MOE software version 2008). In vivo (mouse model) studies showed the suppression of the HepG-2 xenograph with no side effects observed [175].

**Scheme 42:**
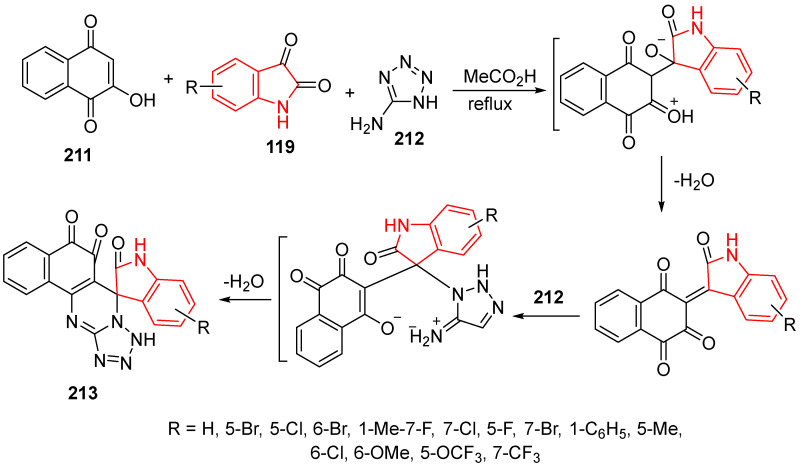
Synthesis of spirooxindoles **213**.

A variety of spirooxindoles **213** was obtained through a reaction of 2-hydroxy-1,4-naphthoquinone **211**, isatins **119**, and 5-amino tetrazole **212** in refluxing acetic acid (Scheme 42). Some of the synthesized analogs displayed noticeable antiproliferative properties (MTT methodology) against HepG-2 and safe behavior against normal LO2 cancer cell lines (Supplementary Figure S39). The most promising agents are those with R = 5-F, 7-Cl, and 7-CF_3_ (IC_50_ = 2.86, 3.03, and 7.9 μM, respectively) relative to the positive control tanshinon IIA (TSA “natural cytotoxic agent isolated from *Salvia miltiorrhiza*”, IC_50_ = 23.85 μM) [176].

### 3.7. Prostate Cancer

**Scheme 43:**
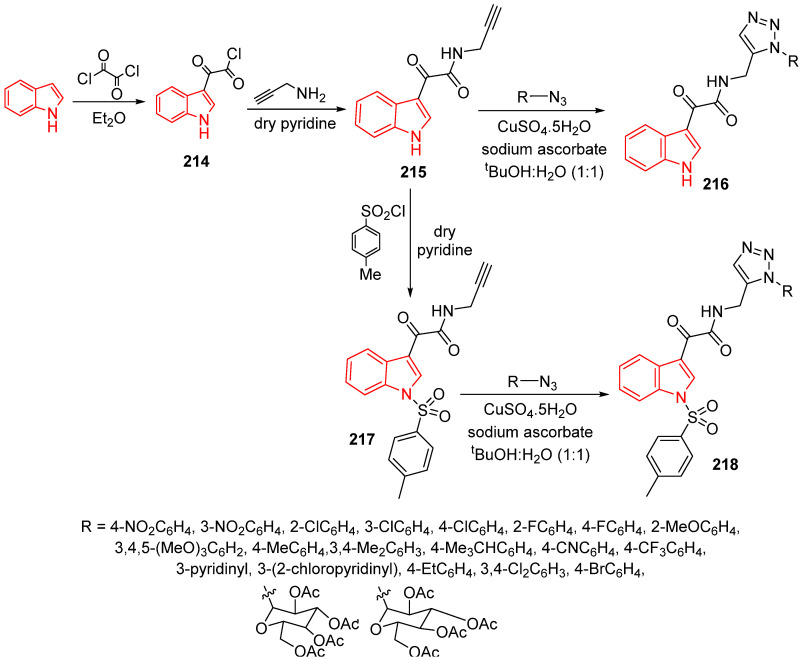
Synthetic route towards 1,2,3-triazole-indole-3-glyoxamides **216** and **218**.

In an attempt to determine the role of COX (cyclooxygenase) and 5-LOX (5-lipoxygenase) as hypothesized biochemical pathways potentially correlated in cancer inhibition/antiproliferation, a set of 1,2,3-triazole-indole-3-glyoxamides **216** and **218** was designed and explored for their potential properties against the targeted anti-inflammatory and antitumor enzymes. The reaction of indole-3-glyoxalyl chloride **214** with propargyl amine produced the corresponding propargylated agent **215**. The click reaction of **215** with azide analogs (in tert-BuOH—H_2_O “1:1 *v*/*v*” using CuSO_4_.5H_2_O, sodium ascorbate) yielded the corresponding indole-triazole conjugates **216**. Similarly, the indole-triazole conjugates bearing the sulfonyl group **218** were also synthesized (Scheme 43). The antiproliferation properties of the synthesized agents were assessed against SKOV3, DU145, and HELA (ovarian, prostate, and cervical, respectively) cell lines (MTT assay). A few of the synthesized agents **216** (R = 4-C_2_H_5_C_6_H_5_ and R = 4-FC_6_H_5_) showed promising antiproliferation properties relative to etoposide (VP16) against the DU145 cell line (IC_50_ = 8.17, 8.69, and 9.80 µM, respectively). Tubulin polymerization inhibition was evidenced for the promising agent discovered **216** (R = 4-C_2_H_5_C_6_H_5_). Promising COX-2 and 5-LOS inhibitory properties were revealed for the synthesized agent discovered **216** (R = 4-C_2_H_5_C_6_H_5_, IC_50_ = 0.12 and 7.73, respectively), relative to the anti-inflammatory drugs indomethacin and celecoxib (IC_50_ against COX-2 = 0.049 and 0.041 μM, respectively), and norhihydroguaiaretic acid (NDGA, IC_50_ against 5-LOX = 7.31 μM) (Supplementary Figures S40 and S41). Molecular docking studies (PDB ID: 4RRX, 3V99, Maestro version 9.6 implemented from Schrodinger software suite) evidenced the observations against COX and 5-LOX bio-properties. Additionally, in silico studies (PDB ID: 4O2B) supported the ability of the promising agent(s) discovered for mapping in the colchicine binding site. Anti-inflammatory properties were supported for the promising agents discovered through in-vivo testing in rats (carrageenan paw edema method) with no gastric ulceration [177].

### 3.8. Cervical Cancer

**Scheme 44:**
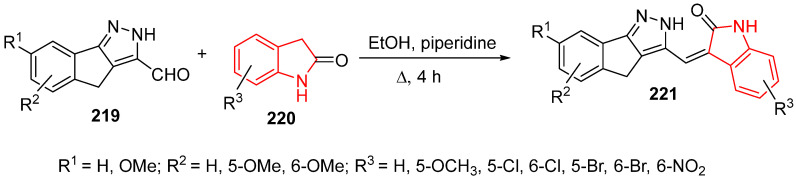
Synthetic route towards 3-[(indeno[1,2-*c*]pyrazole-3-yl)methylene]indolin-2-ones **221**.

A set of 3-[(indeno[1,2-*c*]pyrazole-3-yl)methylene]indolin-2-ones **221** was assessed as tubulin polymerization inhibitors. The targeted agents **221** were synthesized through Knoevenagel condensation of indolin-2-ones **220** with indeno[1,2-*c*]pyrazole-3-carbaldehydes **219** in refluxing EtOH using piperidine as a basic catalyst (Scheme 44). The antiproliferation properties (SRB assay) of the targeted compounds against HeLa, A549, and MDA-MB-231 (cervical, lung, and breast) cancer cell lines and compared to non-cancer HEK-293 cell lines were studied relative to combretastatin A-4 (CA-4) (Supplementary Figure S42). Amongst all, analog **221** with R^1^ = OMe, R^2^ = 5-OCH_3_, and R^3^ = 6-Cl exhibited promising properties relative to CA-4 (IC_50_ = 1.33 and 1.43 μM, respectively). It also increased the checkpoint protein levels (cyclin B1 and CDK1), exhibiting cell cycle arrest in HeLa at the G2/M phase (leading to apoptosis, flow cytometry). Upregulation of tumor suppressor proteins (p53, p21, and pro-apoptotic Bax) was also observed. Tubulin polymerization inhibition was evidenced via the occupation of the colchicine binding pocket in molecular docking studies (PDB ID: 1SA0, Autodock 4 software) [178].

**Scheme 45:**
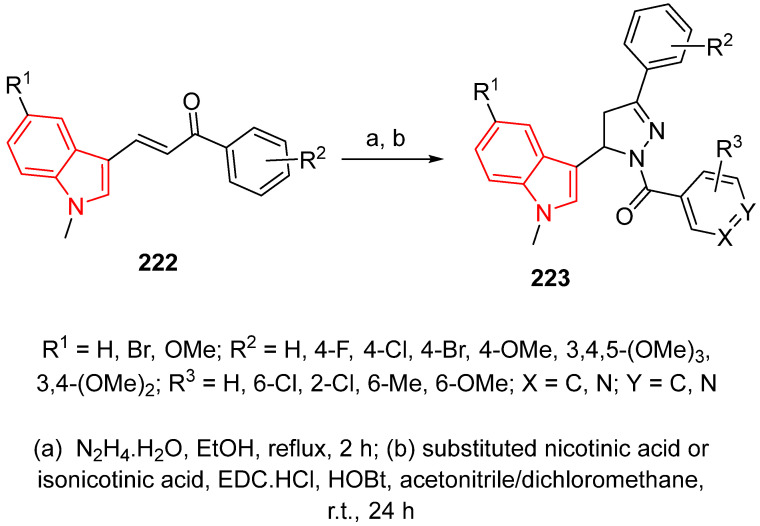
Synthetic route towards nicotinoyl pyrazolines bearing *N*-methyl indolyl heterocycle **223**.

Sets of nicotinoyl/isonicotinyl pyrazolines featuring indolyl heterocycle **223** were designed as tubulin polymerization inhibitors. The targeted compounds were obtained through the reaction of indolyl chalcones **222** with hydrazine hydrate in refluxing EtOH. Then, the pyrazolinyl intermediates were allowed to react with nicotinic or isonicotinic acid in an inert atmosphere immediately, without any purification (Scheme 45). The antiproliferative properties of the targeted agents were assessed against four cancer cell lines (MTT technique). Promising antiproliferative properties were noticed by some of the synthesized agents. The most promising is that with R^1^ = OMe, R^2^ = 3-OMe, R^3^ = 6-Me, X = N, and Y = C against the tested cell lines MCF-7, A549, HepG2, and HeLa relative to CA-4 (GI_50_ = 0.09, 0.59, 0.029, and 0.034; 0.14, 0.31, 017, and 0.092 μM, respectively) with safe observations against the non-cancer 293T cell line (CC_50_ = > 300 μM for both). Remarkable tubulin polymerization inhibition was noticed by the promising agent discovered relative to that of CA-4 (IC_50_ = 1.6 and 2.1 μM, respectively) (Supplementary Figure S43). In vivo testing (HeLa-xenograft mouse model) of the promising agent revealed evidence of better tumor inhibition without weight loss or tissue damage relative to the standard CA-4 (% inhibition = 61.52 and 59.92, respectively). Molecular docking (PDB ID: 1SA0, Discovery Studio 3.5 software) and molecular dynamics (Desmond, Schrödinger software) supported the mode of action mentioned [179].

**Scheme 46:**
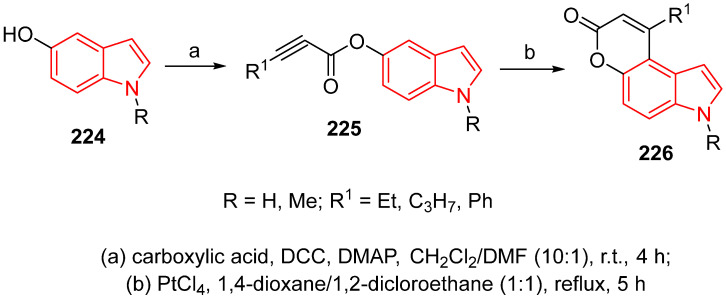
Synthetic route towards indoles **225** and pyranoindoles **226**.

A set of indoles **225** and pyranoindole **226** has been explored as anticancer agents with tubulin polymerization inhibitory properties. Esterification of 5-hydroxyindoles **224** with the appropriate carboxylic acid (pent-2-ynoic acid, es-2-ynoic acid, or phenylpropiolic acid) afforded the corresponding esters **225**. The intramolecular cyclization reaction of **225** under reflux in the presence of PtCl_4_ as a catalyst produced the corresponding pyranoindoles **226** (Scheme 46). Some of the synthesized agents showed considerable antiproliferation properties (MTT method), of which **225** with R = H and R^1^ = Ph relative to vinblastine against the HeLa cell line (IC_50_ = 3.6 and 6.7 × 10^−2^ μM, respectively) showed tubulin polymerization inhibition (Supplementary Figure S44). In silico/docking studies (PDB ID: 5J2T, Autodock v 4.2.2. software) explained the mode of action [180].

### 3.9. Ovarian Cancer

**Scheme 47:**
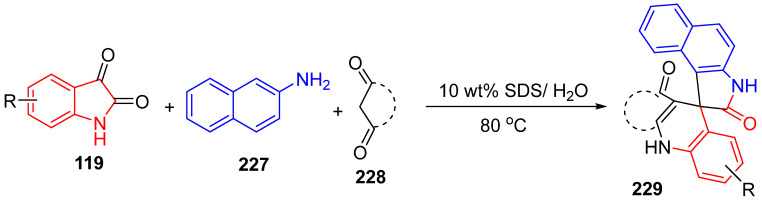
Synthesitic route towards spiroindoles **229**.

**Figure 22:**
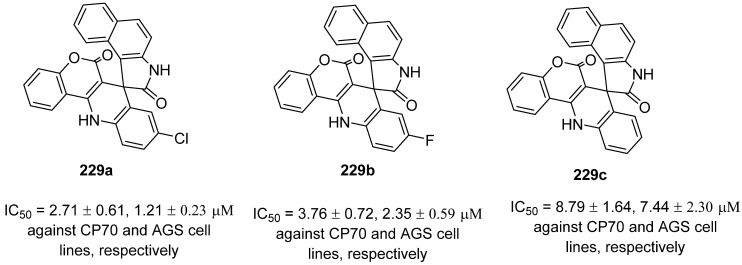
Antiproliferation properties of the promising spiroindoles **229a**–**c** against ovarian cancer cell lines.

A set of 1*H*-benzo[*e*]indole-2(3*H*)-one spirocyclic derivatives **229** was designed as pyroptosis inducers and synthesized through greenish technique in a one-pot reaction of isatins **119**, 2-naphthylamine **227**, and 1,3-dicarbonyl compounds (including barbituric acid, 1,3-dimethylbarbituric acid, thiobarbituric acid, 1,3-cyclohexanone, 5,5-dimethyl-1,3-cyclohexanone, and 2,4-dimethylbenzopyranone) **228** utilizing free-catalyst conditions and using water as a solvent containing SDS (sodium dodecyl sulfate and cationic surfactant, 10 w%) at 80 °C. X-ray studies have evidenced the structure of **229** (Scheme 47). Antiproliferative properties (MTT assay) were determined against ovarian cancer cell lines (CP70 and AGS). Some of the synthesized agents (Figure 22) revealed considerable antiproliferation properties against the tested cell lines relative to the standard references (5-fluorouracil and oxaliplatin, IC_50_ = 55.90 ± 0.08 and 4.01 ± 0.67; 35.81 ± 0.77 and 1.76 ± 0.68 μM against CP70 and AGS cell lines, respectively). The most promising agent, **229a**, was subjected to further pharmacological studies, observing its ability to hinder the formation of colonies, migration, and invasion of ovarian carcinoma cells. Upregulation of the expression of GSDME-N (pyroptosis-related proteins) in ovarian cancer cells tested (CP70 and A2780) was also evidenced by Western blotting studies. A reduction in ovarian cancer volume and weight was noticed through in vivo studies (mouse xenograft model) [181].

### 3.10. Leukemia

**Scheme 48:**
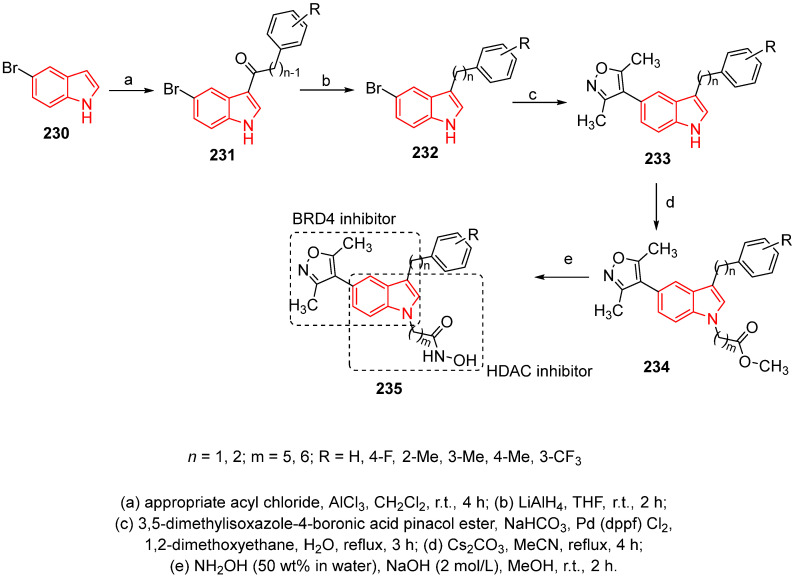
Synthetic route towards indole-isoxazole conjugates **235**.

A set of indole-isoxazole conjugates as histone deacetylases (HDACs)/BRD4 (bromodomain-containing protein) dual inhibitors was designed and synthesized as promising anticancer agents. The targeted conjugates **235** were obtained through the acylation reaction of 5-bromoindole **230,** giving the intermediates **231**, which, via the hydrogenation reaction (LiAlH_4_, THF), afforded the corresponding indolyl derivatives **232**. Coupling **232** with 3,5-dimethylisoxazole-4-boronic acid and pinacol ester, followed by alkylation, produced **234**. Ammonolysis of **234** (NH_2_OH, NaOH, and MeOH/H_2_O) afforded the targeted hydroxamic conjugates **235** (Scheme 48). Moderate antiproliferation properties of the targeted conjugates **235** against the THP-1 (leukemia) cell line with promising inhibition of HDAC and BRD4 were exhibited (Supplementary Figure S45). The most promising agent **235** discovered is that with *n* = 1, m = 6, and R = 4-F (IC_50_ = 5 nM against HDAC3 and the % inhibition of BRD4 = 88% at 10 μM). The downregulation of the c-Myc protein and the upregulation of acetylated histone H3 (Ac-H3) are in accordance with the tumor growth inhibitory effect [182].

**Scheme 49:**
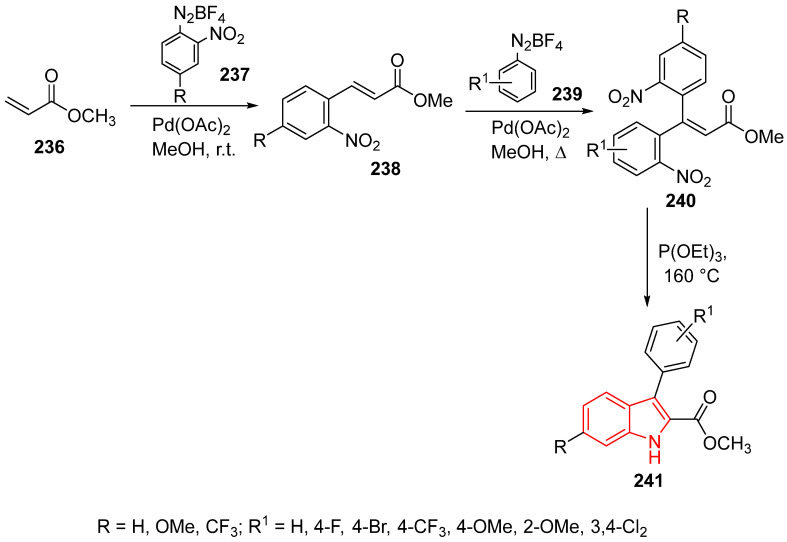
Synthetic route towards 2-carbomethoxy-3-arylindoles **241**.

3,6-Disubstituted-2-carboxyindoles **241** were reported as anti-leukemic agents. The targeted agents **241** were synthesized through Heck-Matsuda arylation of methyl acrylate **236** with arenediazonium salts **237** in the presence of palladium acetate as a catalyst, producing cinnamates **238**. The Heck-Matsuda reaction with a 2 mol equivalent of arenediazonium salt 239 under the same catalytic reaction conditions afforded β,β-diarylacrylates 240. Cadogan-Sundberg reductive cyclization of **240**, promoted by P(OEt)_3_, furnished the final targets 241 (Scheme 49). The cytotoxic properties of **241** (MTT assay) against CEM, RS4, and 11 (leukemia) cancer cell lines were studied (Supplementary Figure S46). Indolyl analog **241** with R = OMe and R^1^ = CF_3_ displayed the most promising properties (IC_50_ = 0.20 and 0.30 μM, respectively), with tubulin polymerization inhibition targeting/arresting the G2/M phase in addition to DNA damage and apoptosis induction. In vivo studies (xenograft mouse, i.p. 10 mg/kg × 5 per week) evidenced overall animal survival [183].

## 4. Conclusions

In conclusion, the indole scaffold has emerged as a promising foundation for developing potential anticancer agents, providing numerous opportunities for future research and therapeutic applications. Indole derivatives exhibit diverse chemical structures and versatile pharmacological activities, making them attractive drug discovery and development candidates. The indole scaffold possesses several inherent properties, contributing to its potential as an anticancer agent. It demonstrates favorable drug-like characteristics such as good oral bioavailability, metabolic stability, and cell permeability. Furthermore, indole derivatives have displayed various mechanisms of action, including inhibition of cell proliferation, induction of apoptosis, and interference with key signaling pathways involved in cancer development and progression. A significant advantage of the indole scaffold is its structural flexibility, which allows for extensive modifications and optimization of drug-like properties. Researchers can explore different synthetic strategies to introduce functional groups, alter substitution patterns, and fine-tune the physicochemical properties of indole-based compounds. This enables the design of highly potent and selective anticancer agents with improved efficacy and reduced toxicity. Additionally, the indole scaffold shows promise in targeting specific molecular targets crucial for cancer cell survival and proliferation, such as enzymes like kinases, histone deacetylases, and topoisomerases. Indole-based compounds have demonstrated potent anticancer activity in preclinical studies by selectively inhibiting these targets. Furthermore, aside from their direct anticancer effects, indole derivatives have the potential to modulate multidrug resistance in cancer cells, a common challenge in cancer treatment, by inhibiting efflux pumps and enhancing the intracellular accumulation of chemotherapeutic agents, thus overcoming resistance and sensitizing cancer cells to treatment.

Indole-based compounds are poised to play a significant role in anticancer research in the future. Continual advancements in synthetic chemistry, computational modeling, and high-throughput screening techniques are expected to uncover new indole derivatives with improved potency, selectivity, and pharmacokinetic properties. Moreover, breakthroughs in personalized medicine and identifying specific biomarkers linked to the response to indole-based compounds will facilitate targeted therapy and enhance patient outcomes.

## Figures and Tables

**Table 1:** Indole-containing drugs against different diseases.

Drug	Bio-Properties	Reference
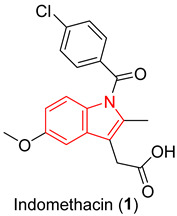	Non-steroidal anti-inflammatory drug, “NSAID”	[30]
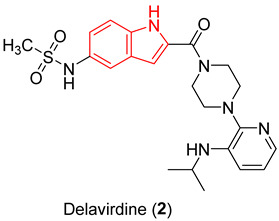	Anti-HIV, “human immunodeficiency virus”	[31]
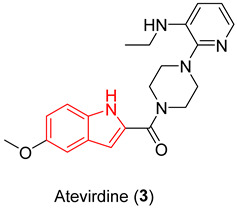	Anti-HIV	[32]
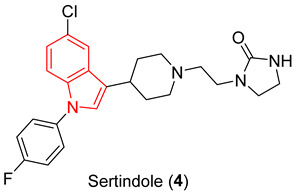	Neuroleptic	[33]
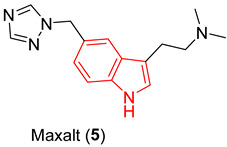	Analgesic	[34]
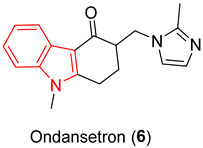	Antiemetic	[35]
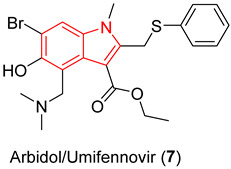	Antiviral	[36]
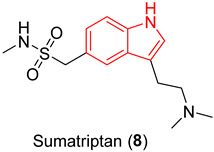	For treatment of migraines and cluster headaches	[37]
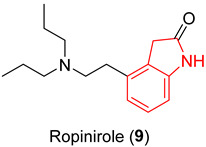	For treatment of symptoms of Parkinson’s disease and restless legs syndrome	[38]
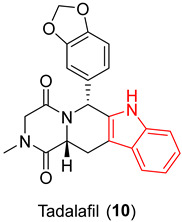	For treatment of erectile dysfunction, benign prostatic hyperplasia, and pulmonary arterial hypertension	[39]
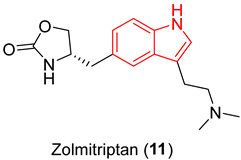	For treatment of acute migraine with or without aura in adults	[40]
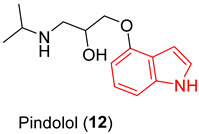	Antihypertensive (β-antagonist)	[41]

## Supplementary Materials

The following supporting information can be downloaded at: https://www.mdpi.com/article/10.3390/ph17070922/s1, Figure S1–S46: Exhibited the chemical structure, biological properties and mode of action of the mentioned compounds.

## Abbreviations

5-LOX	5-Lipoxygenase
AAZ	Acetazolamide
ACD	Accidental cell death
BAX	Bcl-2-associated X protein
Bcl-2	B-cell lymphoma 2
BRD	Bromodomain-containing protein
BTG1	B cell translocation gene 1
CA-4	Combretastatin A-4
cdc-2	Cyclin-dependent kinase 1
Cell-CuI NPs	Cellulose-supported CuI nanoparticles
COX	Cyclooxygenase
CPT	Camptothecin
DDR	Discoidin domain receptors
DIPEA	*N*,*N*-Diisopropylethylamine
DME	Dimethoxyethane)
EDC	*N*-Ethyl-*N*-(3-dimethylaminopropyl)carbodiimide
EGF	Epidermal growth factor
ER-α	Estrogen receptor-α
FGFR	Fibroblast growth factor receptor
hCA	Human carbonic anhydrases
HDAC	Histone deacetylase
HDACs	Histone deacetylases
HFIP	Hexafluoroisopropanol
HFIP	Hexafluoroisopropanol
HIV	Human immunodeficiency virus
HOBt	Hydroxybenzotriazole
HPV	Human papillomavirus
IBX	Iodoxybenzoic acid
MTT	3-(4,5-Dimethylthiazol-2-yl)-2,5-diphenyl-tetrazolium bromide
NMPA	National Medical Products Administration
NSAID	Non-steroidal anti-inflammatory drug
NSCLC	Non-small cell lung cancer
PARP-1	Poly(ADP-ribose) polymerase-1
PD-1	Phosphodiesterase 1
PDGFR	Platelet-derived growth factor receptor
p-Erk	Phosphorylated extracellular signal-regulate kinase
RCD	Regulated cell death
ROS	Reactive oxygen species
ROS	Reactive oxygen species
SARS-CoV-2	Severe acute respiratory syndrome coronavirus-2
SDS	Sodium dodecyl sulfate
SRB	Sulforhodamine B
TEBA	Benzyltriethylammonium chloride
Tf_2_NH	Bis(trifluoromethane sulfonimide)
TK	tyrosine kinase
VEGFR	Vascular endothelial growth factor receptor
