# Supplementary material for: Indole Compounds in Oncology: Therapeutic Potential and Mechanistic Insights
Source: Pharmaceuticals (Basel). 2024 Jul 10;17(7):922. doi: 10.3390/ph17070922 (PMC11280311; doi:10.3390/ph17070922)
Supplement: Supplementary file 1 [file pharmaceuticals-17-00922-s001.zip › pharmaceuticals-3088241-supplementary.pdf]

Indole Compounds in Oncology: Therapeutic Potential and Mechanistic Insights

Sara M. Hassan[1], Alyaa Farid[1], Siva S. Panda[2,3,*], Mohamed S. Bekheit[4], Holden Dinkins[2], Walid Fayad[5] and Adel S. Girgis[4,*]

[1] *Biotechnology Department, Faculty of Science, Cairo University, Giza 12613, Egypt*

[2] *Department of Chemistry and Biochemistry, Augusta University, Augusta, GA 30912, USA*

[3] *Department of Biochemistry and Molecular Biology, Augusta University, Augusta, GA 30912, USA*

[4] *Department of Pesticide Chemistry, National Research Centre, Dokki, Giza 12622, Egypt; m_bekheit@yahoo.com*

[5] *Drug Bioassay-Cell Culture Laboratory, Pharmacognosy Department, National Research Centre, Dokki, Giza, 12622. Egypt*

* Correspondence: sipanda@augusta.edu or sspanda12@gmail.com (S.S. Panda); girgisas10@yahoo.com or as.girgis@nrc.sci.eg (A.S.G.)

Supplementary material

Figure captions

Fig. S1. Antiproliferation properties (μM ± SD) and carbonic anhydrase inhibitory activity (μM) of the prepared chalcones **50** and standard references (Doxorubicin and AZM)……………………………………………………….. 6

Fig. S2. Antiproliferation properties (μM ± SD) of the synthesized agents **54**, **55**, Camptothecin and Harmine…………………………………………………….. 8

Fig. S3. Antiproliferation properties (μM) and binding affinity with ER-a (nM) of the synthesized indole-benzimidazole conjugates **58**……………………………. 12

Fig. S4. Antiproliferation properties of the synthesized indolyl sulfonohydrazones **66** and Doxorubicin………………………………………….. 14

Fig. S5. Antiproliferation and tubulin polymerization inhibitory properties (μM ± SD) of the synthesized thiazolyl hydrazones linked to indolyl scaffold **71**, Colchicine and CA-4 (control drugs)……………………………………………... 16
Fig. S6. Antiproliferation (μM ± SD) and % inhibitory properties of tubulin polymerization at 10 μM of the synthesized 3-amidoindoles **80** and CA-4 (control drug)……………………………………………………………………….. 21
Fig. S7. Antiproliferation and tubulin polymerization inhibitory properties (μM ± SD) of the synthesized 3-arylthio-1*H*-indoles **83**, 3-aroyl-1*H*-indoles **85** and standard drugs (CA-4 and Colchicine)……………………………………………. 26
Fig. S8. Antiprolifeation (nM ± SD) and tubulin polymerization inhibitory (μM ± SD) properties of 6-ary-3-aroyllindoles **89** and CA-4 (standard reference)……… 29
Fig. S9. Antiproliferation properties (μM ± SD) of the synthesized indolyl-arylaminopropenone conjugates 93 and reference drug (Doxorubicin)…………... 31
Fig. S10. Antiproliferation properties (μM ± SD) of the synthesized 3-pyrrolylisatin-triazole conjugates **104** and Tamoxifen citrate "reference drug" (ND = not determined)………………………………………………………. 34
Fig. S11. Antiproliferation properties (μM ± SD) of spirochromenocarbazols linked to 1,2,3-triazole **106**, and standard references (Paclitaxel and Doxorubicin)……………………………………………………………………. 37
Fig. S12. Antiproliferation properties (μM) of tetrahydro-b-carboline and isatin scaffolds connected by 1*H*-1,2,3-triazolyl heterocycle **109** and reference standards (Plumbagin, Peganumine A and Tamoxifen)…………………………. 41
Fig. S13. Antiproliferation properties (μM) of the synthesized ospemifene-isatins **116** and ospemifene-spiroisatins **117** conjugates and reference standards (Ospemifene, Tamoxifen and Plumbagin) agains MCF-7 and MDA-MB-231…. 45
Fig. S14. Antiproliferation properties of spiroxindoles bearing 2-furanyl heterocycle **121** and Staurosporine……………………………………………….. 49
Fig. S15. Antiproliferation properties (μM) against MDA-MB-231 and inhibitory properties (μM ± SD) agaist EGFR of spiroxindoles **124** and standard references (Doxorubicin (Adriamycin) and Erlotinib………………………………………… 51
Fig. S16. Antiproliferation properties (μM ± SD) and EF-α inhibitory properties

(nM ± SD) of the synthesized *N*-(1*H*-indole-6-yl) benzamides **127** and their benzene sulfonamide analogs **128**……………………………………………… 53
Fig. S17. Antiproliferation properties (μM) of [1,3]thiazino[3,2-*a*]indol-4-ones **131**………………………………………………………………………………. 54
Fig. S18. Antiproliferation properties (μM ± SD) of the synthesized indole-podophyllotoxin conjugates **133**, and standards (Colchicine, Podophyllotoxin and Nocodazole) against HepG-2, HL-7702 (liver), HeLa, H8 (cervical), A549, MRC-5 (lung), MCF-7, and HMEC (breast) cancer cell lines…………………… 55
Fig. S19. Antiproliferation properties (μM ± SD) of the synthesized indolylthiosemicarbazones **139** and standards (Etoposide and Colchicine)…….. 57
Fig. S20. Antiproliferation properties (μM) and % inhibition of microtubule polymerization at 10 μM of the respective ligand of the synthesized bis(indolyl)hydrazide-hydrazones **142** and Colchicine………………………… 60
Fig. S21. Antiproliferation properties of indole-based tambjamine analogs **144** and Cisplatin………………………………………………………………………. 61
Fig. S22. Antiproliferation properties (μM ± SD) of indirubin **145**, indirubin-piperidine conjugates **146-148** and Bortezomib………………………………. 62
Fig. S23. Antiproliferation properties (μM) of the synthesized piperlongumine pharmacophore conjugated with indolyl scaffold **152**. and its precursor piperlongumine **149**………………………………………………………………. 63
Fig. S24. Kinase inhibitory (% control at 1.0 μM) and antiproliferation (μM ± SD) properties of the synthesized indole-containing compounds linked to urea function **156** and Dasatinib (reference standard)……………………………… 64
Fig. S25. enzymatic inhibitory properties of synthesized agents **160**, **161** and Gefitinib against EGFR[L858R]……………………………………………….. 69
Fig. S26. Antiproliferation properties (μM ± SD) of the synthesized coumarin-indole conjugates **168**, Cisplatin and Colchicine……………………………… 73
Fig. S27. Antiroliferation properties (μM ± SD) of thiochromeno[4,3-*c*]pyrazole-indole conjugates **173**, Etoposide and Cisplatin (standard references)………… 75
Fig. S28. Antiproliferation properties of the synthesized indolylhydrazine-1-carboximidamide **175**………………………………………………………… 77

Fig. S29. Antiproliferation properties of the synthesized 2-oxo-3-indolylidene-2-indolecarbohydrazones **179** and Sunitinib……………………………………….. 78
Fig. S30. Antiproliferation and carbonic anhydrase inhibitory properties of 1-(indole-2-carbonyl)thiosemicarbazides incolaborating sulfonamide group **182** and standard references (Doxorubicin and Acetazolamide "AAZ")………………….. 81
Fig. S31. Antiproliferation properties of thiazolidinone-indoles **184** and Podophyllotoxin………………………………………………………………... 84
Fig. S32. Antiproliferation properties of spiro[indoline-3,3'-pyrrolizin]-2-ones **186** and cisplatin against HCT-116, HepG2, PC-3 (cancer) and VERO-B (normal) cell lines……………………………………………………………… 89
Fig. S33. Antiproliferation properties of spiroindoles **187** and Cisplatin agains HCT-116, HepG2, PC-3 (cancer) and VERO-B (normal) cell lines…………….. 91
Fig. S34. Antiproliferation (IC_{50}, μM ± SEM against the tested cell lines), and % inhibitory properties against EGFR and VEGFR-2 ustilizing IC50 observed against MCF7 for the tested spiroindoles **192** and standard drugs (5-Fluorouracil and Sunitinib)………………………………………………………………… 93
Fig. S35. Antiproliferation (IC_{50}, μM ± SE "standard error") and enzymatic inhibitory properties (IC_{50}, μM ± SD) of 3-alkenyl-2-oxindoles **196**, **200** and Sunitinib………………………………………………………………………… 96
Fig. S36. Antiproliferation properties (μM ± SEM) of indole linked to imidazo[2,1-*b*][1,3,4]thiadiazoles **203**……………………………………………. 98
Fig. S37. Antiproliferation (μM ± SE) and enzymatic inhibitory properties against EGFR of 5-(morpholinosulfonyl)-2-indolinones **206**, 3-ylidene-2-indolinones **208** and standard references (Doxorubicin and Lapatinib)………… 99
Fig. S38. Antiproliferation properties (μM ± SD) of the synthesized sophoridine-indole conjugates **210**, Sophoridine **209** and Camptothecin (standard reference).... 100
Fig. S39. Antiproliferation properties of spirooxindoles **213** and Tanshinon IIA (TSA)………………………………………………………………………………. 105
Fig. S40. Antiproliferation and enzymation inhibitory properties of indole-triazole conjugates **216**, Etoposide, Indomethacin, Celecoxib, and Norhihydroguaiaretic acid (ND = not done)……………………………………… 107

Fig. S41. Antiproliferation and enzymation inhibitory properties of indole-triazole conjugates **218**, Etoposide, Indomethacin, Celecoxib, and Norhihydroguaiaretic acid (ND = not done)………………………………………… 111
Fig. S42. Antiproliferation properties (µM ± SD) of 3-[(indeno[1,2-*c*]pyrazole-3-yl)methylene]indolin-2-ones **221** and Combretastatin A-4 (CA-4)……………… 116
Fig. S43. Antiproliferation and tubulin polymerization inhibitory properties of the synthesized nicotinoyl/isonicotinyl pyrazolines featuring indolyl heterocycle **223** and Combretastatin A-4 (CA-4)…………………………………………………….. 119
Fig. S44. Antiproliferation properties (µM ± SD) of the synthesized indoles **225**, pyranoindole **226** and Vinblastine………………………………………………… 122
Fig. S45. Antiproferation and inhibitory properties of HDAC and BRD4 of the synthesized indole-isoxazole conjugates **235**, Vorinostat and JQ1……………… 123
Fig. S46. Antiproliferation properties of 3,6-disubstituted-2-carboxyindoles **241**………………………………………………………………………………. 127

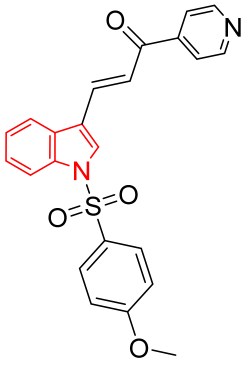

50a

IC_{50} = 65.8 ± 1.35, 26.0 ± 2.47 μM against MCF7 and HepG2, respectively; IC_{50} = 26.15, 29.35 μM against hCA IX, hCA II, respectively.

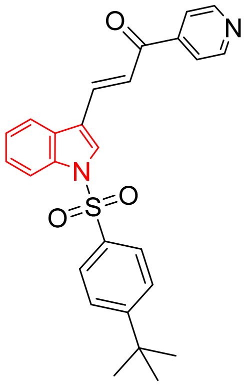

50b

IC_{50} = 24.0 ± 1.16, 27 ± 1.67 μM against MCF7 and HepG2, respectively; IC_{50} = 0.15, 12.10 μM against hCA IX, hCA II, respectively.

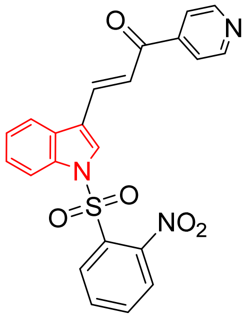

50c

IC_{50} = 36.9 ± 0.79, 31.7 ± 1.10 μM against MCF7 and HepG2, respectively; IC_{50} = 57.31, 36.21 μM against hCA IX, hCA II, respectively.

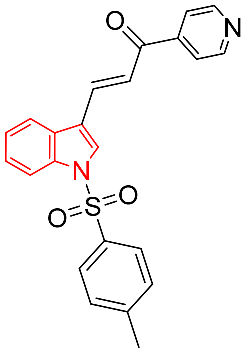

50d

IC_{50} = 48.9 ± 1.19, 44.8 ± 1.35 μM against MCF7 and HepG2, respectively; IC_{50} = 29.9, 38.40 μM against hCA IX, hCA II, respectively.

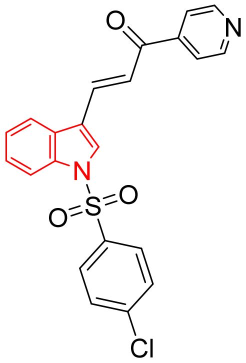

50e

IC_{50} = 37.5 ± 1.34, 35.1 ± 1.47 μM against MCF7 and HepG2, respectively; IC_{50} = 14.41, 13.50 μM against hCA IX, hCA II, respectively.

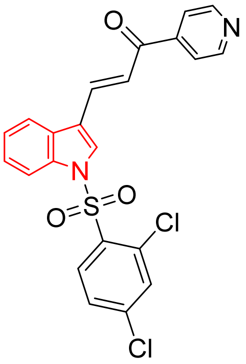

50f

IC_{50} = 12.2 ± 1.02, 14.8 ± 1.28 μM against MCF7 and HepG2, respectively; IC_{50} = 0.13, 9.10 μM against hCA IX, hCA II, respectively.

Fig. S1. Antiproliferation properties (μM ± SD) and carbonic anhydrase inhibitory activity (μM) of the prepared chalcones **50** and standard references (Doxorubicin and AZM).

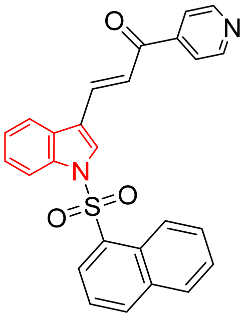

50g

IC_{50} = 58.0 ± 2.18, 62.9 ± 1.34 µM against MCF7 and HepG2, respectively; IC_{50} = 44.08, 65.22 µM against hCA IX, hCA II, respectively.

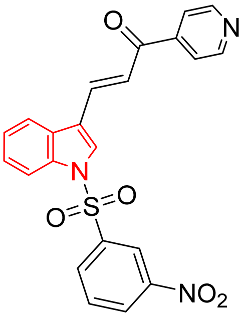

50h

IC_{50} = 36.3 ± 1.16, 42.6 ± 1.41 µM against MCF7 and HepG2, respectively; IC_{50} = 37.56, 24.32 µM against hCA IX, hCA II, respectively.

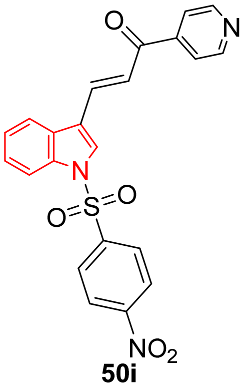

50i

IC_{50} = 14.5 ± 1.15, 18.3 ± 1.44 µM against MCF7 and HepG2, respectively; IC_{50} = 0.15, 14.10 µM against hCA IX, hCA II, respectively.

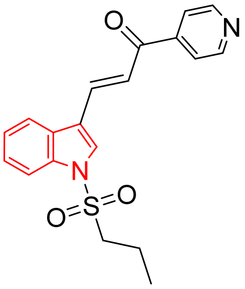

50j

IC_{50} = 52.4 ± 1.22, 46.2 ± 1.14 µM against MCF7 and HepG2, respectively; IC_{50} = 44.52, >100.00 µM against hCA IX, hCA II, respectively.

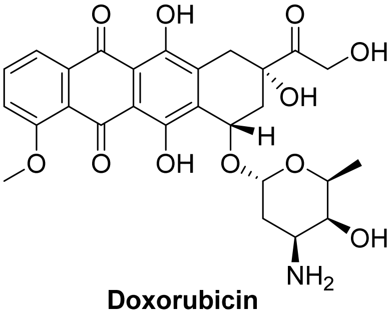

Doxorubicin

IC_{50} = 20.2 ± 1.70, 18.7 ± 1.37, 142.8 ± 1.62 µM against MCF7, HepG2, and HEK-293 respectively.

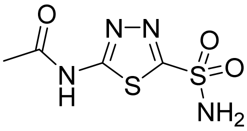

Acetazolamide (AZM)

IC_{50} = 0.027, 0.013 µM against hCA IX, hCA II, respectively.

Fig. S1 (continued). Antiproliferation properties (µM ± SD) and carbonic anhydrase inhibitory activity (µM) of the prepared chalcones **50** and standard references (Doxorubicin and AZM).

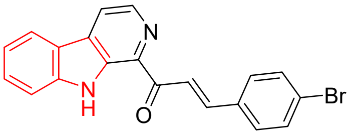

54a

IC_{50} = 3.54 ± 0.10, 9.21 ± 0.08, >20, 9.73 ± 0.23, 12.18 ± 0.16, >10, >20 μM against MCF-7, MDA-MB-231, HepG2, HT29, A549, PC-3 and L02, respectively

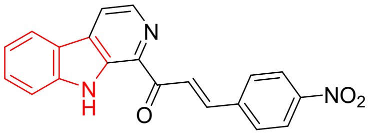

54b

IC_{50} = 5.87 ± 0.05, 0.62 ± 0.05, >20, 3.96 ± 0.21, >20, >10, >20 μM against MCF-7, MDA-MB-231, HepG2, HT29, A549, PC-3 and L02, respectively

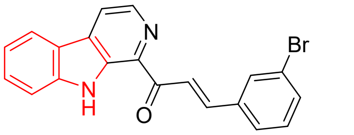

54c

IC_{50} = 4.99 ± 0.13, 6.44 ± 0.17, 5.89 ± 0.21, 7.24 ± 0.10, 11.78 ± 0.07, 2.69 ± 0.08, 14.26 ± 0.08 μM against MCF-7, MDA-MB-231, HepG2, HT29, A549, PC-3 and L02, respectively

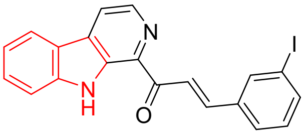

54d

IC_{50} = 1.60 ± 0.07, 5.17 ± 0.12, >20, >20, >20, >10, >20 μM against MCF-7, MDA-MB-231, HepG2, HT29, A549, PC-3 and L02, respectively

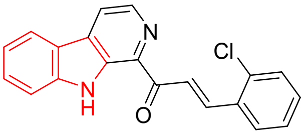

54e

IC_{50} = 3.35 ± 0.11, 3.43 ± 0.14, 2.8 ± 0.28, 12.51 ± 0.22, 5.37 ± 0.09, 2.39 ± 0.08, 11.37 ± 0.12 μM against MCF-7, MDA-MB-231, HepG2, HT29, A549, PC-3 and L02, respectively

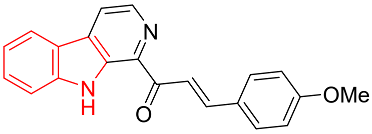

54f

IC_{50} = 17.34 ± 0.03, >20, >20, 18.97 ± 0.19, >20, >10, >20 μM against MCF-7, MDA-MB-231, HepG2, HT29, A549, PC-3 and L02, respectively

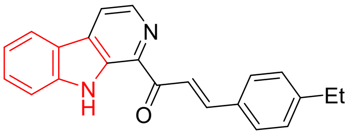

54g

IC_{50} = >20, >20, >20, >20, >20, >10, >20 μM against MCF-7, MDA-MB-231, HepG2, HT29, A549, PC-3 and L02, respectively

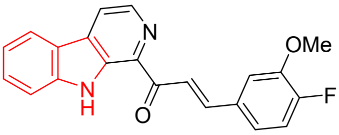

54h

IC_{50} = 2.38 ± 0.2, 3.25 ± 0.11, 5.7 ± 0.35, 10.86 ± 0.11, 3.07 ± 0.14, 1.02 ± 0.2, >20 μM against MCF-7, MDA-MB-231, HepG2, HT29, A549, PC-3 and L02, respectively

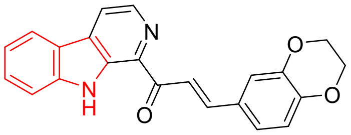

54i

IC_{50} = >20, 15.18 ± 0.16, 13.21 ± 0.08, >20, >20, >10, >20 μM against MCF-7, MDA-MB-231, HepG2, HT29, A549, PC-3 and L02, respectively

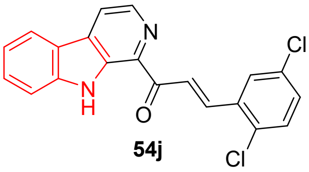

54j

IC_{50} = 0.59 ± 0.06, 2.77 ± 0.33, 3.37 ± 0.16, 3.14 ± 0.14, 6.78 ± 0.14, 1.40 ± 0.11, 9.91 ± 0.12 μM against MCF-7, MDA-MB-231, HepG2, HT29, A549, PC-3 and L02, respectively

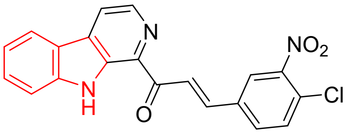

54k

IC_{50} = 0.34 ± 0.01, 0.98 ± 0.16, 1.61 ± 0.15, 0.57 ± 0.03, 2.02 ± 0.14, 1.17 ± 0.01, 9.61 ± 0.13 μM against MCF-7, MDA-MB-231, HepG2, HT29, A549, PC-3 and L02, respectively

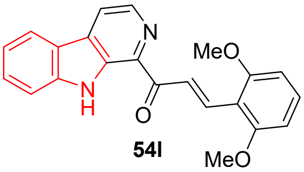

54l

IC_{50} = 10.45 ± 0.18, >20, >20, >20, >20, >10, >20 μM against MCF-7, MDA-MB-231, HepG2, HT29, A549, PC-3 and L02, respectively

Fig. S2. Antiproliferation properties (μM ± SD) of the synthesized agents **54**, **55**, Camptothecin and Harmine.

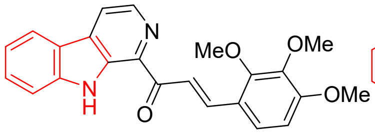

54m

IC_{50} = >20, >20, 11.55 ± 0.25, >20, >20, >10, >20 μM against MCF-7, MDA-MB-231, HepG2, HT29, A549, PC-3 and L02, respectively

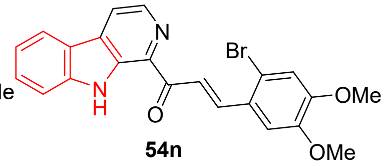

54n

IC_{50} = 2.21 ± 0.05, 7.03 ± 0.14, 9.65 ± 0.32, 3.75 ± 0.14, 9.83 ± 0.15, >10, >20 μM against MCF-7, MDA-MB-231, HepG2, HT29, A549, PC-3 and L02, respectively

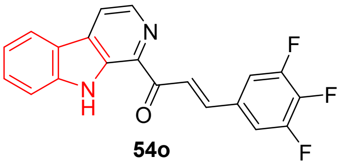

54o

IC_{50} = 0.88 ± 0.13, 2.05 ± 0.11, 0.96 ± 0.03, 0.89 ± 0.14, 5.73 ± 0.13, 1.19 ± 0.08, 17.78 ± 0.10 μM against MCF-7, MDA-MB-231, HepG2, HT29, A549, PC-3 and L02, respectively

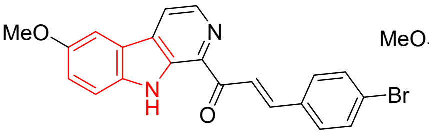

54p

IC_{50} = 8.45 ± 0.04, 5.67 ± 0.19, 15.68 ± 0.34, 7.74 ± 0.14, >20, 4.18 ± 0.02, >20 μM against MCF-7, MDA-MB-231, HepG2, HT29, A549, PC-3 and L02, respectively

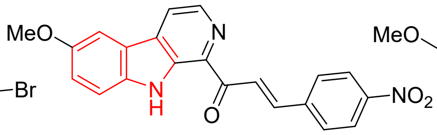

54q

IC_{50} = 15.48 ± 0.06, >20, >20, >20, >20, >10, >20 μM against MCF-7, MDA-MB-231, HepG2, HT29, A549, PC-3 and L02, respectively

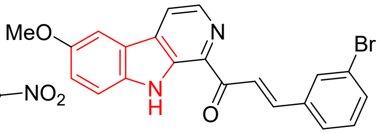

54r

IC_{50} = >20, >20, >20, 0.49 ± 0.03, >20, >10, >20 μM against MCF-7, MDA-MB-231, HepG2, HT29, A549, PC-3 and L02, respectively

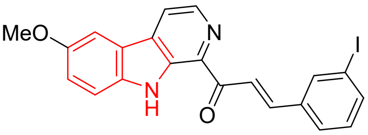

54s

IC_{50} = 1.58 ± 0.06, 8.71 ± 0.08, >20, 2.34 ± 0.1, >20, >10, >20 μM against MCF-7, MDA-MB-231, HepG2, HT29, A549, PC-3 and L02, respectively

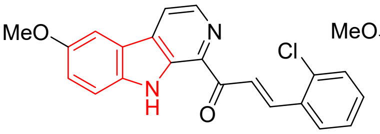

54t

IC_{50} = >20, 1.43 ± 0.16, >20, 0.52 ± 0.08, >20, >10, >20 μM against MCF-7, MDA-MB-231, HepG2, HT29, A549, PC-3 and L02, respectively

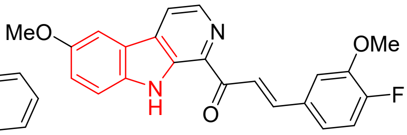

54u

IC_{50} = 0.77 ± 0.12, 1.41 ± 0.08, 6.33 ± 0.26, 2.47 ± 0.14, 6.48 ± 0.06, 0.99 ± 0.08, >20 μM against MCF-7, MDA-MB-231, HepG2, HT29, A549, PC-3 and L02, respectively

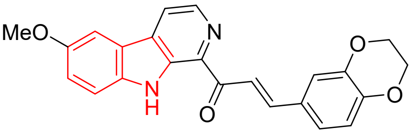

54v

IC_{50} = 14.46 ± 0.25, 18.08 ± 0.08, 15.6 ± 0.21, >20, >20, 3.79 ± 0.05, >20 μM against MCF-7, MDA-MB-231, HepG2, HT29, A549, PC-3 and L02, respectively

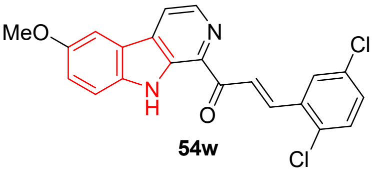

54w

IC_{50} = 7.95 ± 0.18, 8.13 ± 0.12, >20, 0.68 ± 0.02, >20, >10, >20 μM against MCF-7, MDA-MB-231, HepG2, HT29, A549, PC-3 and L02, respectively

Fig. S2 (continued). Antiproliferation properties (μM ± SD) of the synthesized agents **54**, **55**, Camptothecin and Harmine.

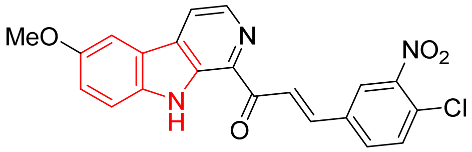

54x

IC_{50} = >20, 2.99 ± 0.28, 7.72 ± 0.16, >20, 5.72 ± 0.21, >10, >20 μM against MCF-7, MDA-MB-231, HepG2, HT29, A549, PC-3 and L02, respectively

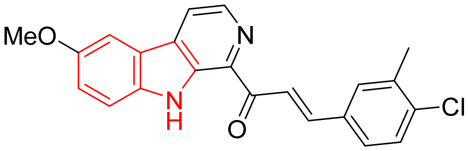

54y

IC_{50} = 4.38 ± 0.2, 6.45 ± 0.11, 2.74 ± 0.17, 1.89 ± 0.21, 7.49 ± 0.10, >10, >20 μM against MCF-7, MDA-MB-231, HepG2, HT29, A549, PC-3 and L02, respectively

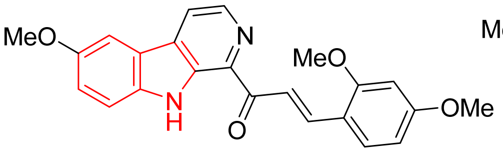

54z

IC_{50} = 5.71 ± 0.08, 11.98 ± 0.27, 7.13 ± 0.26, 13.59 ± 0.21, 15.3 ± 0.13, >10, >20 μM against MCF-7, MDA-MB-231, HepG2, HT29, A549, PC-3 and L02, respectively

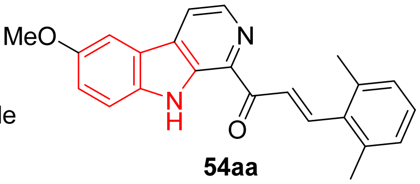

54aa

IC_{50} = 10.14 ± 0.18, >20, >20, >20, >20, >10, >20 μM against MCF-7, MDA-MB-231, HepG2, HT29, A549, PC-3 and L02, respectively

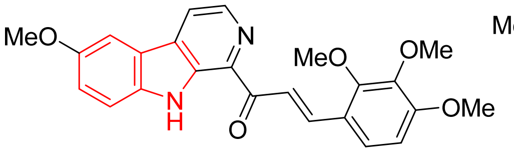

54ab

IC_{50} = 6.1 ± 0.14, >20, >20, 6.08 ± 0.20, 7.1 ± 0.16, >10, >20 μM against MCF-7, MDA-MB-231, HepG2, HT29, A549, PC-3 and L02, respectively

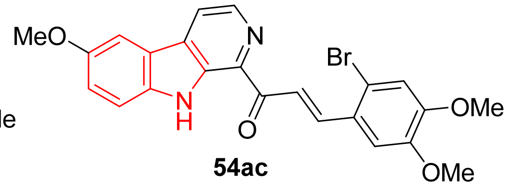

54ac

IC_{50} = 8.05 ± 0.25, >20, >20, 11.34 ± 0.17, 4.51 ± 0.13, >10, >20 μM against MCF-7, MDA-MB-231, HepG2, HT29, A549, PC-3 and L02, respectively

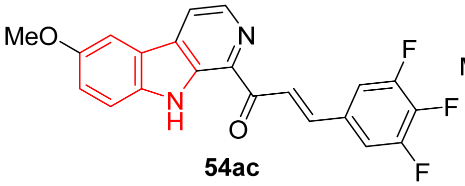

54ac

IC_{50} = 4.23 ± 0.09, 6.52 ± 0.23, 15.94 ± 0.31, 2.72 ± 0.04, >20, >10, >20 μM against MCF-7, MDA-MB-231, HepG2, HT29, A549, PC-3 and L02, respectively

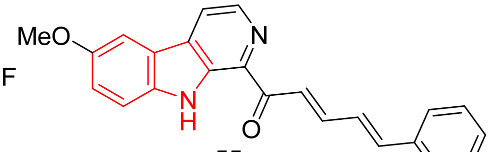

55

IC_{50} = 17.53 ± 0.02, >20, 18.93 ± 0.23, >20, >20, >10, >20 μM against MCF-7, MDA-MB-231, HepG2, HT29, A549, PC-3 and L02, respectively

Fig. S2 (continued). Antiproliferation properties (μM ± SD) of the synthesized agents **54**, **55**, Camptothecin and Harmine.

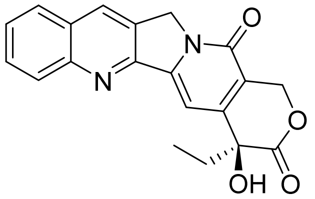

Camptothecin

IC_{50} = 6.85 ± 0.11, 0.62 ± 0.27, 1.80 ± 0.14, 1.87 ± 0.09, 7.80 ± 0.14, 6.60 ± 0.29, 0.59 ± 0.08 μM against MCF-7, MDA-MB-231, HepG2, HT29, A549, PC-3 and L02, respectively

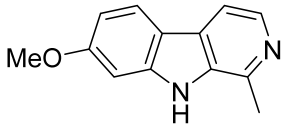

Harmine

IC_{50} = 13.37 ± 0.05, 13.21 ± 0.08, 19.23 ± 0.19, 12.15 ± 0.18, 17.3 ± 0.04, 15.57 ± 0.10, 9.49 ± 0.11 μM against MCF-7, MDA-MB-231, HepG2, HT29, A549, PC-3 and L02, respectively

Fig. S2 (continued). Antiproliferation properties (μM ± SD) of the synthesized agents **54**, **55**, Camptothecin and Harmine.

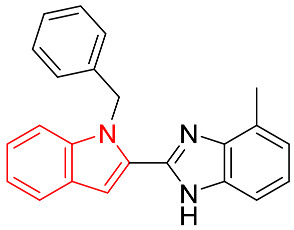

58a

$IC_{50} > 25\ \mu M$
against T47D cell line;
$IC_{50} = 387.37 \pm 13.12$ nM
binding affinity with ER-α

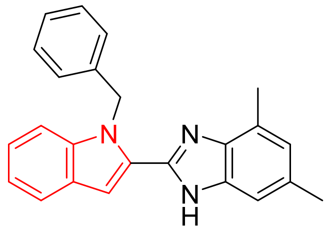

58b

$IC_{50} = 21.8 \pm 1.86\ \mu M$
against T47D cell line;
$IC_{50} = 33.13 \pm 5.37$ nM
binding affinity with ER-α

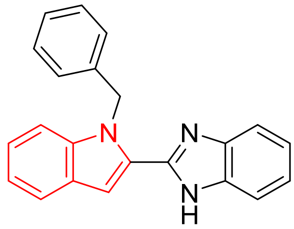

58c

$IC_{50} = 21.45 \pm 0.60\ \mu M$
against T47D cell line;
$IC_{50} = 259.16 \pm 26.42$ nM
binding affinity with ER-α

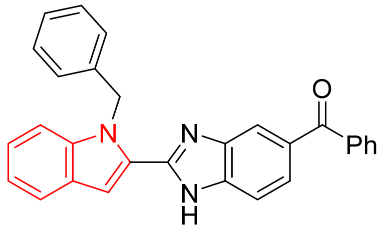

58d

$IC_{50} > 25 \mu M$ against
T47D cell line;
$IC_{50} > 1000$ nM
binding affinity with ER-α

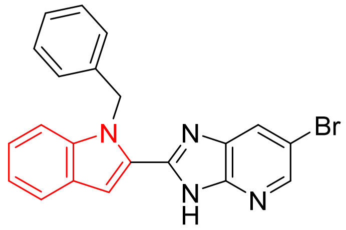

58e

$IC_{50} > 25 \mu M$ against
T47D cell line;
$IC_{50} = 300.43 \pm 10.40$ nM
binding affinity with ER-α

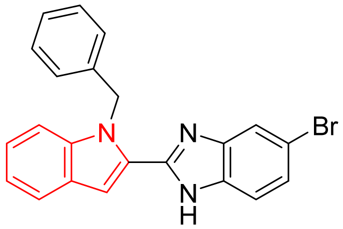

58f

$IC_{50} = 15.48 \pm 0.10\ \mu M$
against T47D cell line;
$IC_{50} = 73.61 \pm 3.25$ nM
binding affinity with ER-α

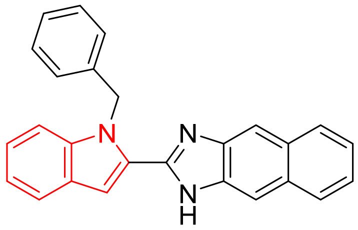

58g

$IC_{50} = 13.44 \pm 0.04\ \mu M$
against T47D cell line;
$IC_{50} > 1000$ nM
binding affinity with ER-α

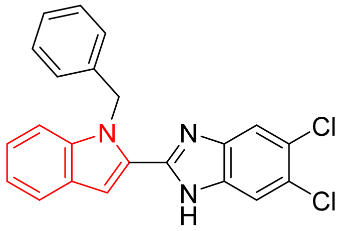

58h

$IC_{50} = 22.5 \pm 2.12\ \mu M$
against T47D cell line;
$IC_{50} = 76.73 \pm 6.35$ nM
binding affinity with ER-α

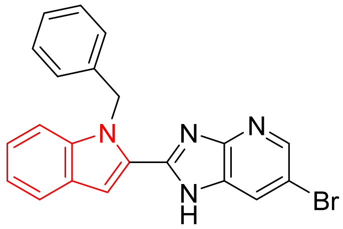

58i

$IC_{50} > 25 \mu M$ against
T47D cell line;
$IC_{50} = 539.18 \pm 34.79$ nM
binding affinity with ER-α

Fig. S3. Antiproliferation properties (μM) and binding affinity with ER-α (nM) of the synthesized indole-benzimidazole conjugates **58**.

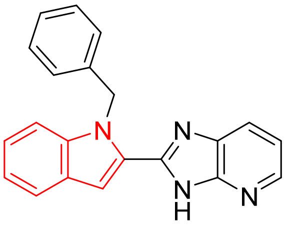

58j

IC_{50} > 25μM against T47D cell line;
IC_{50} > 1000 nM binding affinity with ER-α

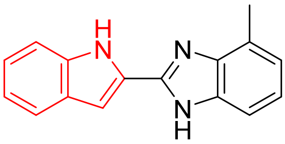

58k

IC_{50} = 8.9 ± 0.57 μM against T47D cell line;
IC_{50} = 191.69 ± 14.99 nM binding affinity with ER-α

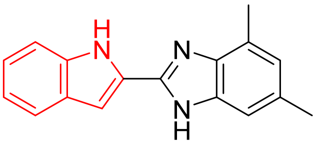

58l

IC_{50} = 24.8 ± 0.66 μM against T47D cell line;
IC_{50} > 1000 nM binding affinity with ER-α

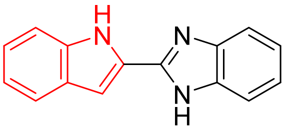

58m

IC_{50} = 6.2 ± 0.88 μM against T47D cell line;
IC_{50} > 1000 nM binding affinity with ER-α

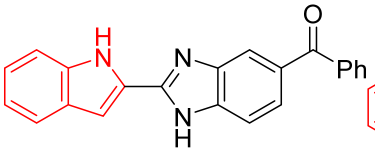

58n

IC_{50} > 25μM against T47D cell line;
IC_{50} = 46.88 ± 0.95 nM binding affinity with ER-α

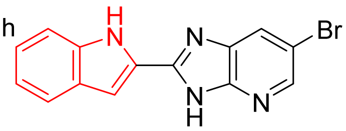

58o

IC_{50} = 21.03 ± 0.03 μM against T47D cell line;
IC_{50} > 1000 nM binding affinity with ER-α

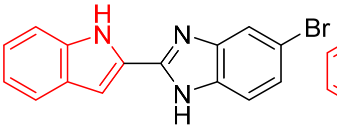

58p

IC_{50} = 4.99 ± 0.60 μM against T47D cell line;
IC_{50} = 80.36 ± 7.02 nM binding affinity with ER-α

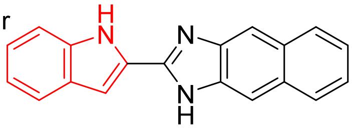

58q

IC_{50} = 23.42 ± 0.07 μM against T47D cell line;
IC_{50} > 1000 nM binding affinity with ER-α

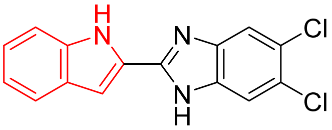

58r

IC_{50} = 21.16 ± 1.59 μM against T47D cell line;
IC_{50} > 1000 nM binding affinity with ER-α

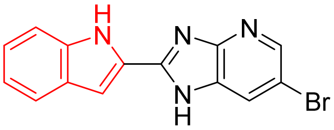

58s

IC_{50} = 24.4 ± 0.84 μM against T47D cell line;
IC_{50} > 1000 nM binding affinity with ER-α

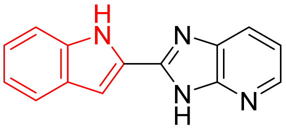

58t

IC_{50} > 25μM against T47D cell line;
IC_{50} > 1000 nM binding affinity with ER-α

Fig. S3 (continued). Antiproliferation properties (μM) and binding affinity with ER-α (nM) of the synthesized indole-benzimidazole conjugates **58**.

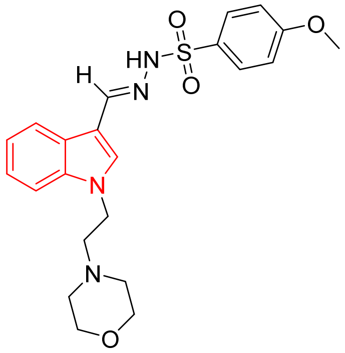

66a

IC_{50} = 62.75, 43.32 μM against MCF7 and MDA-MB468, respectively

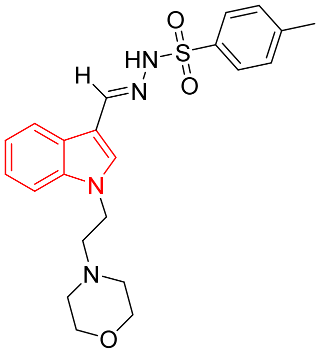

66b

IC_{50} = 61.14, 40.81 μM against MCF7 and MDA-MB468, respectively

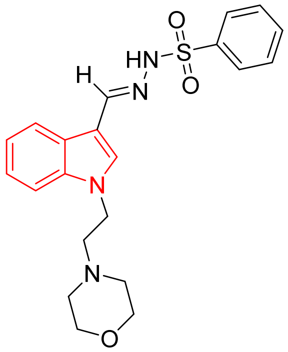

66c

IC_{50} = 73.15, 47.02 μM against MCF7 and MDA-MB468, respectively

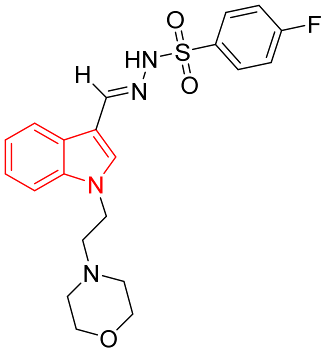

66d

IC_{50} = 32.59, 15.00 μM against MCF7 and MDA-MB468, respectively

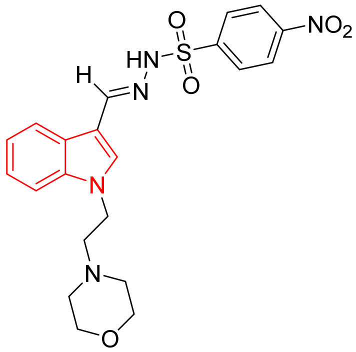

66e

IC_{50} = 82.03, 37.07 μM against MCF7 and MDA-MB468, respectively

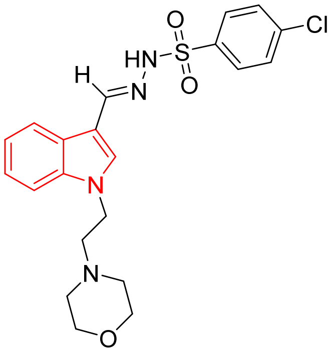

66f

IC_{50} = 13.2, 8.2 μM against MCF7 and MDA-MB468, respectively

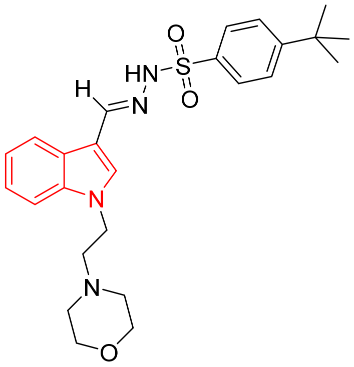

66g

IC_{50} = 67.84, 29.63 μM against MCF7 and MDA-MB468, respectively

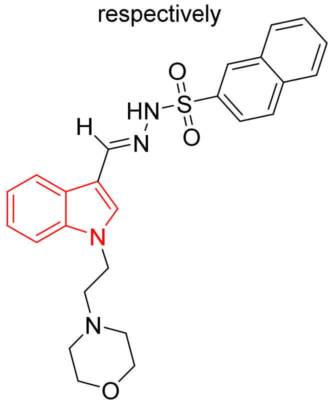

66h

IC_{50} = 29.93, 53.38 μM against MCF7 and MDA-MB468, respectively

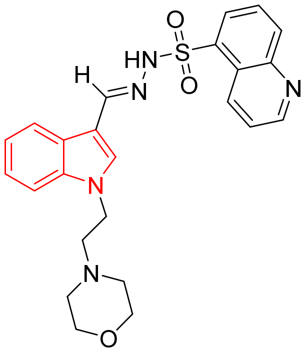

66i

IC_{50} = 79.13, 32.20 μM against MCF7 and MDA-MB468, respectively

Fig. S4. Antiproliferation properties of the synthesized indolyl sulfonohydrazones **66** and Doxorubicin.

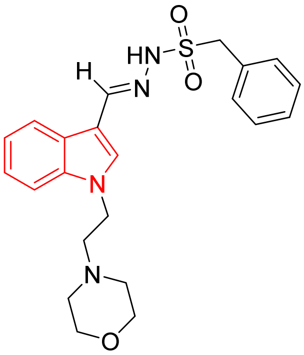

66j

IC_{50} = 62.53, 50.63 μM against MCF7 and MDA-MB468, respectively

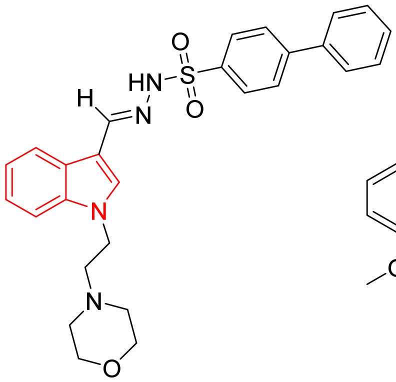

66k

IC_{50} = 17.30, 31.98 μM against MCF7 and MDA-MB468, respectively

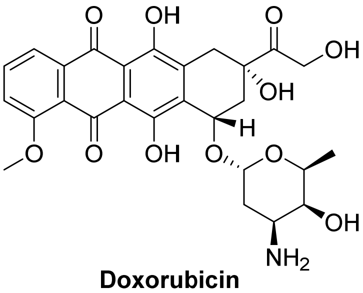

Doxorubicin

IC_{50} = 0.06, 0.08 μM against MCF7 and MDA-MB468, respectively

Fig. S4 (continued). Antiproliferation properties of the synthesized indolyl sulfonohydrazones **66** and Doxorubicin.

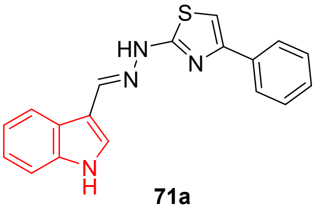

71a

IC_{50} = 3.41 ± 0.12, 2.87 ± 0.25, 3.25 ± 0.23 μM against MCF-7, A549 and Hela cell lines, respectively; IC_{50} = 14.77 ± 0.24 μM of tubulin polymerization inhibition

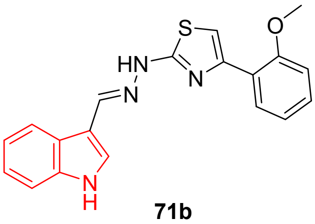

71b

IC_{50} = 8.68 ± 0.03, 7.06 ± 0.13, 8.22 ± 0.45 μM against MCF-7, A549 and Hela cell lines, respectively; IC_{50} = 28.72 ± 0.16 μM of tubulin polymerization inhibition

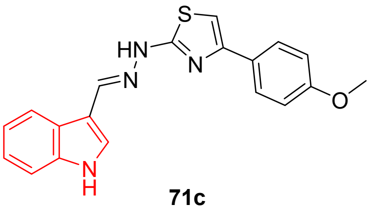

71c

IC_{50} = 6.26 ± 0.05, 5.48 ± 0.03, 5.78 ± 0.02 μM against MCF-7, A549 and Hela cell lines, respectively; IC_{50} = 27.11 ± 0.08 μM of tubulin polymerization inhibition

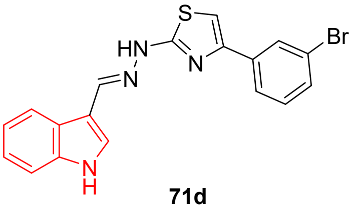

71d

IC_{50} = 0.57 ± 0.06, 0.28 ± 0.21, 0.57 ± 0.15 μM against MCF-7, A549 and Hela cell lines, respectively; IC_{50} = 2.61 ± 0.17 μM of tubulin polymerization inhibition

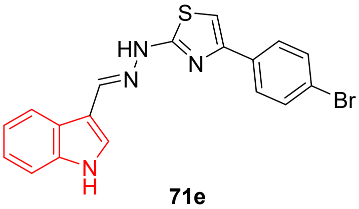

71e

IC_{50} = 0.62 ± 0.06, 0.34 ± 0.12, 0.64 ± 0.08 μM against MCF-7, A549 and Hela cell lines, respectively; IC_{50} = 2.95 ± 0.26 μM of tubulin polymerization inhibition

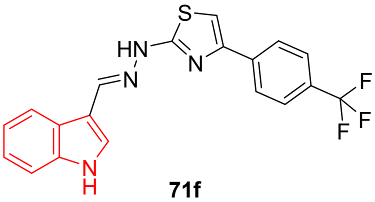

71f

IC_{50} = 1.58 ± 0.32, 1.32 ± 0.22, 1.46 ± 0.21 μM against MCF-7, A549 and Hela cell lines, respectively; IC_{50} = 8.06 ± 0.12 μM of tubulin polymerization inhibition

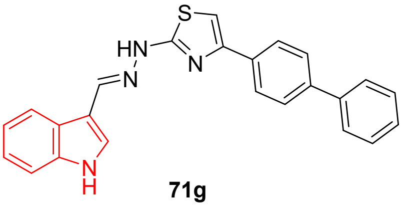

71g

IC_{50} = 8.94 ± 0.07, 8.06 ± 0.04, 8.35 ± 0.11 μM against MCF-7, A549 and Hela cell lines, respectively; IC_{50} = 31.45 ± 0.36 μM of tubulin polymerization inhibition

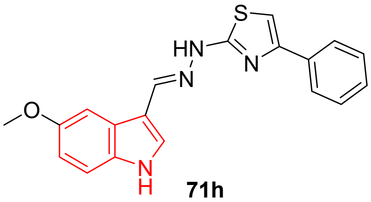

71h

IC_{50} = 2.38 ± 0.03, 1.65 ± 0.21, 1.69 ± 0.15 μM against MCF-7, A549 and Hela cell lines, respectively; IC_{50} = 9.67 ± 0.27 μM of tubulin polymerization inhibition

Fig. S5. Antiproliferation and tubulin polymerization inhibitory properties (μM ± SD) of the synthesized thiazolyl hydrazones linked to indolyl scaffold **71**, Colchicine and CA-4 (control drugs).

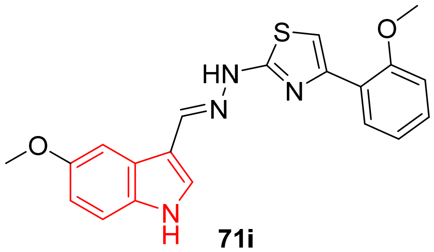

71i

IC_{50} = 5.82 ± 0.08, 5.09 ± 0.04, 5.52 ± 0.18 μM against MCF-7, A549 and Hela cell lines, respectively; IC_{50} = 24.62 ± 0.19 μM of tubulin polymerization inhibition

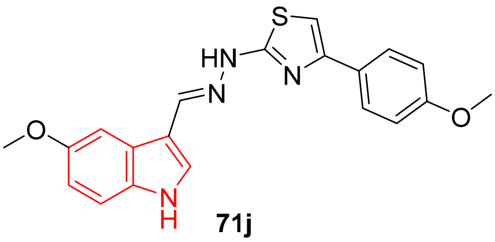

71j

IC_{50} = 5.29 ± 0.11, 4.86 ± 0.07, 5.08 ± 0.25 μM against MCF-7, A549 and Hela cell lines, respectively; IC_{50} = 24.13 ± 0.02 μM of tubulin polymerization inhibition

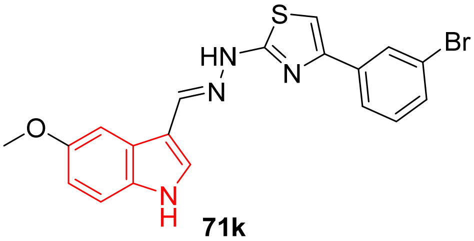

71k

IC_{50} = 0.46 ± 0.13, 0.21 ± 0.02, 0.32 ± 0.26 μM against MCF-7, A549 and Hela cell lines, respectively; IC_{50} = 1.68 ± 0.07 μM of tubulin polymerization inhibition

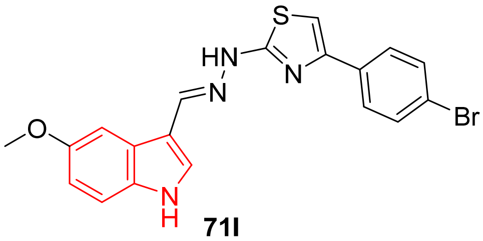

71l

IC_{50} = 0.50 ± 0.04, 0.23 ± 0.42, 0.45 ± 0.04 μM against MCF-7, A549 and Hela cell lines, respectively; IC_{50} = 1.96 ± 0.04 μM of tubulin polymerization inhibition

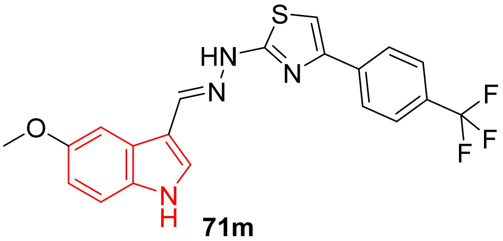

71m

IC_{50} = 1.23 ± 0.35, 0.94 ± 0.15, 1.17 ± 0.11 μM against MCF-7, A549 and Hela cell lines, respectively; IC_{50} = 6.42 ± 0.32 μM of tubulin polymerization inhibition

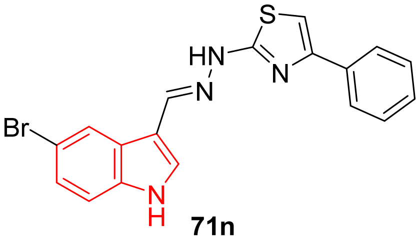

71n

IC_{50} = 5.09 ± 0.07, 4.72 ± 0.12, 4.94 ± 0.09 μM against MCF-7, A549 and Hela cell lines, respectively; IC_{50} = 22.35 ± 0.15 μM of tubulin polymerization inhibition

Fig. S5 (continued). Antiproliferation and tubulin polymerization inhibitory properties (μM ± SD) of the synthesized thiazolyl hydrazones linked to indolyl scaffold **71**, Colchicine and CA-4 (control drugs).

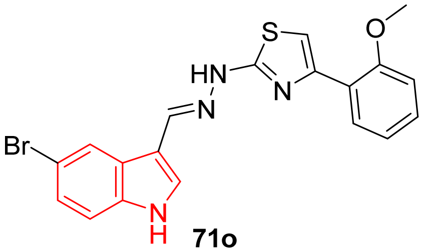

71o

IC_{50} = 11.32 ± 0.26, 9.45 ± 0.05, 10.33 ± 0.12 μM against MCF-7, A549 and Hela cell lines, respectively; IC_{50} = 38.40 ± 0.06 μM of tubulin polymerization inhibition

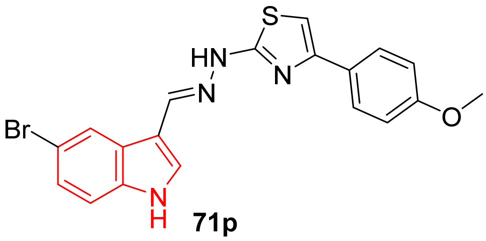

71p

IC_{50} = 9.23 ± 0.07, 8.44 ± 0.07, 8.81 ± 0.06 μM against MCF-7, A549 and Hela cell lines, respectively; IC_{50} = 35.26 ± 0.27 μM of tubulin polymerization inhibition

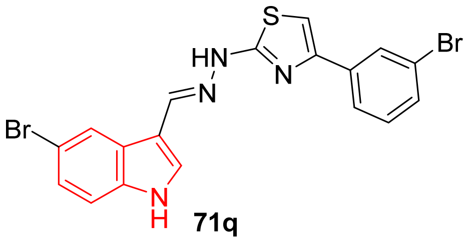

71q

IC_{50} = 0.77 ± 0.03, 0.72± 0.15, 0.75 ± 0.08 μM against MCF-7, A549 and Hela cell lines, respectively; IC_{50} = 4.66 ± 0.04 μM of tubulin polymerization inhibition

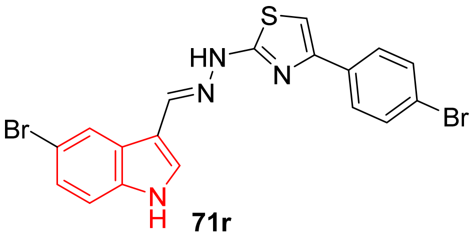

71r

IC_{50} = 0.84 ± 0.26, 0.79 ± 0.34, 0.82 ± 0.13 μM against MCF-7, A549 and Hela cell lines, respectively; IC_{50} = 5.77 ± 0.23 μM of tubulin polymerization inhibition

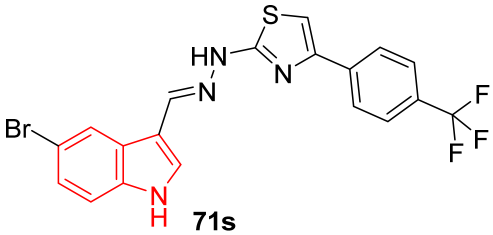

71s

IC_{50} = 4.86 ± 0.08, 3.75 ± 0.12, 4.39 ± 0.21 μM against MCF-7, A549 and Hela cell lines, respectively; IC_{50} = 21.32 ± 0.08 μM of tubulin polymerization inhibition

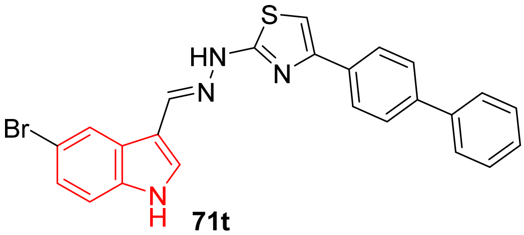

71t

IC_{50} = 14.24 ± 0.13, 10.42 ± 0.37, 11.44 ± 0.18 μM against MCF-7, A549 and Hela cell lines, respectively; IC_{50} = 41.33 ± 0.11 μM of tubulin polymerization inhibition

Fig. S5 (continued). Antiproliferation and tubulin polymerization inhibitory properties (μM ± SD) of the synthesized thiazolyl hydrazones linked to indolyl scaffold **71**, Colchicine and CA-4 (control drugs).

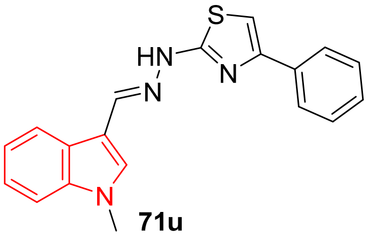

71u

IC_{50} = 4.67 ± 0.08, 3.30 ± 0.05, 3.42 ± 0.09 μM against MCF-7, A549 and Hela cell lines, respectively; IC_{50} = 17.21 ± 0.03 μM of tubulin polymerization inhibition

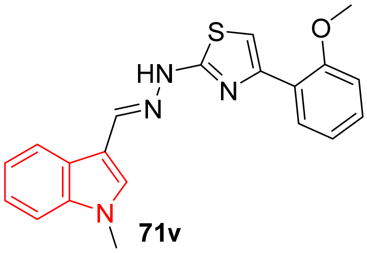

71v

IC_{50} = 9.86 ± 0.15, 8.56 ± 0.33, 8.83 ± 0.22 μM against MCF-7, A549 and Hela cell lines, respectively; IC_{50} = 36.18 ± 0.09 μM of tubulin polymerization inhibition

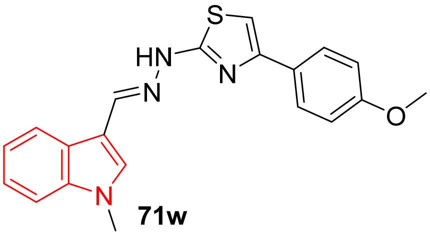

71w

IC_{50} = 7.08 ± 0.16, 5.78 ± 0.08, 5.86 ± 0.14 μM against MCF-7, A549 and Hela cell lines, respectively; IC_{50} = 28.16 ± 0.22 μM of tubulin polymerization inhibition

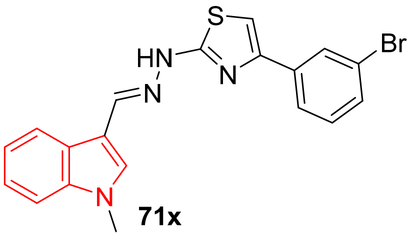

71x

IC_{50} = 0.69 ± 0.26, 0.61 ± 0.19, 0.68 ± 0.15 μM against MCF-7, A549 and Hela cell lines, respectively; IC_{50} = 3.12 ± 0.42 μM of tubulin polymerization inhibition

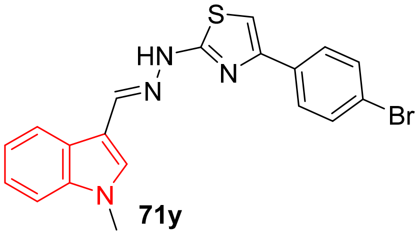

71y

IC_{50} = 0.78 ± 0.25, 0.76 ± 0.22, 0.76 ± 0.13 μM against MCF-7, A549 and Hela cell lines, respectively; IC_{50} = 4.86 ± 0.31 μM of tubulin polymerization inhibition

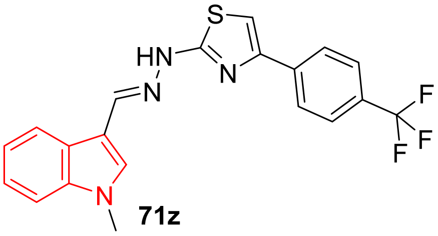

71z

IC_{50} = 2.84 ± 0.09, 1.85 ± 0.04, 1.93 ± 0.23 μM against MCF-7, A549 and Hela cell lines, respectively; IC_{50} = 11.43 ± 0.15 μM of tubulin polymerization inhibition

Fig. S5 (continued). Antiproliferation and tubulin polymerization inhibitory properties (μM ± SD) of the synthesized thiazolyl hydrazones linked to indolyl scaffold **71**, Colchicine and CA-4 (control drugs).

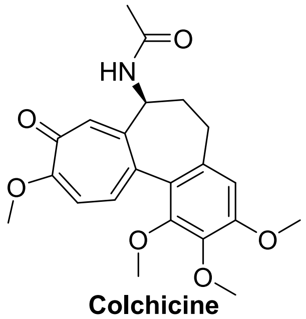

Colchicine

IC_{50} = 0.75 ± 0.08, 0.68 ± 0.12, 0.72 ± 0.16 μM against MCF-7, A549 and Hela cell lines, respectively; IC_{50} = 3.28 ± 0.25 μM of tubulin polymerization inhibition

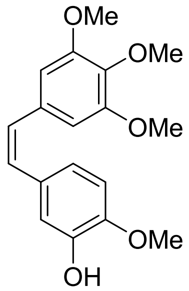

CA-4

IC_{50} = 0.52 ± 0.04, 0.24 ± 0.21, 0.48 ± 0.24 μM against MCF-7, A549 and Hela cell lines, respectively; IC_{50} = 2.12 ± 0.07 μM of tubulin polymerization inhibition

Fig. S5 (continued). Antiproliferation and tubulin polymerization inhibitory properties (μM ± SD) of the synthesized thiazolyl hydrazones linked to indolyl scaffold **71**, Colchicine and CA-4 (control drugs).

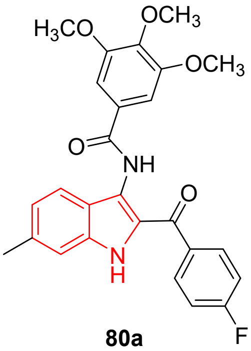

80a

IC_{50} = 8.89 ± 0.64, 5.17 ± 0.49, 8.66 ± 0.27, 5.72 ± 0.66, 6.16 ± 0.24, 8.95 ± 0.43 μM against MCF-7, MDA-MB-231, BT549, T47D, MDA-MB-468 and HS578T cell lines, respectively; % inhibition = 17 of tubulin polymerization at 10 μM

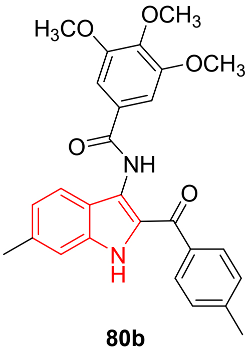

80b

IC_{50} = 69.48 ± 7.52, 33.43 ± 2.71, 69.75 ± 2.09, 40.16 ± 1.07, 75.10 ± 2.20, 43.68 ± 4.05 μM against MCF-7, MDA-MB-231, BT549, T47D, MDA-MB-468 and HS578T cell lines, respectively

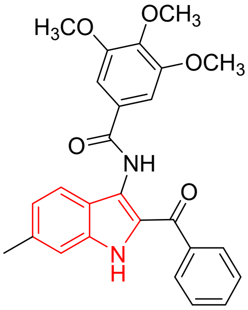

80c

IC_{50} = 32.74 ± 7.03, 16.60 ± 3.33, 42.26 ± 2.25, >100, >100, 49.06 ± 3.19 μM against MCF-7, MDA-MB-231, BT549, T47D, MDA-MB-468 and HS578T cell lines, respectively

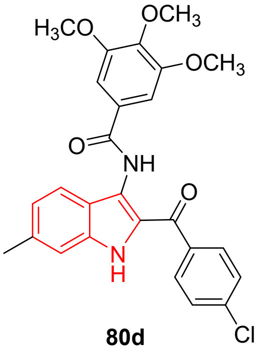

80d

IC_{50} = 3.85 ± 0.87, 3.22 ± 0.43, 4.81 ± 0.57, 6.25 ± 0.25, 4.12 ± 0.39, 3.59 ± 0.07 μM against MCF-7, MDA-MB-231, BT549, T47D, MDA-MB-468 and HS578T cell lines, respectively; % inhibition = 15 of tubulin polymerization at 10 μM

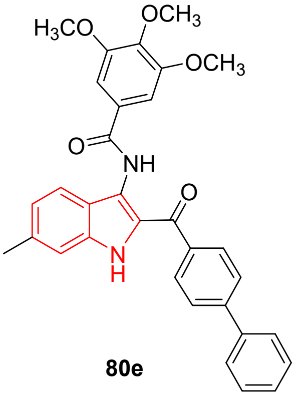

80e

IC_{50} = >100, 18.21 ± 2.23, >100, >100, >100, >100 μM against MCF-7, MDA-MB-231, BT549, T47D, MDA-MB-468 and HS578T cell lines, respectively

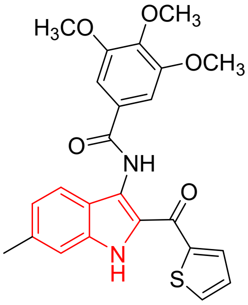

80f

IC_{50} = >100, >100, >100, >100, >100, >100 μM against MCF-7, MDA-MB-231, BT549, T47D, MDA-MB-468 and HS578T cell lines, respectively

Fig. S6. Antiproliferation (μM ± SD) and % inhibitory properties of tubulin polymerization at 10 μM of the synthesized 3-amidoindoles **80** and CA-4 (control drug).

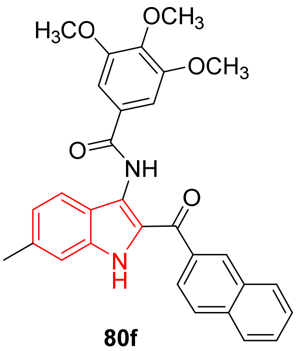

80f

IC_{50} = >100, >100, 39.75 ± 3.36, >100, >100, 53.32 ± 6.62 μM against MCF-7, MDA-MB-231, BT549, T47D, MDA-MB-468 and HS578T cell lines, respectively

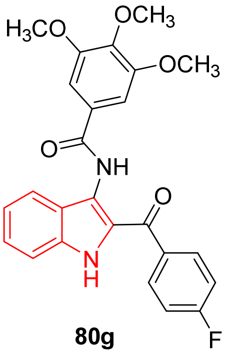

80g

IC_{50} = 77.92 ± 10.01, 50.76 ± 3.86, 42.58 ± 2.97, 60.86 ± 3.32, >100, >100 μM against MCF-7, MDA-MB-231, BT549, T47D, MDA-MB-468 and HS578T cell lines, respectively

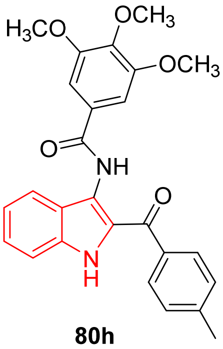

80h

IC_{50} = 50.00 ± 14.35, 12.61 ± 1.78, 17.07 ± 0.87, 20.06 ± 2.04, 14.52 ± 0.99, 50.49 ± 2.26 μM against MCF-7, MDA-MB-231, BT549, T47D, MDA-MB-468 and HS578T cell lines, respectively

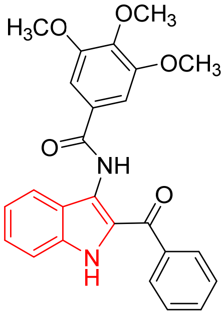

80i

IC_{50} = >100, 17.22 ± 2.04, >100, >100, >100, >100 μM against MCF-7, MDA-MB-231, BT549, T47D, MDA-MB-468 and HS578T cell lines, respectively

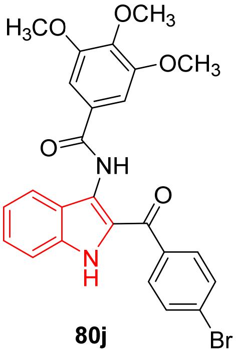

80j

IC_{50} = 7.14 ± 1.15, 6.47 ± 0.95, 10.31 ± 1.42, 9.55 ± 0.31, 7.87 ± 0.55, 7.52 ± 0.61 μM against MCF-7, MDA-MB-231, BT549, T47D, MDA-MB-468 and HS578T cell lines, respectively, % inhibition = 37 of tubulin polymerization at 10 μM

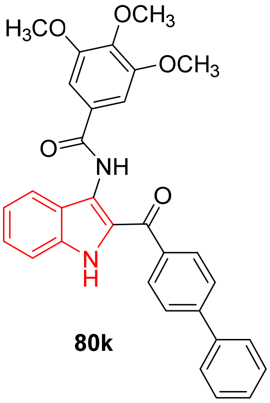

80k

IC_{50} = >100, >100, >100, >100, >100, 22.24 ± 1.42 μM against MCF-7, MDA-MB-231, BT549, T47D, MDA-MB-468 and HS578T cell lines, respectively

Fig. S6 (continued). Antiproliferation (μM ± SD) and % inhibitory properties of tubulin polymerization at 10 μM of the synthesized 3-amidoindoles **80** and CA-4 (control drug).

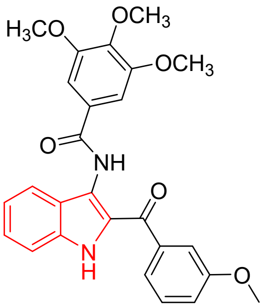

80l

IC_{50} = >100, >100,
65.53 ± 7.13, 53.39 ± 3.25,
>100, 9.06 ± 4.22 µM
against MCF-7, MDA-MB-231,
BT549, T47D, MDA-MB-468
and HS578T cell lines,
respectively

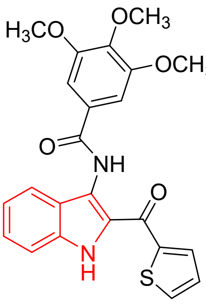

80m

IC_{50} = >100, >100,
>100, >100,
>100, 29.57 ± 1.13 µM
against MCF-7, MDA-MB-231,
BT549, T47D, MDA-MB-468
and HS578T cell lines,
respectively

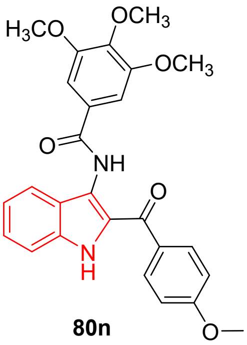

80n

IC_{50} = 10.70 ± 0.77, 34.96 ± 2.92,
10.37 ± 0.23, 5.56 ± 0.37,
7.09 ± 0.78, >100 µM
against MCF-7, MDA-MB-231,
BT549, T47D, MDA-MB-468
and HS578T cell lines,
respectively, % inhibition = 20
of tubulin polymerization at 10 µM

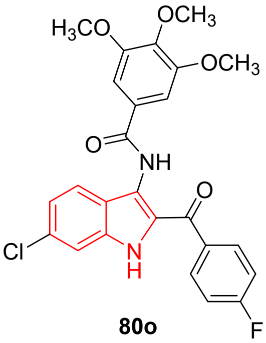

80o

IC_{50} = 61.08 ± 17.61, 46.65 ± 7.47,
35.54 ± 3.13, 76.17 ± 2.93,
>100, >100 µM
against MCF-7, MDA-MB-231,
BT549, T47D, MDA-MB-468
and HS578T cell lines,
respectively

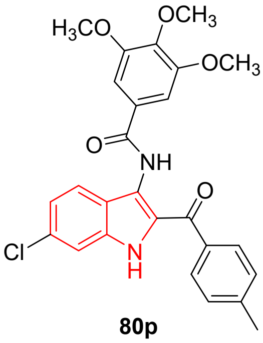

80p

IC_{50} = 34.33 ± 2.98, 33.65 ± 0.61,
25.05 ± 2.09, 16.65 ± 0.34,
5.79 ± 0.09, 14.19 ± 0.54 µM
against MCF-7, MDA-MB-231,
BT549, T47D, MDA-MB-468
and HS578T cell lines,
respectively, % inhibition = 36
of tubulin polymerization at 10 µM

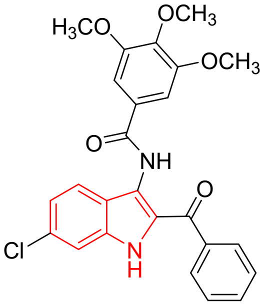

80q

IC_{50} = 51.78 ± 5.84, 32.66 ± 5.27,
26.89 ± 1.78, 45.51 ± 2.69,
11.04 ± 0.19, >100 µM
against MCF-7, MDA-MB-231,
BT549, T47D, MDA-MB-468
and HS578T cell lines,
respectively, % inhibition = 41
of tubulin polymerization at 10 µM

Fig. S6 (continued). Antiproliferation (µM ± SD) and % inhibitory properties of tubulin polymerization at 10 µM of the synthesized 3-amidoindoles **80** and CA-4 (control drug).

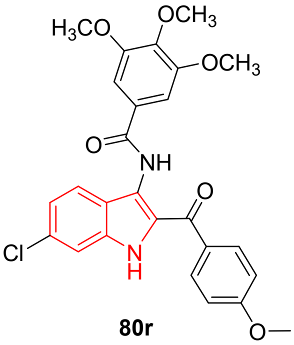

80r

IC_{50} = 17.62 ± 0.40, 9.17 ± 1.01, 11.14 ± 0.43, 11.09 ± 1.31, 8.61 ± 0.51, 15.93 ± 1.27 μM against MCF-7, MDA-MB-231, BT549, T47D, MDA-MB-468 and HS578T cell lines, respectively, % inhibition = 47 of tubulin polymerization at 10 μM

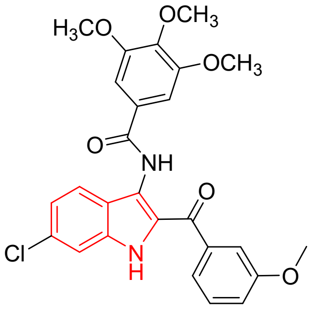

80s

IC_{50} = >100, >100, >100, 52.41 ± 1.06, >100, >100 μM against MCF-7, MDA-MB-231, BT549, T47D, MDA-MB-468 and HS578T cell lines, respectively

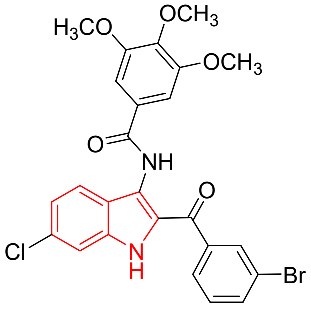

80t

IC_{50} = 23.02 ± 2.47, 26.75 ± 1.12, 8.42 ± 1.06, 14.07 ± 0.61, 20.79 ± 0.34, 4.10 ± 0.27 μM against MCF-7, MDA-MB-231, BT549, T47D, MDA-MB-468 and HS578T cell lines, respectively, % inhibition = 25 of tubulin polymerization at 10 μM

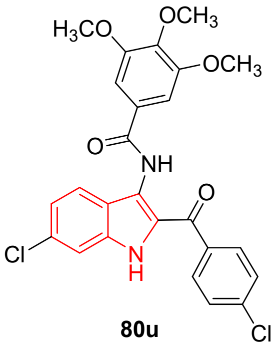

80u

IC_{50} = 10.87 ± 0.87, 6.43 ± 1.12, 3.17 ± 0.46, 0.04 ± 0.06, 7.92 ± 1.36, >100 μM against MCF-7, MDA-MB-231, BT549, T47D, MDA-MB-468 and HS578T cell lines, respectively, % inhibition = 59 of tubulin polymerization at 10 μM

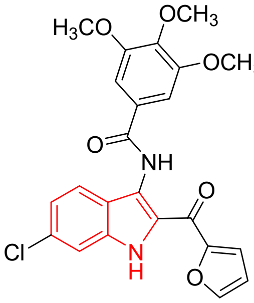

80v

IC_{50} = >100, >100, >100, >100, >100, >100 μM against MCF-7, MDA-MB-231, BT549, T47D, MDA-MB-468 and HS578T cell lines, respectively

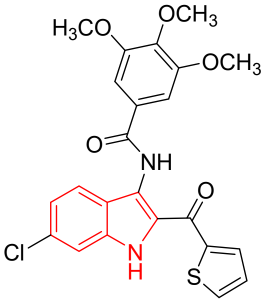

80w

IC_{50} = 47.46 ± 3.29, >100, 37.51 ± 7.14, 31.45 ± 1.87, 15.33 ± 3.11, 49.23 ± 4.6 μM against MCF-7, MDA-MB-231, BT549, T47D, MDA-MB-468 and HS578T cell lines, respectively

Fig. S6 (continued). Antiproliferation (μM ± SD) and % inhibitory properties of tubulin polymerization at 10 μM of the synthesized 3-amidoindoles **80** and CA-4 (control drug).

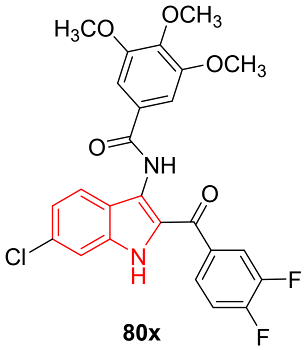

80x

IC_{50} = 10.14 ± 1.16, >100, 10.14 ± 1.16, >100, >100, 64.57 ± 5.73 µM against MCF-7, MDA-MB-231, BT549, T47D, MDA-MB-468 and HS578T cell lines, respectively

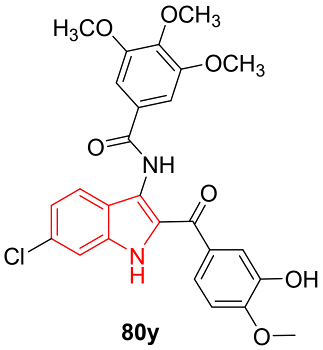

80y

IC_{50} = 32.60 ± 0.68, 22.18 ± 1.38, 7.48 ± 0.19, 9.28 ± 0.13, 23.21 ± 2.08, 4.35 ± 0.14 µM against MCF-7, MDA-MB-231, BT549, T47D, MDA-MB-468 and HS578T cell lines, respectively, % inhibition = 30 of tubulin polymerization at 10 µM

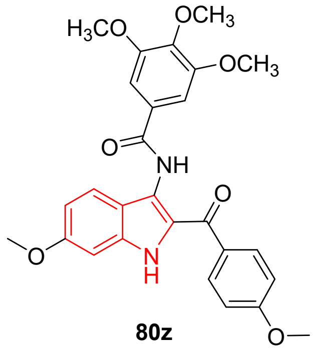

80z

IC_{50} = 79.27 ± 3.05, 48.84 ± 2.76, 18.20 ± 0.13, 6.30 ± 0.52, 26.38 ± 1.36, 10.44 ± 0.66 µM against MCF-7, MDA-MB-231, BT549, T47D, MDA-MB-468 and HS578T cell lines, respectively, % inhibition = 25 of tubulin polymerization at 10 µM

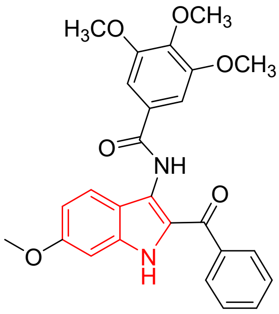

80aa

IC_{50} = >100, >100, >100, >100, >100, >100 µM against MCF-7, MDA-MB-231, BT549, T47D, MDA-MB-468 and HS578T cell lines, respectively

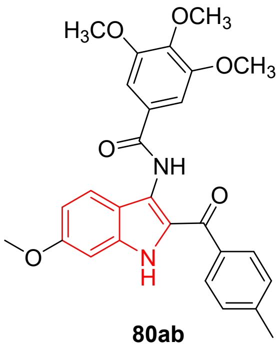

80ab

IC_{50} = >100, >100, >100, >100, >100, >100 µM against MCF-7, MDA-MB-231, BT549, T47D, MDA-MB-468 and HS578T cell lines, respectively

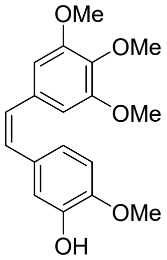

CA-4

IC_{50} = 3.00 ± 0.65, 3.17 ± 0.57, 1.71 ± 0.44, 1.89 ± 0.34, 1.55 ± 0.18, 1.36 ± 0.23 µM against MCF-7, MDA-MB-231, BT549, T47D, MDA-MB-468 and HS578T cell lines, respectively, % inhibition = 80 of tubulin polymerization at 10 µM

Fig. S6 (continued). Antiproliferation (µM ± SD) and % inhibitory properties of tubulin polymerization at 10 µM of the synthesized 3-amidoindoles **80** and CA-4 (control drug).

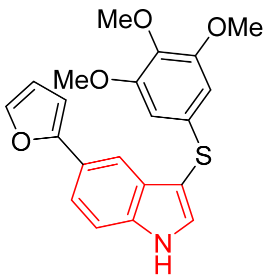

83a

IC_{50} = 70 ± 10 μM against MCF-7, IC_{50} = 0.87 ± 0.1 μM tubulin polymerization inhibition

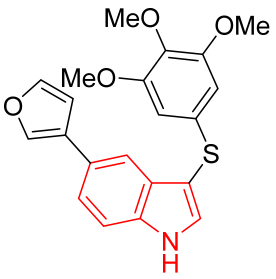

83b

IC_{50} = 200 ± 100 μM against MCF-7, IC_{50} = 1.8 ± 0.1 μM tubulin polymerization inhibition

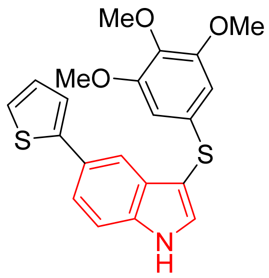

83c

IC_{50} = 150 ± 70 μM against MCF-7, IC_{50} = 1.3 ± 0.09 μM tubulin polymerization inhibition

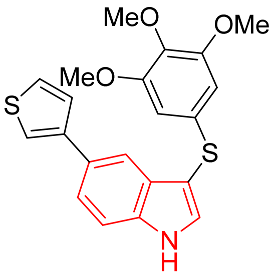

83d

IC_{50} = 200 ± 0 μM against MCF-7, IC_{50} = 1.7 ± 0.03 μM tubulin polymerization inhibition

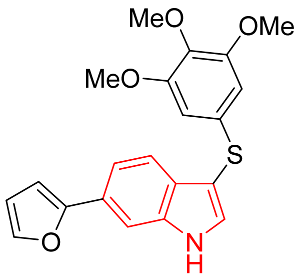

83e

IC_{50} = 10 ± 3 μM against MCF-7, IC_{50} = 0.59 ± 1 μM tubulin polymerization inhibition

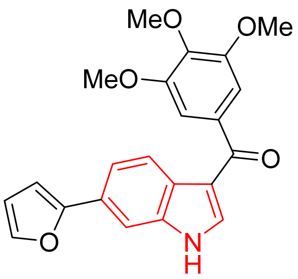

85a

IC_{50} = 4.7 ± 0.6 μM against MCF-7, IC_{50} = 0.76 ± 0.04 μM tubulin polymerization inhibition

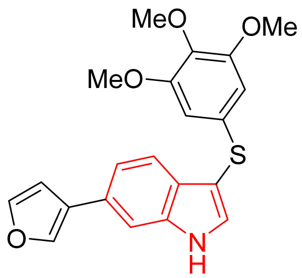

83f

IC_{50} = 10 ± 0 μM against MCF-7, IC_{50} = 2.3 ± 0.04 μM tubulin polymerization inhibition

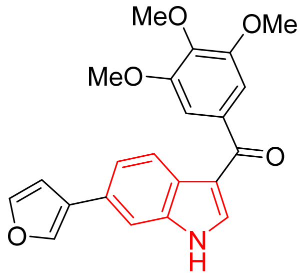

85b

IC_{50} = 4.3 ± 0.6 μM against MCF-7, IC_{50} = 0.61 ± 0.06 μM tubulin polymerization inhibition

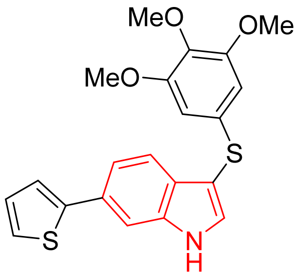

83g

IC_{50} = 6.0 ± 1 μM against MCF-7, IC_{50} = 0.47 ± 0.05 μM tubulin polymerization inhibition

Fig. S7. Antiproliferation and tubulin polymerization inhibitory properties (μM ± SD) of the synthesized 3-arylthio-1*H*-indoles **83**, 3-aroyl-1*H*-indoles **85** and standard drugs (CA-4 and Colchicine).

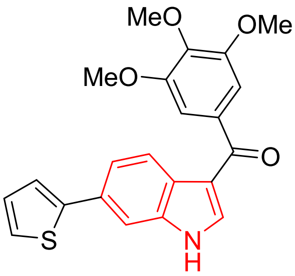

85c

IC_{50} = 9.6 ± 0.6 μM against MCF-7, IC_{50} = 0.38 ± 0.1 μM tubulin polymerization inhibition

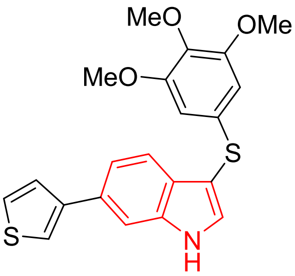

83h

IC_{50} = 4.5 ± 1 μM against MCF-7, IC_{50} = 0.58 ± 0.06 μM tubulin polymerization inhibition

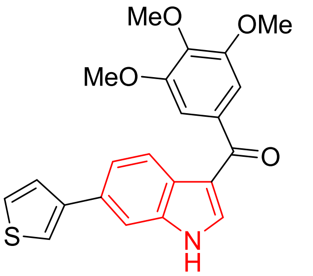

85d

IC_{50} = 18 ± 4 μM against MCF-7, IC_{50} = 0.60 ± 0.04 μM tubulin polymerization inhibition

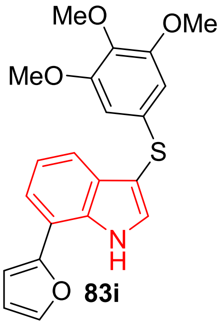

83i

IC_{50} = 15 ± 4 μM against MCF-7, IC_{50} = 0.64 ± 0.06 μM tubulin polymerization inhibition

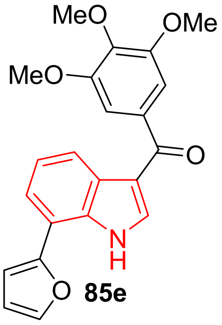

85e

IC_{50} = 320 ± 10 μM against MCF-7, IC_{50} = 1.9 ± 0.03 μM tubulin polymerization inhibition

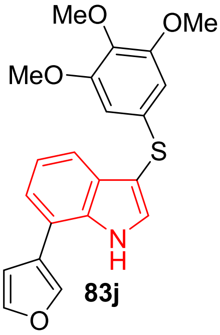

83j

IC_{50} = 24 ± 2 μM against MCF-7, IC_{50} = 1.3 ± 0.1 μM tubulin polymerization inhibition

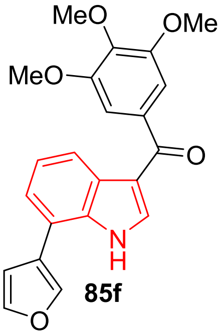

85f

IC_{50} = 1300 ± 0 μM against MCF-7, IC_{50} = 3.0 ± 0.3 μM tubulin polymerization inhibition

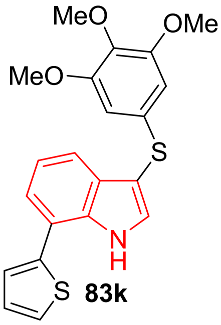

83k

IC_{50} = 29 ± 1 μM against MCF-7, IC_{50} = 0.57 ± 0.03 μM tubulin polymerization inhibition

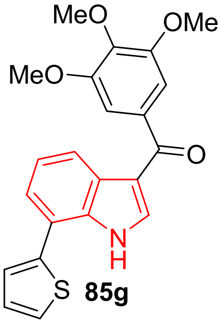

85g

IC_{50} = 550 ± 70 μM against MCF-7, IC_{50} = 2.6 ± 0.04 μM tubulin polymerization inhibition

Fig. S7 (continued). Antiproliferation and tubulin polymerization inhibitory properties (μM ± SD) of the synthesized 3-arylthio-1*H*-indoles **83**, 3-aroyl-1*H*-indoles **85** and standard drugs (CA-4 and Colchicine**).**

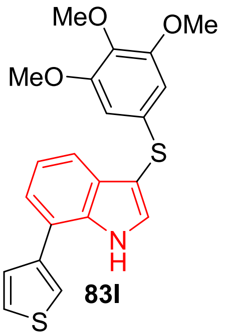

83l

IC_{50} = 38 ± 10 μM against MCF-7, IC_{50} = 1.3 ± 0.01 μM tubulin polymerization inhibition

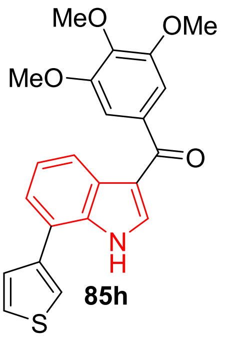

85h

IC_{50} = 1400 ± 100 μM against MCF-7, IC_{50} = 6.2 ± 1 μM tubulin polymerization inhibition

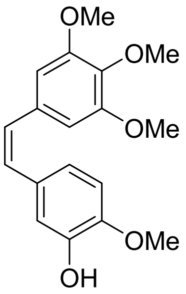

CA-4

IC_{50} = 13 ± 3 μM against MCF-7, IC_{50} = 1.0 ± 0.1 μM tubulin polymerization inhibition

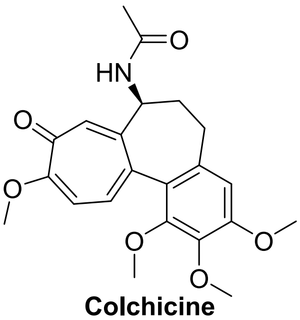

Colchicine

IC_{50} = 5 ± 1 μM against MCF-7, IC_{50} = 3.2 ± 0.4 μM tubulin polymerization inhibition

Fig. S7 (continued). Antiproliferation and tubulin polymerization inhibitory properties (μM ± SD) of the synthesized 3-arylthio-1*H*-indoles **83**, 3-aroyl-1*H*-indoles **85** and standard drugs (CA-4 and Colchicine).

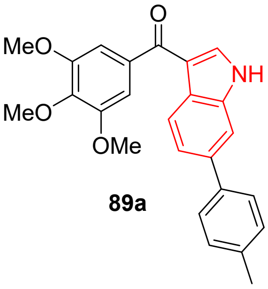

89a

GI_{50} = 560 ± 60, 2010 ± 433 (nM) against MCF-7, MDA-MB-231, respectively; IC_{50} = 1.7 ± 0.08 μM of tubulin polymerization inhibition

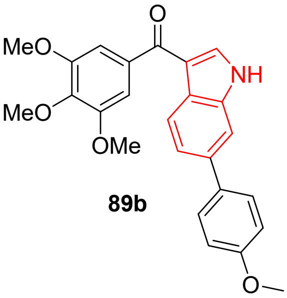

89b

GI_{50} = 98 ± 30, 1860 ± 285 (nM) against MCF-7, MDA-MB-231, respectively; IC_{50} = 2.5 ± 0.06 μM of tubulin polymerization inhibition

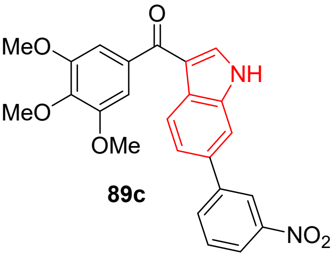

89c

GI_{50} = 28 ± 10, 110 ± 15 (nM) against MCF-7, MDA-MB-231, respectively; IC_{50} = 0.65 ± 0.06 μM of tubulin polymerization inhibition

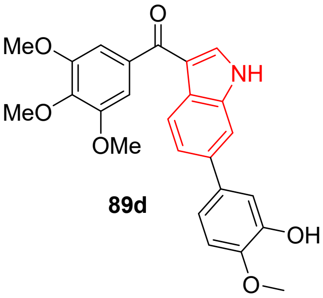

89d

GI_{50} = 1500 ± 100, 828 ± 155 (nM) against MCF-7, MDA-MB-231, respectively; IC_{50} = >20 μM of tubulin polymerization inhibition

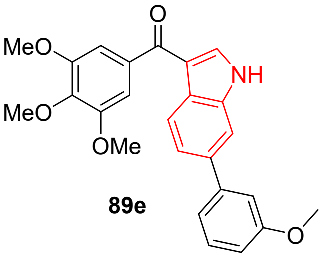

89e

GI_{50} = 23 ± 7, 36 ± 4 (nM) against MCF-7, MDA-MB-231, respectively; IC_{50} = 0.37 ± 0.04 μM of tubulin polymerization inhibition

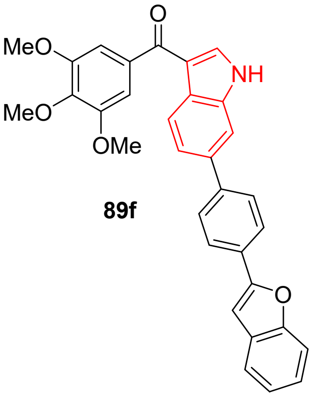

89f

GI_{50} = 950 ± 200, 2066 ± 302 (nM) against MCF-7, MDA-MB-231, respectively; IC_{50} = >20 μM of tubulin polymerization inhibition

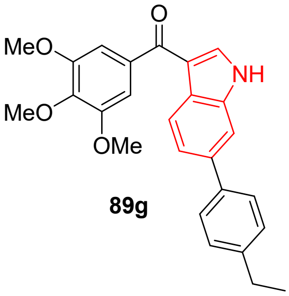

89g

GI_{50} = 1800 ± 500, 2220 ± 180 (nM) against MCF-7, MDA-MB-231, respectively; IC_{50} = >20 μM of tubulin polymerization inhibition

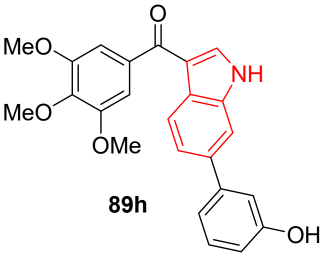

89h

GI_{50} = 320 ± 20, 102 ± 14 (nM) against MCF-7, MDA-MB-231, respectively; IC_{50} = 0.57 ± 0.02 μM of tubulin polymerization inhibition

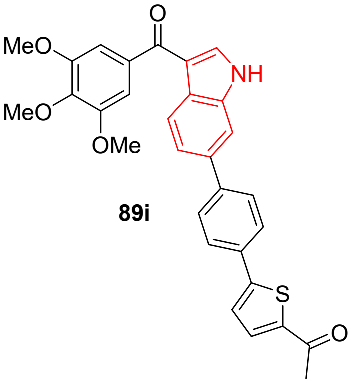

89i

GI_{50} = 73 ± 4, 3231 ± 482 (nM) against MCF-7, MDA-MB-231, respectively; IC_{50} = >20 μM of tubulin polymerization inhibition

Fig. S8. Antiprolifeation (nM ± SD) and tubulin polymerization inhibitory (μM ± SD) properties of 6-ary-3-aroyllindoles **89** and CA-4 (standard reference).

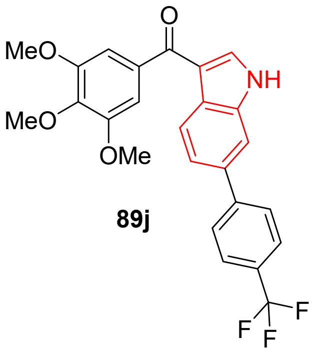

89j

GI_{50} = 1400 ± 100, 4062 ± 822
(nM) against MCF-7,
MDA-MB-231, respectively;
IC_{50} = >20 μM of
tubulin polymerization inhibition

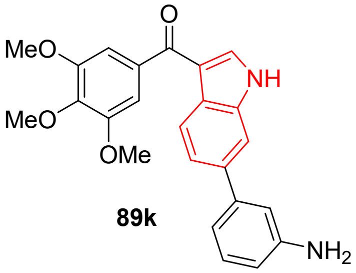

89k

GI_{50} = 65 ± 7, 25 ± 5
(nM) against MCF-7,
MDA-MB-231, respectively;
IC_{50} = 0.42 ± 0.08 μM of
tubulin polymerization inhibition

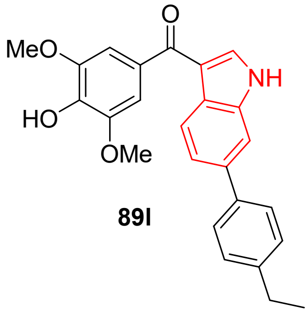

89l

GI_{50} = >5000, >5000
(nM) against MCF-7,
MDA-MB-231, respectively;
IC_{50} = >20 μM of
tubulin polymerization inhibition

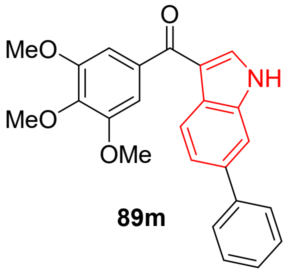

89m

GI_{50} = 73 ± 10, 156 ± 20
(nM) against MCF-7,
MDA-MB-231, respectively;
IC_{50} = 0.28 ± 0.04 μM of
tubulin polymerization inhibition

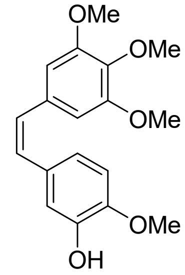

CA-4

GI_{50} = 9.0 ± 2, 3.5 ± 0.4
(nM) against MCF-7,
MDA-MB-231, respectively;
IC_{50} = 0.75 ± 0.06 μM of
tubulin polymerization inhibition

Fig. S8 (continued). Antiproliferation (nM ± SD) and tubulin polymerization inhibitory (μM ± SD) properties of 6-ary-3-aroyllindoles **89** and CA-4 (standard reference).

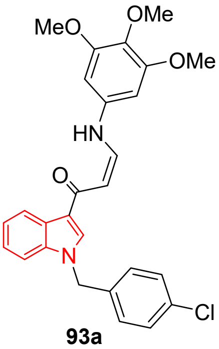

93a

IC_{50} = 2.3 ± 0.2, 9.3 ± 0.3, 7.2 ± 0.4, 8.7 ± 0.4 μM against MCF-7, HeLa, A549, and DU-145 cell ines, respectively

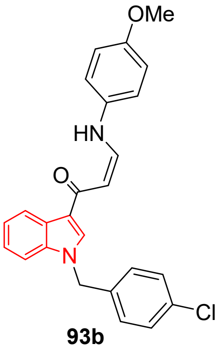

93b

IC_{50} = 7.3 ± 0.4, 8.9 ± 0.3, 11.2 ± 0.3, 18.3 ± 0.6 μM against MCF-7, HeLa, A549, and DU-145 cell ines, respectively

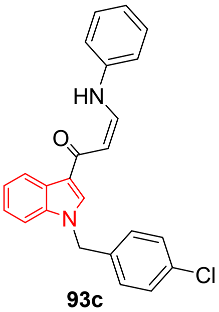

93c

IC_{50} = 36.8 ± 0.4, 42.6 ± 0.9, 32.0 ± 0.4, 42.7 ± 0.7 μM against MCF-7, HeLa, A549, and DU-145 cell ines, respectively

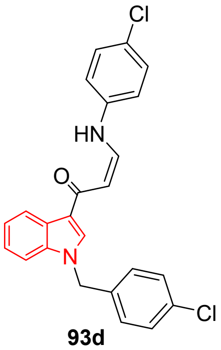

93d

IC_{50} = 30.2 ± 0.2, 39.7 ± 0.7, 41.2 ± 0.3, 29.1 ± 0.2 μM against MCF-7, HeLa, A549, and DU-145 cell ines, respectively

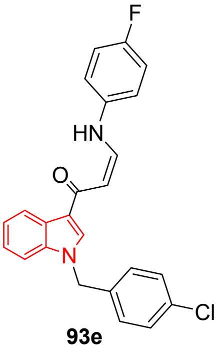

93e

IC_{50} = 20.9 ± 0.3, 18.9 ± 0.6, 20.2 ± 0.5, 47.2 ± 0.5 μM against MCF-7, HeLa, A549, and DU-145 cell ines, respectively

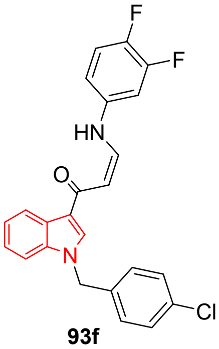

93f

IC_{50} = 31.9 ± 0.1, 28.0 ± 0.4, 42.8 ± 0.8, >50 μM against MCF-7, HeLa, A549, and DU-145 cell ines, respectively

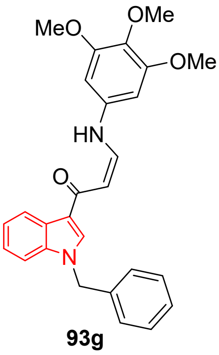

93g

IC_{50} = 1.9 ± 0.1, 4.1 ± 0.4, 4.7 ± 0.3, 7.5 ± 0.4 μM against MCF-7, HeLa, A549, and DU-145 cell ines, respectively

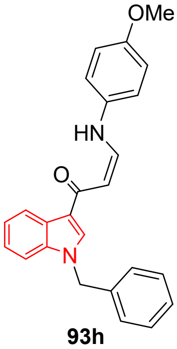

93h

IC_{50} = 5.7 ± 0.1, 6.1 ± 0.2, 9.6 ± 0.4, 13.6 ± 0.6 μM against MCF-7, HeLa, A549, and DU-145 cell ines, respectively

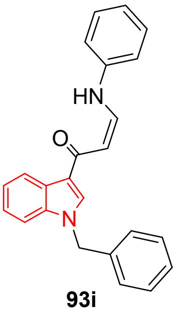

93i

IC_{50} = 32.0 ± 0.4, 28.6 ± 0.5, 23.8 ± 0.7, 17.3 ± 0.3 μM against MCF-7, HeLa, A549, and DU-145 cell ines, respectively

Fig. S9. Antiproliferation properties (μM ± SD) of the synthesized indolyl-arylaminopropenone conjugates **93** and reference drug (Doxorubicin).

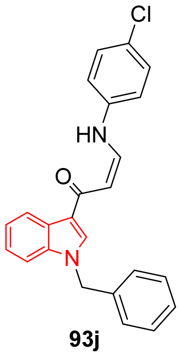

93j

IC_{50} = 18.3 ± 0.1, 28.1 ± 0.7, 35.2 ± 0.3, 30.3 ± 0.3 μM against MCF-7, HeLa, A549, and DU-145 cell ines, respectively

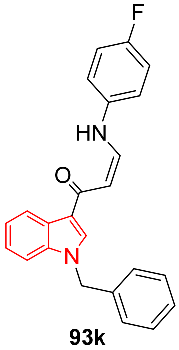

93k

IC_{50} = 22.7 ± 0.3, 39.4 ± 0.3, 36.2 ± 0.5, 20.0 ± 0.2 μM against MCF-7, HeLa, A549, and DU-145 cell ines, respectively

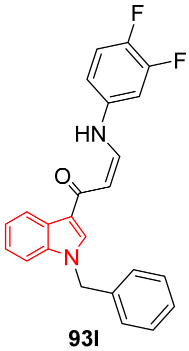

93l

IC_{50} = 15.6 ± 0.4, 26.9 ± 0.2, 23.5 ± 0.2, 32.7 ± 0.8 μM against MCF-7, HeLa, A549, and DU-145 cell ines, respectively

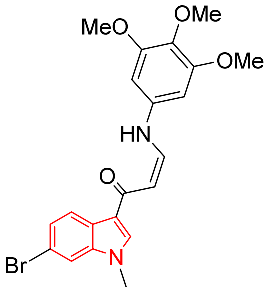

93m

IC_{50} = 15.3 ± 0.6, 12.5 ± 0.3, 21.6 ± 0.9, 14.6 ± 0.5 μM against MCF-7, HeLa, A549, and DU-145 cell ines, respectively

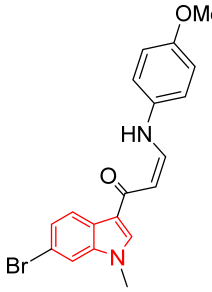

93n

IC_{50} = 18.2 ± 0.6, 19.2 ± 0.1, 15.5 ± 0.4, 27.8 ± 0.4 μM against MCF-7, HeLa, A549, and DU-145 cell ines, respectively

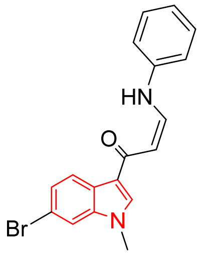

93o

IC_{50} = 32.4 ± 0.5, 21.2 ± 0.4, 41.6 ± 0.9, 30.2 ± 0.5 μM against MCF-7, HeLa, A549, and DU-145 cell ines, respectively

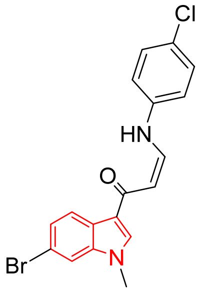

93p

IC_{50} = 39.8 ± 0.8, 33.5 ± 0.3, >50, 43.9 ± 0.6 μM against MCF-7, HeLa, A549, and DU-145 cell ines, respectively

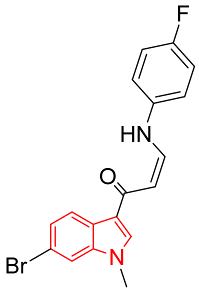

93q

IC_{50} = >50, 24.8 ± 0.2, >50, >50 μM against MCF-7, HeLa, A549, and DU-145 cell ines, respectively

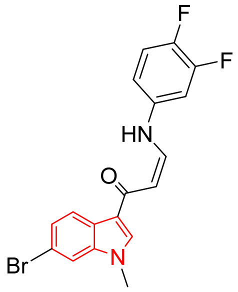

93r

IC_{50} = 36.2 ± 0.6, 44.2 ± 0.9, 28.7 ± 0.4, >50 μM against MCF-7, HeLa, A549, and DU-145 cell ines, respectively

Fig. S9 (continued). Antiproliferation properties (μM ± SD) of the synthesized indolyl-arylaminopropenone conjugates **93** and reference drug (Doxorubicin).

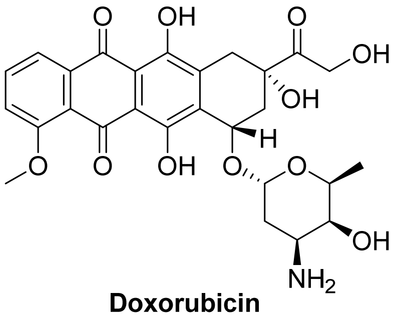

Doxorubicin

IC_{50} = 0.8 ± 0.2, 1.6 ± 0.1,
2.0 ± 0.1, 0.9 ± 0.1 μM
against MCF-7, HeLa,
A549, and DU-145 cell ines,
respectively

Fig. S9 (continued). Antiproliferation properties (μM ± SD) of the synthesized indolyl-arylaminopropenone conjugates **93** and reference drug (Doxorubicin).

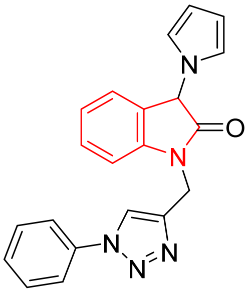

104a

IC_{50} = >50, >50, ND
µM against MCF-7,
MDA-MB-231 and HEK-293
cell lines, respectively

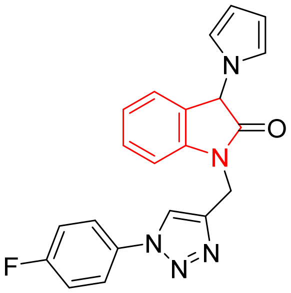

104b

IC_{50} = >50, >50, ND
µM against MCF-7,
MDA-MB-231 and HEK-293
cell lines, respectively

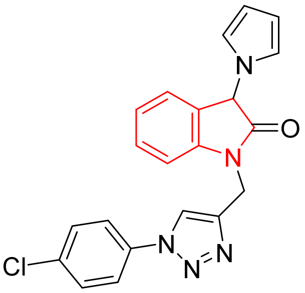

104c

IC_{50} = >50, >50, ND
µM against MCF-7,
MDA-MB-231 and HEK-293
cell lines, respectively

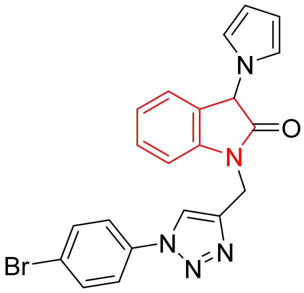

104d

IC_{50} = >50, 18.9 $\pm$ 0.01, ND
µM against MCF-7,
MDA-MB-231 and HEK-293
cell lines, respectively

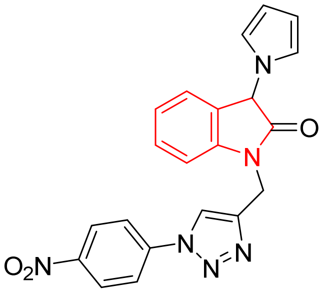

104e

IC_{50} = >50, >50, ND
µM against MCF-7,
MDA-MB-231 and HEK-293
cell lines, respectively

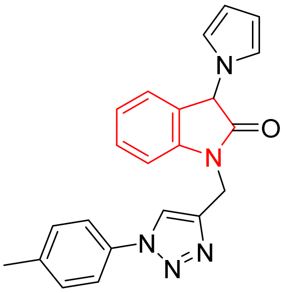

104f

IC_{50} = >50, >50, ND
µM against MCF-7,
MDA-MB-231 and HEK-293
cell lines, respectively

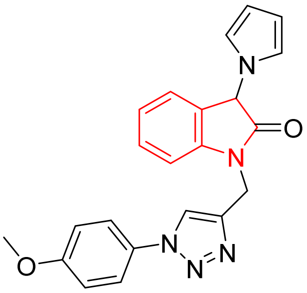

104g

IC_{50} = >50, >50, ND
µM against MCF-7,
MDA-MB-231 and HEK-293
cell lines, respectively

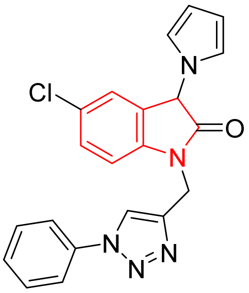

104h

IC_{50} = >50, 6.8 $\pm$ 0.01, >50
µM against MCF-7,
MDA-MB-231 and HEK-293
cell lines, respectively

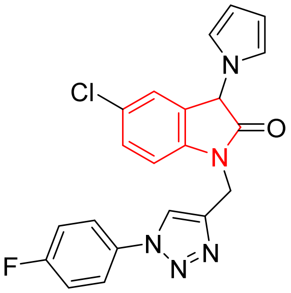

104i

IC_{50} = >50, 35 $\pm$ 0.04, ND
µM against MCF-7,
MDA-MB-231 and HEK-293
cell lines, respectively

Fig. S10. Antiproliferation properties (µM $\pm$ SD) of the synthesized 3-pyrrolylisatin-triazole conjugates **104** and Tamoxifen citrate "reference drug" (ND = not determined).

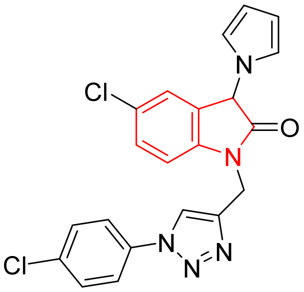

104j

IC_{50} = >50, >50, ND
μM against MCF-7,
MDA-MB-231 and HEK-293
cell lines, respectively

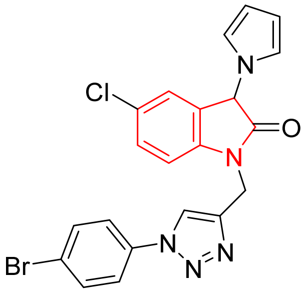

104k

IC_{50} = >50, >50, ND
μM against MCF-7,
MDA-MB-231 and HEK-293
cell lines, respectively

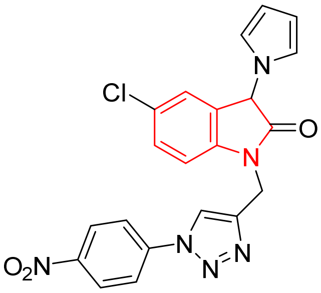

104l

IC_{50} = 30.1 ± 0.02, 17.7 ± 0.01,
ND μM against MCF-7,
MDA-MB-231 and HEK-293
cell lines, respectively

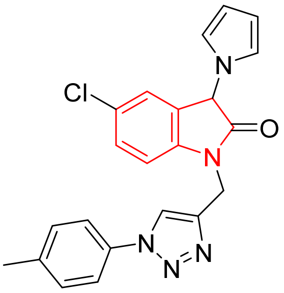

104m

IC_{50} = >50, 20 ± 0.04, ND
μM against MCF-7,
MDA-MB-231 and HEK-293
cell lines, respectively

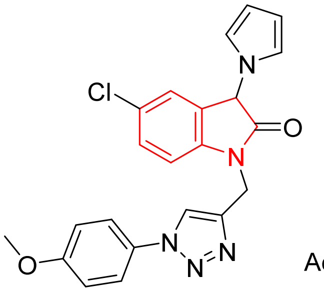

104n

IC_{50} = >50, >50, ND
μM against MCF-7,
MDA-MB-231 and HEK-293
cell lines, respectively

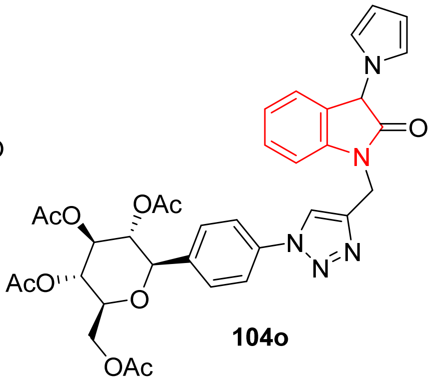

104o

IC_{50} = >50, >50, ND
μM against MCF-7,
MDA-MB-231 and HEK-293
cell lines, respectively

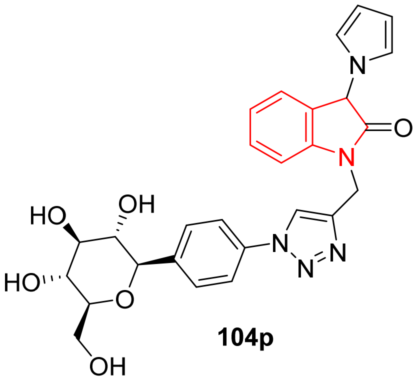

104p

IC_{50} = >50, >50, ND
μM against MCF-7,
MDA-MB-231 and HEK-293
cell lines, respectively

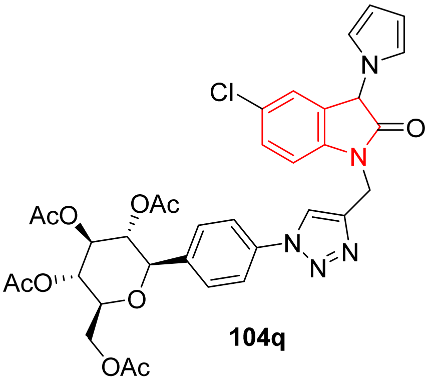

104q

IC_{50} = >50, 6 ± 0.04, >50
μM against MCF-7,
MDA-MB-231 and HEK-293
cell lines, respectively

Fig. S10 (continued). Antiproliferation properties (μM ± SD) of the synthesized 3-pyrrolylisatin-triazole conjugates **104** and Tamoxifen citrate "reference drug" (ND = not determined).

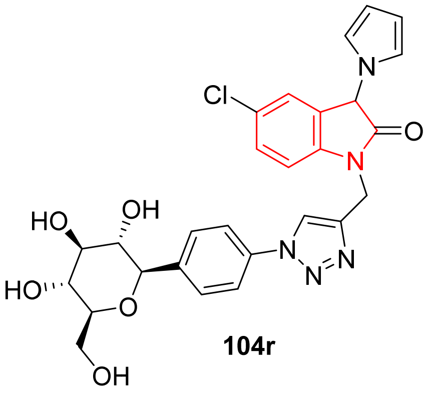

104r

IC_{50} = >50, 5.9 ± 0.02, >50 μM against MCF-7, MDA-MB-231 and HEK-293 cell lines, respectively

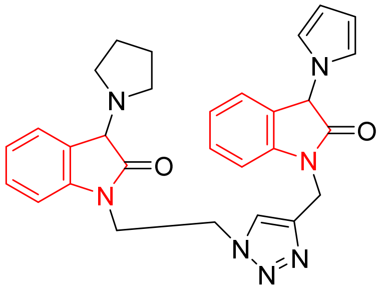

104s

IC_{50} = >50, 7.5 ± 0.02, >50 μM against MCF-7, MDA-MB-231 and HEK-293 cell lines, respectively

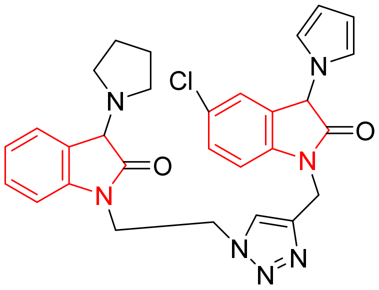

104t

IC_{50} = >50, 25 ± 0.5, ND μM against MCF-7, MDA-MB-231 and HEK-293 cell lines, respectively

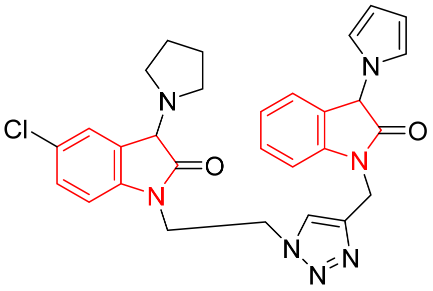

104u

IC_{50} = 44.3 ± 0.03, 6.5 ± 0.02, >50 μM against MCF-7, MDA-MB-231 and HEK-293 cell lines, respectively

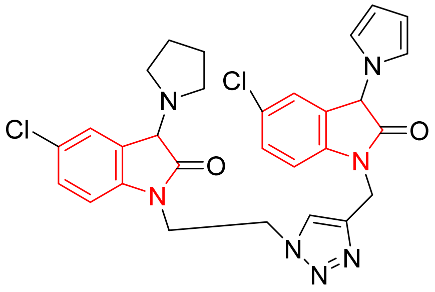

104v

IC_{50} = >50, 6.2 ± 0.01, >50 μM against MCF-7, MDA-MB-231 and HEK-293 cell lines, respectively

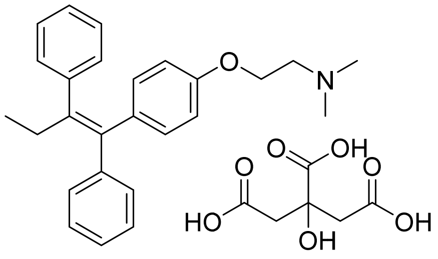

Tamoxifen citrate

IC_{50} = 12 ± 0.02, 18.9 ± 0.01, 24 ± 0.03 μM against MCF-7, MDA-MB-231 and HEK-293 cell lines, respectively

Fig. S10 (continued). Antiproliferation properties (μM ± SD) of the synthesized 3-pyrrolylisatin-triazole conjugates **104** and Tamoxifen citrate "reference drug" (ND = not determined).

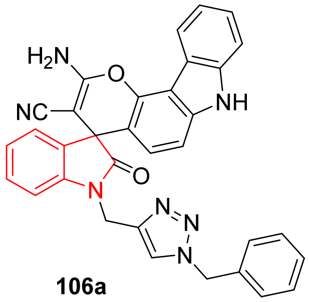

106a

IC_{50} = 7.02 ± 0.93, 17.25 ± 0.08, 12.35 ± 0.17, >100 μM against MCF-7, MDA-MB-231, HeLa and HUVEC cell lines, respectively

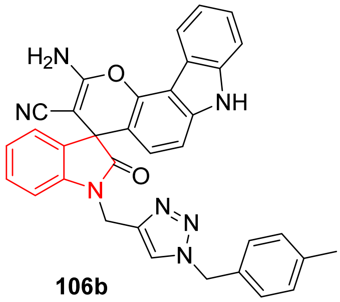

106b

IC_{50} = 25.56 ± 2.12, 12.74 ± 2.32, 4.05 ± 0.69, >100 μM against MCF-7, MDA-MB-231, HeLa and HUVEC cell lines, respectively

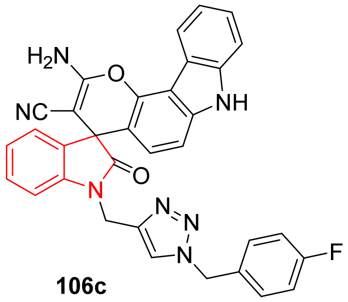

106c

IC_{50} = 14.37 ± 0.95, 13.33 ± 0.48, >100, >100 μM against MCF-7, MDA-MB-231, HeLa and HUVEC cell lines, respectively

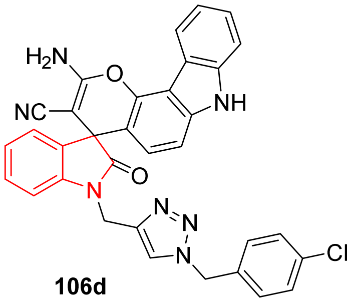

106d

IC_{50} = >100, >100, >100, >100 μM against MCF-7, MDA-MB-231, HeLa and HUVEC cell lines, respectively

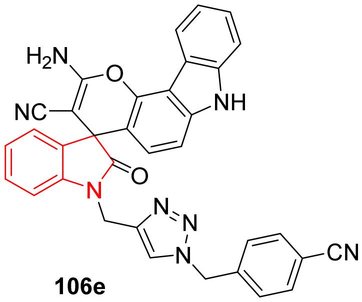

106e

IC_{50} = 25.51 ± 0.32, 37.66 ± 0.55, >100, >100 μM against MCF-7, MDA-MB-231, HeLa and HUVEC cell lines, respectively

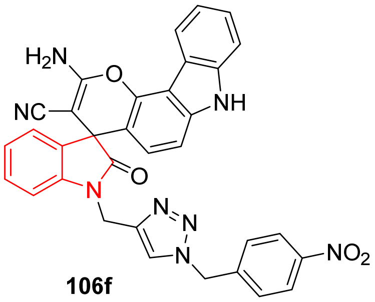

106f

IC_{50} = 2.13 ± 0.16, 24.89 ± 0.74, >100 ± 1.6, >100 μM against MCF-7, MDA-MB-231, HeLa and HUVEC cell lines, respectively

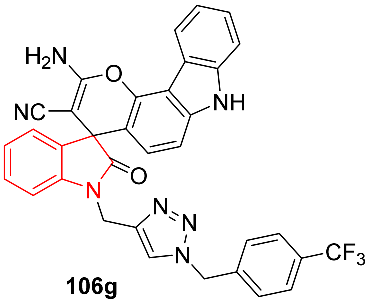

106g

IC_{50} = 6.31 ± 0.63, 14.86 ± 0.12, 3.54 ± 1.24, >100 μM against MCF-7, MDA-MB-231, HeLa and HUVEC cell lines, respectively

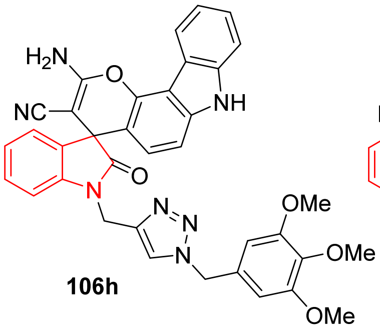

106h

IC_{50} = 6.97 ± 0.83, 23.66 ± 1.02, 15.88 ± 1.12, >100 μM against MCF-7, MDA-MB-231, HeLa and HUVEC cell lines, respectively

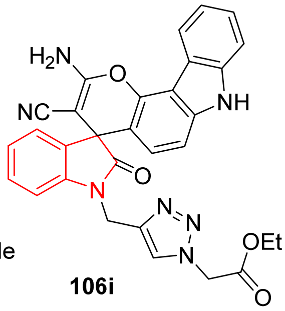

106i

IC_{50} = 18.00 ± 0.32, 41.64 ± 0.04, 10.83 ± 0.6, >100 μM against MCF-7, MDA-MB-231, HeLa and HUVEC cell lines, respectively

Fig. S11. Antiproliferation properties (μM ± SD) of spirochromenocarbazols linked to 1,2,3-triazole **106**, and standard references (Paclitaxel and Doxorubicin).

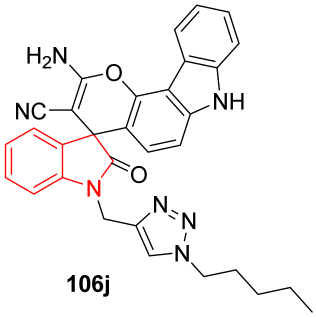

106j

IC_{50} = 4.80 ± 0.25, 8.39 ± 1.84, 9.21 ± 0.34, >100 µM against MCF-7, MDA-MB-231, HeLa and HUVEC cell lines, respectively

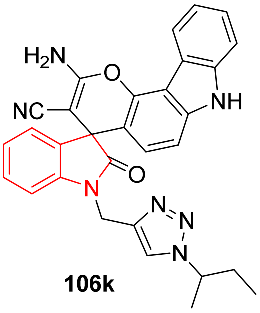

106k

IC_{50} = 51.16 ± 0.53, 3.78 ± 0.74, 61.89 ± 2.53, >100 µM against MCF-7, MDA-MB-231, HeLa and HUVEC cell lines, respectively

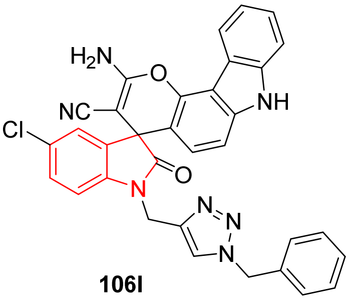

106l

IC_{50} = >100, >100, >100, >100 µM against MCF-7, MDA-MB-231, HeLa and HUVEC cell lines, respectively

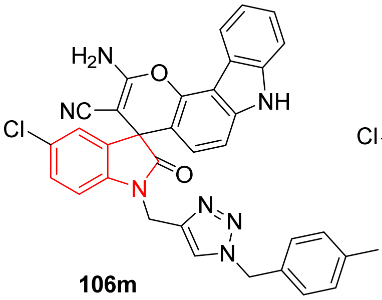

106m

IC_{50} = >100, >100, >100, >100 µM against MCF-7, MDA-MB-231, HeLa and HUVEC cell lines, respectively

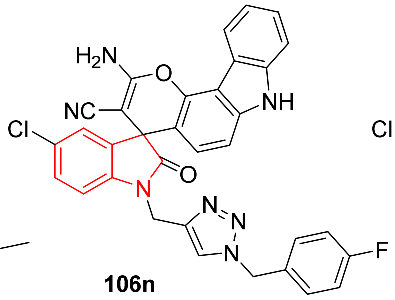

106n

IC_{50} = 62.74 ± 0.17, 71.26 ± 1.18, 75.81 ± 0.61, >100 µM against MCF-7, MDA-MB-231, HeLa and HUVEC cell lines, respectively

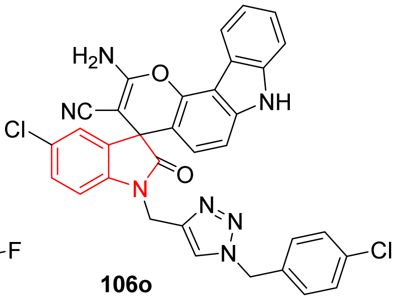

106o

IC_{50} = 42.50 ± 0.67, 60.82 ± 0.38, 13.25 ± 0.37, >100 µM against MCF-7, MDA-MB-231, HeLa and HUVEC cell lines, respectively

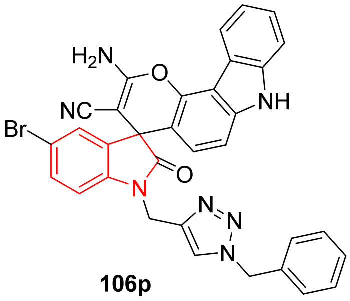

106p

IC_{50} = 9.40 ± 0.36, 42.37 ± 0.55, 32.49 ± 0.15, >100 µM against MCF-7, MDA-MB-231, HeLa and HUVEC cell lines, respectively

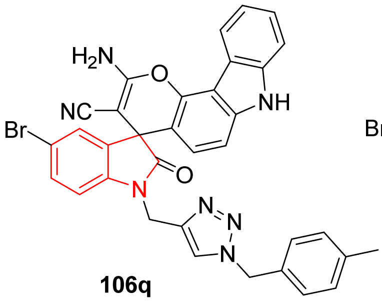

106q

IC_{50} = 7.06 ± 0.35, 30.33 ± 3.8, 61.29 ± 1.67, >100 µM against MCF-7, MDA-MB-231, HeLa and HUVEC cell lines, respectively

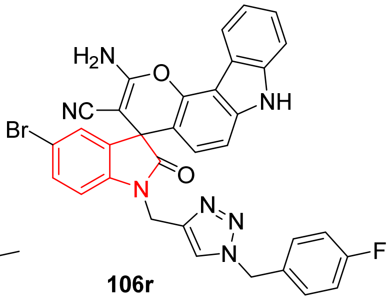

106r

IC_{50} = 6.57 ± 0.26, 25.38 ± 0.69, 42.77 ± 0.22, >100 µM against MCF-7, MDA-MB-231, HeLa and HUVEC cell lines, respectively

Fig. S11 (continued). Antiproliferation properties (µM ± SD) of spirochromenocarbazols linked to 1,2,3-triazole **106**, and standard references (Paclitaxel and Doxorubicin).

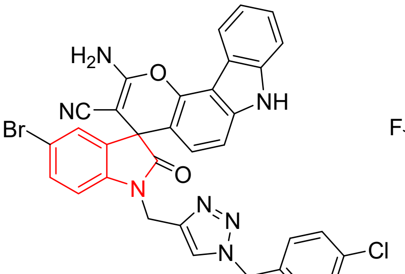

106s

IC_{50} = 14.81 ± 0.61, 9.35 ± 1.10, 3.83 ± 0.51, >100 μM against MCF-7, MDA-MB-231, HeLa and HUVEC cell lines, respectively

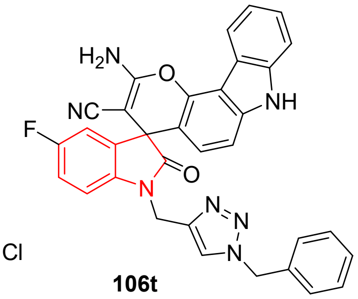

106t

IC_{50} = 9.72 ± 0.89, 99.59 ± 0.55, >100, >100 μM against MCF-7, MDA-MB-231, HeLa and HUVEC cell lines, respectively

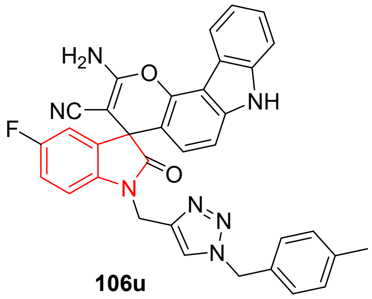

106u

IC_{50} = >100, 75.01 ± 0.28, 3.35 ± 0.17, >100 μM against MCF-7, MDA-MB-231, HeLa and HUVEC cell lines, respectively

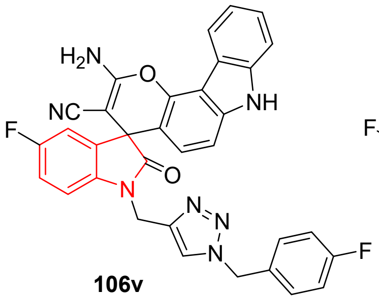

106v

IC_{50} = >100, 63.08 ± 2.14, 37.87 ± 4.11, >100 μM against MCF-7, MDA-MB-231, HeLa and HUVEC cell lines, respectively

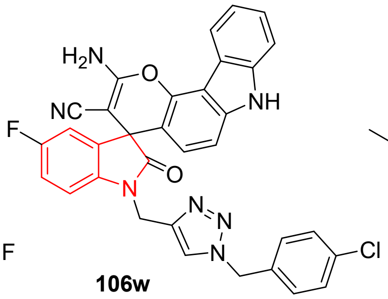

106w

IC_{50} = >100, >100, >100, >100 μM against MCF-7, MDA-MB-231, HeLa and HUVEC cell lines, respectively

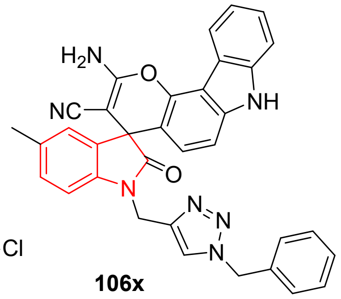

106x

IC_{50} = 88.85 ± 0.06, >100, 59.12 ± 0.32, >100 μM against MCF-7, MDA-MB-231, HeLa and HUVEC cell lines, respectively

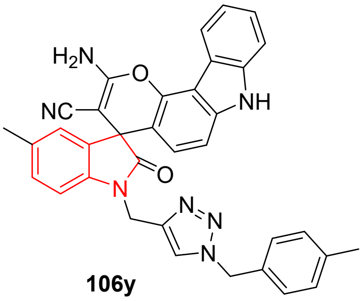

106y

IC_{50} = >100, >100, >100, >100 μM against MCF-7, MDA-MB-231, HeLa and HUVEC cell lines, respectively

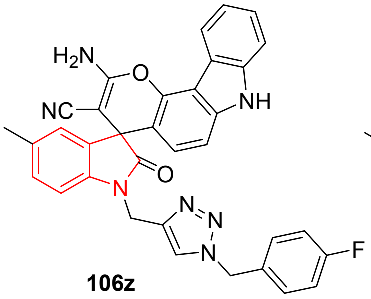

106z

IC_{50} = 16.54 ± 0.51, 27.88 ± 1.67, 7.80 ± 0.58, >100 μM against MCF-7, MDA-MB-231, HeLa and HUVEC cell lines, respectively

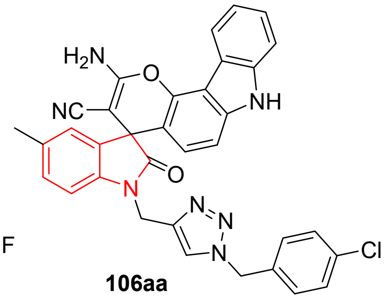

106aa

IC_{50} = 23.69 ± 1.45, 50.32 ± 0.59, 94.77 ± 0.20, >100 μM against MCF-7, MDA-MB-231, HeLa and HUVEC cell lines, respectively

Fig. S11 (continued). Antiproliferation properties (μM ± SD) of spirochromenocarbazols linked to 1,2,3-triazole **106**, and standard references (Paclitaxel and Doxorubicin).

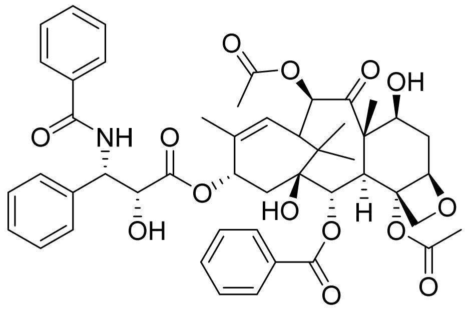

Paclitaxel

IC_{50} = 0.0026 ± 0.00, 0.1 ± 0.07, 0.0061 ± 0.00, >10 μM against MCF-7, MDA-MB-231, HeLa and HUVEC cell lines, respectively

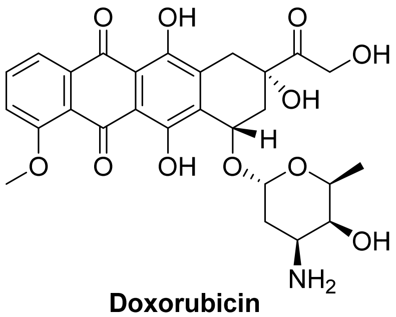

Doxorubicin

IC_{50} = 4.63 ± 0.41, 0. 55 ± 0. 01, 2.66 ± 0.32, >10 μM against MCF-7, MDA-MB-231, HeLa and HUVEC cell lines, respectively

Fig. S11 (continued). Antiproliferation properties (μM ± SD) of spirochromenocarbazols linked to 1,2,3-triazole **106**, and standard references (Paclitaxel and Doxorubicin).

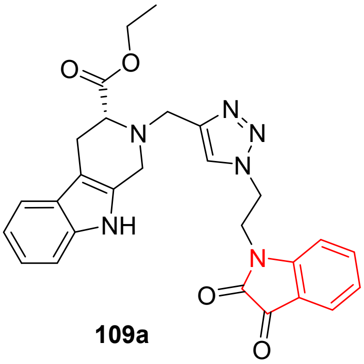

109a

IC_{50} = >100, >100 μM against MCF-7 and MDA-MB231, respectively

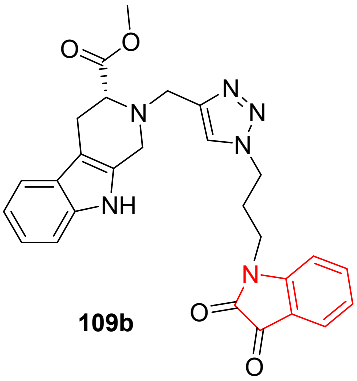

109b

IC_{50} = 37.42, >100 μM against MCF-7 and MDA-MB231, respectively

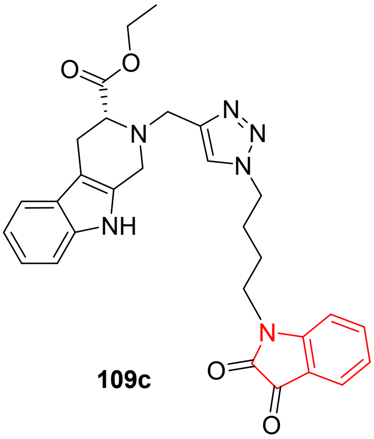

109c

IC_{50} = >100, >100 μM against MCF-7 and MDA-MB231, respectively

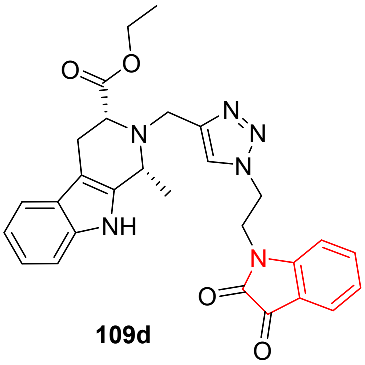

109d

IC_{50} = 45.3, >100 μM against MCF-7 and MDA-MB231, respectively

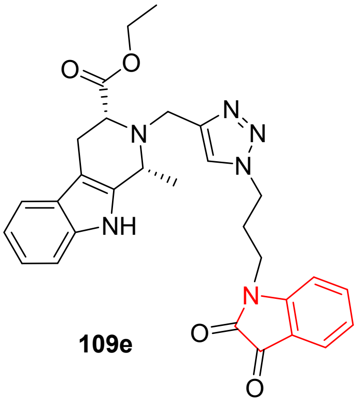

109e

IC_{50} = 50, >100 μM against MCF-7 and MDA-MB231, respectively

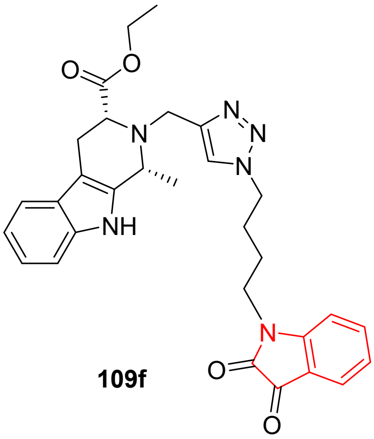

109f

IC_{50} = >100, >100 μM against MCF-7 and MDA-MB231, respectively

Fig. S12. Antiproliferation properties (μM) of tetrahydro-β-carboline and isatin scaffolds connected by 1*H*-1,2,3-triazolyl heterocycle **109** and reference standards (Plumbagin, Peganumine A and Tamoxifen).

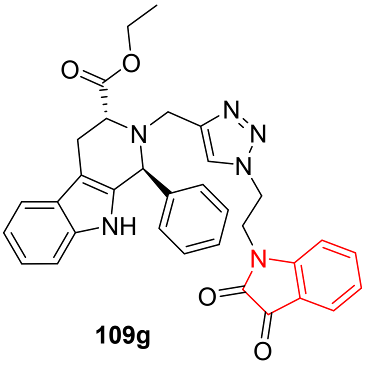

109g

IC$_{50}$ = >100, >100 μM against MCF-7
and MDA-MB231, respectively

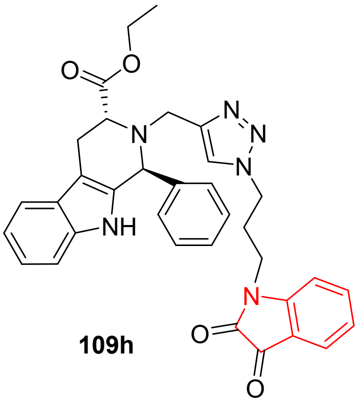

109h

IC$_{50}$ = >100, >100 μM against MCF-7
and MDA-MB231, respectively

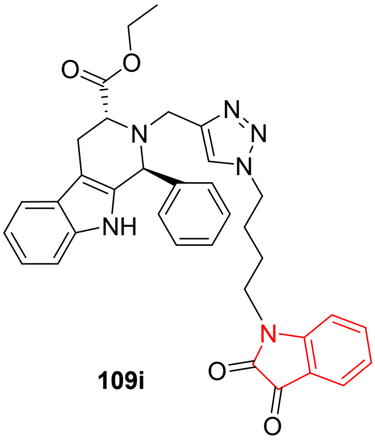

109i

IC$_{50}$ = >100, >100 μM against MCF-7
and MDA-MB231, respectively

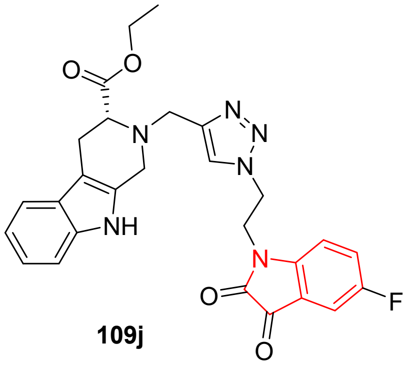

109j

IC$_{50}$ = >100, >100 μM against MCF-7
and MDA-MB231, respectively

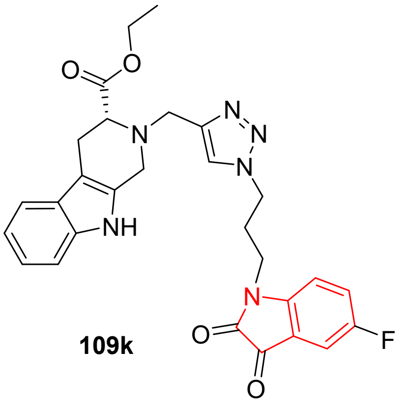

109k

IC$_{50}$ = >100, >100 μM against MCF-7
and MDA-MB231, respectively

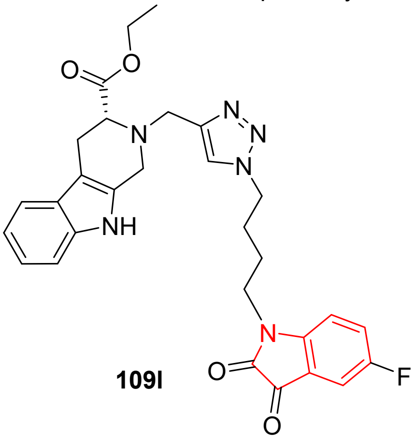

109l

IC$_{50}$ = >100, >100 μM against MCF-7
and MDA-MB231, respectively

Fig. S12 (continued). Antiproliferation properties (μM) of tetrahydro-β-carboline and isatin scaffolds connected by 1*H*-1,2,3-triazolyl heterocycle **109** and reference standards (Plumbagin, Peganumine A and Tamoxifen).

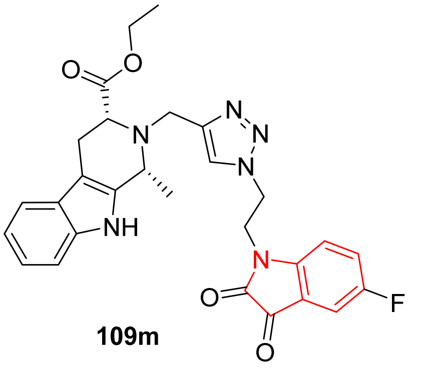

109m

IC$_{50}$ = >100, >100 μM against MCF-7 and MDA-MB231, respectively

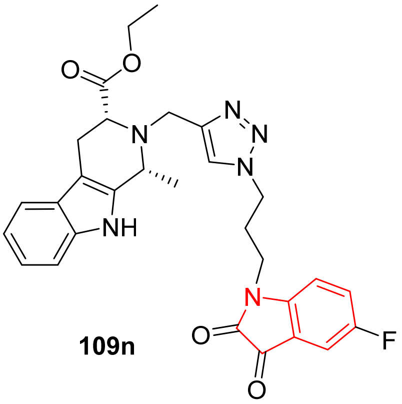

109n

IC$_{50}$ = >100, >100 μM against MCF-7 and MDA-MB231, respectively

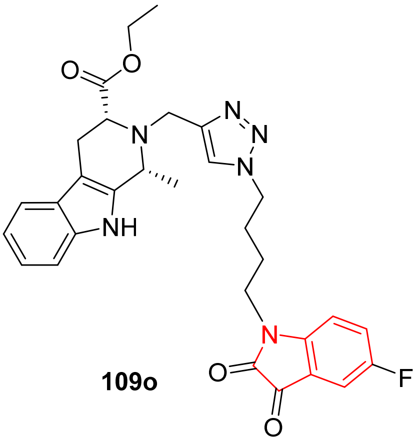

109o

IC$_{50}$ = >100, >100 μM against MCF-7 and MDA-MB231, respectively

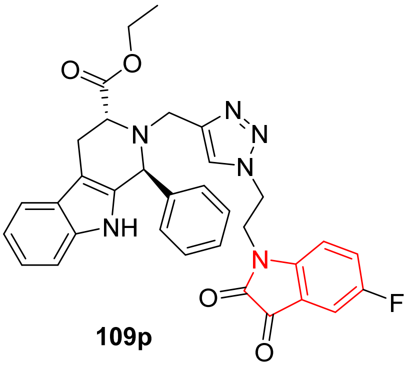

109p

IC$_{50}$ = >100, >100 μM against MCF-7 and MDA-MB231, respectively

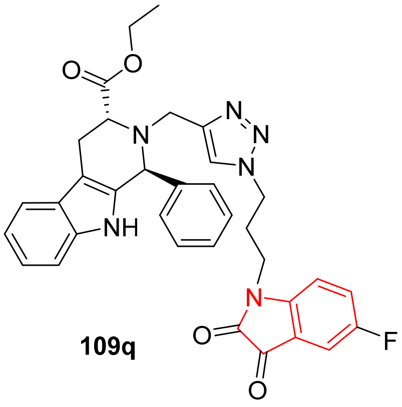

109q

IC$_{50}$ = >100, >100 μM against MCF-7 and MDA-MB231, respectively

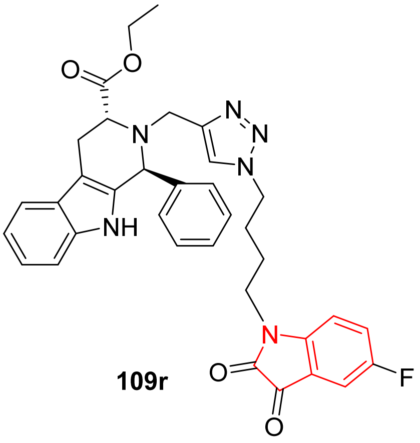

109r

IC$_{50}$ = 42.11, >100 μM against MCF-7 and MDA-MB231, respectively

Fig. S12 (continued). Antiproliferation properties (μM) of tetrahydro-β-carboline and isatin scaffolds connected by 1*H*-1,2,3-triazolyl heterocycle **109** and reference standards (Plumbagin, Peganumine A and Tamoxifen).

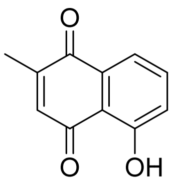

Plumbagin

IC_{50} = 3.5, 4.4 μM
against MCF-7
and MDA-MB231,
respectively

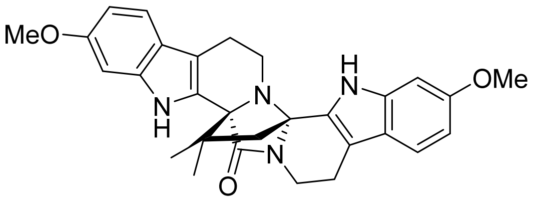

Peganumine A

IC_{50} = 38.5 μM against MCF^{-7}

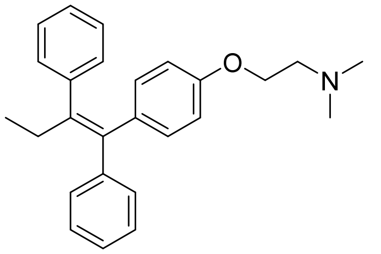

Tamoxifen

IC_{50} = 50, 75 μM against MCF-7
and MDA-MB231, respectively

Fig. S12 (continued). Antiproliferation properties (μM) of tetrahydro-β-carboline and isatin scaffolds connected by 1*H*-1,2,3-triazolyl heterocycle **109** and reference standards (Plumbagin, Peganumine A and Tamoxifen).

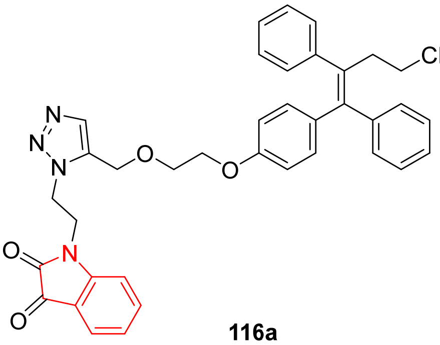

116a

IC_{50} = 53.10, >100 µM against MCF-7 and MDA-MB-231, respectively

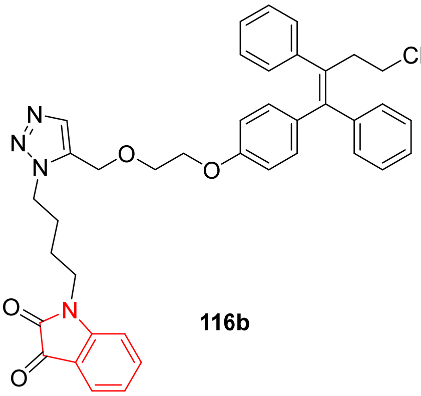

116b

IC_{50} = >100, >100 µM against MCF-7 and MDA-MB-231, respectively

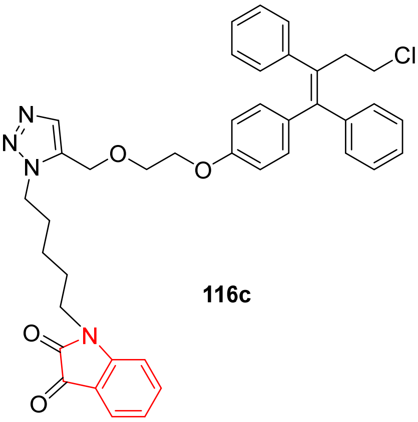

116c

IC_{50} = >100, >100 µM against MCF-7 and MDA-MB-231, respectively

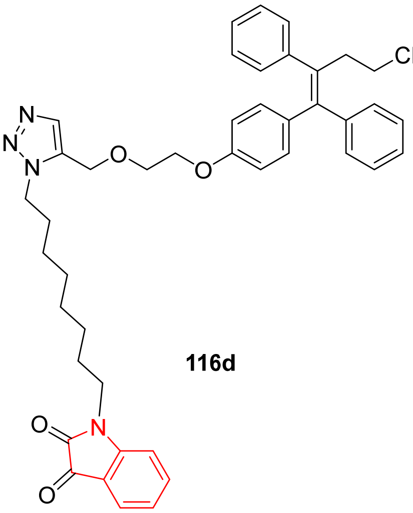

116d

IC_{50} = >100, >100 µM against MCF-7 and MDA-MB-231, respectively

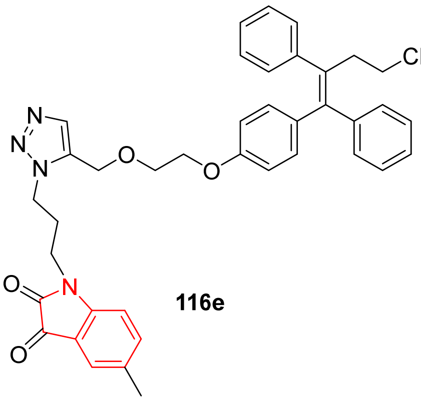

116e

IC_{50} = 70.71, >100 µM against MCF-7 and MDA-MB-231, respectively

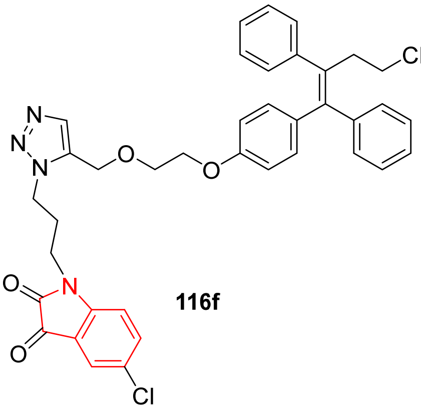

116f

IC_{50} = >100, >100 µM against MCF-7 and MDA-MB-231, respectively

Fig. S13. Antiproliferation properties (µM) of the synthesized ospemifene-isatins **116** and ospemifene-spiroisatins **117** conjugates and reference standards (Ospemifene, Tamoxifen and Plumbagin) agains MCF-7 and MDA-MB-231.

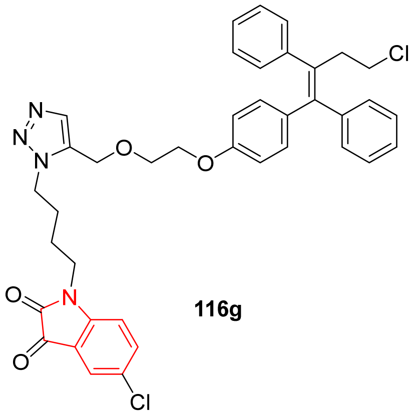

116g

IC$_{50}$ = >100, >100 μM against MCF-7
and MDA-MB-231, respectively

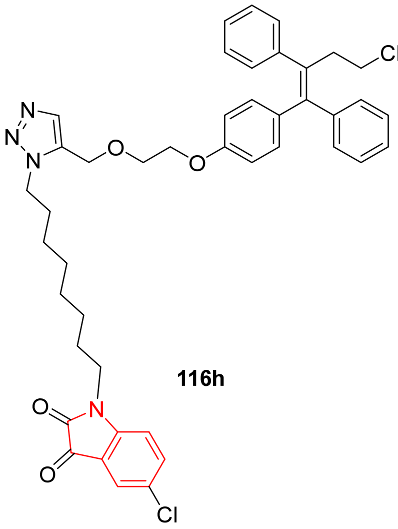

116h

IC$_{50}$ = >100, >100 μM against MCF-7
and MDA-MB-231, respectively

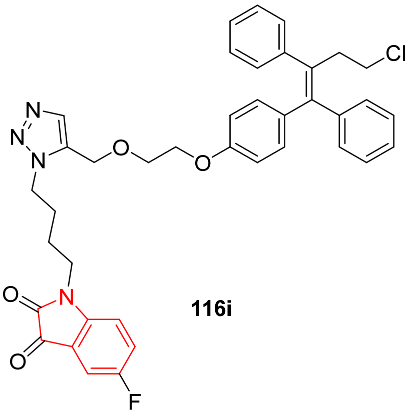

116i

IC$_{50}$ = >100, 71.40 μM against MCF-7
and MDA-MB-231, respectively

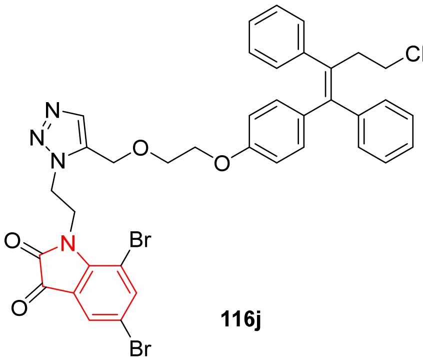

116j

IC$_{50}$ = 1.56, 48.46 μM against MCF-7
and MDA-MB-231, respectively

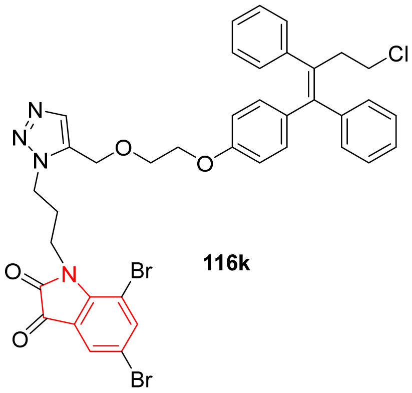

116k

IC$_{50}$ = 16.54, >100 μM against MCF-7
and MDA-MB-231, respectively

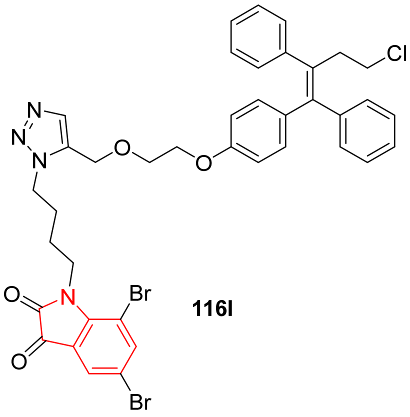

116l

IC$_{50}$ = 10.99, >71.40 μM against MCF-7
and MDA-MB-231, respectively

Fig. S13 (continued). Antiproliferation properties (μM) of the synthesized ospemifene-isatins **116** and ospemifene-spiroisatins **117** conjugates and reference standards (Ospemifene, Tamoxifen and Plumbagin) agains MCF-7 and MDA-MB-231.

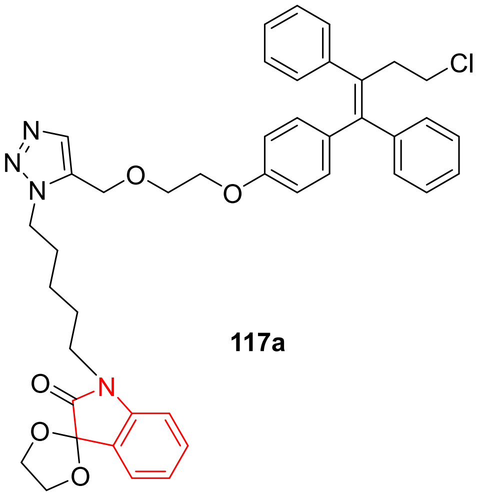

117a

IC_{50} = >100, >100 μM against MCF-7 and MDA-MB-231, respectively

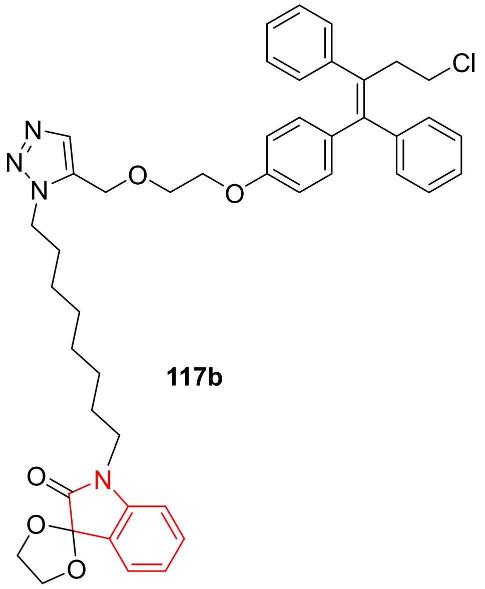

117b

IC_{50} = >100, >100 μM against MCF-7 and MDA-MB-231, respectively

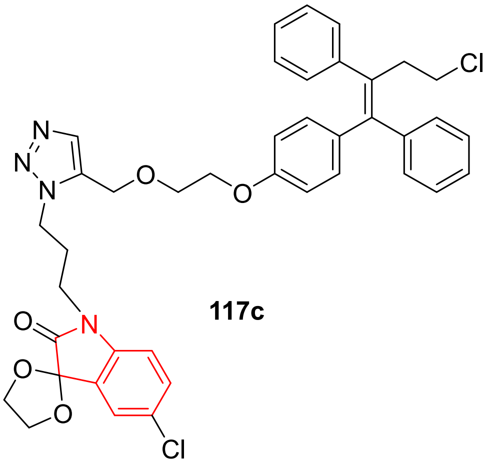

117c

IC_{50} = >100, >100 μM against MCF-7 and MDA-MB-231, respectively

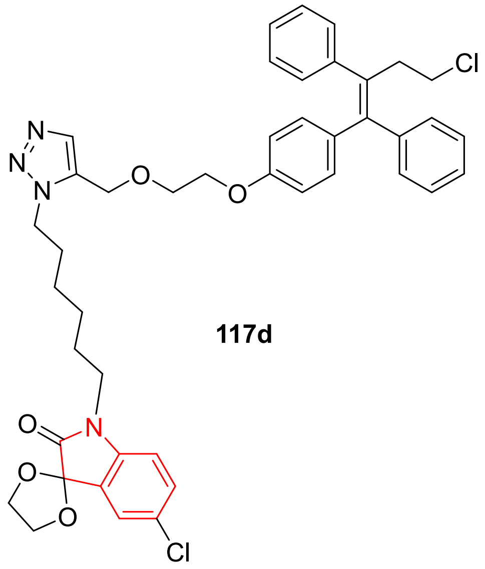

117d

IC_{50} = >100, >100 μM against MCF-7 and MDA-MB-231, respectively

Fig. S13 (continued). Antiproliferation properties (μM) of the synthesized ospemifene-isatins **116** and ospemifene-spiroisatins **117** conjugates and reference standards (Ospemifene, Tamoxifen and Plumbagin) agains MCF-7 and MDA-MB-231.

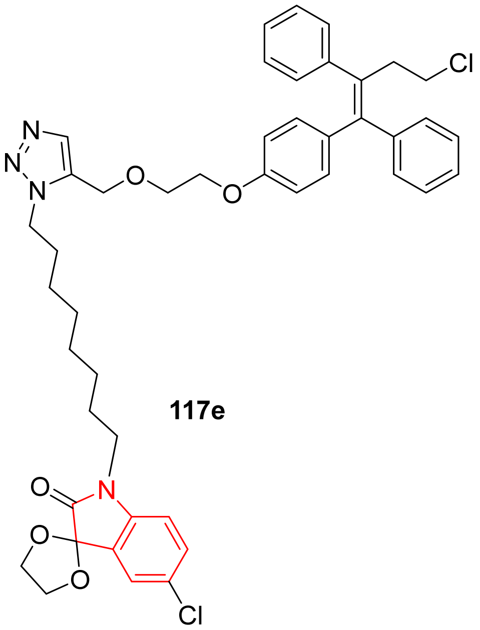

117e

IC$_{50}$ = >100, >100 μM against MCF-7
and MDA-MB-231, respectively

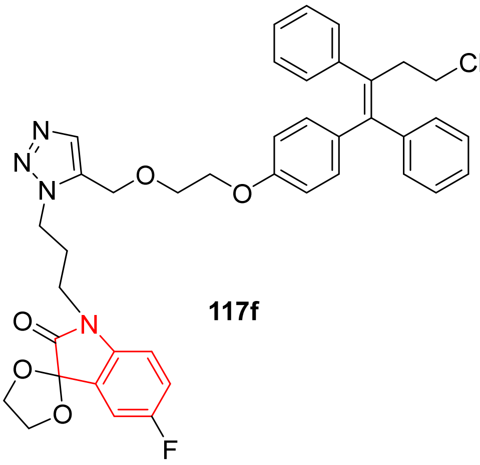

117f

IC$_{50}$ = >100, 38.35 μM against MCF-7
and MDA-MB-231, respectively

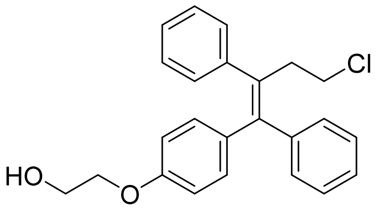

Ospemifene

IC$_{50}$ = 55, >100 μM against MCF-7
and MDA-MB-231, respectively

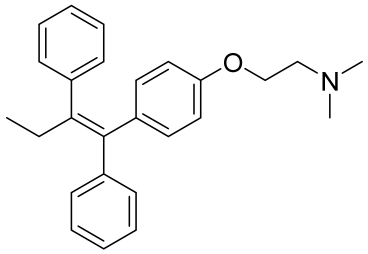

Tamoxifen

IC$_{50}$ = 50, 75 μM against MCF-7
and MDA-MB-231, respectively

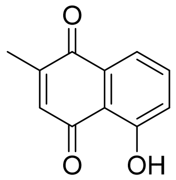

Plumbagin

IC$_{50}$ = 3.5, 4.4 μM against MCF-7
and MDA-MB-231, respectively

Fig. S13 (continued). Antiproliferation properties (μM) of the synthesized ospemifene-isatins **116** and ospemifene-spiroisatins **117** conjugates and reference standards (Ospemifene, Tamoxifen and Plumbagin) agains MCF-7 and MDA-MB-231.

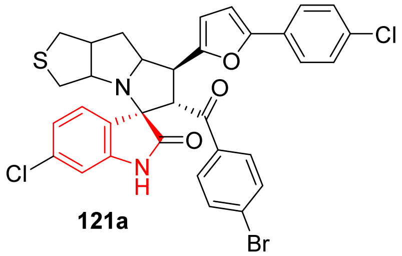

121a

IC_{50} = 63.1 ± 2.45, 91.4 ± 2.86 μM/mL against MCF-7 and HepG2 cell lines, respectively

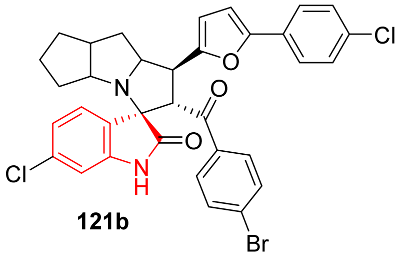

121b

IC_{50} = 18.2 ± 0.69, 27.6 ± 0.84 μM/mL against MCF-7 and HepG2 cell lines, respectively

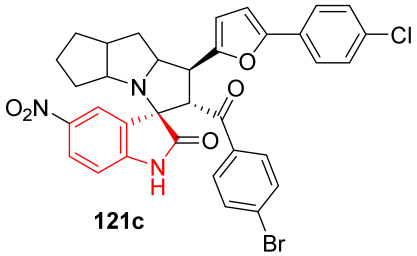

121c

IC_{50} = 36.7 ± 1.41, 45.0 ± 1.39 μM/mL against MCF-7 and HepG2 cell lines, respectively

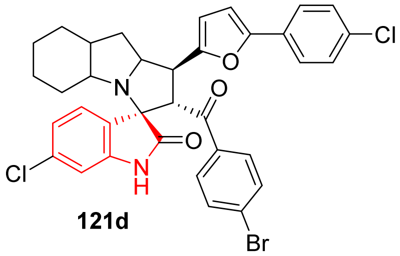

121d

IC_{50} = 4.3 ± 0.18, 6.9 ± 0.23 μM/mL against MCF-7 and HepG2 cell lines, respectively

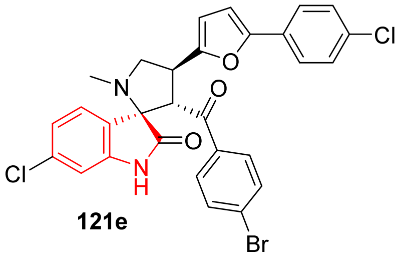

121e

IC_{50} = 41.2 ± 1.49, 25.4 ± 0.74 μM/mL against MCF-7 and HepG2 cell lines, respectively

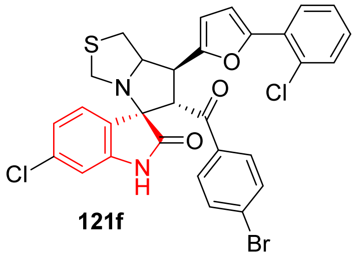

121f

IC_{50} = 10.33 ± 040, 3.5 ± 0.11 μM/mL against MCF-7 and HepG2 cell lines, respectively

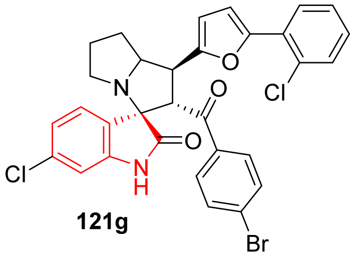

121g

IC_{50} = 60.5 ± 2.28, 36.1 ± 1.10 μM/mL against MCF-7 and HepG2 cell lines, respectively

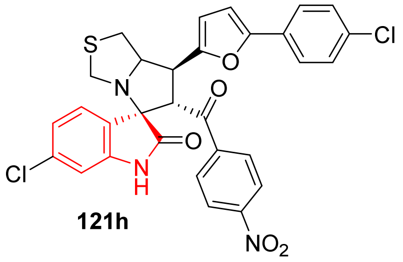

121h

IC_{50} = 174.3 ± 6.41, 54.6 ± 1.62 μM/mL against MCF-7 and HepG2 cell lines, respectively

Fig. S14. Antiproliferation properties of spiroxindoles bearing 2-furanyl heterocycle **121** and Staurosporine.

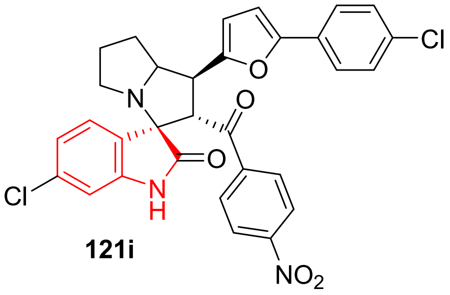

121i

IC_{50} = 10.7 ± 0.38, 19.6 ± 0.56 μM/mL against MCF-7 and HepG2 cell lines, respectively

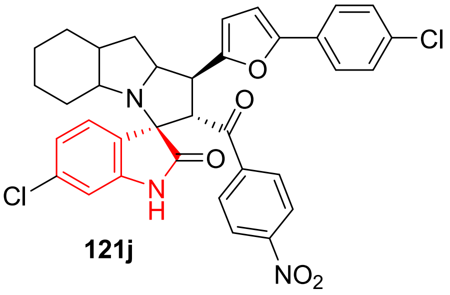

121j

IC_{50} = 4.7 ± 0.18, 11.8 ± 0.37 μM/mL against MCF-7 and HepG2 cell lines, respectively

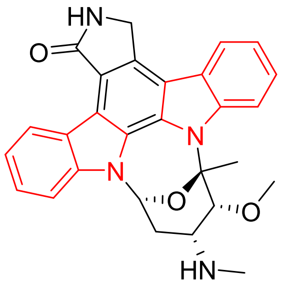

Staurosporine

IC_{50} = 17.8 ± 0.50, 10.3 ± 0.23 μM/mL against MCF-7 and HepG2 cell lines, respectively

Fig. S14 (continued). Antiproliferation properties of spiroxindoles bearing 2-furanyl heterocycle **121** and Staurosporine.

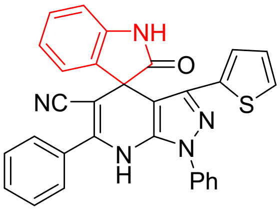

124a

IC_{50} = 6.70 μM against MDA-MB-231; IC_{50} = 0.43 ± 0.04 μM against EGFR

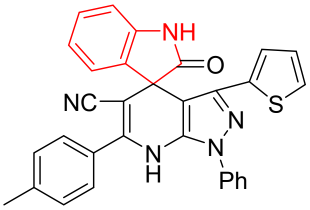

124b

IC_{50} = 29.70 μM against MDA-MB-231

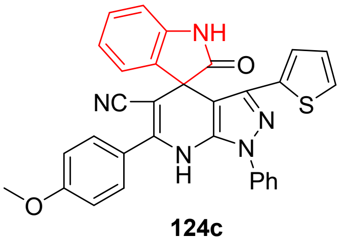

124c

IC_{50} = 37.80 μM against MDA-MB-231

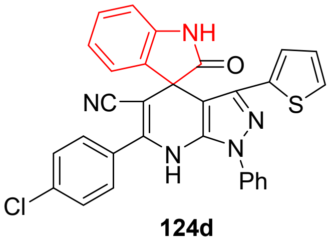

124d

IC_{50} = 12.00 μM against MDA-MB-231; IC_{50} = 0.36 ± 0.02 μM against EGFR

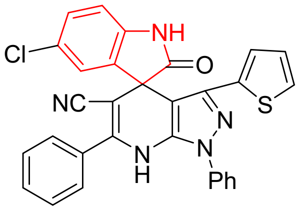

124e

IC_{50} = 6.40 μM against MDA-MB-231; IC_{50} = 0.15 ± 0.02 μM against EGFR

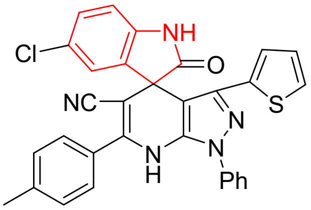

124f

IC_{50} = 18.20 μM against MDA-MB-231

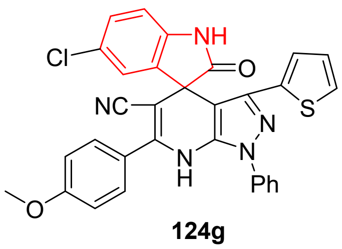

124g

IC_{50} = 24.10 μM against MDA-MB-231

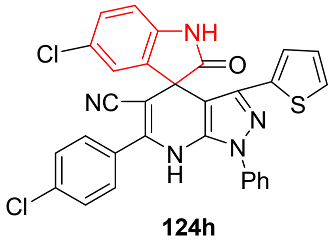

124h

IC_{50} = 16.60 μM against MDA-MB-231

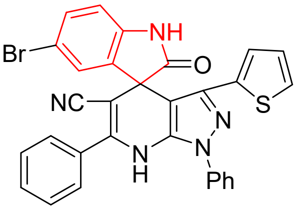

124i

IC_{50} = 6.70 μM against MDA-MB-231; IC_{50} = 0.12 ± 0.01 μM against EGFR

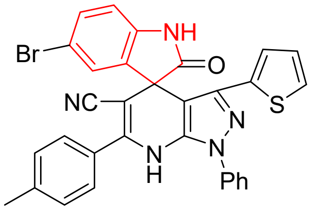

124j

IC_{50} = 13.50 μM against MDA-MB-231; IC_{50} = 0.51 ± 0.04 μM against EGFR

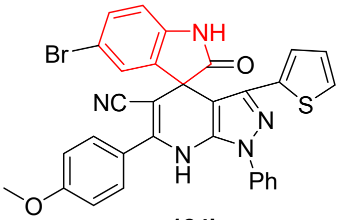

124k

IC_{50} = 12.70 μM against MDA-MB-231; IC_{50} = 0.31 ± 0.02 μM against EGFR

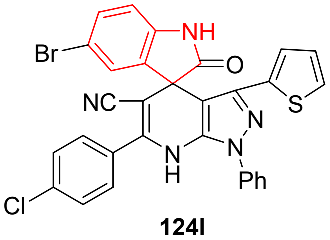

124l

IC_{50} = 30.60 μM against MDA-MB-231

Fig. S15. Antiproliferation properties (μM) against MDA-MB-231 and inhibitory properties (μM ± SD) agaist EGFR of spiroxindoles **124** and standard references [Doxorubicin (Adriamycin) and Erlotinib].

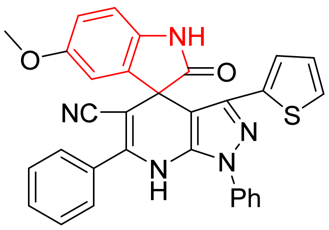

124m

IC_{50} = 17.50 μM against MDA-MB-231

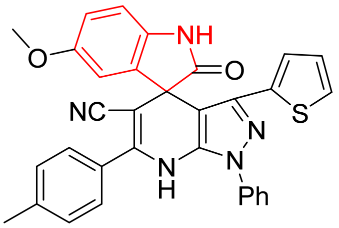

124n

IC_{50} = >50 μM against MDA-MB-231

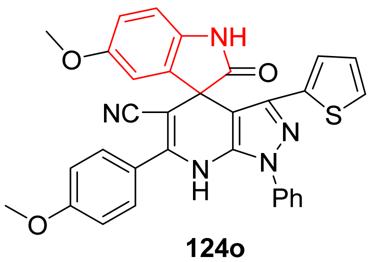

124o

IC_{50} = >50 μM against MDA-MB-231

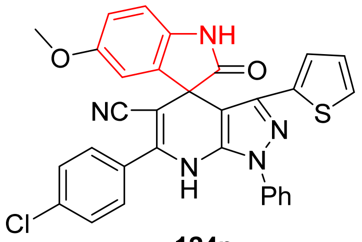

124p

IC_{50} = >50 μM against MDA-MB-231

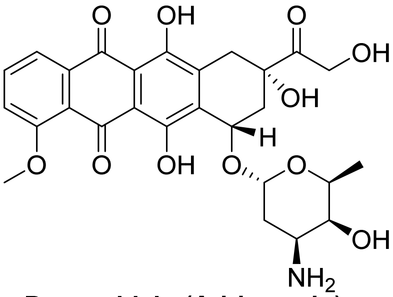

Doxorubicin (Adriamycin)

IC_{50} = 0.12 μM against MDA-MB-231

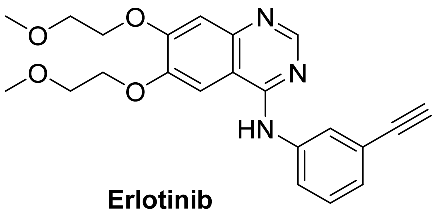

Erlotinib

IC_{50} = 0.11 ± 0.01 μM against EGFR

Fig. S15 (continued). Antiproliferation properties (μM) against MDA-MB-231 and inhibitory properties (μM ± SD) agaist EGFR of spiroxindoles **124** and standard references [Doxorubicin (Adriamycin) and Erlotinib].

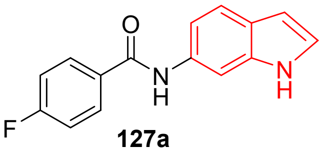

127a

IC_{50} = 45.93 ± 0.98, 35.06 ± 0.75 μM against MCF-7 and T-47D, respectively; IC_{50} = 69.46 nM against ER-α

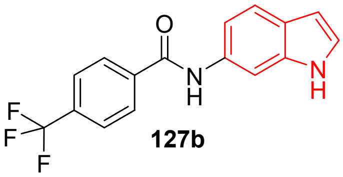

127b

IC_{50} = 63.43 ± 2.14, 51.34 ± 0.98 μM against MCF-7 and T-47D, respectively; IC_{50} = 8.15 nM against ER-α

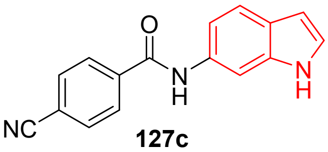

127c

IC_{50} = 33.56 ± 0.46, 35.75 ± 0.95 μM against MCF-7 and T-47D, respectively; IC_{50} = 2.40 nM against ER-α

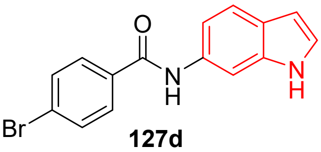

127d

IC_{50} = 38.14 ± 0.85, 63.74 ± 2.30 μM against MCF-7 and T-47D, respectively; IC_{50} = 4.71 nM against ER-α

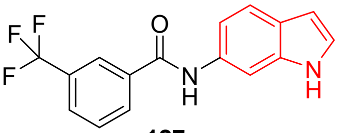

127e

IC_{50} = 30.63 ± 0.72, 28.23 ± 0.95 μM against MCF-7 and T-47D, respectively; IC_{50} = 1.76 nM against ER-α

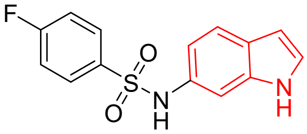

128a

IC_{50} = 30.89 ± 0.96, 32.96 ± 0.69 μM against MCF-7 and T-47D, respectively; IC_{50} = 3.31 nM against ER-α

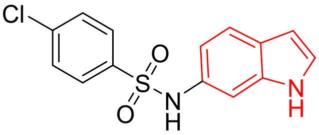

128b

IC_{50} = 54.02 ± 0.84, 46.16 ± 1.76 μM against MCF-7 and T-47D, respectively; IC_{50} = 44.43 nM against ER-α

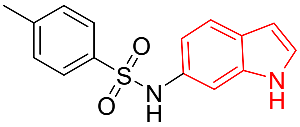

128c

IC_{50} = 37.2 ± 1.20, 57.13 ± 1.43 μM against MCF-7 and T-47D, respectively; IC_{50} = 204.75 nM against ER-α

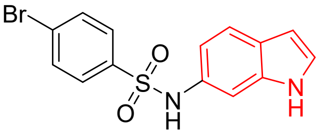

128d

IC_{50} = 37.75 ± 1.08, 40.06 ± 1.53 μM against MCF-7 and T-47D, respectively; IC_{50} = 22.67 nM against ER-α

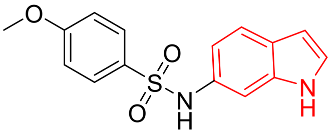

128e

IC_{50} = 57.22 ± 2.02, 45.04 ± 0.89 μM against MCF-7 and T-47D, respectively; IC_{50} = 7.53 nM against ER-α

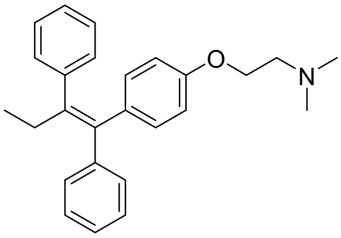

Tamoxifen

IC_{50} = 42.40 ± 0.78, 34.42 ± 0.83 μM against MCF-7 and T-47D, respectively; IC_{50} = 1.50 nM against ER-α

Fig. S16. Antiproliferation properties (μM ± SD) and EF-α inhibitory properties (nM ± SD) of the synthesized *N*-(1*H*-indole-6-yl) benzamides **127** and their benzene sulfonamide analogs **128**.

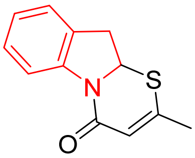

131a

IC_{50} = 302, 116 μM against MDA-231 and MDA-468, respectively

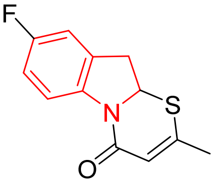

131b

IC_{50} = 827, 793 μM against MDA-231 and MDA-468, respectively

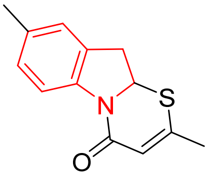

131c

IC_{50} = 1075, 423 μM against MDA-231 and MDA-468, respectively

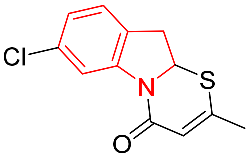

131d

IC_{50} = 825, 380 μM against MDA-231 and MDA-468, respectively

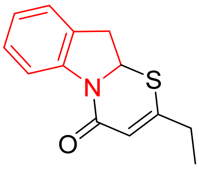

131e

IC_{50} = 3183, 3040 μM against MDA-231 and MDA-468, respectively

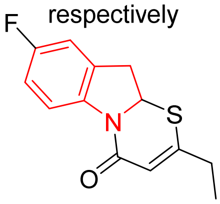

131f

IC_{50} = 996, 202 μM against MDA-231 and MDA-468, respectively

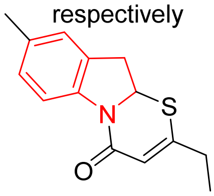

131g

IC_{50} = 1361, 206 μM against MDA-231 and MDA-468, respectively

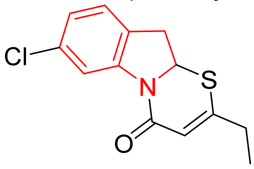

131h

IC_{50} = >2000, >2000 μM against MDA-231 and MDA-468, respectively

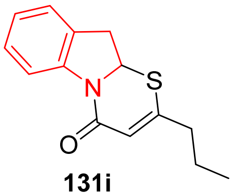

131i

IC_{50} = 690, 736 μM against MDA-231 and MDA-468, respectively

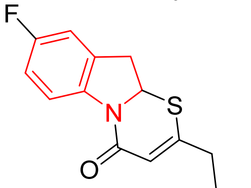

131j

IC_{50} = 790, 785 μM against MDA-231 and MDA-468, respectively

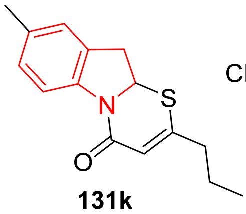

131k

IC_{50} = 330, 97 μM against MDA-231 and MDA-468, respectively

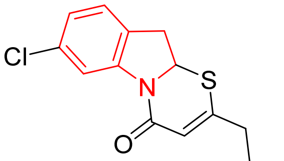

131l

IC_{50} = >2000, 475 μM against MDA-231 and MDA-468, respectively

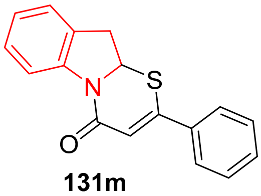

131m

IC_{50} = >2000, >2000 μM against MDA-231 and MDA-468, respectively

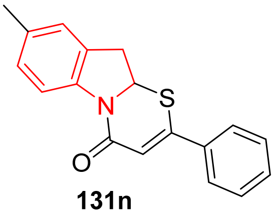

131n

IC_{50} = >2000, >2000 μM against MDA-231 and MDA-468, respectively

Fig. S17. Antiproliferation properties (μM) of [1,3]thiazino[3,2-*a*]indol-4-ones **131**.

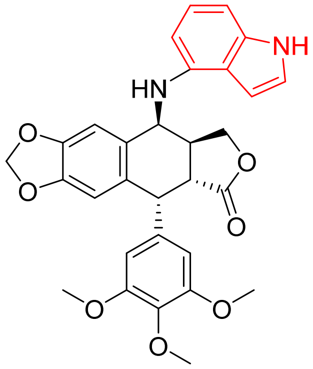

133a

IC_{50} = 1.8 ± 0.2, 33.6 ± 1.5,
2.1 ± 0.3, 29.2 ± 3.5,
2.4 ± 0.2, 32.4 ± 2.9,
1.3 ± 0.2, 64.2 ± 2.4 μM
against HepG-2, HL-7702,
HeLa, H8, A549, MRC-5,
MCF-7, and HMEC, respectively

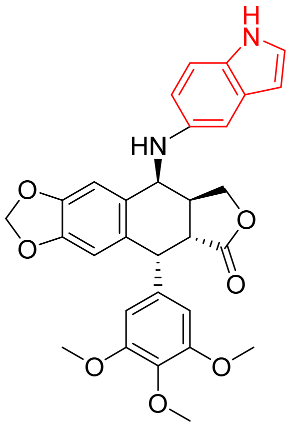

133b

IC_{50} = 0.9 ± 0.1, 43.3 ± 2.8,
0.9 ± 0.01, 29.2 ± 3.5,
0.5 ± 0.01, 51.3 ± 3.1,
0.4 ± 0.01, 55.2 ± 5.3 μM
against HepG-2, HL-7702,
HeLa, H8, A549, MRC-5,
MCF-7, and HMEC, respectively

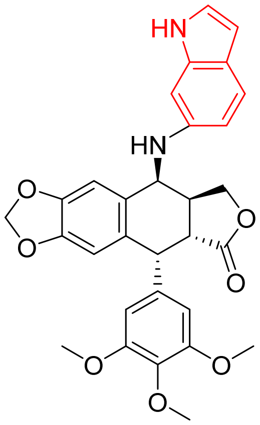

133c

IC_{50} = 0.1 ± 0.01, 94.6 ± 3.2,
0.08 ± 0.0, 84.5 ± 2.9,
0.08 ± 0.0, 69.1 ± 2.9,
0.07 ± 0.0, 61.6 ± 2.3 μM
against HepG-2, HL-7702,
HeLa, H8, A549, MRC-5,
MCF-7, and HMEC, respectively

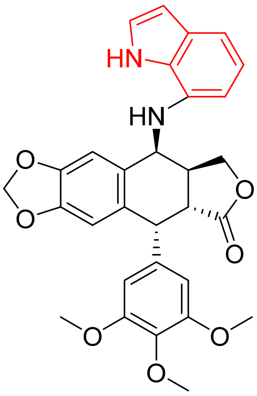

133d

IC_{50} = 2.5 ± 0.6, 30.5 ± 2.7,
3.8 ± 0.2, 19.4 ± 1.7,
2.2 ± 0.9, 38.5 ± 0.9,
4.8 ± 0.9, 45.5 ± 3.5 μM
against HepG-2, HL-7702,
HeLa, H8, A549, MRC-5,
MCF-7, and HMEC, respectively

Fig. S18. Antiproliferation properties (μM ± SD) of the synthesized indole-podophyllotoxin conjugates **133**, and standards (Colchicine, Podophyllotoxin and Nocodazole) against HepG-2, HL-7702 (liver), HeLa, H8 (cervical), A549, MRC-5 (lung), MCF-7, and HMEC (breast) cancer cell lines.

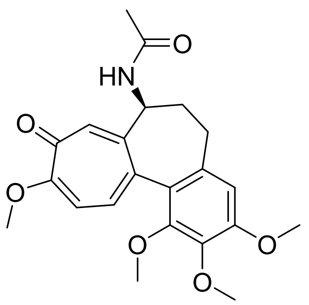

Colchicine

IC_{50} = 5.8 ± 0.1, 9.2 ± 0.3,
10.2 ± 0.4, 6.1 ± 0.4,
9.7 ± 0.2, 6.2 ± 0.7,
14.3 ± 0.5, 8.1 ± 0.2 μM
against HepG-2, HL-7702,
HeLa, H8, A549, MRC-5,
MCF-7, and HMEC, respectively

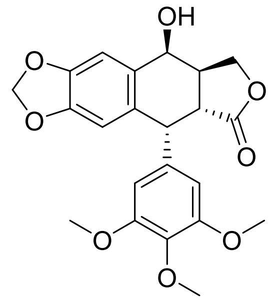

Podophyllotoxin

IC_{50} = 2.4 ± 0.1, 5.0 ± 0.6,
6.9 ± 0.1, 8.4 ± 0.5,
2.6 ± 0.1, 10.6 ± 1.2,
2.4 ± 0.3, 5.6 ± 0.1 μM
against HepG-2, HL-7702,
HeLa, H8, A549, MRC-5,
MCF-7, and HMEC, respectively

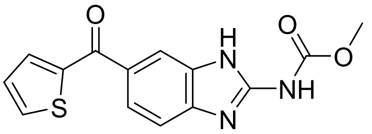

Nocodazole

IC_{50} = 0.4 ± 0.2, 13.5 ± 1.5,
0.3 ± 0.1, 18.0 ± 2.6,
0.2 ± 0.05, 15.3 ± 1.9,
0.2 ± 0.1, 11.5 ± 2.2 μM
against HepG-2, HL-7702,
HeLa, H8, A549, MRC-5,
MCF-7, and HMEC, respectively

Fig. S18 (continued). Antiproliferation properties (μM ± SD) of the synthesized indole-podophyllotoxin conjugates **133**, and standards (Colchicine, Podophyllotoxin and Nocodazole) against HepG-2, HL-7702 (liver), HeLa, H8 (cervical), A549, MRC-5 (lung), MCF-7, and HMEC (breast) cancer cell lines.

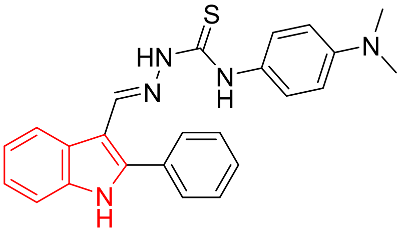

139a

IC_{50} = >100, >100, >100 µM against
MCF-7, A-549 and HepG-2, respectively

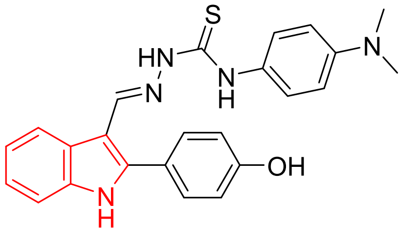

139b

IC_{50} = 31.38 ± 4.96, >100, >100 µM against
MCF-7, A-549 and HepG-2, respectively

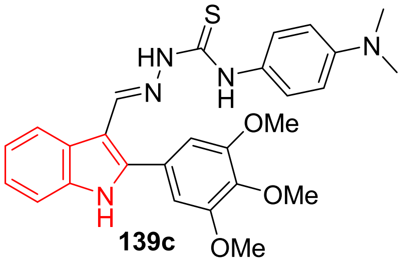

139c

IC_{50} = 35.78 ± 6.89, >100, >100 µM against
MCF-7, A-549 and HepG-2, respectively

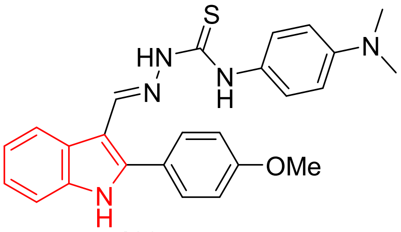

139d

IC_{50} = >100, >100, 36.43 ± 4.73 µM against
MCF-7, A-549 and HepG-2, respectively

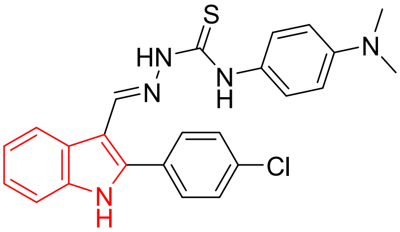

139e

IC_{50} = >100, >100, 72.68 ± 6.45 µM against
MCF-7, A-549 and HepG-2, respectively

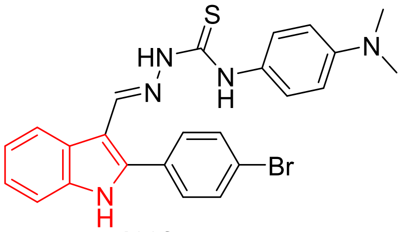

139f

IC_{50} = >100, >100, >100 µM against
MCF-7, A-549 and HepG-2, respectively

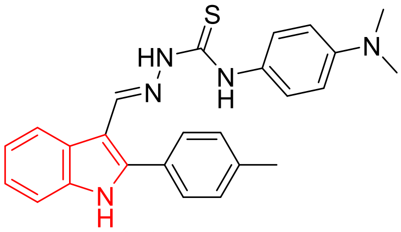

139g

IC_{50} = 23.39 ± 5.47, 80.84 ± 7.5,
24.46 ± 1.05 µM against
MCF-7, A-549 and HepG-2, respectively

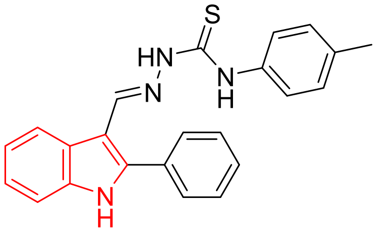

139h

IC_{50} = >100, >100, 42.83 ± 5.54 µM against
MCF-7, A-549 and HepG-2, respectively

Fig. S19. Antiproliferation properties (µM ± SD) of the synthesized indolylthiosemicarbazones **139** and standards (Etoposide and Colchicine).

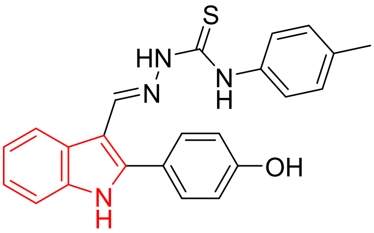

139i

IC_{50} = 24.97 ± 5.32, >100, >100 μM against
MCF-7, A-549 and HepG-2, respectively

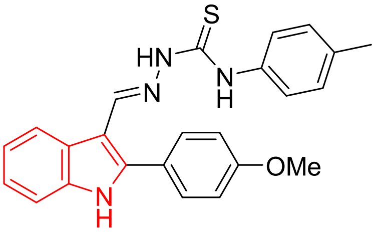

139j

IC_{50} = >100, >100, >100 μM against
MCF-7, A-549 and HepG-2, respectively

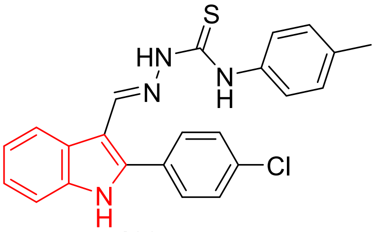

139k

IC_{50} = >100, >100, >100 μM against
MCF-7, A-549 and HepG-2, respectively

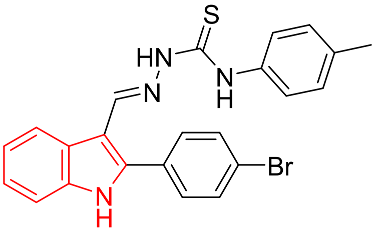

139l

IC_{50} = >100, >100, >100 μM against
MCF-7, A-549 and HepG-2, respectively

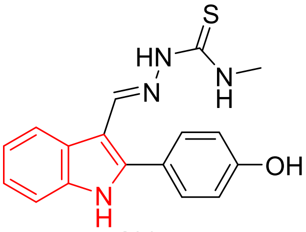

139m

IC_{50} = >100, 24.20 ± 1.70,
51.70 ± 4.10 μM against
MCF-7, A-549 and HepG-2, respectively

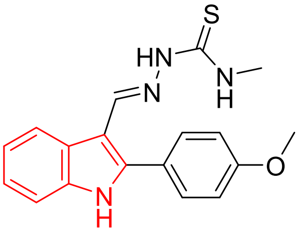

139n

IC_{50} = >100, 12.50 ± 1.00,
56.00 ± 6.30 μM against
MCF-7, A-549 and HepG-2, respectively

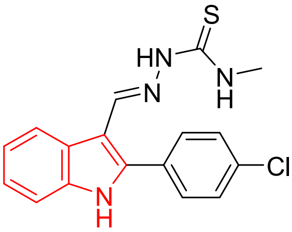

139o

IC_{50} = >100, 22.95 ± 1.63,
48.94 ± 3.91 μM against
MCF-7, A-549 and HepG-2, respectively

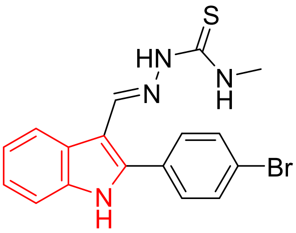

139p

IC_{50} = >100, 92.11 ± 6.33,
49.97 ± 5.52 μM against
MCF-7, A-549 and HepG-2, respectively

Fig. S19 (continued). Antiproliferation properties (μM ± SD) of the synthesized indolylthiosemicarbazones **139** and standards (Etoposide and Colchicine).

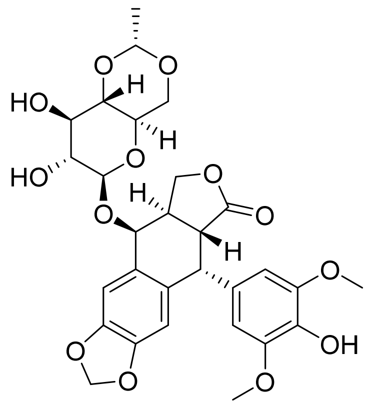

Etoposide

IC_{50} = 34.25 ± 3.12, 38.23 ± 1.89,
33.17 ± 3.19 μM against
MCF-7, A-549 and HepG-2, respectively

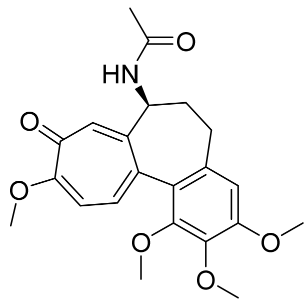

Colchicine

IC_{50} = 7.10 ± 0.61, 1.9 ± 0.23,
6.0 ± 0.49 μM against
MCF-7, A-549 and HepG-2, respectively

Fig. S19 (continued). Antiproliferation properties (μM ± SD) of the synthesized indolylthiosemicarbazones **139** and standards (Etoposide and Colchicine).

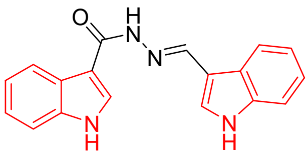

142a

IC_{50} = 18 µM against A549 cell line; % inhibition of microtubule polymerization = 10 at 10 µM of the respective ligand

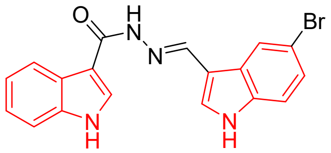

142b

IC_{50} = 7.5 µM against A549 cell line; % inhibition of microtubule polymerization = 44 at 10 µM of the respective ligand

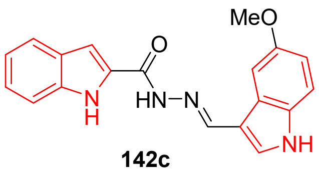

142c

IC_{50} = 2 µM against A549 cell line; % inhibition of microtubule polymerization = 62 at 10 µM of the respective ligand

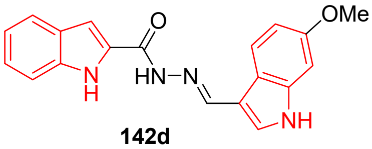

142d

IC_{50} = 6 µM against A549 cell line; % inhibition of microtubule polymerization = 53 at 10 µM of the respective ligand

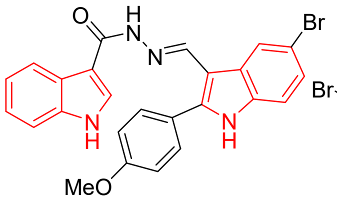

142e

IC_{50} = 21 µM against A549 cell line

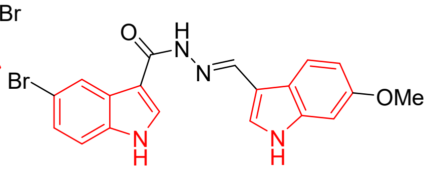

142f

IC_{50} = 16.5 µM against A549 cell line; % inhibition of microtubule polymerization = 11 at 10 µM of the respective ligand

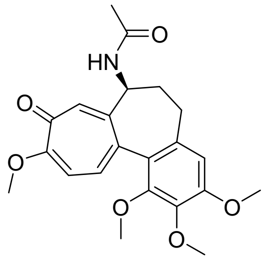

Colchicine

IC_{50} = 0.02 µM against A549 cell line; % inhibition of microtubule polymerization = 11 at 79 µM of the respective ligand

Fig. S20. Antiproliferation properties (µM) and % inhibition of microtubule polymerization at 10 µM of the respective ligand of the synthesized bis(indolyl)hydrazide-hydrazones **142** and Colchicine.

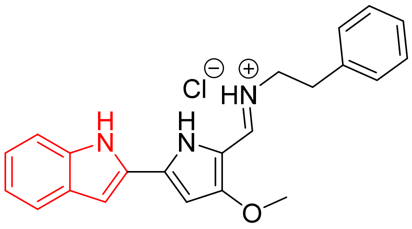

144a

IC_{50} = 10.66 ± 0.68, 8.04 ± 0.45, 8.67 ± 0.06, 8.37 ± 0.24, 4.04 ± 1.29, 4.34 ± 0.25 against A549, DMS53, SW900, H460, PC#8 and PC#13, respectively

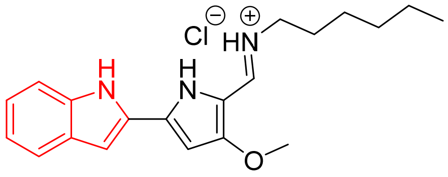

144b

IC_{50} = 7.61 ± 0.23, 6.46 ± 0.52, 7.55 ± 0.89, 7.29 ± 0.29, 3.34 ± 0.86, 4.03 ± 0.19 against A549, DMS53, SW900, H460, PC#8 and PC#13, respectively

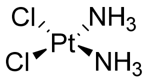

Cisplatin

IC_{50} = >200, 107.9 ± 31.16, 49.4 ± 10.56, 74.2 ± 6.72, 49.28 ± 9.88, 79.57 ± 9.37 against A549, DMS53, SW900, H460, PC#8 and PC#13, respectively

Fig. S21. Antiproliferation properties of indole-based tambjamine analogs **144** and Cisplatin.

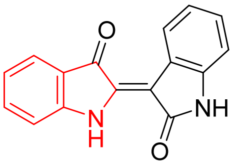

145

IC_{50} = >25, >25, >25, >25 μM against SW480, A549, HepG2 and B16F10 cell lines, respectively

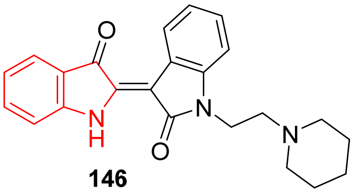

146

IC_{50} = >25, >25, >25, >25 μM against SW480, A549, HepG2 and B16F10 cell lines, respectively

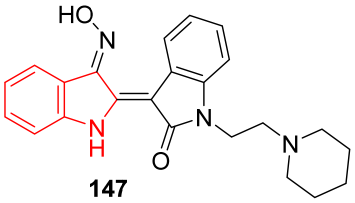

147

IC_{50} = 1.53 ± 0.01, 0.57 ± 0.07, 1.64 ± 0.11, 1.64 ± 0.03 μM against SW480, A549, HepG2 and B16F10 cell lines, respectively

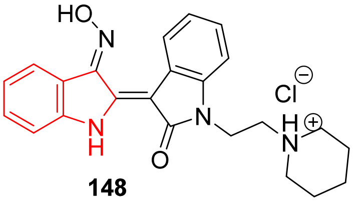

148

IC_{50} = 0.53 ± 0.06, 0.38 ± 0.01, 0.37 ± 0.01, 0.53 ± 0.01 μM against SW480, A549, HepG2 and B16F10 cell lines, respectively

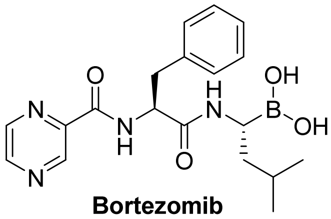

Bortezomib

IC_{50} = >25, >25, 18.17 ± 1.42, 13.470 ± 1.73 μM against SW480, A549, HepG2 and B16F10 cell lines, respectively

Fig. S22. Antiproliferation properties (μM ± SD) of indirubin **145**, indirubin-piperidine conjugates **146–148** and Bortezomib.

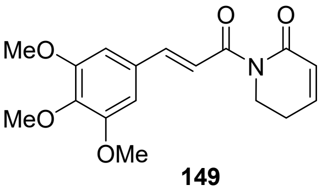

149

IC_{50} = 47.80, 50.13, 15.16, 21.74, >100 μM against A54, HCT116, ZR-75-30, MDAMB-231 and MRC-5, respectively

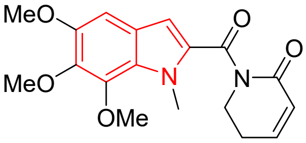

152a

IC_{50} = 36.09, 93.51, 15.83, 41.67, 51.14 μM against A54, HCT116, ZR-75-30, MDAMB-231 and MRC-5, respectively

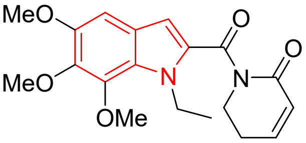

152b

IC_{50} = 44.56, 85.16, 3.16, 19.52, 64.75 μM against A54, HCT116, ZR-75-30, MDAMB-231 and MRC-5, respectively

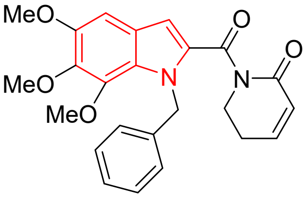

152c

IC_{50} = 27.45, 78.68, 24.09, 50.04, 90.07 μM against A54, HCT116, ZR-75-30, MDAMB-231 and MRC-5, respectively

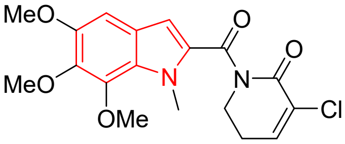

152d

IC_{50} = 12.67, 4.73, 2.24, 10.30, 45.70 μM against A54, HCT116, ZR-75-30, MDAMB-231 and MRC-5, respectively

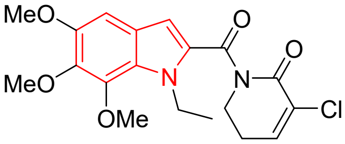

152e

IC_{50} = 19.17, 7.99, 2.88, 12.30, 57.96 μM against A54, HCT116, ZR-75-30, MDAMB-231 and MRC-5, respectively

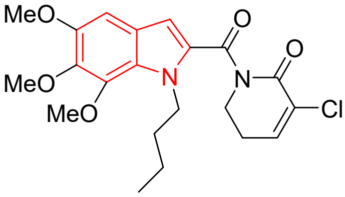

152f

IC_{50} = 23.54, 8.40, 3.57, 8.59, 44.56 μM against A54, HCT116, ZR-75-30, MDAMB-231 and MRC-5, respectively

Fig. S23. Antiproliferation properties (μM) of the synthesized piperlongumine pharmacophore conjugated with indolyl scaffold **152**. and its precursor piperlongumine **149**.

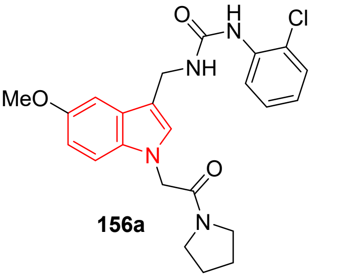

156a

% control = 47.52 ± 2.18, 67.77 ± 1.94, 95.70 ± 4.83, 99.30 ± 4.88, 69.56 ± 3.36 against DDR1, DDR2, TRK-A, TRK-B, and TRK-C, respectively at 1.0 μM

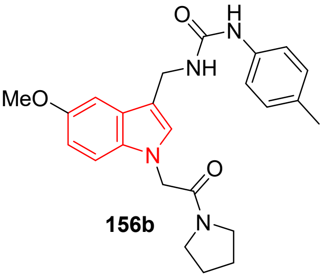

156b

% control = 69.92 ± 0.86, 84.85 ± 2.92, 75.25 ± 0.35, 87.87 ± 6.06, 77.49 ± 2.98 against DDR1, DDR2, TRK-A, TRK-B, and TRK-C, respectively at 1.0 μM

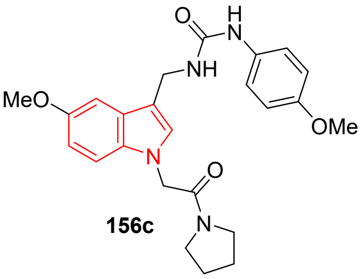

156c

% control = 81.6 ± 2.50, 91.50 ± 3.90, 112.7 ± 4.76, 102.4 ± 4.95, 88.11 ± 1.25 against DDR1, DDR2, TRK-A, TRK-B, and TRK-C, respectively at 1.0 μM

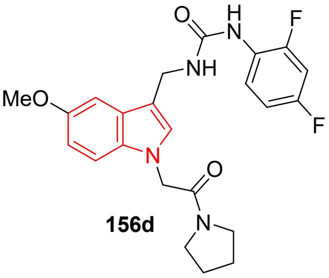

156d

% control = 53.71 ± 1.81, 50.83 ± 3.12, 74.02 ± 1.81, 104.0 ± 1.44, 76.23 ± 5.37 against DDR1, DDR2, TRK-A, TRK-B, and TRK-C, respectively at 1.0 μM; IC_{50} = 1.37 ± 0.14, 1.33 ± 0.094 μM against DDR1 and DDR2, respectively; IC_{50} = 6.96 ± 0.64, 7.35 ± 0.76, 3.72 ± 0.28 μM against A549, SPC-A-1 and H1975, respectively

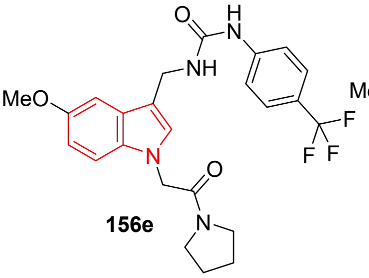

156e

% control = 94.66 ± 3.85, 99.52 ± 2.75, 90.39 ± 0.85, 72.2 ± 2.84, 89.72 ± 4.11 against DDR1, DDR2, TRK-A, TRK-B, and TRK-C, respectively at 1.0 μM

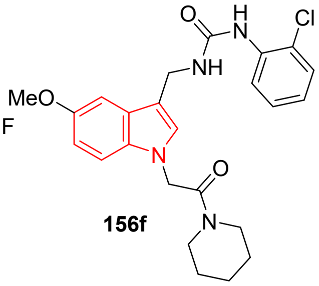

156f

% control = 55.26 ± 1.10, 64.35 ± 0.91, 74.75 ± 2.15, 87.42 ± 4.22, 68.90 ± 3.39 against DDR1, DDR2, TRK-A, TRK-B, and TRK-C, respectively at 1.0 μM

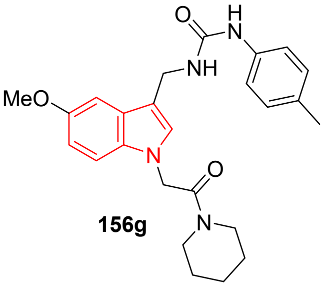

156g

% control = 103.0 ± 7.94, 83.2 ± 2.61, 82.27 ± 4.35, 85.68 ± 2.98, 92.48 ± 2.75 against DDR1, DDR2, TRK-A, TRK-B, and TRK-C, respectively at 1.0 μM

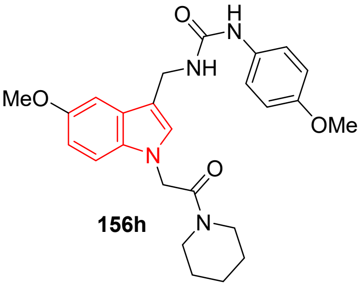

156h

% control = 66.73 ± 2.87, 72.42 ± 3.06, 92.12 ± 3.34, 87.4 ± 3.44, 92.69 ± 1.15 against DDR1, DDR2, TRK-A, TRK-B, and TRK-C, respectively at 1.0 μM

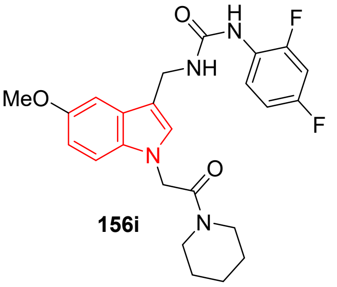

156i

% control = 48.0 ± 1.56, 74.75 ± 6.43, 84.71 ± 4.51, 70.76 ± 4.35, 68.56 ± 3.72 against DDR1, DDR2, TRK-A, TRK-B, and TRK-C, respectively at 1.0 μM

Fig. S24. Kinase inhibitory (% control at 1.0 μM) and antiproliferation (μM ± SD) properties of the synthesized indole-containing compounds linked to urea function **156** and Dasatinib (reference standard).

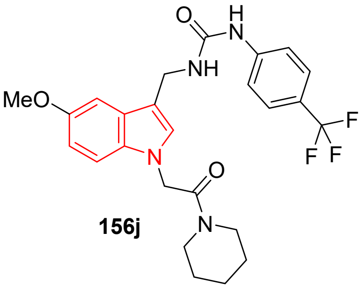

156j

% control = 100.9 ± 6.28,
73.75 ± 4.53, 91.60 ± 2.26,
74.94 ± 1.92, 104.7 ± 4.58
against DDR1, DDR2, TRK-A,
TRK-B, and TRK-C, respectively
at 1.0 μM

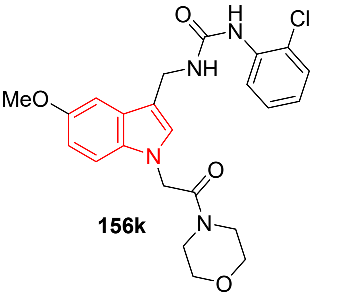

156k

% control = 76.1 ± 0.69,
54.72 ± 2.26, 91.73 ± 4.11,
97.24 ± 4.19, 85.98 ± 4.91
against DDR1, DDR2, TRK-A,
TRK-B, and TRK-C, respectively
at 1.0 μM

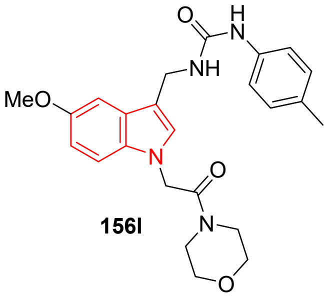

156l

% control = 73.68 ± 2.37,
89.61 ± 2.56, 81.27 ± 5.62,
83.99 ± 3.46, 87.55 ± 3.60
against DDR1, DDR2, TRK-A,
TRK-B, and TRK-C, respectively
at 1.0 μM

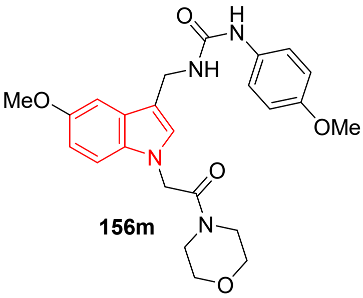

156m

% control = 63.20 ± 2.80,
95.27 ± 4.54, 98.88 ± 4.45,
92.48 ± 2.75, 89.03 ± 1.47
against DDR1, DDR2, TRK-A,
TRK-B, and TRK-C, respectively
at 1.0 μM

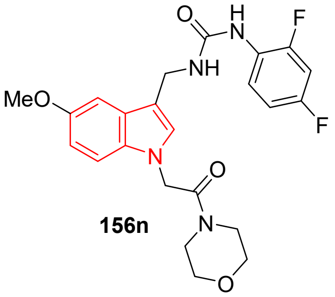

156n

% control = 75.67 ± 1.91,
74.70 ± 3.49, 77.77 ± 5.37,
73.14 ± 2.98, 60.55 ± 2.96
against DDR1, DDR2, TRK-A,
TRK-B, and TRK-C, respectively
at 1.0 μM

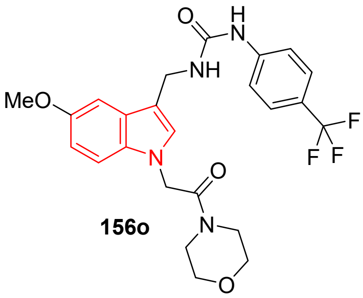

156o

% control = 90.2 ± 2.93,
82.16 ± 2.12, 71.75 ± 2.76,
79.04 ± 2.84, 102.6 ± 3.90
against DDR1, DDR2, TRK-A,
TRK-B, and TRK-C, respectively
at 1.0 μM

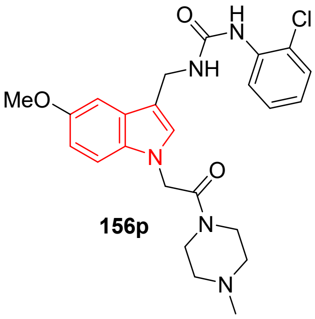

156p

% control = 66.55 ± 0.31,
55.91 ± 2.99, 94.03 ± 3.09,
93.44 ± 1.81, 82.89 ± 2.67
against DDR1, DDR2, TRK-A,
TRK-B, and TRK-C, respectively
at 1.0 μM

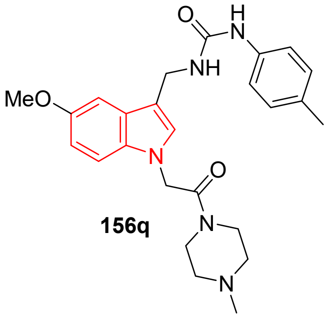

156q

% control = 85.42 ± 2.14,
96.15 ± 3.66, 98.65 ± 4.02,
108.3 ± 3.97, 90.86 ± 2.34
against DDR1, DDR2, TRK-A,
TRK-B, and TRK-C, respectively
at 1.0 μM

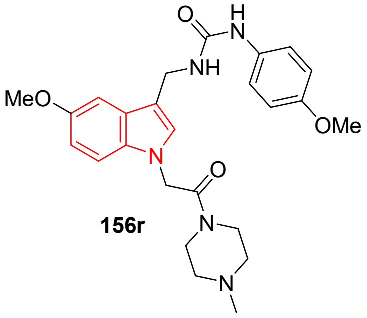

156r

% control = 81.9 ± 4.01,
93.41 ± 4.41, 108 ± 7.05,
107.6 ± 4.85, 94.55 ± 4.31
against DDR1, DDR2, TRK-A,
TRK-B, and TRK-C, respectively
at 1.0 μM

Fig. S24 (continued). Kinase inhibitory (% control at 1.0 μM) and antiproliferation (μM ± SD) properties of the synthesized indole-containing compounds linked to urea function **156** and Dasatinib (reference standard).

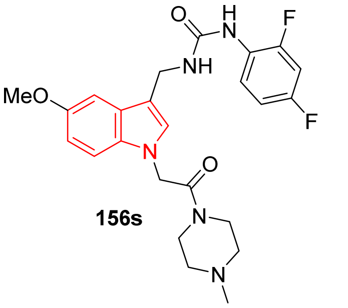

156s

% control = 62.37 ± 2.86, 66.55 ± 2.21, 93.1 ± 1.55, 88.88 ± 1.24, 68.83 ± 4.92 against DDR1, DDR2, TRK-A, TRK-B, and TRK-C, respectively at 1.0 μM

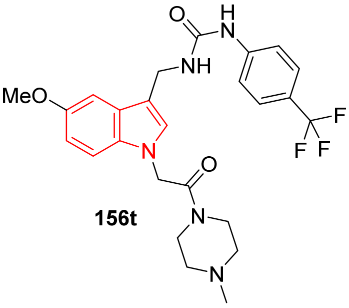

156t

% control = 75.46 ± 1.08, 101.5 ± 2.72, 95.97 ± 8.62, 104.3 ± 5.96, 105.5 ± 1.33 against DDR1, DDR2, TRK-A, TRK-B, and TRK-C, respectively at 1.0 μM

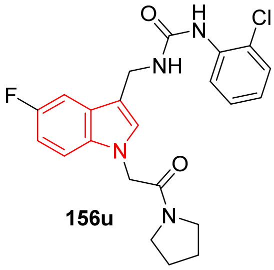

156u

% control = 55.46 ± 2.54, 73.75 ± 1.76, 78.03 ± 2.44, 81.45 ± 3.36, 68.64 ± 2.65 against DDR1, DDR2, TRK-A, TRK-B, and TRK-C, respectively at 1.0 μM

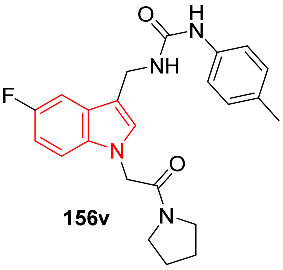

156v

% control = 69.59 ± 2.68, 95.37 ± 3.03, 100.6 ± 2.33, 99.52 ± 4.31, 91.18 ± 2.76 against DDR1, DDR2, TRK-A, TRK-B, and TRK-C, respectively at 1.0 μM

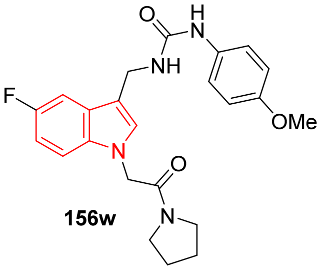

156w

% control = 86.92 ± 2.33, 86.33 ± 3.16, 71.61 ± 3.82, 83.83 ± 0.67, 110.5 ± 3.63 against DDR1, DDR2, TRK-A, TRK-B, and TRK-C, respectively at 1.0 μM

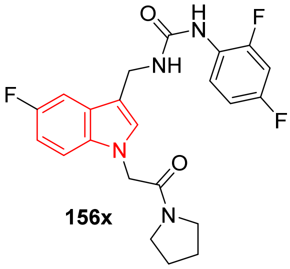

156x

% control = 40.59 ± 1.53, 32.53 ± 0.75, 95.7 ± 2.83, 69.53 ± 2.30, 75.19 ± 3.62 against DDR1, DDR2, TRK-A, TRK-B, and TRK-C, respectively at 1.0 μM; IC_{50} = 1.86 ± 0.19, 1.67 ± 0.15 μM against DDR1 and DDR2, respectively; IC_{50} = 11.18 ± 0.95, 10.48 ± 1.26, 5.34 ± 0.57 μM against A549, SPC-A-1 and H1975, respectively

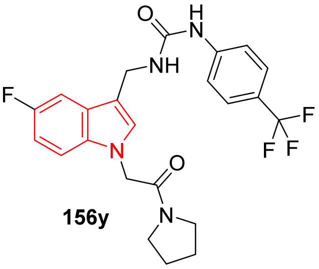

156y

% control = 65.34 ± 4.06, 81.06 ± 0.99, 100.6 ± 4.10, 101.2 ± 2.99, 94.31 ± 7.29 against DDR1, DDR2, TRK-A, TRK-B, and TRK-C, respectively at 1.0 μM

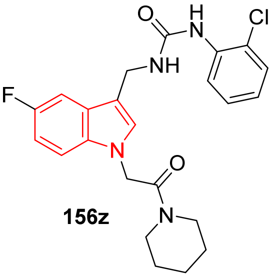

156z

% control = 63.36 ± 4.46, 47.99 ± 1.92, 90.3 ± 2.58, 75.72 ± 1.94, 84.18 ± 2.53 against DDR1, DDR2, TRK-A, TRK-B, and TRK-C, respectively at 1.0 μM

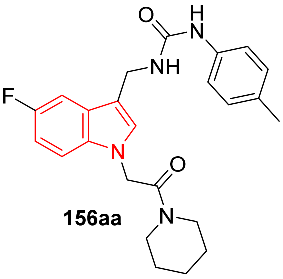

156aa

% control = 91.22 ± 2.30, 92.46 ± 5.71, 99.66 ± 6.48, 97.03 ± 3.89, 99.65 ± 2.65 against DDR1, DDR2, TRK-A, TRK-B, and TRK-C, respectively at 1.0 μM

Fig. S24 (continued). Kinase inhibitory (% control at 1.0 μM) and antiproliferation (μM ± SD) properties of the synthesized indole-containing compounds linked to urea function **156** and Dasatinib (reference standard).

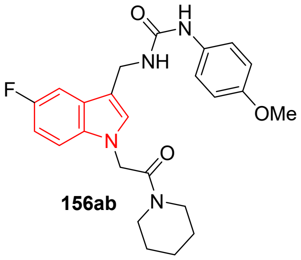

156ab

% control = 77.06 ± 4.14, 80.58 ± 5.27, 92.69 ± 0.43, 80.08 ± 3.32, 101 ± 9.07 against DDR1, DDR2, TRK-A, TRK-B, and TRK-C, respectively at 1.0 μM

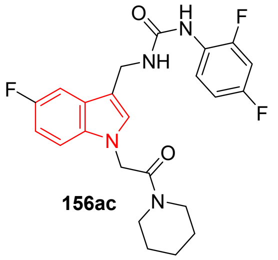

156ac

% control = 30.18 ± 1.28, 41.27 ± 2.01, 88.2 ± 3.81, 106.0 ± 4.36, 83.55 ± 4.75 against DDR1, DDR2, TRK-A, TRK-B, and TRK-C, respectively at 1.0 μM; IC_{50} = 0.42 ± 0.031, 1.19 ± 0.099 μM against DDR1 and DDR2, respectively; IC_{50} = 1.67 ± 0.16, 1.73 ± 0.12, 3.86 ± 0.35 μM against A549, SPC-A-1 and H1975, respectively

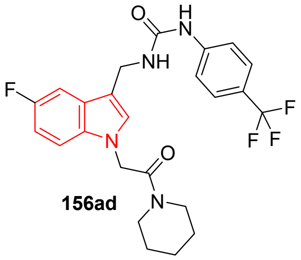

156ad

% control = 68.34 ± 4.47, 70.12 ± 3.11, 87.05 ± 2.81, 108.1 ± 5.98, 104.9 ± 2.28 against DDR1, DDR2, TRK-A, TRK-B, and TRK-C, respectively at 1.0 μM

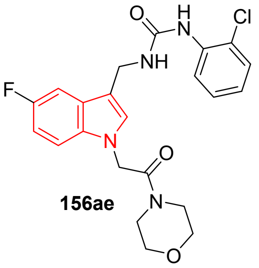

156ae

% control = 50.66 ± 2.09, 54.53 ± 2.72, 78.02 ± 2.34, 83.83 ± 1.17, 87.42 ± 3.20 against DDR1, DDR2, TRK-A, TRK-B, and TRK-C, respectively at 1.0 μM

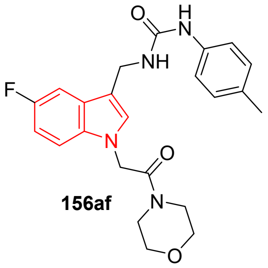

156af

% control = 69.92 ± 2.34, 72.42 ± 2.52, 113.6 ± 3.73, 83.81 ± 2.49, 111.4 ± 7.25 against DDR1, DDR2, TRK-A, TRK-B, and TRK-C, respectively at 1.0 μM

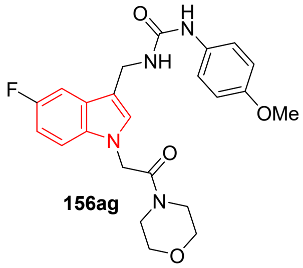

156ag

% control = 96.82 ± 5.37, 79.40 ± 2.56, 90.78 ± 3.16, 106.84 ± 5.24, 82.65 ± 3.25 against DDR1, DDR2, TRK-A, TRK-B, and TRK-C, respectively at 1.0 μM

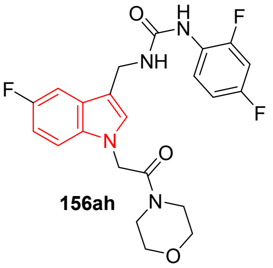

156ah

% control = 41.95 ± 0.69, 53.55 ± 2.31, 93.84 ± 1.50, 72.75 ± 2.68, 82.68 ± 3.36 against DDR1, DDR2, TRK-A, TRK-B, and TRK-C, respectively at 1.0 μM; IC_{50} = 0.62 ± 0.065, 0.45 ± 0.060 μM against DDR1 and DDR2, respectively; IC_{50} = 3.72 ± 0.54, 2.82 ± 0.31, 1.98 ± 0.22 μM against A549, SPC-A-1 and H1975, respectively

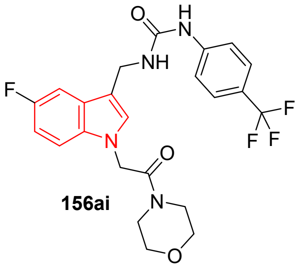

156ai

% control = 89.17 ± 4.39, 90.16 ± 1.50, 77.52 ± 5.07, 72.25 ± 1.96, 98.33 ± 7.03 against DDR1, DDR2, TRK-A, TRK-B, and TRK-C, respectively at 1.0 μM

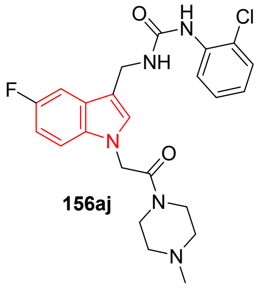

156aj

% control = 64.86 ± 2.83, 73.48 ± 3.00, 81.81 ± 0.66, 89.63 ± 4.01, 90.16 ± 2.60 against DDR1, DDR2, TRK-A, TRK-B, and TRK-C, respectively at 1.0 μM

Fig. S24 (continued). Kinase inhibitory (% control at 1.0 μM) and antiproliferation (μM ± SD) properties of the synthesized indole-containing compounds linked to urea function **156** and Dasatinib (reference standard).

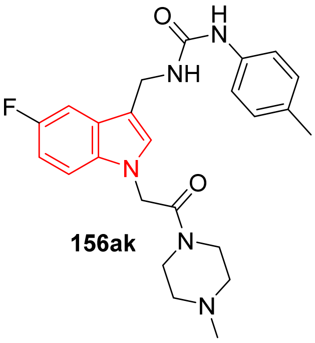

156ak

% control = 101.3 ± 7.83, 78.17 ± 2.78, 91.67 ± 1.92, 90.38 ± 3.94, 103.6 ± 3.17 against DDR1, DDR2, TRK-A, TRK-B, and TRK-C, respectively at 1.0 µM

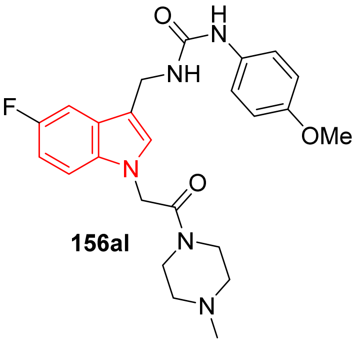

156al

% control = 89.17 ± 6.51, 73.48 ± 4.18, 79.98 ± 4.27, 103.56 ± 3.74, 105.6 ± 3.59 against DDR1, DDR2, TRK-A, TRK-B, and TRK-C, respectively at 1.0 µM

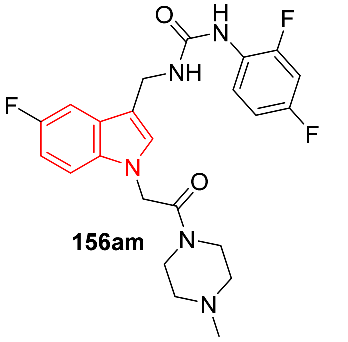

156am

% control = 12.16 ± 0.79, 13.67 ± 0.88, 84.93 ± 4.61, 106.4 ± 3.15, 78.64 ± 2.44 against DDR1, DDR2, TRK-A, TRK-B, and TRK-C, respectively at 1.0 µM; IC_{50} = 0.045 ± 0.006, 0.126 ± 0.038 µM against DDR1 and DDR2, respectively; IC_{50} = 1.84 ± 0.333, 3.51 ± 0.54, 1.87 ± 0.19 µM against A549, SPC-A-1 and H1975, respectively

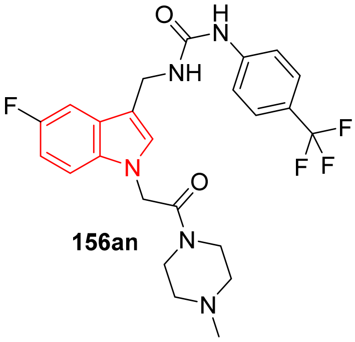

156an

% control = 98.61 ± 2.28, 74.21 ± 1.72, 81.45 ± 3.36, 105.3 ± 3.70, 93.73 ± 2.49 against DDR1, DDR2, TRK-A, TRK-B, and TRK-C, respectively at 1.0 µM

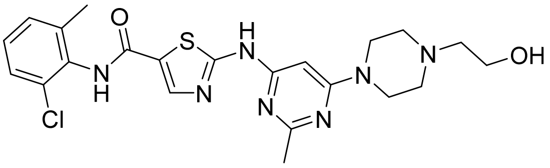

Dasatinib

IC_{50} = 0.026 ± 0.004, 0.054 ± 0.011 µM against DDR1 and DDR2, respectively; IC_{50} = 2.55 ± 0.22, 2.46 ± 0.32, 1.26 ± 0.07 µM against A549, SPC-A-1 and H1975, respectively

Fig. S24 (continued). Kinase inhibitory (% control at 1.0 µM) and antiproliferation (µM ± SD) properties of the synthesized indole-containing compounds linked to urea function **156** and Dasatinib (reference standard).

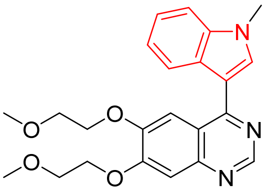

160a

IC_{50} = 9.3 nM against EGFR[L858R]

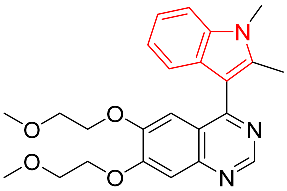

160b

IC_{50} = 81 nM against EGFR[L858R]

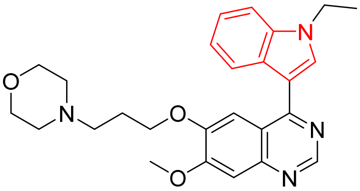

161a

IC_{50} = 6.5 nM against EGFR[L858R]

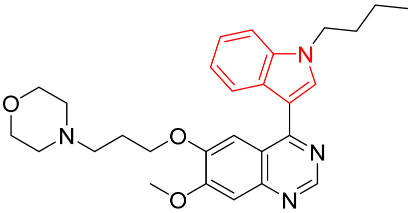

161b

IC_{50} = 3.0 nM against EGFR[L858R]

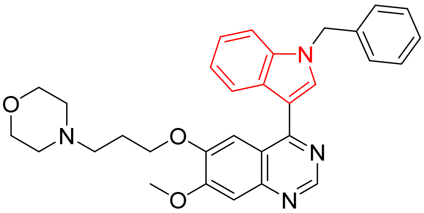

161c

IC_{50} = 2.8 nM against EGFR[L858R]

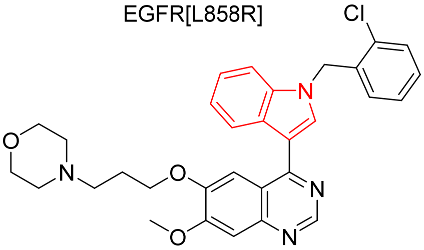

161d

IC_{50} = 3.0 nM against EGFR[L858R]

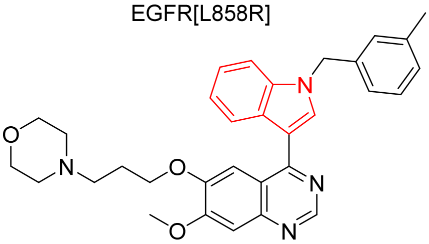

161e

IC_{50} = 2.8 nM against EGFR[L858R]

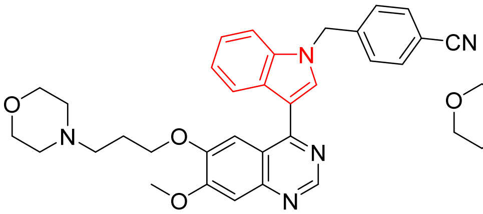

161f

IC_{50} = 5.6 nM against EGFR[L858R]

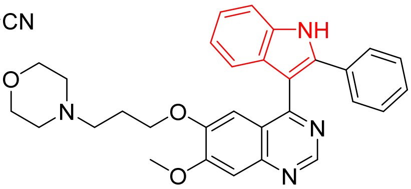

161g

IC_{50} = 0.67 nM against EGFR[L858R]

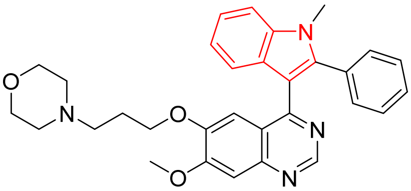

161h

IC_{50} = 1.3 nM against EGFR[L858R]

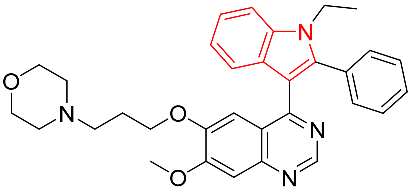

161i

IC_{50} = 1.9 nM against EGFR[L858R]

Fig. S25. enzymatic inhibitory properties of synthesized agents **160**, **161** and Gefitinib against EGFR[L858R].

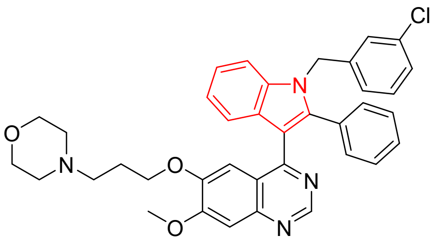

161j

IC_{50} = 4.9 nM against EGFR[L858R]

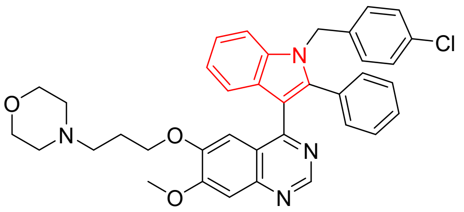

161k

IC_{50} = 7.7 nM against EGFR[L858R]

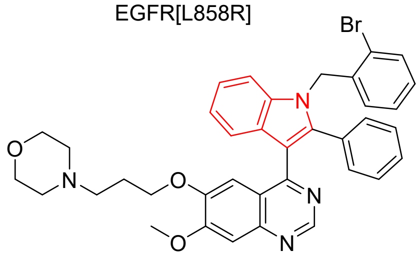

161l

IC_{50} = 7.0 nM against EGFR[L858R]

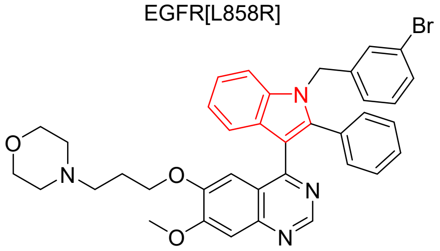

161m

IC_{50} = 6.5 nM against EGFR[L858R]

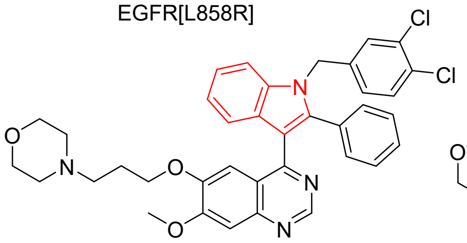

161n

IC_{50} = 22 nM against EGFR[L858R]

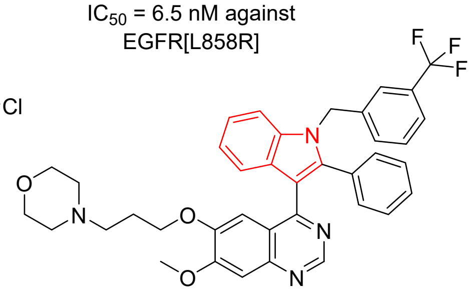

161o

IC_{50} = 20 nM against EGFR[L858R]

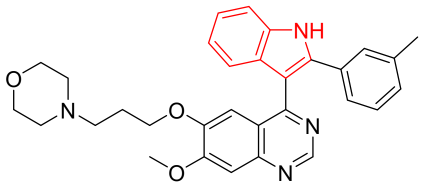

161p

IC_{50} = 1.7 nM against EGFR[L858R]

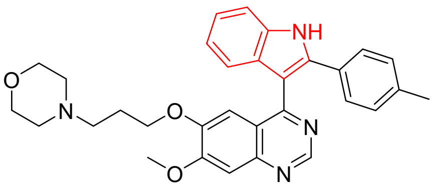

161q

IC_{50} = 5.3 nM against EGFR[L858R]

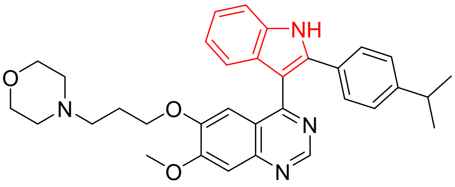

161r

IC_{50} = >100 nM against EGFR[L858R]

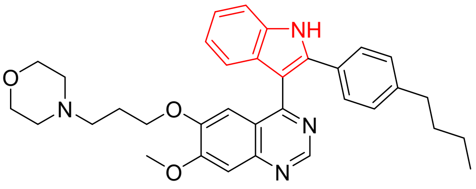

161s

IC_{50} = >100 nM against EGFR[L858R]

Fig. S25 (continued). enzymatic inhibitory properties of synthesized **160**, **161** and Gefitinib against EGFR[L858R].

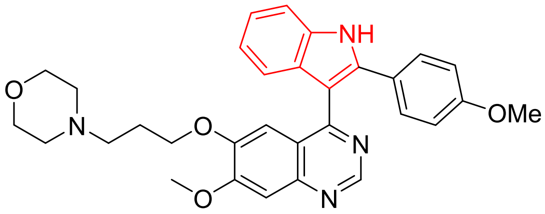

161t

IC_{50} = >100 nM against
EGFR[L858R]

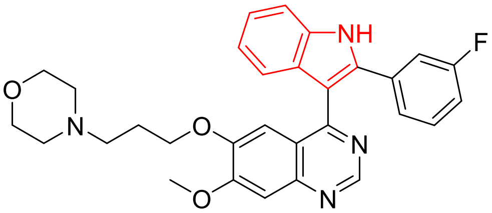

161u

IC_{50} = 1.3 nM against
EGFR[L858R]

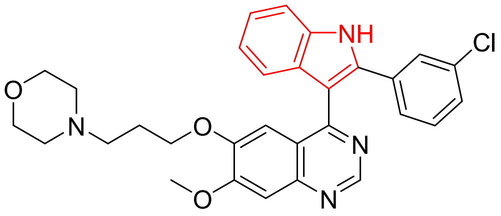

161v

IC_{50} = 1.5 nM against
EGFR[L858R]

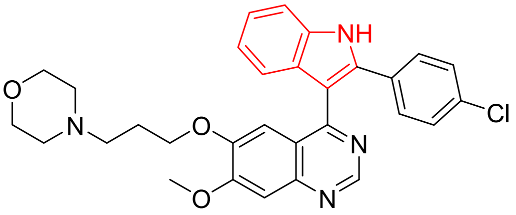

161w

IC_{50} = 9.4 nM against
EGFR[L858R]

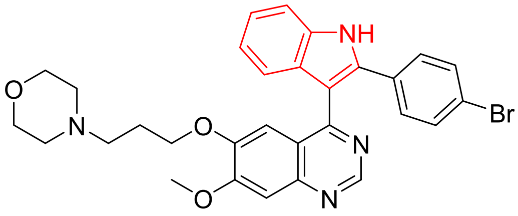

161w

IC_{50} = 3.0 nM against
EGFR[L858R]

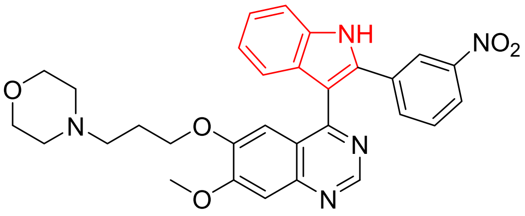

161x

IC_{50} = 4.1 nM against
EGFR[L858R]

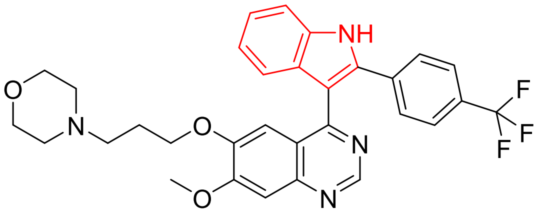

161y

IC_{50} = 84 nM against
EGFR[L858R]

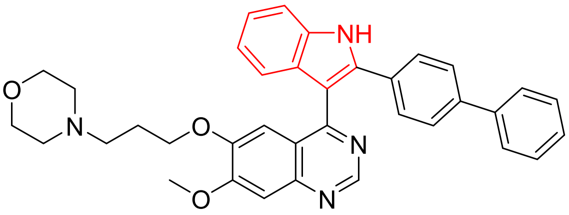

161z

IC_{50} = >100 nM against
EGFR[L858R]

Fig. S25 (continued). enzymatic inhibitory properties of synthesized **160**, **161** and Gefitinib against EGFR[L858R].

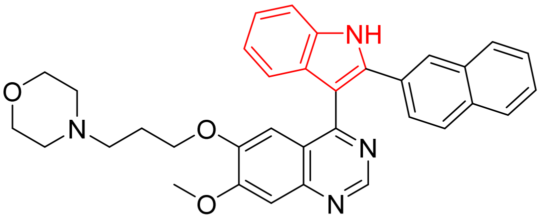

161aa

IC_{50} = 39 nM against EGFR[L858R]

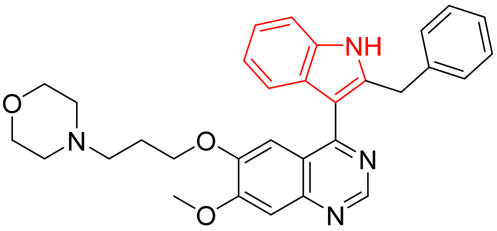

161ab

IC_{50} = 11 nM against EGFR[L858R]

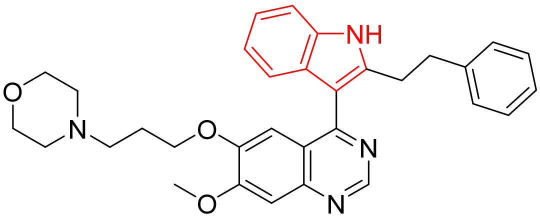

161ac

IC_{50} = >100 nM against EGFR[L858R]

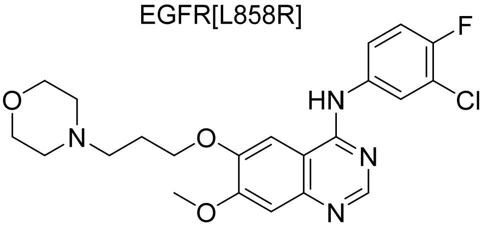

Gefitinib

IC_{50} = 1.3 nM against EGFR[L858R]

Fig. S25 (continued). enzymatic inhibitory properties of synthesized **160**, **161** and Gefitinib against EGFR[L858R].

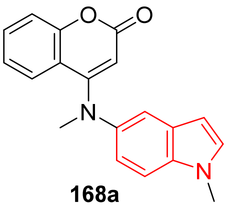

168a

IC_{50} = 1.79 ± 0.10 x 10^{-3}, >50, 0.04 ± 0.00 µM against A549, HepG2 and MCF-7, respectively

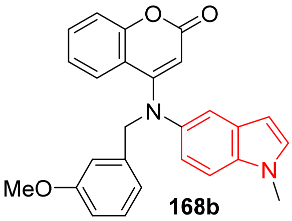

168b

IC_{50} = 11.81 ± 0.52, >50, 26.43 ± 0.43 µM against A549, HepG2 and MCF-7, respectively

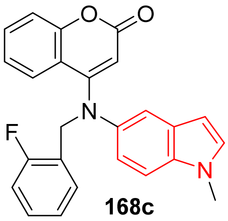

168c

IC_{50} = 4.32 ± 0.22, 26.12 ± 0.95, 9.46 ± 0.40 µM against A549, HepG2 and MCF-7, respectively

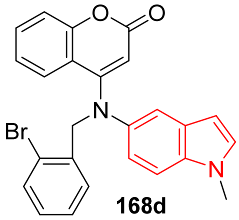

168d

IC_{50} = >30, >50, >30 µM against A549, HepG2 and MCF-7, respectively

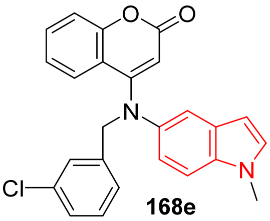

168e

IC_{50} = >30, >50, >30 µM against A549, HepG2 and MCF-7, respectively

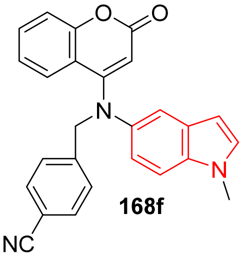

168f

IC_{50} = >30, >50, >30 µM against A549, HepG2 and MCF-7, respectively

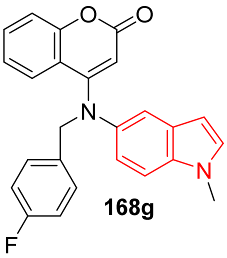

168g

IC_{50} = 7.33 ± 0.15, 34.46 ± 0.83, >30 µM against A549, HepG2 and MCF-7, respectively

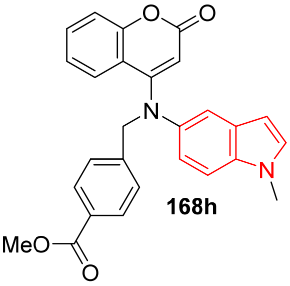

168h

IC_{50} = 15.81 ± 0.26, >50, 11.16 ± 0.36 µM against A549, HepG2 and MCF-7, respectively

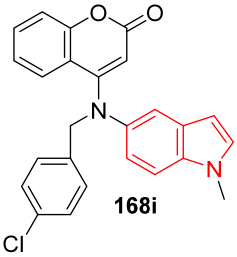

168i

IC_{50} = 17.26 ± 0.25, >50, 19.60 ± 0.30 µM against A549, HepG2 and MCF-7, respectively

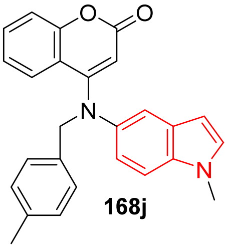

168j

IC_{50} = 5.24 ± 0.12, 40.55 ± 0.17, 25.92 ± 0.29 µM against A549, HepG2 and MCF-7, respectively

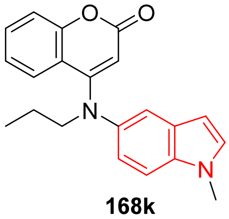

168k

IC_{50} = 0.08 ± 0.00, >50, 0.16± 0.01 µM against A549, HepG2 and MCF-7, respectively

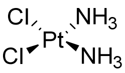

Cisplatin

IC_{50} = 5.62 ± 0.24, 10.81 ± 0.43, 5.06 ± 0.19 µM against A549, HepG2 and MCF-7, respectively

Fig. S26. Antiproliferation properties (µM ± SD) of the synthesized coumarin-indole conjugates **168**, Cisplatin and Colchicine.

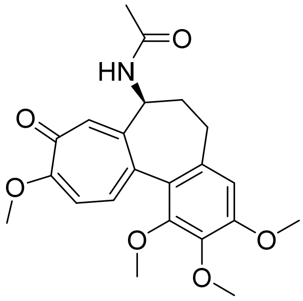

Colchicine

IC_{50} = 0.01 ± 0.00, 8.36 ± 0.28, 0.04 ± 0.00 μM against A549, HepG2 and MCF-7, respectively

Fig. S26 (continued). Antiproliferation properties (μM ± SD) of the synthesized coumarin-indole conjugates **168**, Cisplatin and Colchicine.

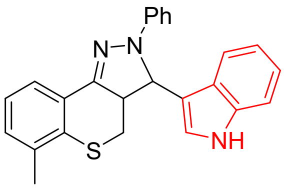

173a

IC_{50} = 68.02 ± 0.99, 36.39 ± 1.04, 21.93 ± 1.35, >120, 158.2 ± 5.91 µM against MCF-7, Hela, MGC-803, Bel-7404, and L929 cell lines, respectively

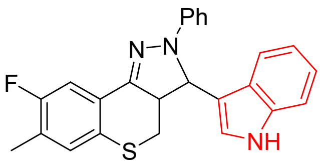

173b

IC_{50} = 47.75 ± 1.35, 41.10 ± 0.98, 27.80 ± 0.94, 61.88 ± 2.31, 149.5 ± 1.75 µM against MCF-7, Hela, MGC-803, Bel-7404, and L929 cell lines, respectively

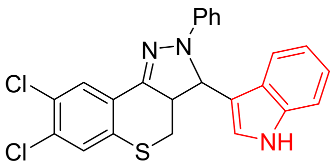

173c

IC_{50} = 72.79 ± 4.11, 27.36 ± 2.14, 27.06 ± 1.03, >120, 164.3 ± 2.60 µM against MCF-7, Hela, MGC-803, Bel-7404, and L929 cell lines, respectively

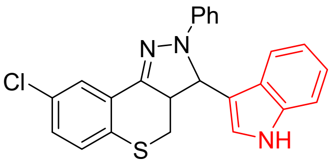

173d

IC_{50} = 26.09 ± 0.88, 38.17 ± 1.06, 15.43 ± 1.07, 42.92 ± 2.05, 159.9 ± 2.72 µM against MCF-7, Hela, MGC-803, Bel-7404, and L929 cell lines, respectively

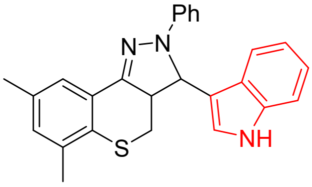

173e

IC_{50} = 26.98 ± 2.01, 29.25 ± 3.04, 26.53 ± 0.99, 23.73 ± 0.74, 133.9 ± 2.25 µM against MCF-7, Hela, MGC-803, Bel-7404, and L929 cell lines, respectively

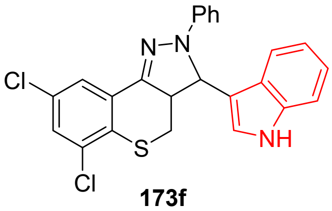

173f

IC_{50} = 25.60 ± 0.55, 24.96 ± 0.74, 20.54 ± 1.01, 21.02 ± 2.36, 105.1 ± 1.30 µM against MCF-7, Hela, MGC-803, Bel-7404, and L929 cell lines, respectively

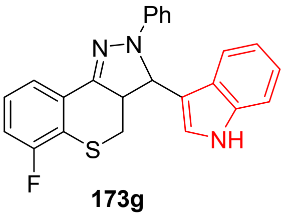

173g

IC_{50} = 35.69 ± 1.05, 31.61 ± 4.13, 31.13 ± 3.11, 36.25 ± 0.91, 158.3 ± 2.42 µM against MCF-7, Hela, MGC-803, Bel-7404, and L929 cell lines, respectively

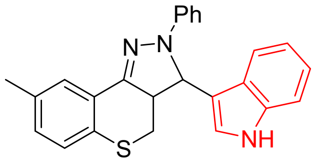

173h

IC_{50} = 37.96 ± 2.10, 36.97 ± 1.43, 28.04 ± 2.11, 57.34 ± 1.25, 189.3 ± 2.18 µM against MCF-7, Hela, MGC-803, Bel-7404, and L929 cell lines, respectively

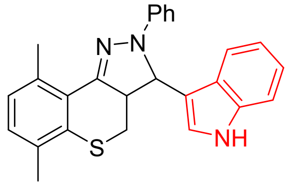

173i

IC_{50} = 37.68 ± 0.98, 39.66 ± 2.10, 35.56 ± 3.17, 37.47 ± 2.43, 183.3 ± 1.46 µM against MCF-7, Hela, MGC-803, Bel-7404, and L929 cell lines, respectively

Fig. S27. Antiroliferation properties (µM ± SD) of thiochromeno[4,3-*c*]pyrazole-indole conjugates **173**, Etoposide and Cisplatin (standard references).

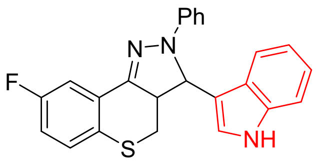

173j

IC_{50} = 37.84 ± 1.05, 50.04 ± 5.32, 39.27 ± 4.06, 50.24 ± 1.46, 174.7 ± 2.41 μM against MCF-7, Hela, MGC-803, Bel-7404, and L929 cell lines, respectively

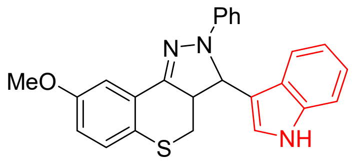

173k

IC_{50} = >120, >120, 36.38 ± 1.68, >120, 120.5 ± 2.13 μM against MCF-7, Hela, MGC-803, Bel-7404, and L929 cell lines, respectively

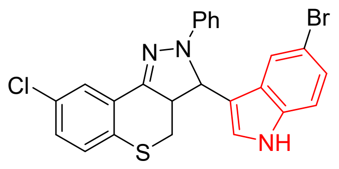

173l

IC_{50} = 37.14 ± 1.21, 35.08 ± 2.06, 29.26 ± 3.15, 32.14 ± 0.45, 153.5 ± 2.59 μM against MCF-7, Hela, MGC-803, Bel-7404, and L929 cell lines, respectively

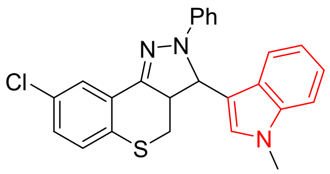

173m

IC_{50} = 51.12 ± 2.38, 48.67 ± 1.48, 33.13 ± 2.02, 36.17 ± 1.33, 261.8 ± 4.2 μM against MCF-7, Hela, MGC-803, Bel-7404, and L929 cell lines, respectively

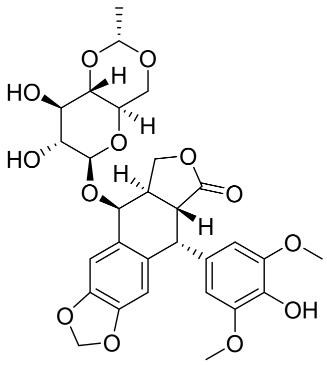

Etoposide

IC_{50} = 1.45 ± 0.65, 5.35 ± 0.47, 11.18 ± 0.33, 7.76 ± 0.46, 80.36 ± 1.91 μM against MCF-7, Hela, MGC-803, Bel-7404, and L929 cell lines, respectively

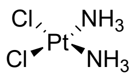

Cisplatin

IC_{50} = 0.86 ± 0.75, 3.153 ± 0.89, 11.38 ± 0.91, 37.47 ± 1.05, 31.69 ± 1.50 μM against MCF-7, Hela, MGC-803, Bel-7404, and L929 cell lines, respectively

Fig. S27 (continued). Antiroliferation properties (μM ± SD) of thiochromeno[4,3-*c*]pyrazole-indole conjugates **173**, Etoposide and Cisplatin (standard references).

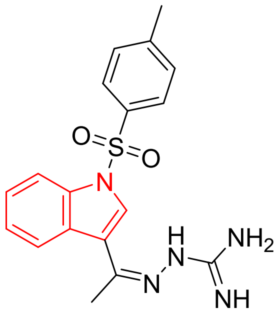

175a

IC_{50} = 2.68, 2.38, 52.99 μM against A590, SGC7901 and HEK 293T, respectively

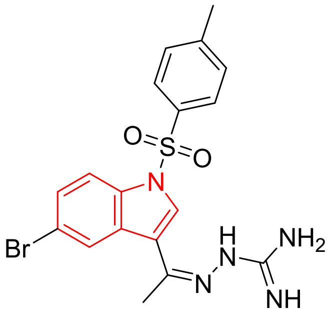

175b

IC_{50} = 4.44, 1.51, 56.39 μM against A590, SGC7901 and HEK 293T, respectively

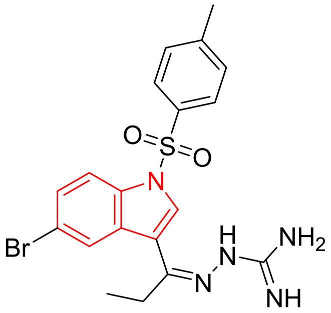

175c

IC_{50} = 2.33, 3.92, 57.64 μM against A590, SGC7901 and HEK 293T, respectively

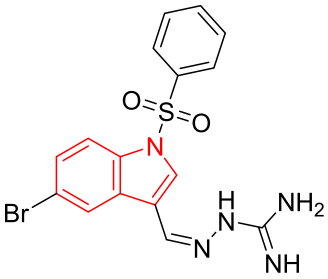

175d

IC_{50} = 14.67, 3.06, 71.61 μM against A590, SGC7901 and HEK 293T, respectively

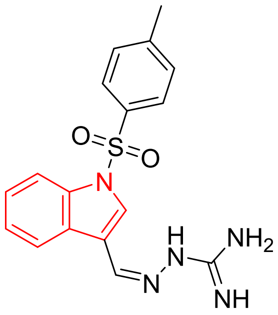

175d

IC_{50} = 12.22, 2.12, 86.66 μM against A590, SGC7901 and HEK 293T, respectively

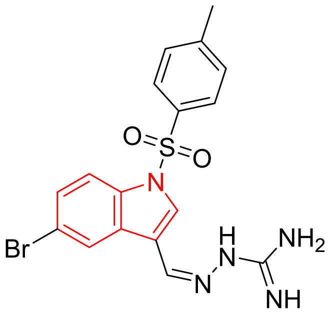

175e

IC_{50} = 15.12, 4.00, 73.46 μM against A590, SGC7901 and HEK 293T, respectively

Fig. S28. Antiproliferation properties of the synthesized indolylhydrazine-1-carboximidamide **175.**

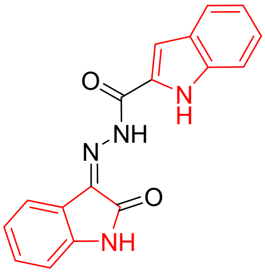

179a

IC_{50} = 19.74 ± 1.95, 22.73 ± 5.71, 12.98 ± 2.46 μM against HT-29, ZR-75 and A-549, respectively

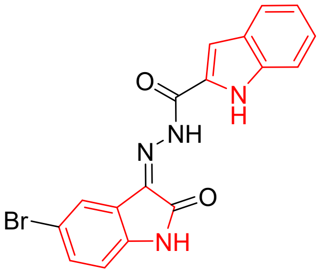

179b

IC_{50} = 21.93 ± 1.29, 21.92 ± 4.93, 16.01 ± 2.26 μM against HT-29, ZR-75 and A-549, respectively

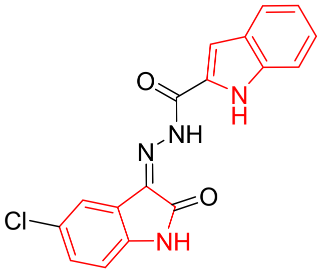

179c

IC_{50} = >30, 18.01 ± 18.47, 19.68 ± 5.20 μM against HT-29, ZR-75 and A-549, respectively

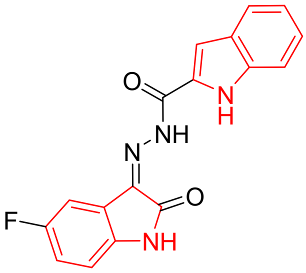

179d

IC_{50} = >30, >30, >30 μM against HT-29, ZR-75 and A-549, respectively

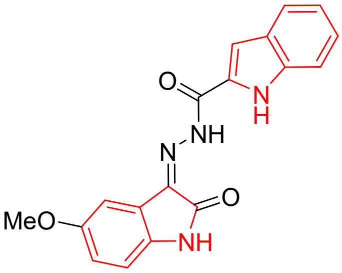

179e

IC_{50} = >30, 17.87 ± 7.24, 6.68 ± 1.52 μM against HT-29, ZR-75 and A-549, respectively

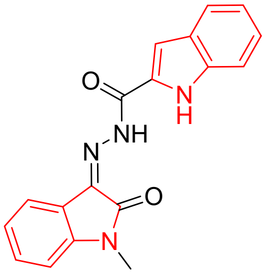

179f

IC_{50} = 12.88 ± 1.03, 1.53 ± 31.5, 4.04 ± 0.83 μM against HT-29, ZR-75 and A-549, respectively

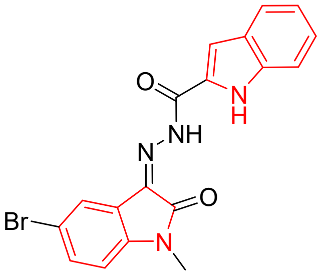

179g

IC_{50} = 3.68 ± 0.29, 1.01 ± 3.28, 1.49 ± 0.15 μM against HT-29, ZR-75 and A-549, respectively

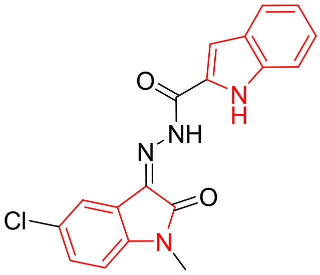

179h

IC_{50} = 5.67 ± 0.43, 0.84 ± 7.65, 2.13 ± 0.42 μM against HT-29, ZR-75 and A-549, respectively

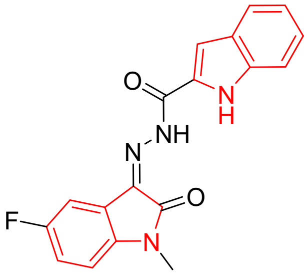

179i

IC_{50} = 8.92 ± 0.67, 4.31 ± 7.98, 2.82 ± 0.30 μM against HT-29, ZR-75 and A-549, respectively

Fig. S29. Antiproliferation properties of the synthesized 2-oxo-3-indolylidene-2-indolecarbohydrazones **179** and Sunitinib.

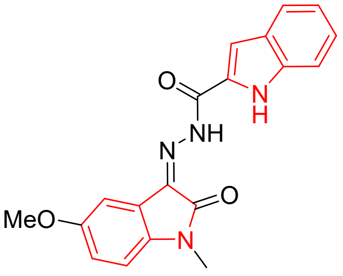

179j

IC_{50} = 5.73 ± 0.67,
1.48 ± 7.06, 1.93 ± 0.27 μM
against HT-29, ZR-75
and A-549, respectively

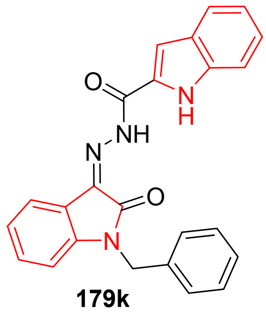

179k

IC_{50} = 6.19 ± 1.58,
1.12 ± 3.05, 1.06 ± 0.37 μM
against HT-29, ZR-75
and A-549, respectively

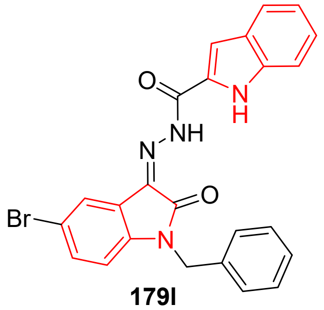

179l

IC_{50} = 2.26 ± 0.33,
0.70 ± 12.04, 0.65 ± 0.12 μM
against HT-29, ZR-75
and A-549, respectively

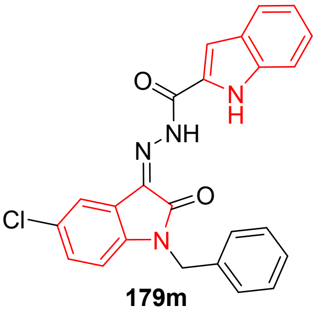

179m

IC_{50} = 2.02 ± 0.36,
0.74 ± 0.88, 0.76 ± 0.12 μM
against HT-29, ZR-75
and A-549, respectively

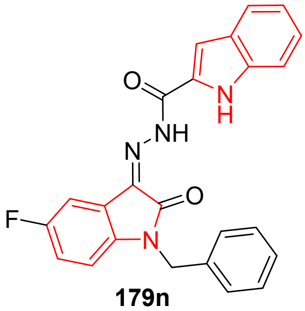

179n

IC_{50} = 3.50 ± 0.49,
2.10 ± 3.15, 1.41 ± 0.21 μM
against HT-29, ZR-75
and A-549, respectively

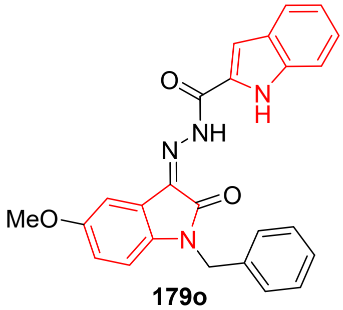

179o

IC_{50} = 2.28 ± 0.22,
0.86 ± 0.76, 0.64 ± 0.24 μM
against HT-29, ZR-75
and A-549, respectively

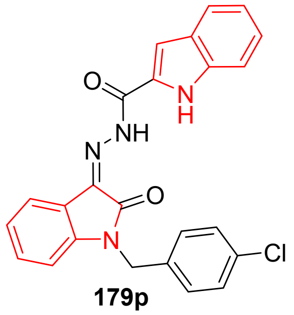

179p

IC_{50} = 2.64 ± 0.27,
1.30 ± 0.58, 1.05 ± 0.15 μM
against HT-29, ZR-75
and A-549, respectively

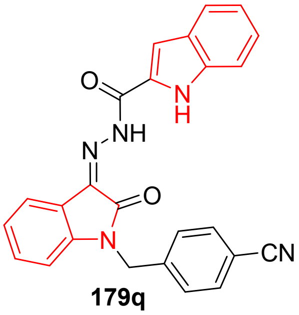

179q

IC_{50} = 8.11 ± 1.27,
1.51 ± 5.03, 2.13 ± 0.49 μM
against HT-29, ZR-75
and A-549, respectively

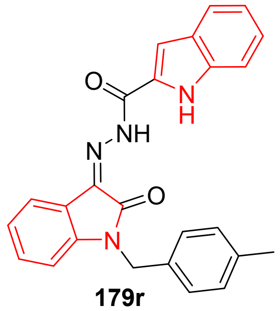

179r

IC_{50} = 2.45 ± 0.32,
1.28 ± 12.44, 0.64 ± 0.61 μM
against HT-29, ZR-75
and A-549, respectively

Fig. S29 (cintinued). Antiproliferation properties of the synthesized 2-oxo-3-indolylidene-2-indolecarbohydrazones **179** and Sunitinib.

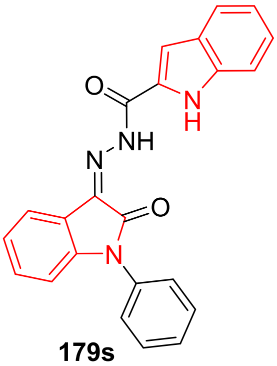

179s

IC_{50} = 6.34 ± 0.76,
1.32 ± 2.89, 2.41 ± 0.29 μM
against HT-29, ZR-75
and A-549, respectively

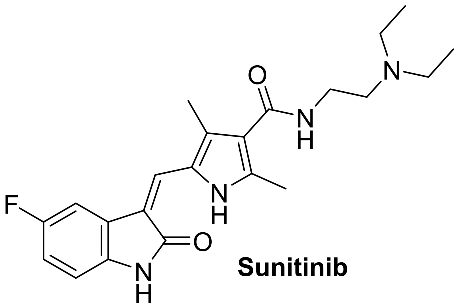

Sunitinib

IC_{50} = 10.14 ± 0.8,
8.31 ± 2.4, 5.87 ± 0.3 μM
against HT-29, ZR-75
and A-549, respectively

Fig. S29 (cintinued). Antiproliferation properties of the synthesized 2-oxo-3-indolylidene-2-indolecarbohydrazones **179** and Sunitinib.

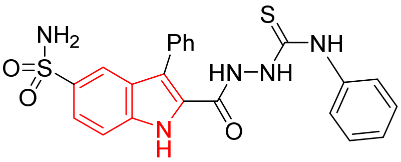

182a

IC_{50} = 132.28 ± 26.06, 80.04 ± 18.01 μM against HT-29, and CCD-986Sk, respectively; K_i = 946.1, 375.3, 28.6, 54.2 nM against hCA I, hCA II, hCA XI, and hCA XII, respectively

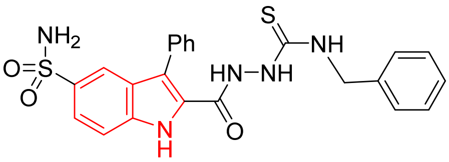

182b

IC_{50} = 153.55 ± 32.95, 102.73 ± 13.86 μM against HT-29, and CCD-986Sk, respectively; K_i = 1809.5, 525.2, 112.5, 88.5 nM against hCA I, hCA II, hCA IX, and hCA XII, respectively

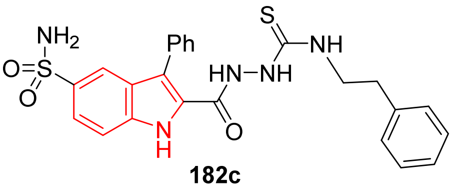

182c

IC_{50} = 454.04 ± 122.22, 62.73 ± 9.25 μM against HT-29, and CCD-986Sk, respectively; K_i = 5566.3, 778.5, 250.5, 306.2 nM against hCA I, hCA II, hCA IX, and hCA XII, respectively

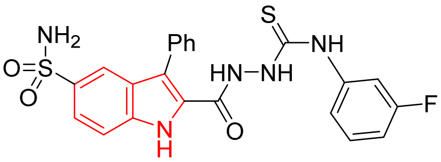

182d

IC_{50} = 111.81 ± 13,22, 82.98 ± 16.20 μM against HT-29, and CCD-986Sk, respectively; K_i = 6239.3, 284.4, 45.6, 130.3 nM against hCA I, hCA II, hCA IX, and hCA XII, respectively

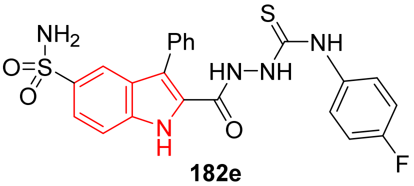

182e

IC_{50} = 53.32 ± 7.74, 74.64 ± 14.15 μM against HT-29, and CCD-986Sk, respectively; K_i = 4162.4, 211.5, 31.0, 87.0 nM against hCA I, hCA II, hCA IX, and hCA XII, respectively

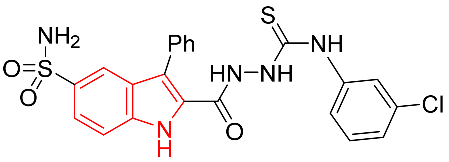

182f

IC_{50} = 115.69 ± 30.88, 57.69 ± 6.05 μM against HT-29, and CCD-986Sk, respectively; K_i = 4789.8, 194.6, 34.8, 61.4 nM against hCA I, hCA II, hCA IX, and hCA XII, respectively

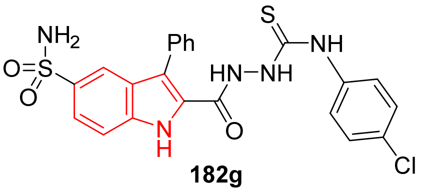

182g

IC_{50} = 76.69 ± 19.31, 55.55 ± 11.71 μM against HT-29, and CCD-986Sk, respectively; K_i = 3627.0, 159.5, 25.7, 56.1 nM against hCA I, hCA II, hCA IX, and hCA XII, respectively

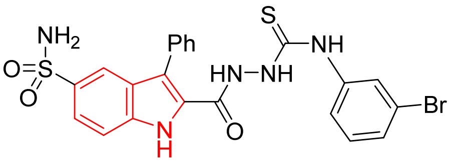

182h

IC_{50} = 192.77 ± 69.81, 60.67 ± 5.52 μM against HT-29, and CCD-986Sk, respectively; K_i = 3977.2, 539.4, 11.7, 46.2 nM against hCA I, hCA II, hCA IX, and hCA XII, respectively

Fig. S30. Antiproliferation and carbonic anhydrase inhibitory properties of 1-(indole-2-carbonyl)thiosemicarbazides incolaborating sulfonamide group **182** and standard references (Doxorubicin and Acetazolamide "AAZ").

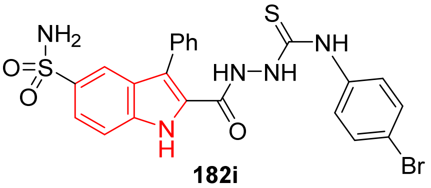

182i

IC_{50} = 71.70 ± 15.97, 68.25 ± 15.13 μM against HT-29, and CCD-986Sk, respectively; K_i = 2651.5, 421.4, 9.1, 31.3 nM against hCA I, hCA II, hCA IX, and hCA XII, respectively

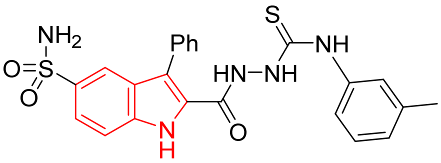

182j

IC_{50} = 200.12 ± 59.51, 68.28 ± 12.24 μM against HT-29, and CCD-986Sk, respectively; K_i = 2833.3, 849.9, 94.6, 80.0 nM against hCA I, hCA II, hCA IX, and hCA XII, respectively

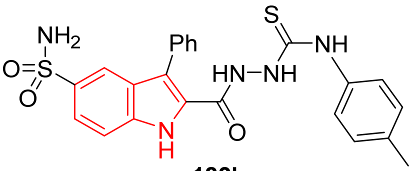

182k

IC_{50} = 94.06 ± 18.52, 74.95 ± 15.13 μM against HT-29, and CCD-986Sk, respectively; K_i = 2137.0, 744.4, 63.4, 87.1 nM against hCA I, hCA II, hCA IX, and hCA XII, respectively

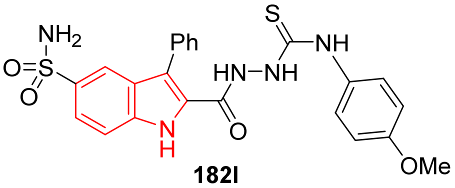

182l

IC_{50} = < 1000, 88.49 ± 10.34 μM against HT-29, and CCD-986Sk, respectively; K_i = 3448.8, 532.9, 131.9, 93.9 nM against hCA I, hCA II, hCA IX, and hCA XII, respectively

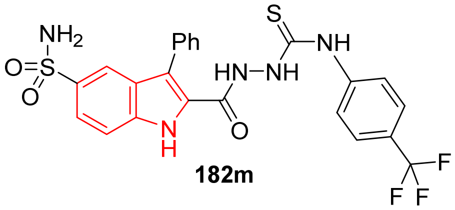

182m

IC_{50} = 145.58 ± 40.85, 96.67 ± 19.95 μM against HT-29, and CCD-986Sk, respectively; K_i = 4071.5, 500.4, 30.8, 75.2 nM against hCA I, hCA II, hCA IX, and hCA XII, respectively

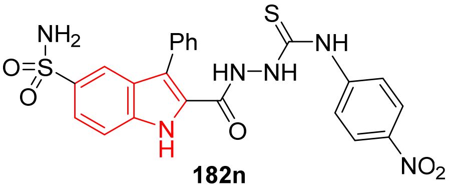

182n

IC_{50} = 183.17 ± 53.37, 134.14 ± 38.10 μM against HT-29, and CCD-986Sk, respectively; K_i = 614.6, 63.2, 12.7, 21.8 nM against hCA I, hCA II, hCA IX, and hCA XII, respectively

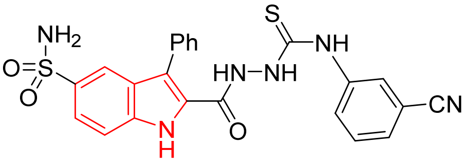

182o

IC_{50} = 215.68 ± 46.15, 119.12 ± 12.58 μM against HT-29, and CCD-986Sk, respectively; K_i = 475.0, 73.5, 79.2, 19.3 nM against hCA I, hCA II, hCA IX, and hCA XII, respectively

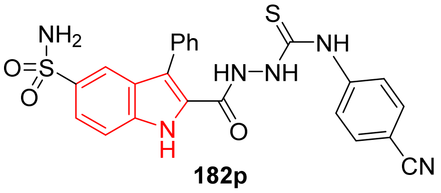

182p

IC_{50} = 364.95 ± 84.84, 111.81 ± 33.02 μM against HT-29, and CCD-986Sk, respectively; K_i = 5719.5, 284.4, 191.3, 42.4 nM against hCA I, hCA II, hCA IX, and hCA XII, respectively

Fig. S30 (continued). Antiproliferation and carbonic anhydrase inhibitory properties of 1-(indole-2-carbonyl)thiosemicarbazides incolaborating sulfonamide group **182** and standard references (Doxorubicin and Acetazolamide "AAZ").

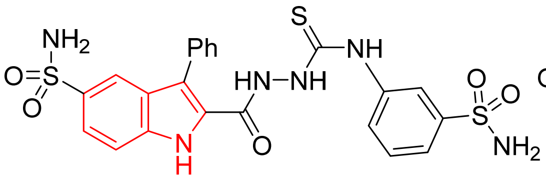

182q

IC_{50} = 508.05 ± 130.77, 155.63 ± 16.97 µM against HT-29, and CCD-986Sk, respectively; K_i = 78.7, 38.0, 2.1, 0.69 nM against hCA I, hCA II, hCA IX, and hCA XII, respectively

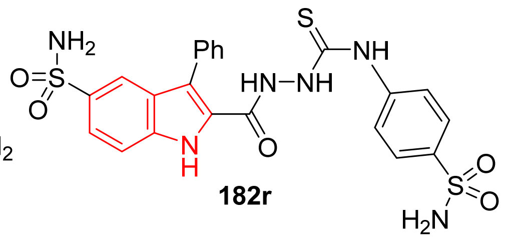

182r

IC_{50} = 407.14 ± 103.07, 322.15 ± 43.69 µM against HT-29, and CCD-986Sk, respectively; K_i = 75.9, 19.5, 1.4, 0.87 nM against hCA I, hCA II, hCA IX, and hCA XII, respectively

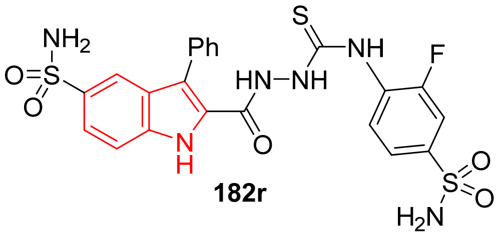

182r

IC_{50} = 335.46 ± 75.93, 132.50 ± 13.52 µM against HT-29, and CCD-986Sk, respectively; K_i = 101.9, 58.4, 12.7, 5.3 nM against hCA I, hCA II, hCA IX, and hCA XII, respectively

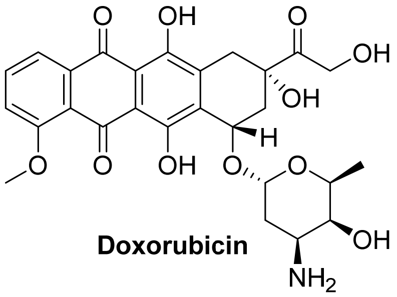

Doxorubicin

IC_{50} = 17.20 ± 3.80, 0.17 ± 0.05 µM against HT-29, and CCD-986Sk, respectively

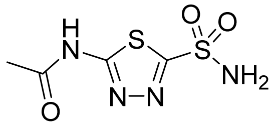

Acetazolamide (AAZ)

K_i = 250.0, 12.5, 25.0, 5.7 nM against hCA I, hCA II, hCA IX, and hCA XII, respectively

Fig. S30 (continued). Antiproliferation and carbonic anhydrase inhibitory properties of 1-(indole-2-carbonyl)thiosemicarbazides incolaborating sulfonamide group **182** and standard references (Doxorubicin and Acetazolamide "AAZ").

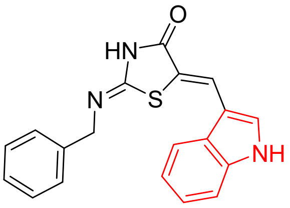

184a

IC_{50} = 22.19 ± 2.31, >30, 19.22 ± 0.11, >30, 25.72 ± 2.12 µM against A549, MDA-MB-231, HCT-29, HCT-15, and NCI-H460, respectively

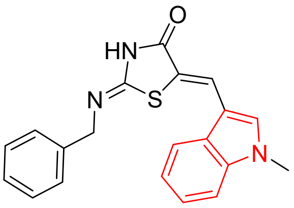

184b

IC_{50} = >30, >30, 25.10 ± 1.57, >30, >30 µM against A549, MDA-MB-231, HCT-29, HCT-15, and NCI-H460, respectively

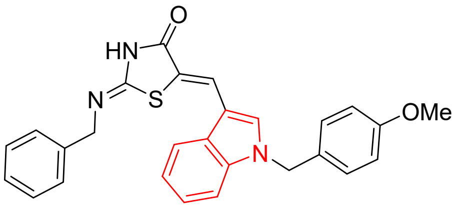

184c

IC_{50} = 17.34 ± 1.53, >30, 19.15 ± 0.12, 15.90 ± 0.55, >30 µM against A549, MDA-MB-231, HCT-29, HCT-15, and NCI-H460, respectively

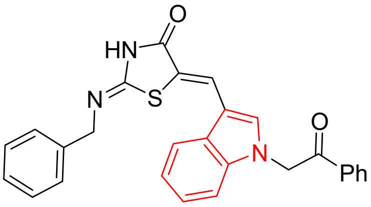

184d

IC_{50} = >30, >30, >30, >30, >30 µM against A549, MDA-MB-231, HCT-29, HCT-15, and NCI-H460, respectively

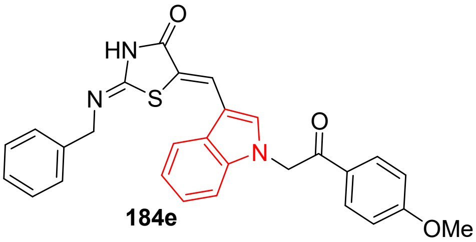

184e

IC_{50} = 18.11 ± 1.07, 26.72 ± 0.64, >30, 22.80 ± 2.13, 25.10 ± 1.57 µM against A549, MDA-MB-231, HCT-29, HCT-15, and NCI-H460, respectively

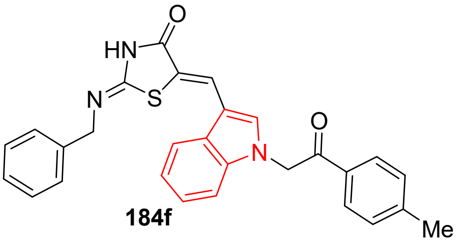

184f

IC_{50} = 20.38 ± 0.79, >30, 17.13 ± 1.20, >30, >30 µM against A549, MDA-MB-231, HCT-29, HCT-15, and NCI-H460, respectively

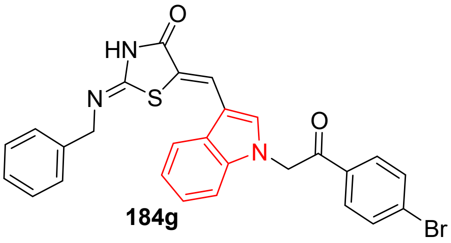

184g

IC_{50} = >30, >30, >30, >30, >30 µM against A549, MDA-MB-231, HCT-29, HCT-15, and NCI-H460, respectively

Fig. S31. Antiproliferation properties of thiazolidinone-indoles **184** and Podophyllotoxin.

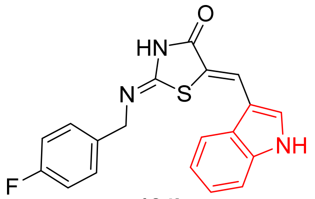

184h

IC$_{50}$ = 17.38 ± 0.90, 29.79 ± 1.42, 19.25 ± 0.98, >30, >30 μM against A549, MDA-MB-231, HCT-29, HCT-15, and NCI-H460, respectively

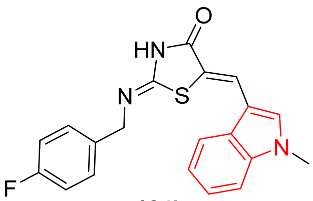

184i

IC$_{50}$ = >30, 25.09 ± 2.32, 29.09 ± 2.92, 24.13 ± 0.32, >30 μM against A549, MDA-MB-231, HCT-29, HCT-15, and NCI-H460, respectively

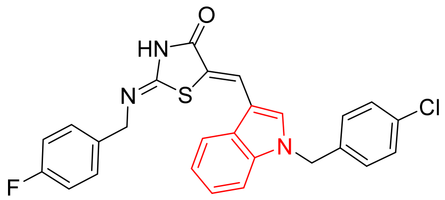

184j

IC$_{50}$ = 15.01 ± 2.16, 25.41 ± 2.52, 23.88 ± 1.09, >30, >30 μM against A549, MDA-MB-231, HCT-29, HCT-15, and NCI-H460, respectively

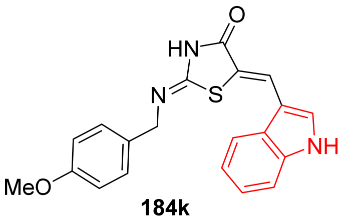

184k

IC$_{50}$ = 18.21 ± 1.09, 15.93 ± 2.40, >30, 18.97 ± 1.57, 20.03 ± 1.21 μM against A549, MDA-MB-231, HCT-29, HCT-15, and NCI-H460, respectively

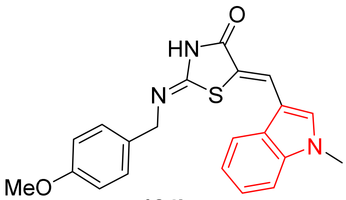

184l

IC$_{50}$ = >30, >30, >30, >30, >30 μM against A549, MDA-MB-231, HCT-29, HCT-15, and NCI-H460, respectively

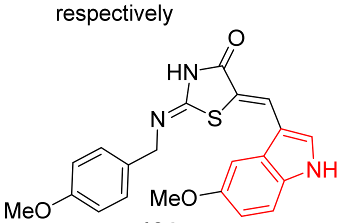

184m

IC$_{50}$ = 28.92 ± 1.75, 18.33 ± 0.51, 22.69 ± 2.02, >30, >30 μM against A549, MDA-MB-231, HCT-29, HCT-15, and NCI-H460, respectively

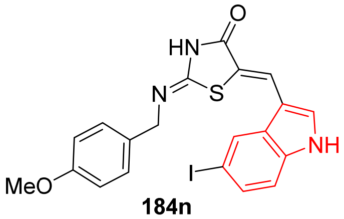

184n

IC$_{50}$ = >30, 26.09 ± 0.47, >30, 23.10 ± 0.31, >30 μM against A549, MDA-MB-231, HCT-29, HCT-15, and NCI-H460, respectively

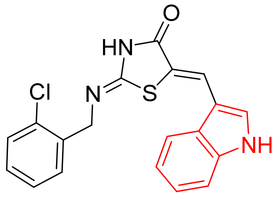

184o

IC$_{50}$ = >30, >30, >30, >30, >30 μM against A549, MDA-MB-231, HCT-29, HCT-15, and NCI-H460, respectively

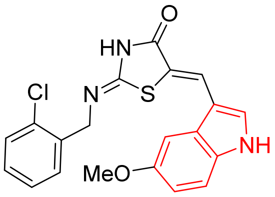

184p

IC$_{50}$ = >30, >30, >30, >30, 23.87 ± 1.06 μM against A549, MDA-MB-231, HCT-29, HCT-15, and NCI-H460, respectively

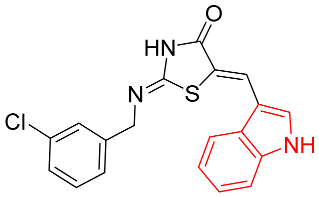

184q

IC$_{50}$ = >30, >30, >30, >30, >30 μM against A549, MDA-MB-231, HCT-29, HCT-15, and NCI-H460, respectively

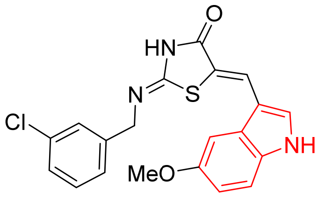

184r

IC$_{50}$ = 29.32 ± 1.85, >30, 21.94 ± 2.67, >30, >30 μM against A549, MDA-MB-231, HCT-29, HCT-15, and NCI-H460, respectively

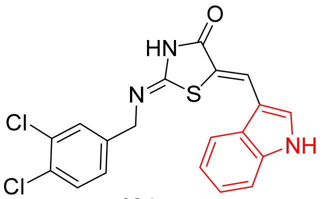

184s

IC$_{50}$ = >30, >30, >30, >30, >30 μM against A549, MDA-MB-231, HCT-29, HCT-15, and NCI-H460, respectively

Fig. S31 (continued). Antiproliferation properties of thiazolidinone-indoles **184** and Podophyllotoxin.

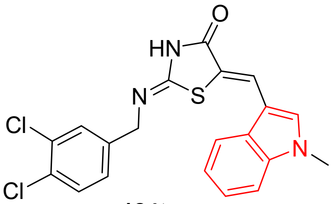

184t

IC_{50} = >30, >30, >30, >30, >30 μM against A549, MDA-MB-231, HCT-29, HCT-15, and NCI-H460, respectively

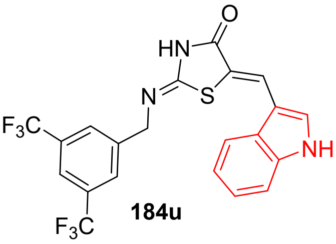

184u

IC_{50} = 10.83 ± 0.70, 16.99 ± 2.11, 19.52 ± 4.30, 15.10 ± 4.30, 4.02 ± 0.47 μM against A549, MDA-MB-231, HCT-29, HCT-15, and NCI-H460, respectively

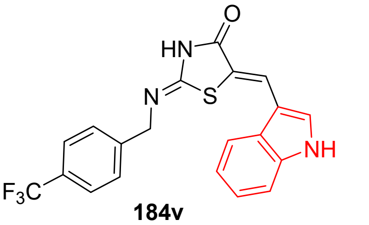

184v

IC_{50} = 19.32 ± 1.83, 22.59 ± 1.02, >30, >30, 20.38 ± 0.90 μM against A549, MDA-MB-231, HCT-29, HCT-15, and NCI-H460, respectively

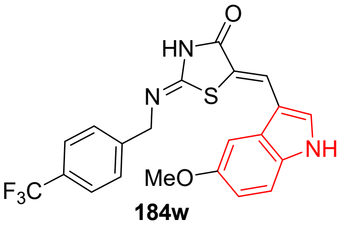

184w

IC_{50} = 26.59 ± 2.10, 18.76 ± 0.63, 28.96 ± 1.95, >30, >30 μM against A549, MDA-MB-231, HCT-29, HCT-15, and NCI-H460, respectively

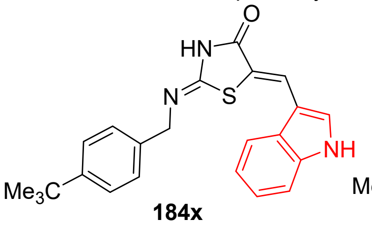

184x

IC_{50} = 16.18 ± 1.07, >30, >30, 20.09 ± 0.89, 24.32 ± 1.75 μM against A549, MDA-MB-231, HCT-29, HCT-15, and NCI-H460, respectively

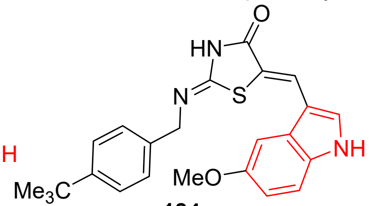

184y

IC_{50} = 24.03 ± 1.90, >30, 19.79 ± 2.37, >30, >30 μM against A549, MDA-MB-231, HCT-29, HCT-15, and NCI-H460, respectively

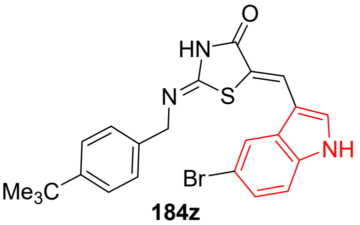

184z

IC_{50} = 8.68 ± 0.11, 19.73 ± 1.42, 14.05 ± 2.28, 3.93 ± 0.46, 17.19 ± 1.13 μM against A549, MDA-MB-231, HCT-29, HCT-15, and NCI-H460, respectively

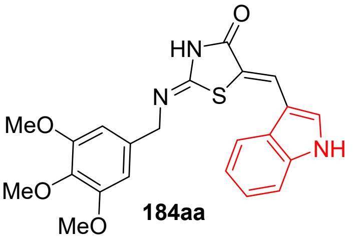

184aa

IC_{50} = 20.37 ± 0.79, 13.97 ± 1.78, 17.97 ± 0.90, 12.28 ± 0.30, 22.84 ± 10.00 μM against A549, MDA-MB-231, HCT-29, HCT-15, and NCI-H460, respectively

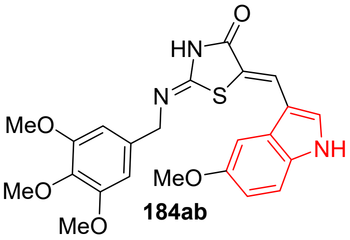

184ab

IC_{50} = 25.90 ± 2.11, >30, >30, 27.90 ± 0.75, 29.75 ± 2.01 μM against A549, MDA-MB-231, HCT-29, HCT-15, and NCI-H460, respectively

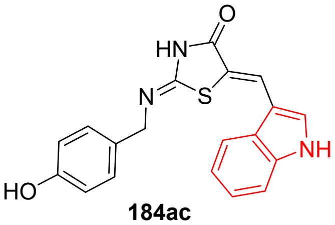

184ac

IC_{50} = >30, >30, >30, >30, >30 μM against A549, MDA-MB-231, HCT-29, HCT-15, and NCI-H460, respectively

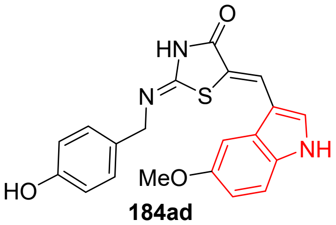

184ad

IC_{50} = >30, 28.68 ± 0.21, >30, 26.09 ± 0.70, >30 μM against A549, MDA-MB-231, HCT-29, HCT-15, and NCI-H460, respectively

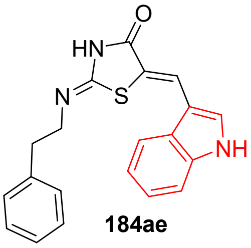

184ae

IC_{50} = 21.24 ± 1.90, >30, 27.09 ± 0.55, 23.76 ± 0.29, >30 μM against A549, MDA-MB-231, HCT-29, HCT-15, and NCI-H460, respectively

Fig. S31 (continued). Antiproliferation properties of thiazolidinone-indoles **184** and Podophyllotoxin.

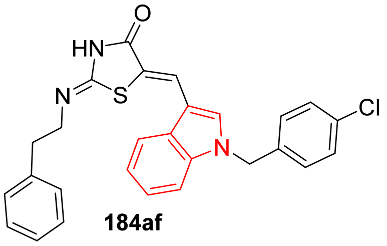

184af

IC_{50} = >30, >30, 19.54 ± 0.82, >30, 25.09 ± 2.03 μM against A549, MDA-MB-231, HCT-29, HCT-15, and NCI-H460, respectively

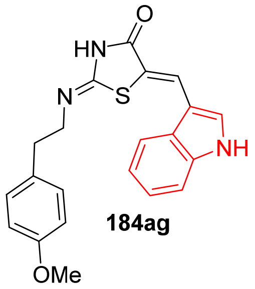

184ag

IC_{50} = 15.93 ± 2.11, 19.45 ± 2.28, 17.13 ± 5.01, 1.26 ± 0.17, 19.79 ± 6.49 μM against A549, MDA-MB-231, HCT-29, HCT-15, and NCI-H460, respectively

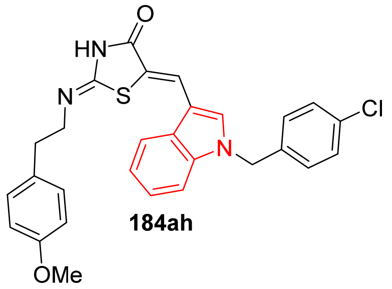

184ah

IC_{50} = 24.58 ± 2.49, >30, 20.01 ± 0.85, 15.29 ± 2.01, >30 μM against A549, MDA-MB-231, HCT-29, HCT-15, and NCI-H460, respectively

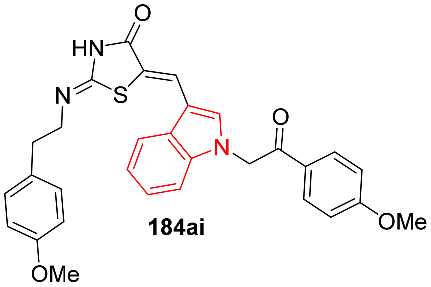

184ai

IC_{50} = 29.04 ± 0.66, 23.59 ± 2.84, >30, 29.17 ± 1.45, >30 μM against A549, MDA-MB-231, HCT-29, HCT-15, and NCI-H460, respectively

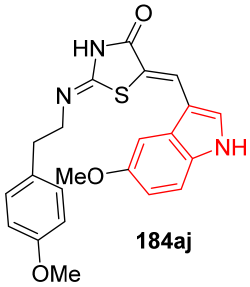

184aj

IC_{50} = 22.73 ± 1.27, >30, >30, 17.31 ± 1.02, 26.53 ± 2.17 μM against A549, MDA-MB-231, HCT-29, HCT-15, and NCI-H460, respectively

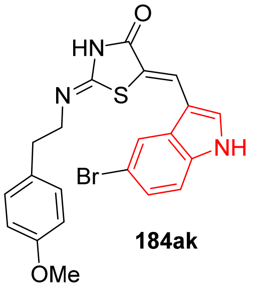

184ak

IC_{50} = 4.20 ± 0.02, 12.07 ± 0.91, 2.802 ± 0.02, 0.92 ± 0.12, 4.64 ± 1.86, 10.84 ± 0.94 μM against A549, MDA-MB-231, HCT-29, HCT-15, NCI-H460, and L132, respectively

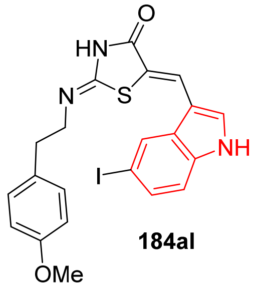

184al

IC_{50} = 5.44 ± 0.28, 25.47 ± 2.60, 4.85 ± 0.23, 18.13 ± 0.36, 6.93 ± 0.71 μM against A549, MDA-MB-231, HCT-29, HCT-15, and NCI-H460, respectively

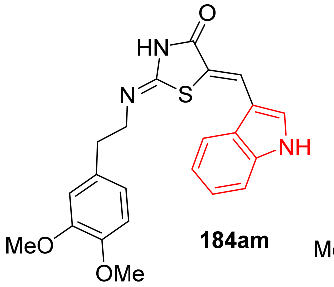

184am

IC_{50} = 19.04 ± 0.98, >30, 17.91 ± 2.54, 23.74 ± 1.31, 27.41 ± 0.10 μM against A549, MDA-MB-231, HCT-29, HCT-15, and NCI-H460, respectively

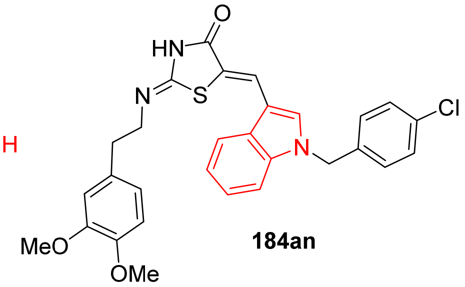

184an

IC_{50} = 23.90 ± 0.49, 29.76 ± 2.78, >30, >30, 17.52 ± 1.03 μM against A549, MDA-MB-231, HCT-29, HCT-15, and NCI-H460, respectively

Fig. S31 (continued). Antiproliferation properties of thiazolidinone-indoles **184** and Podophyllotoxin.

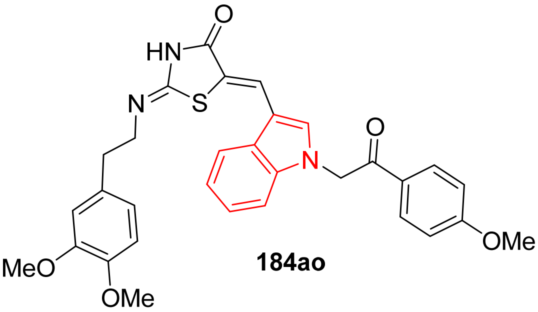

184ao

IC_{50} = 29.05 ± 0.11, >30, 24.85 ± 1.38, >30, 16.52 ± 0.94 μM against A549, MDA-MB-231, HCT-29, HCT-15, and NCI-H460, respectively

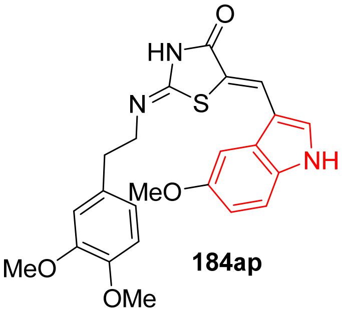

184ap

IC_{50} = 15.09 ± 2.74, >30, 21.03 ± 2.31, >30, 23.18 ± 0.51 μM against A549, MDA-MB-231, HCT-29, HCT-15, and NCI-H460, respectively

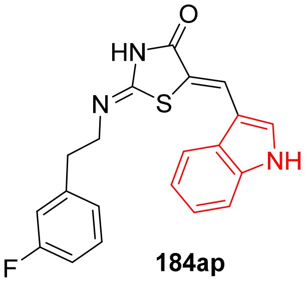

184ap

IC_{50} = >30, 27.83 ± 2.61, >30, 26.40 ± 2.80, >30 μM against A549, MDA-MB-231, HCT-29, HCT-15, and NCI-H460, respectively

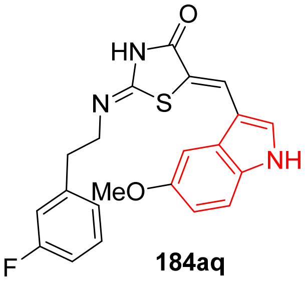

184aq

IC_{50} = 29.01 ± 2.13, >30, 25.94 ± 1.37, 17.13 ± 2.50, 20.09 ± 0.57 μM against A549, MDA-MB-231, HCT-29, HCT-15, and NCI-H460, respectively

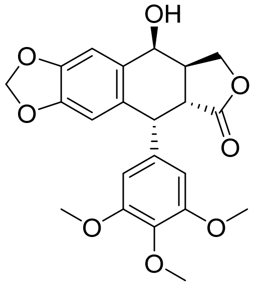

Podophyllotoxin

IC_{50} = 0.027 ± 0.25, 0.014 ± 1.21, 0.08 ± 0.24, 0.029 ± 0.98, 0.037 ± 2.54, 0.021 ± 1.78 mM against A549, MDA-MB-231, HCT-29, HCT-15, NCI-H460, and L132, respectively

Fig. S31 (continued). Antiproliferation properties of thiazolidinone-indoles **184** and Podophyllotoxin.

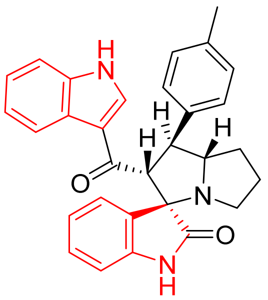

186a

IC_{50} = 21 ± 2, 11.8 ± 2, 16.3 ± 2, 26 μM against HCT-116, HepG2, PC-3 and VERO-B, respectively

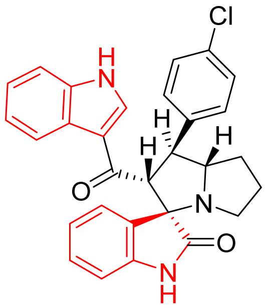

186b

IC_{50} = 20 ± 1.5, 8 ± 0.5, 11.8 ± 1.3, 22 μM against HCT-116, HepG2, PC-3 and VERO-B, respectively

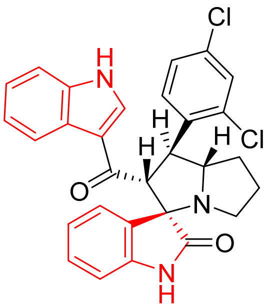

186c

IC_{50} = 9 ± 0.6, 2 ± 0.1, 2 ± 0.125, 9 μM against HCT-116, HepG2, PC-3 and VERO-B, respectively

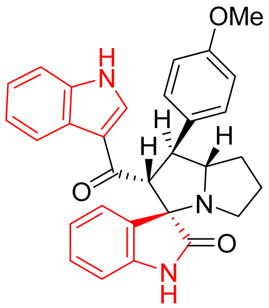

186d

IC_{50} = 26 ± 2, 17.3 ± 3, 15.5 ± 2, 26 μM against HCT-116, HepG2, PC-3 and VERO-B, respectively

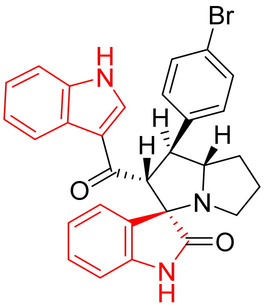

186e

IC_{50} = 21 ± 1.3, 12 ± 1.5, 16.3 ± 2.5, 30 μM against HCT-116, HepG2, PC-3 and VERO-B, respectively

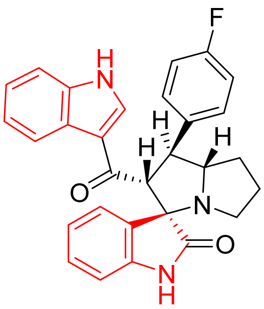

186f

IC_{50} = 16 ± 1, 20 ± 2, 16.3 ± 30, 40 μM against HCT-116, HepG2, PC-3 and VERO-B, respectively

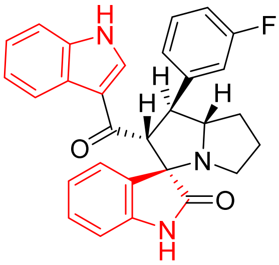

186g

IC_{50} = 15 ± 1.4, 14 ± 0.22, 11.5 ± 11.5, 18 μM against HCT-116, HepG2, PC-3 and VERO-B, respectively

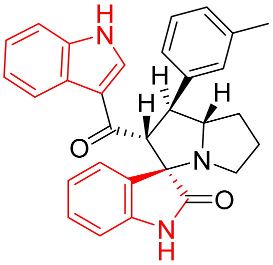

186h

IC_{50} = 7 ± 0.2, 7 ± 0.40, 7 ± 0.6, 15 μM against HCT-116, HepG2, PC-3 and VERO-B, respectively

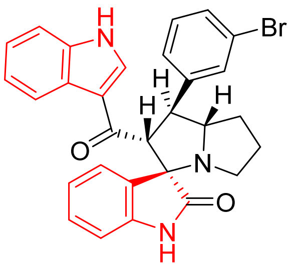

186i

IC_{50} = 9 ± 0.5, 8 ± 1, 7 ± 0.2, 20 μM against HCT-116, HepG2, PC-3 and VERO-B, respectively

Fig. S32. Antiproliferation properties of spiro[indoline-3,3'-pyrrolizin]-2-ones **186** and cisplatin against HCT-116, HepG2, PC-3 (cancer) and VERO-B (normal) cell lines.

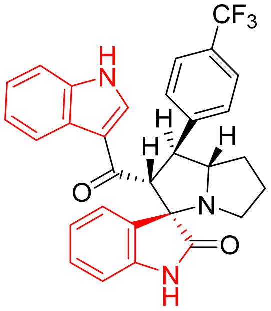

186j

IC_{50} = 9 ± 0.5, 10 ± 1.25, 9 ± 0.2, 22 μM against HCT-116, HepG2, PC-3 and VERO-B, respectively

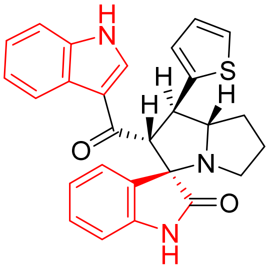

186k

IC_{50} = 50 ± 3.5, 50 ± 3, 29 ± 3, 60 μM against HCT-116, HepG2, PC-3 and VERO-B, respectively

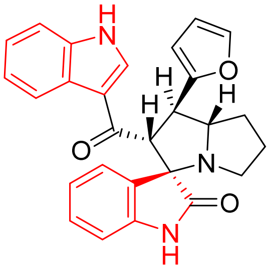

186l

IC_{50} = 29 ± 2, 40 ± 5, 26 ± 1.7, 50 μM against HCT-116, HepG2, PC-3 and VERO-B, respectively

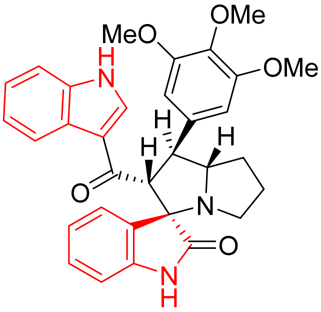

186m

IC_{50} = 20 ± 1.25, 28 ± 2, 17 ± 2, 40 μM against HCT-116, HepG2, PC-3 and VERO-B, respectively

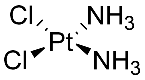

Cisplatin

IC_{50} = 12.6 ± 0.40, 5.5 ± 1.5, 5 ± 0.45, 5 μM against HCT-116, HepG2, PC-3 and VERO-B, respectively

Fig. S32 (continued). Antiproliferation properties of spiro[indoline-3,3'-pyrrolizin]-2-ones **186** and cisplatin against HCT-116, HepG2, PC-3 (cancer) and VERO-B (normal) cell lines.

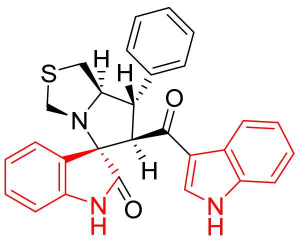

187a

IC_{50} = 30 ± 3, 30 ± 2.4, 30 ± 3.4, 68 μM against HCT-116, HepG2, PC-3 and VERO-B, respectively

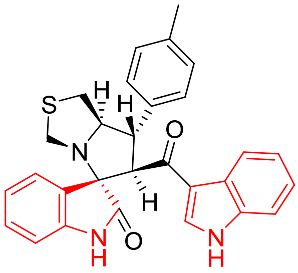

187b

IC_{50} = 9 ± 1.78, 10 ± 2, 15.3 ± 2.3, 28 μM against HCT-116, HepG2, PC-3 and VERO-B, respectively

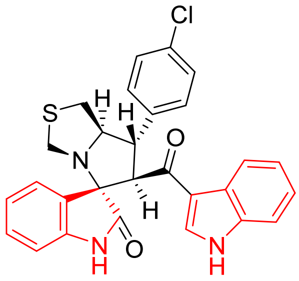

187c

IC_{50} = 11.5 ± 2, 15 ± 1.1, 18.4 ± 1.5, 40 μM against HCT-116, HepG2, PC-3 and VERO-B, respectively

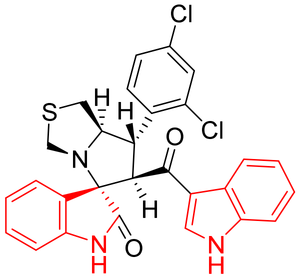

187d

IC_{50} = 9 ± 1.5, 7 ± 1, 6 ± 0.3, 26 μM against HCT-116, HepG2, PC-3 and VERO-B, respectively

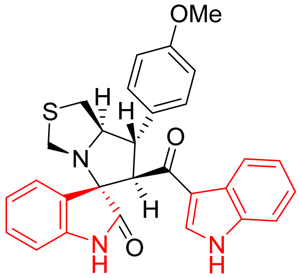

187e

IC_{50} = 26 ± 2, 30 ± 3, 24.6 ± 3, 50 μM against HCT-116, HepG2, PC-3 and VERO-B, respectively

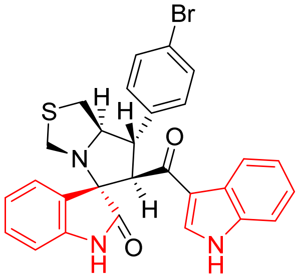

187f

IC_{50} = 11.3 ± 1, 8 ± 1.56, 8 ± 0.5, 22 μM against HCT-116, HepG2, PC-3 and VERO-B, respectively

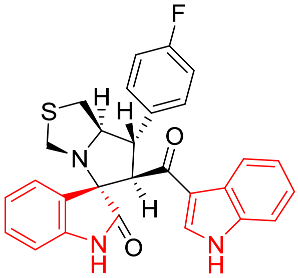

187g

IC_{50} = 13 ± 1.2, 12 ± 1, 16.8 ± 2, 30 μM against HCT-116, HepG2, PC-3 and VERO-B, respectively

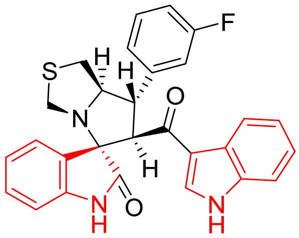

187h

IC_{50} = 9 ± 0.98, 8 ± 2.1, 8 ± 0.45, 22 μM against HCT-116, HepG2, PC-3 and VERO-B, respectively

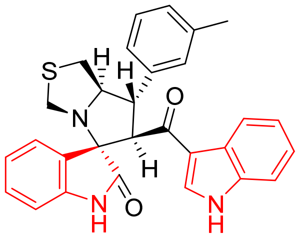

187i

IC_{50} = 15.3 ± 2, 12 ± 2, 17.7 ± 3, 32 μM against HCT-116, HepG2, PC-3 and VERO-B, respectively

Fig. S33. Antiproliferation properties of spiroindoles **187** and Cisplatin against HCT-116, HepG2, PC-3 (cancer) and VERO-B (normal) cell lines.

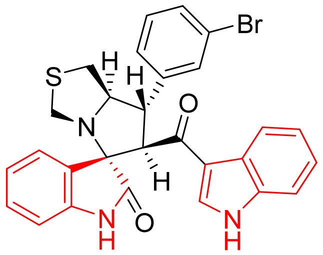

187j

IC_{50} = 9 ± 1, 7 ± 1.5, 7 ± 0.15, 20 μM against HCT-116, HepG2, PC-3 and VERO-B, respectively

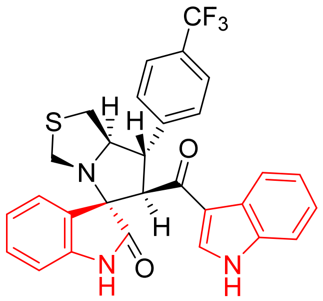

187k

IC_{50} = 7 ± 0.27, 5.5 ± 0.2, 6 ± 0.23, 26 μM against HCT-116, HepG2, PC-3 and VERO-B, respectively

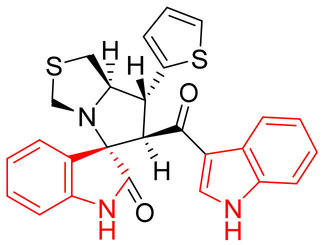

187l

IC_{50} = 90 ± 8, 35 ± 4, 25 ± 2.9, 40 μM against HCT-116, HepG2, PC-3 and VERO-B, respectively

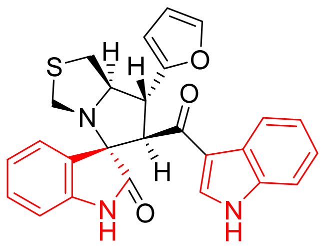

187m

IC_{50} = 40 ± 5, 30 ± 2, 18.5 ± 1.86, 33 μM against HCT-116, HepG2, PC-3 and VERO-B, respectively

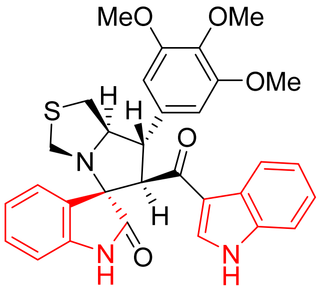

187n

IC_{50} = >40 ± 3, 35 ± 3, 18.5 ± 2, 42 μM against HCT-116, HepG2, PC-3 and VERO-B, respectively

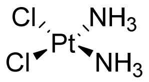

Cisplatin

IC_{50} = 12.6 ± 0.5, 5.5 ± 0.3, 5 ± 0.56, 5 μM against HCT-116, HepG2, PC-3 and VERO-B, respectively

Fig. S33 (continued). Antiproliferation properties of spiroindoles **187** and Cisplatin against HCT-116, HepG2, PC-3 (cancer) and VERO-B (normal) cell lines.

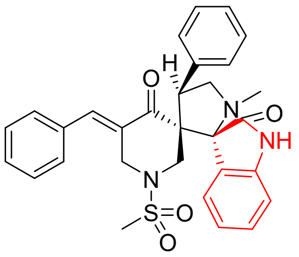

192a

IC_{50} = 19.787 ± 0.99, 15.957 ± 1.10, 32.340 ± 1.14, 48.404 ± 2.23, > 50.000 ± 1.09 μM against MCF7, HCT116, A431, PaCa-2 and RPE1 cell lines, respectively; % inhibition = 66.4, 60.9 against EGFR and VEGFR-2, respectively ustilizing IC_{50} observed against MCF7

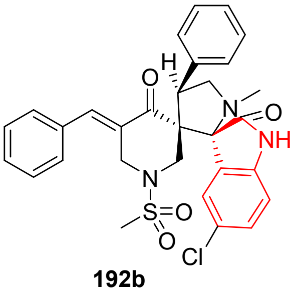

192b

IC_{50} = 7.660 ± 0.68, 6.915 ± 0.52, 9.149 ± 0.70, 20.638 ± 1.17, 24.681 ± 1.26 μM against MCF7, HCT116, A431, PaCa-2 and RPE1 cell lines, respectively; % inhibition = 54.1, 64.2 against EGFR and VEGFR-2, respectively ustilizing IC_{50} observed against MCF7

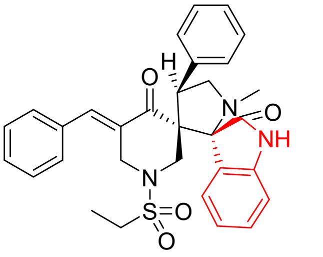

192c

IC_{50} = > 50.000 ± 1.94, 6.125 ± 0.44, 33.191 ± 0.91, > 50.000 ± 2.00, > 50.000 ± 2.00 μM against MCF7, HCT116, A431, PaCa-2 and RPE1 cell lines, respectively; % inhibition = 69.6, 63.8 against EGFR and VEGFR-2, respectively ustilizing IC_{50} observed against MCF7

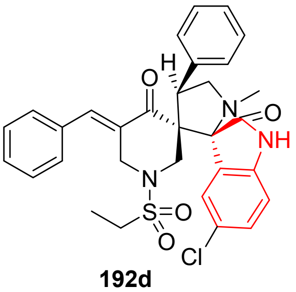

192d

IC_{50} = 6.915 ± 0.55, 5.181 ± 0.61, 4.958 ± 0.25, 13.085 ± 1.10, > 50.000 ± 1.85 μM against MCF7, HCT116, A431, PaCa-2 and RPE1 cell lines, respectively; % inhibition = 62.9, 63.8 against EGFR and VEGFR-2, respectively ustilizing IC_{50} observed against MCF7

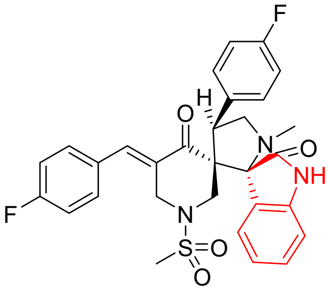

192e

IC_{50} = 15.532 ± 0.76, 9.894 ± 0.85, 16.064 ± 0.99, 39.894 ± 1.89, 49.043 ± 1.11 μM against MCF7, HCT116, A431, PaCa-2 and RPE1 cell lines, respectively; % inhibition = 66.7, 64.5 against EGFR and VEGFR-2, respectively ustilizing IC_{50} observed against MCF7

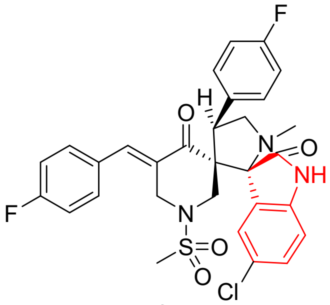

192f

IC_{50} = 5.000 ± 0.39, 5.431 ± 0.46, 4.764 ± 0.37, 11.702 ± 0.94, 17.766 ± 0.87 μM against MCF7, HCT116, A431, PaCa-2 and RPE1 cell lines, respectively; % inhibition = 69.3, 64.7 against EGFR and VEGFR-2, respectively ustilizing IC_{50} observed against MCF7

Fig. S34. Antiproliferation (IC_{50}, μM ± SEM against the tested cell lines), and % inhibitory properties against EGFR and VEGFR-2 utilizing IC_{50} observed against MCF7 for the tested spiroindoles **192** and standard drugs (5-Fluorouracil and Sunitinib).

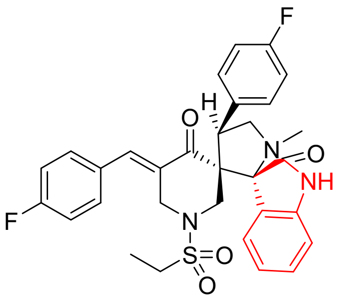

192g

IC_{50} = 10.319 ± 0.86, 4.944 ± 0.25, 6.167 ± 0.44, 28.404 ± 0.85, 33.404 ± 1.22 µM against MCF7, HCT116, A431, PaCa-2 and RPE1 cell lines, respectively; % inhibition = 62.8, 63.7 against EGFR and VEGFR-2, respectively utilizing IC_{50} observed against MCF7

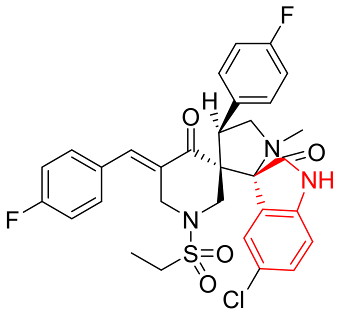

192h

IC_{50} = 4.694 ± 0.44, 4.597 ± 0.18, 6.042 ± 0.26, 14.043 ± 0.73, > 50.000 ± 2.38 µM against MCF7, HCT116, A431, PaCa-2 and RPE1 cell lines, respectively; % inhibition = 67.2, 66.2 against EGFR and VEGFR-2, respectively utilizing IC_{50} observed against MCF7

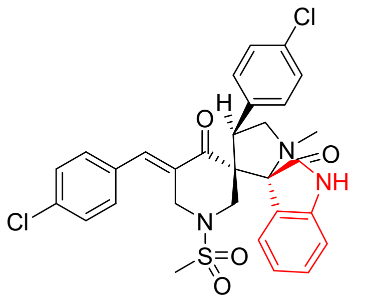

192i

IC_{50} = 5.014 ± 0.29, 5.472 ± 0.32, 4.403 ± 0.49, 9.043 ± 0.62, 14.787 ± 1.57 µM against MCF7, HCT116, A431, PaCa-2 and RPE1 cell lines, respectively; % inhibition = 68.7, 64.2 against EGFR and VEGFR-2, respectively utilizing IC_{50} observed against MCF7

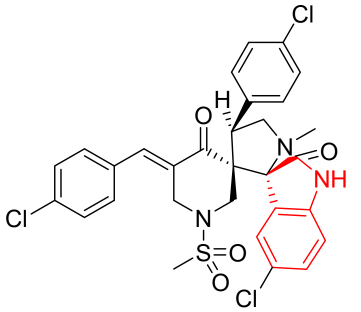

192j

IC_{50} = 4.514 ± 0.39, 4.722 ± 0.25, 4.083 ± 0.21, 8.830 ± 0.51, 12.500 ± 0.86 µM against MCF7, HCT116, A431, PaCa-2 and RPE1 cell lines, respectively; % inhibition = 63.3, 57.9 against EGFR and VEGFR-2, respectively utilizing IC_{50} observed against MCF7

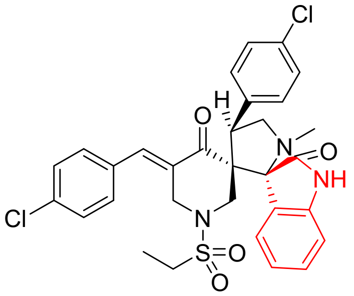

192k

IC_{50} = 4.375 ± 0.26, 4.167 ± 0.38, 2.966 ± 0.29, 8.830 ± 0.70, 14.792 ± 0.99 µM against MCF7, HCT116, A431, PaCa-2 and RPE1 cell lines, respectively; % inhibition = 66.2, 66.2 against EGFR and VEGFR-2, respectively utilizing IC_{50} observed against MCF7

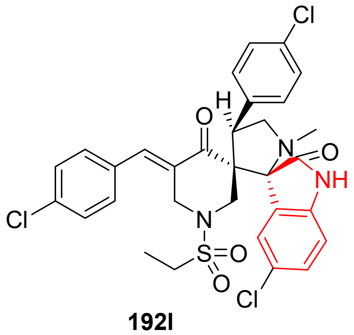

192l

IC_{50} = 3.986 ± 0.31, 4.111 ± 0.41, 3.694 ± 0.33, 11.915 ± 0.83, > 50.000 ± 2.32 µM against MCF7, HCT116, A431, PaCa-2 and RPE1 cell lines, respectively; % inhibition = 65.7, 60.2 against EGFR and VEGFR-2, respectively utilizing IC_{50} observed against MCF7

Fig. S34 (continued). Antiproliferation (IC_{50}, µM ± SEM against the tested cell lines), and % inhibitory properties against EGFR and VEGFR-2 ustilizing IC_{50} observed against MCF7 for the tested spiroindoles **192** and standard drugs (5-Fluorouracil and Sunitinib).

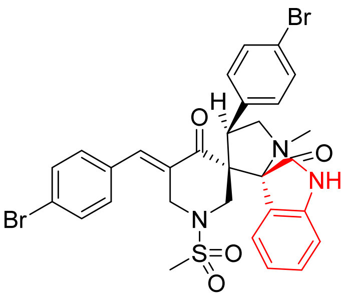

192m

IC_{50} = 3.597 ± 0.19, 3.236 ± 0.27, 2.434 ± 0.18, 12.500 ± 0.67, 14.894 ± 1.61 μM against MCF7, HCT116, A431, PaCa-2 and RPE1 cell lines, respectively; % inhibition = 65.6, 61.3 against EGFR and VEGFR-2, respectively ustilizing IC_{50} observed against MCF7

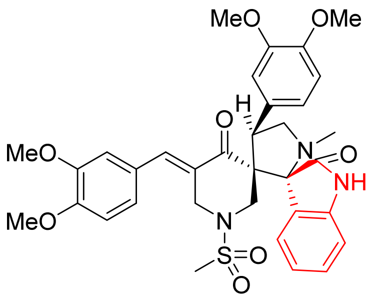

192n

IC_{50} = 40.213 ± 1.10, 15.426 ± 0.52, 34.894 ± 1.36, 32.766 ± 1.21, > 50.000 ± 2.21 μM against MCF7, HCT116, A431, PaCa-2 and RPE1 cell lines, respectively; % inhibition = 69.6, 64.2 against EGFR and VEGFR-2, respectively ustilizing IC_{50} observed against MCF7

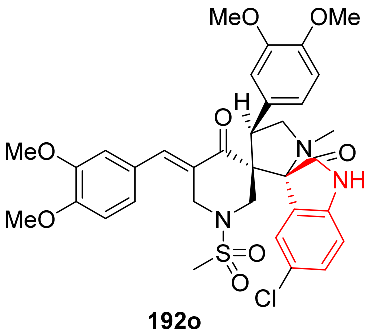

192o

IC_{50} = 48.936 ± 1.84, 28.511 ± 0.75, 45.417 ± 1.84, > 50.000 ± 2.31, > 50.000 ± 2.61 μM against MCF7, HCT116, A431, PaCa-2 and RPE1 cell lines, respectively; % inhibition = 65.9, 61.8 against EGFR and VEGFR-2, respectively ustilizing IC_{50} observed against MCF7

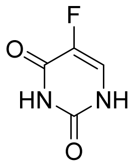

5- Fluorouracil

IC_{50} = 3.15 ± 0.44, 20.43 ± 1.99, 23.44 ± 2.09 μM against MCF7, HCT116 and A431 cell lines, respectively

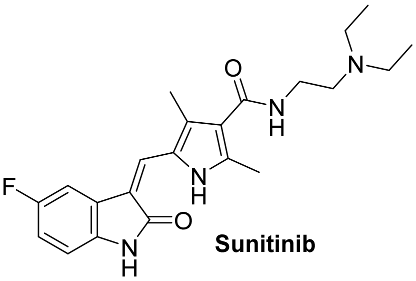

Sunitinib

IC_{50} = 3.97 ± 0.32, 9.67 ± 0.22, 16.91 ± 0.95 μM against MCF7, HCT116, and PaCa-2 cell lines, respectively; % inhibition = 81.4, 74.7 against EGFR and VEGFR-2, respectively ustilizing IC_{50} observed against MCF7

Fig. S34 (continued). Antiproliferation (IC_{50}, μM ± SEM against the tested cell lines), and % inhibitory properties against EGFR and VEGFR-2 ustilizing IC_{50} observed against MCF7 for the tested spiroindoles **192** and standard drugs (5-Fluorouracil and Sunitinib).

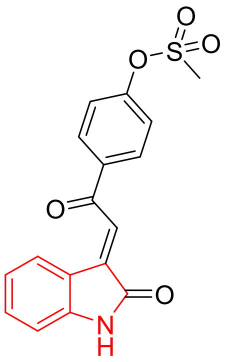

196a

IC_{50} = 8.30 ± 0.44, 6.85 ± 0.37, 16.38 ± 0.70 μM against PaCa-2, MCF7, and HCT116, respectively; IC_{50} = 54.03 ± 5.4, 119.8 ± 12.0 nM against VEGFR-2, and c-kit, respectively

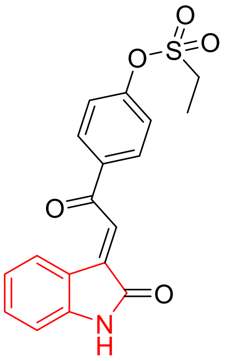

196b

IC_{50} = 5.60 ± 0.57, 4.25 ± 0.23, 12.77 ± 1.41 μM against PaCa-2, MCF7, and HCT116, respectively; IC_{50} = 98.95 ± 9.9, 247.7 ± 24.8 nM against VEGFR-2, and c-kit, respectively

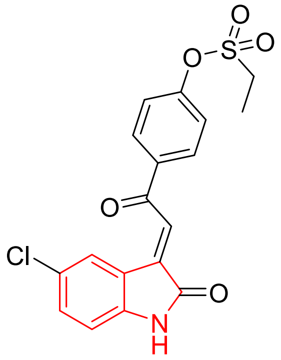

196c

IC_{50} = 4.99 ± 0.29, 4.28 ± 0.51, 5.33 ± 0.46 μM against PaCa-2, MCF7, and HCT116, respectively; IC_{50} = 36.86 ± 3.7, 72.35 ± 7.2 nM against VEGFR-2, and c-kit, respectively

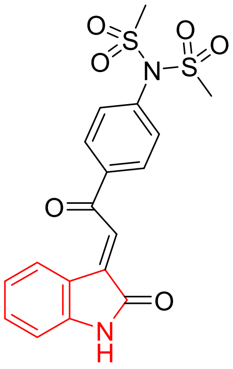

200a

IC_{50} = 6.91 ± 0.89, 6.07 ± 0.83, 20.96 ± 1.75 μM against PaCa-2, MCF7, and HCT116, respectively; IC_{50} = 102.2 ± 10.2, 103.3 ± 10.3 nM against VEGFR-2, and c-kit, respectively

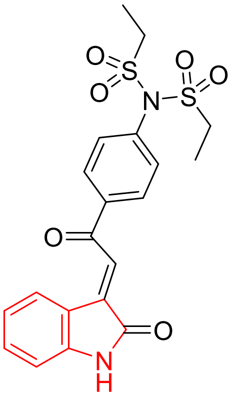

200b

IC_{50} = 5.08 ± 0.57, 4.15 ± 0.78, 13.83 ± 1.06 μM against PaCa-2, MCF7, and HCT116, respectively; IC_{50} = 74.06 ± 7.4, 184.9 ± 18.5 nM against VEGFR-2, and c-kit, respectively

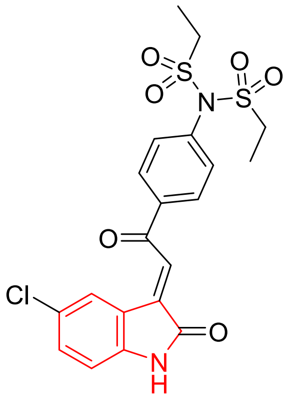

200c

IC_{50} = 6.18 ± 0.32, 4.43 ± 0.47, >50.00 ± 0.84 μM against PaCa-2, MCF7, and HCT116, respectively; IC_{50} = 53.36 ± 5.3, 81.7 ± 8.2 nM against VEGFR-2, and c-kit, respectively

Fig. S35. Antiproliferation (IC_{50}, μM ± SE "standard error") and enzymatic inhibitory properties (IC_{50}, μM ± SD) of 3-alkenyl-2-oxindoles **196**, **200** and Sunitinib.

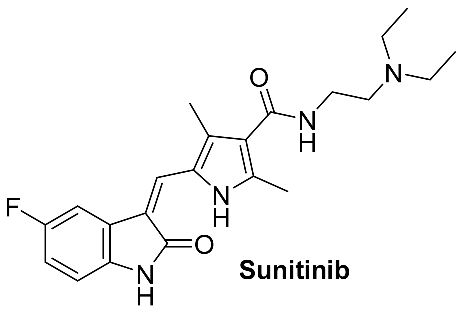

Sunitinib

IC_{50} = 16.91 ± 0.95, 3.97 ± 0.14, 5.03 ± 0.30 μM against PaCa-2, MCF7, and HCT116, respectively; IC_{50} = 47.54 ± 4.8 84.9 ± 8.5 nM against VEGFR-2, and c-kit, respectively

Fig. S35 (continued). Antiproliferation (IC_{50}, μM ± SE "standard error") and enzymatic inhibitory properties (IC_{50}, μM ± SD) of 3-alkenyl-2-oxindoles **196**, **200** and Sunitinib.

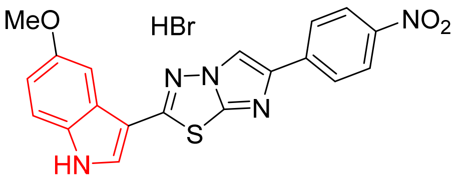

203a

IC_{50} = 5.5 ± 0.19, 5.11 ± 0.29, 5.18 ± 0.12 μM against SUIT-2, Capan-1, and Panc-1, respectively

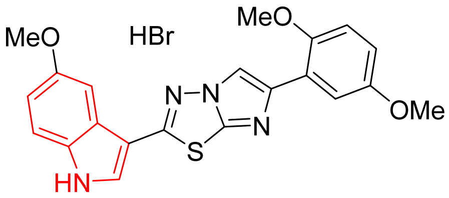

203b

IC_{50} = 10.26 ± 0.20 μM against Panc-1

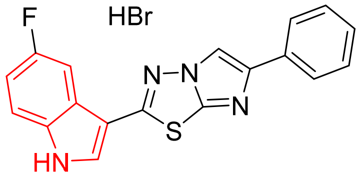

203c

IC_{50} = 10.4 ± 0.07, 8.57 ± 0.51, 10.8 ± 0.13 μM against SUIT-2, Capan-1, and Panc-1, respectively

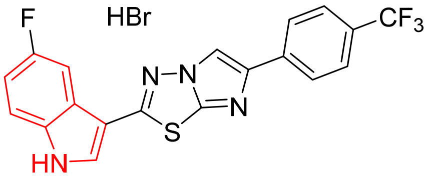

203d

IC_{50} = 11.8 ± 0.54, 10.49 ± 0.16 μM against SUIT-2, and Capan-1, respectively

Fig. S36. Antiproliferation properties (μM ± SEM) of indole linked to imidazo[2,1-*b*][1,3,4]thiadiazoles **203**.

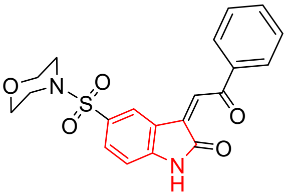

206a

IC_{50} = 8.59 ± 0.31, 7.99 ± 0.37, 11.36 ± 0.44 μM against HEPG-2, HCT-116, and MCF-7, respectively

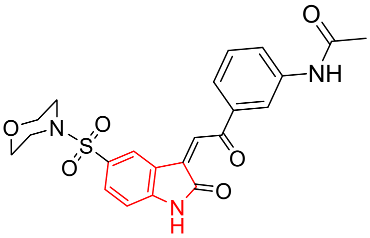

206b

IC_{50} = 6.09 ± 0.32, 5.32 ± 0.21, 9.56 ± 0.47 μM against HEPG-2, HCT-116, and MCF-7, respectively; IC_{50} = 0.0191 μM against EGFR

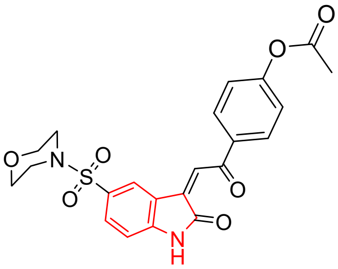

206c

IC_{50} = 4.41 ± 0.12, 3.55 ± 0.09, 6.67 ± 0.36 μM against HEPG-2, HCT-116, and MCF-7, respectively

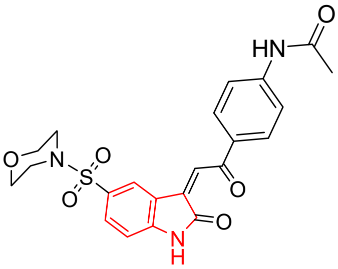

206d

IC_{50} = 6.26 ± 0.22, 8.24 ± 0.32, 14.46 ± 0.62 μM against HEPG-2, HCT-116, and MCF-7, respectively

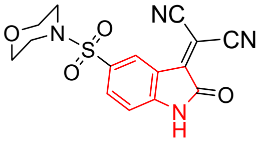

208a

IC_{50} = 6.08 ± 0.34, 5.96 ± 0.22, 6.34 ± 0.21 μM against HEPG-2, HCT-116, and MCF-7, respectively; IC_{50} = 0.0266 μM against EGFR

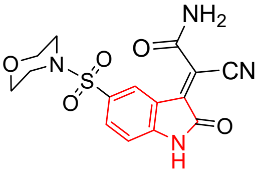

208b

IC_{50} = 8.62 ± 0.3, 8.31 ± 0.25, 11.87 ± 0.41 μM against HEPG-2, HCT-116, and MCF-7, respectively

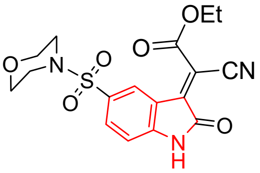

208c

IC_{50} = 5.19 ± 0.14, 7.70 ± 0.34, 6.39 ± 0.22 μM against HEPG-2, HCT-116, and MCF-7, respectively; IC_{50} = 1.382 μM against EGFR

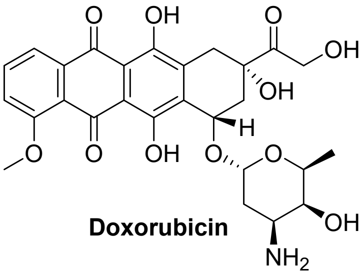

Doxorubicin

IC_{50} = 5.59 ± 0.55, 7.03 ± 0.21, 4.89 ± 0.47 μM against HEPG-2, HCT-116, and MCF-7, respectively

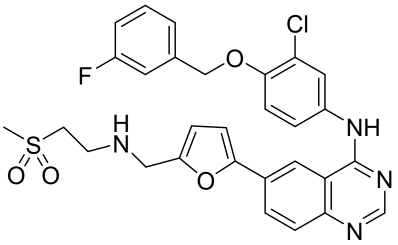

Lapatinib

IC_{50} = 0.0283 μM against EGFR

Fig. S37. Antiproliferation (μM ± SE) and enzymatic inhibitory properties against EGFR of 5-(morpholinosulfonyl)-2-indolinones **206**, 3-ylidene-2-indolinones **208** and standard references (Doxorubicin and Lapatinib).

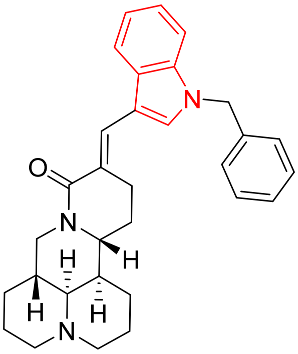

210a

IC_{50} = 6.95 ± 0.47 μM
against HepG2

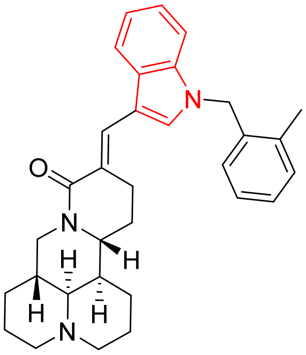

210b

IC_{50} = 16.32 ± 1.35 μM
against HepG2

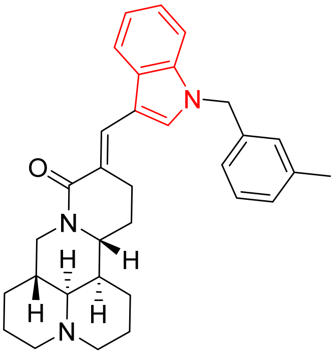

210c

IC_{50} = 8.27 ± 0.90 μM
against HepG2

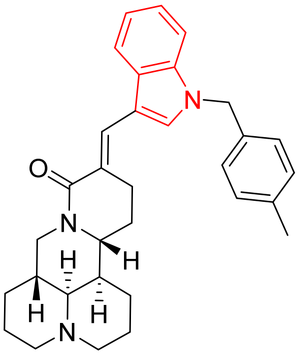

210d

IC_{50} = 9.39 ± 1.01 μM
against HepG2

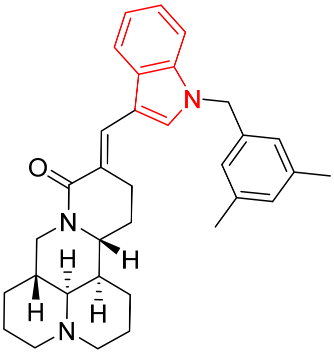

210e

IC_{50} = 4.56 ± 0.44 μM
against HepG2

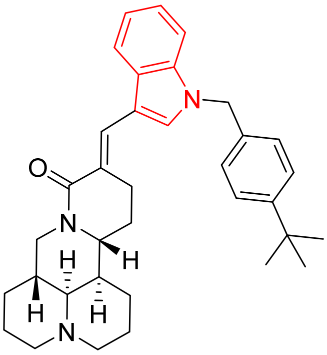

210f

IC_{50} = 4.57 ± 0.51 μM
against HepG2

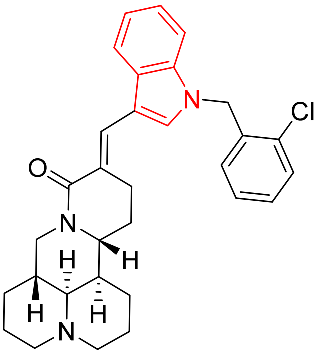

210g

IC_{50} = 9.05 ± 0.88 μM
against HepG2

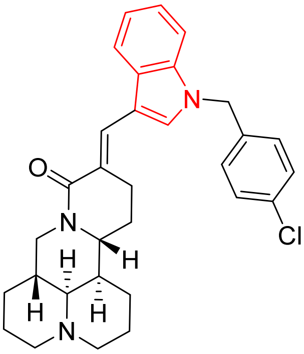

210h

IC_{50} = 6.59 ± 0.62 μM
against HepG2

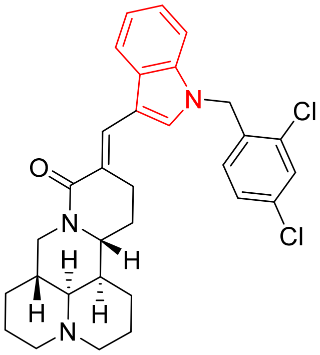

210i

IC_{50} = 9.78 ± 0.81 μM
against HepG2

Fig. S38. Antiproliferation properties (μM ± SD) of the synthesized sophoridine-indole conjugates **210**, Sophoridine **209** and Camptothecin (standard reference).

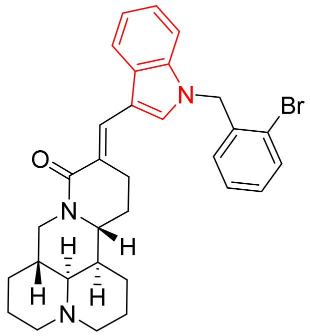

210j

IC_{50} = 15.43 ± 0.99 μM
against HepG2

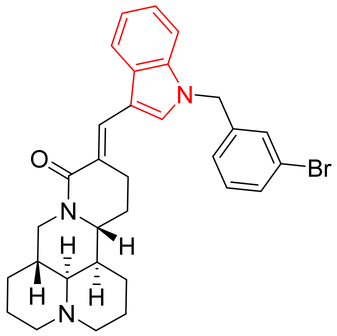

210k

IC_{50} = 8.62 ± 0.98 μM
against HepG2

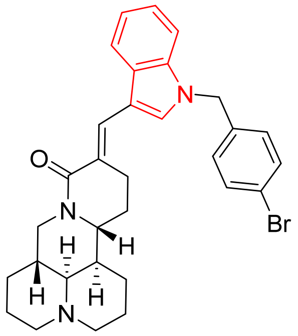

210l

IC_{50} = 5.94 ± 0.49 μM
against HepG2

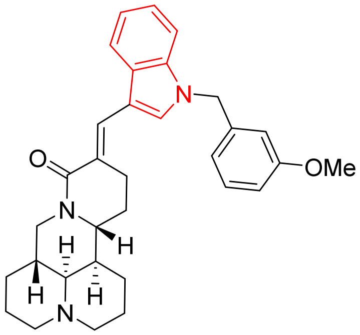

210m

IC_{50} = 8.14 ± 0.73 μM
against HepG2

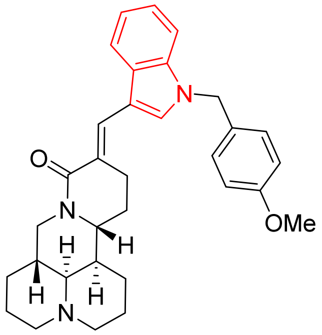

210n

IC_{50} = 8.23 ± 0.76 μM
against HepG2

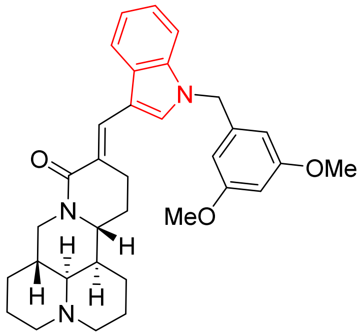

210o

IC_{50} = 6.88 ± 0.61 μM
against HepG2

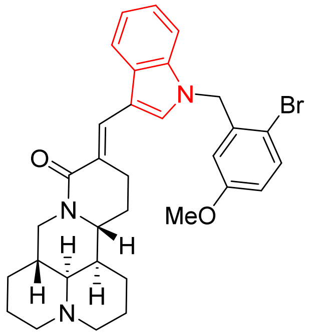

210p

IC_{50} = 4.16 ± 0.39 μM
against HepG2

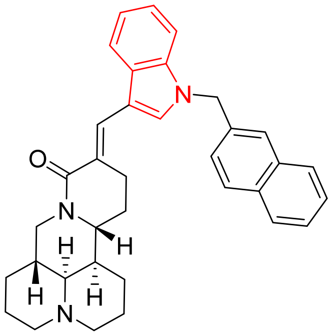

210q

IC_{50} = 12.78 ± 0.94 μM
against HepG2

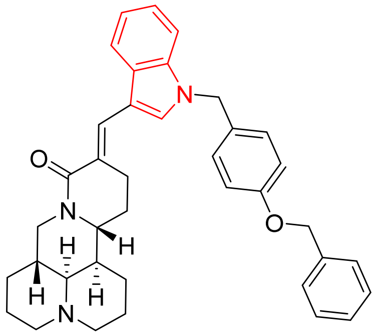

210r

IC_{50} = 3.08 ± 0.40 μM
against HepG2

Fig. S38 (continued). Antiproliferation properties (μM ± SD) of the synthesized sophoridine-indole conjugates **210**, Sophoridine **209** and Camptothecin (standard reference).

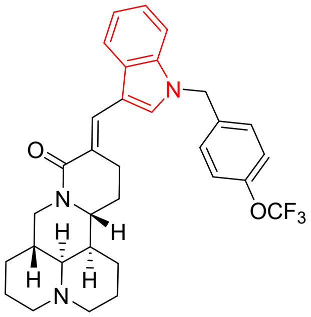

210s

IC_{50} = 8.73 ± 0.72 μM against HepG2

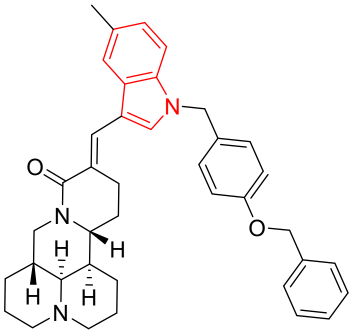

210t

IC_{50} = 7.71 ± 0.71 μM against HepG2

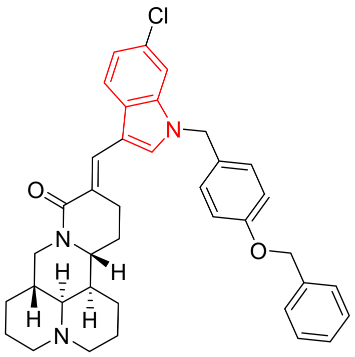

210u

IC_{50} = 14.29 ± 1.27 μM against HepG2

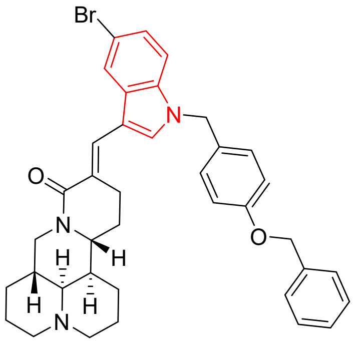

210v

IC_{50} = 3.97 ± 0.35 μM against HepG2

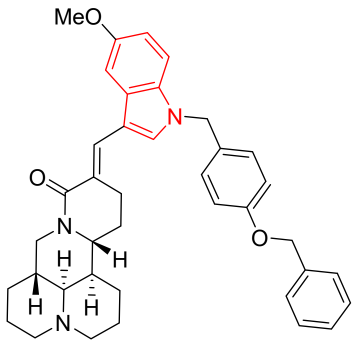

210w

IC_{50} = 1.96 ± 0.39, 1.32 ± 0.09, 1.44 ± 0.12, 1.89 ± 0.13, 1.24 ± 0.19, 0.94 ± 0.11 μM against HepG2, SMMC-7721, Hela, CNE1, CNE2, and MCF7, respectively

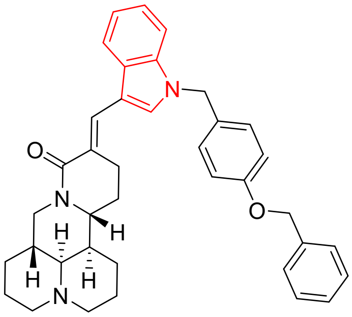

210x

IC_{50} = 15.24 ± 1.20 μM against HepG2

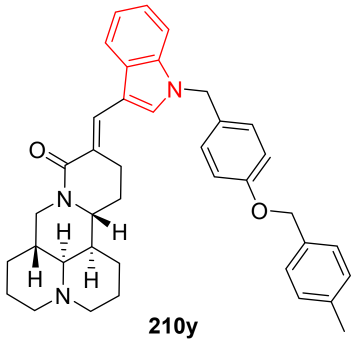

210y

IC_{50} = 5.32 ± 0.45 μM against HepG2

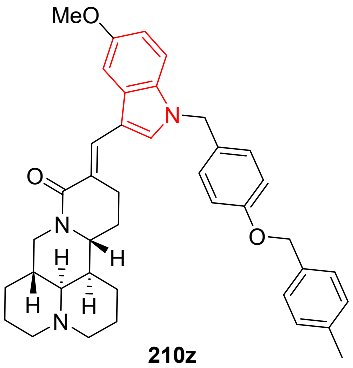

210z

IC_{50} = 4.18 ± 0.38 μM against HepG2

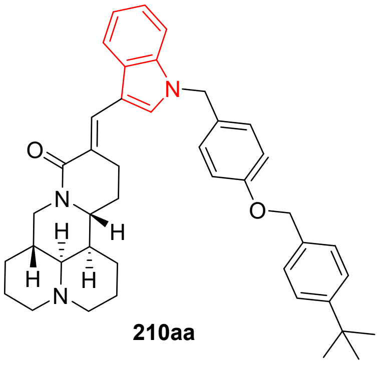

210aa

IC_{50} = 6.07 ± 0.48 μM against HepG2

Fig. S38 (continued). Antiproliferation properties (μM ± SD) of the synthesized sophoridine-indole conjugates **210**, Sophoridine **209** and Camptothecin (standard reference).

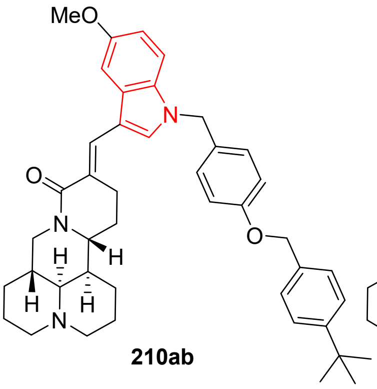

210ab

IC_{50} = 5.86 ± 0.41 μM
against HepG2

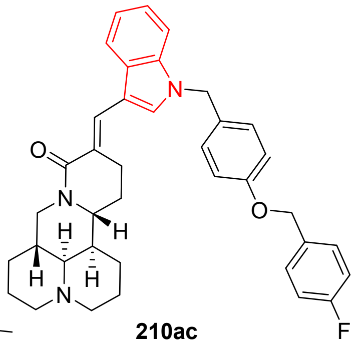

210ac

IC_{50} = 4.83 ± 0.32 μM
against HepG2

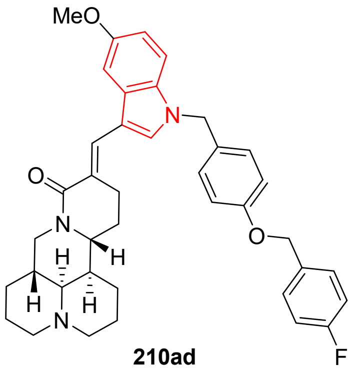

210ad

IC_{50} = 3.11 ± 0.21 μM
against HepG2

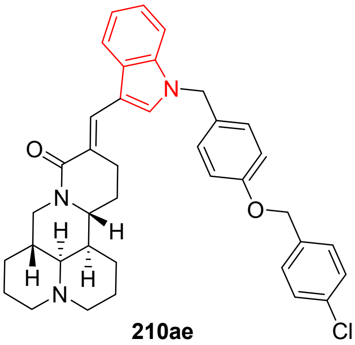

210ae

IC_{50} = 6.17 ± 0.58 μM
against HepG2

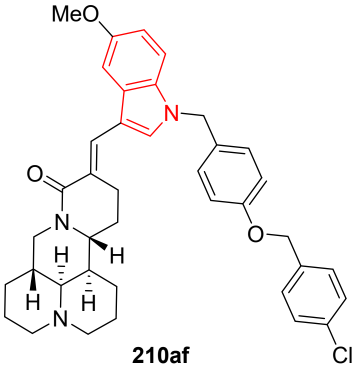

210af

IC_{50} = 3.97 ± 0.24 μM
against HepG2

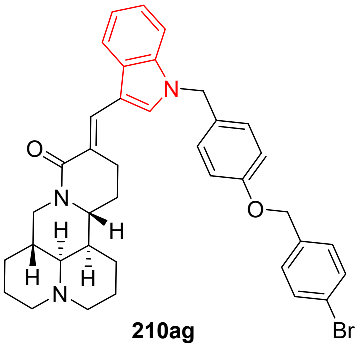

210ag

IC_{50} = 10.08 ± 0.92 μM
against HepG2

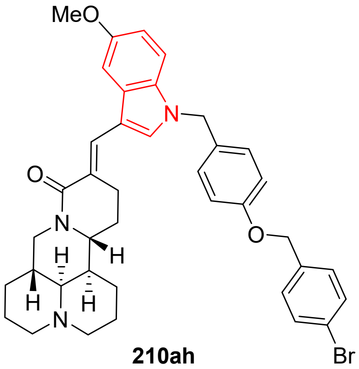

210ah

IC_{50} = 6.31 ± 0.55 μM
against HepG2

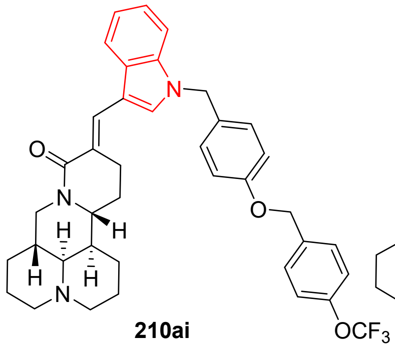

210ai

IC_{50} = 6.01 ± 0.56 μM
against HepG2

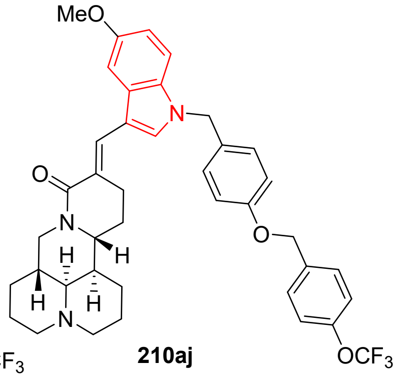

210aj

IC_{50} = 5.72 ± 0.42 μM
against HepG2

Fig. S38 (continued). Antiproliferation properties (μM ± SD) of the synthesized sophoridine-indole conjugates **210**, Sophoridine **209** and Camptothecin (standard reference).

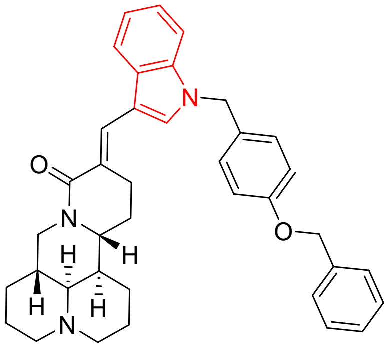

210k

IC_{50} = 9.96 ± 0.78 μM
against HepG2

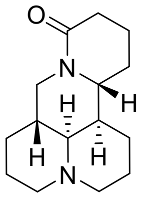

209 (Sophoridine)

IC_{50} = 4670 ± 127 μM
against HepG2

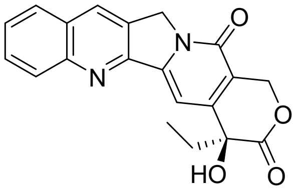

Camptothecin

IC_{50} = 1.36 ± 0.17, 1.08 ± 0.13, 0.66 ± 0.18, 0.34 ± 0.07, 0.98 ± 0.11, 0.42 ± 0.08 μM against HepG2, SMMC-7721, Hela, CNE1, CNE2, and MCF7, respectively

Fig. S38 (continued). Antiproliferation properties (μM ± SD) of the synthesized sophoridine-indole conjugates **210**, Sophoridine **209** and Camptothecin (standard reference).

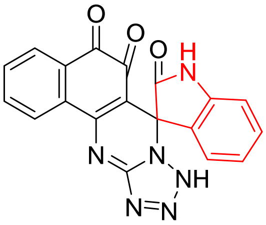

213a

IC_{50} = 23.63, 40.15 μM against HepG2 and LO2, respectively

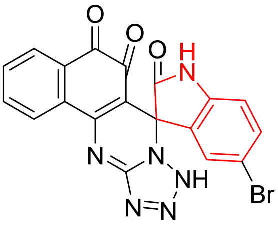

213b

IC_{50} = 23.41, 65.29 μM against HepG2 and LO2, respectively

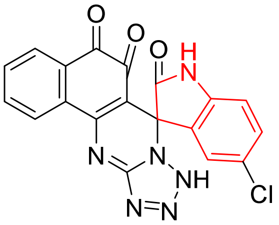

213c

IC_{50} = 21.93, 53.03 μM against HepG2 and LO2, respectively

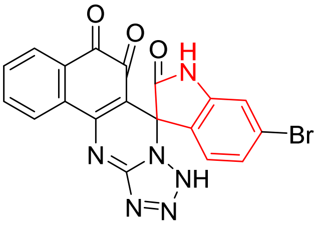

213d

IC_{50} = 36.34, 248.39 μM against HepG2 and LO2, respectively

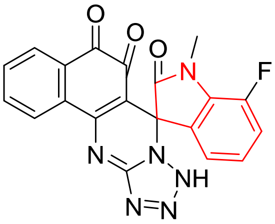

213e

IC_{50} = 31.83, 54 μM against HepG2 and LO2, respectively

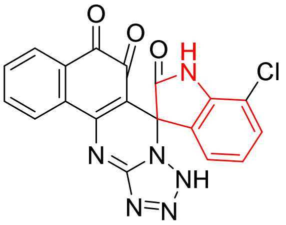

213f

IC_{50} = 3.03, 49.47 μM against HepG2 and LO2, respectively

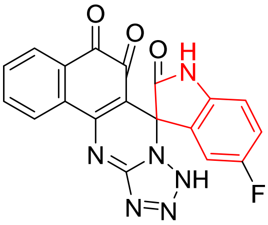

213g

IC_{50} = 2.86, 58.92 μM against HepG2 and LO2, respectively

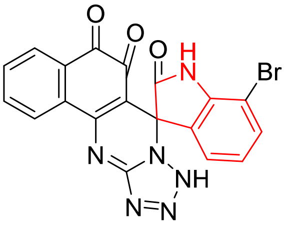

213h

IC_{50} = 20.74, 82.34 μM against HepG2 and LO2, respectively

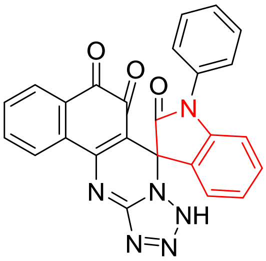

213i

IC_{50} = 27.87, 75.57 μM against HepG2 and LO2, respectively

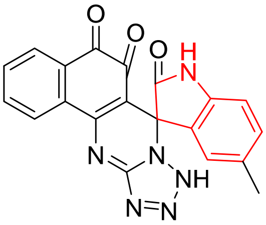

213j

IC_{50} = 12.58, 36.37 μM against HepG2 and LO2, respectively

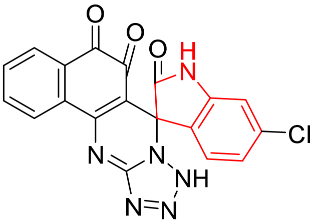

213k

IC_{50} = 21.19, 51.99 μM against HepG2 and LO2, respectively

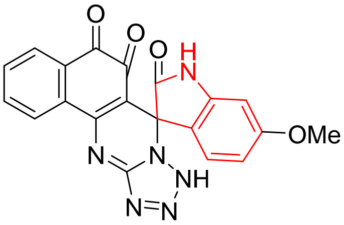

213l

IC_{50} = 17.29, 78.6 μM against HepG2 and LO2, respectively

Fig. S39. Antiproliferation properties of spirooxindoles **213** and Tanshinon IIA (TSA).

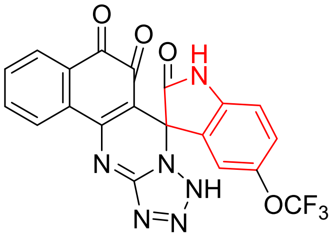

213m

IC_{50} = 18.29, 48.42 μM against HepG2 and LO2, respectively

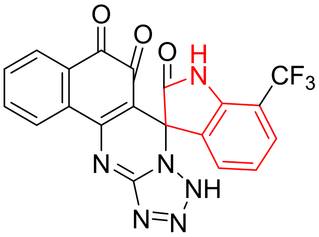

213n

IC_{50} = 7.9, 49.9 μM against HepG2 and LO2, respectively

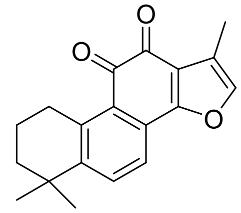

Tanshinon IIA (TSA)

IC_{50} = 23.85, 65.29 μM against HepG2 and LO2, respectively

Fig. S39 (continued). Antiproliferation properties of spirooxindoles **213** and Tanshinon IIA (TSA).

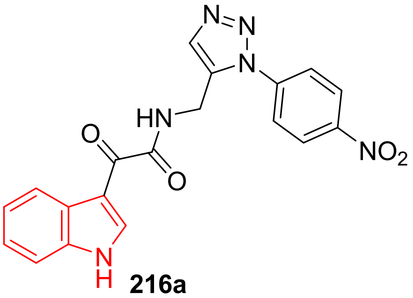

216a

IC_{50} = >100, >100, >100 μM against SKOV3, DU145, HELA, respectively; IC_{50} = 2.03, 0.85, 7.85 μM against COX-1. COX-2, 5-LOX, respectively

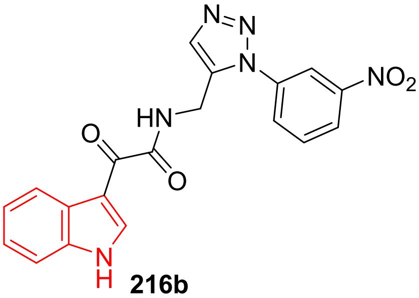

216b

IC_{50} = >100, >100, >100 μM against SKOV3, DU145, HELA, respectively; IC_{50} = 2.43, 0.65, 6.85 μM against COX-1. COX-2, 5-LOX, respectively

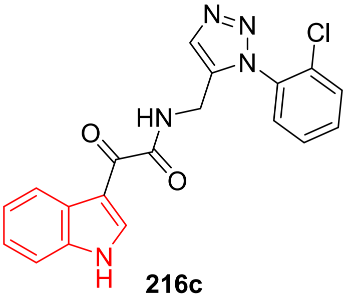

216c

IC_{50} = >100, >100, >100 μM against SKOV3, DU145, HELA, respectively; IC_{50} = 2.23, 0.35, 8.48 μM against COX-1. COX-2, 5-LOX, respectively

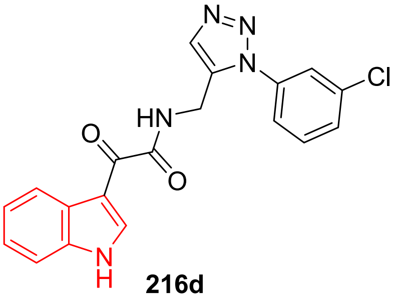

216d

IC_{50} = 22.8, >100, >100 μM against SKOV3, DU145, HELA, respectively; IC_{50} = 2.14, 0.18, 7.42 μM against COX-1. COX-2, 5-LOX, respectively

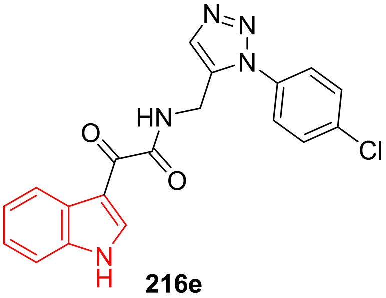

216e

IC_{50} = 12.70, >100, >100 μM against SKOV3, DU145, HELA, respectively; IC_{50} = 2.02, 0.99, 8.62 μM against COX-1. COX-2, 5-LOX, respectively

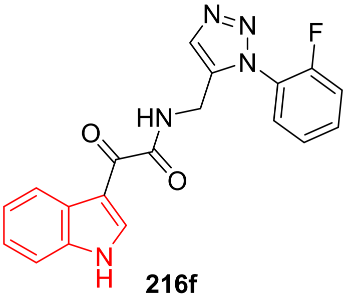

216f

IC_{50} = >100, >100, >100 μM against SKOV3, DU145, HELA, respectively; IC_{50} = 2.23, 0.35, 9.48 μM against COX-1. COX-2, 5-LOX, respectively

Fig. S40. Antiproliferation and enzymation inhibitory properties of indole-triazole conjugates **216**, Etoposide, Indomethacin, Celecoxib, and Norhihydroguaiaretic acid (ND = not done).

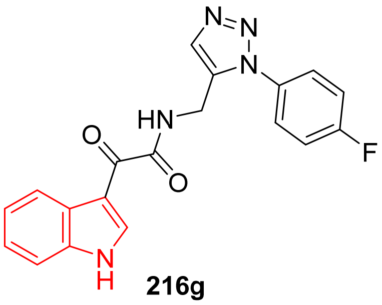

216g

IC_{50} = >100, 8.69, >100 μM against SKOV3, DU145, HELA, respectively; IC_{50} = 2.14, 1.99, 8.62 μM against COX-1. COX-2, 5-LOX, respectively

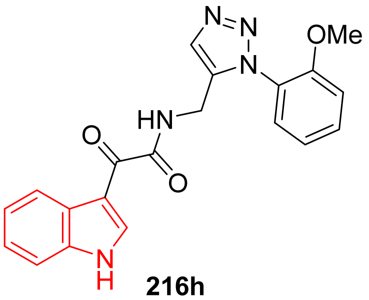

216h

IC_{50} = >100, 28.65, >100 μM against SKOV3, DU145, HELA, respectively; IC_{50} = 3.01, 1.84, 9.54 μM against COX-1. COX-2, 5-LOX, respectively

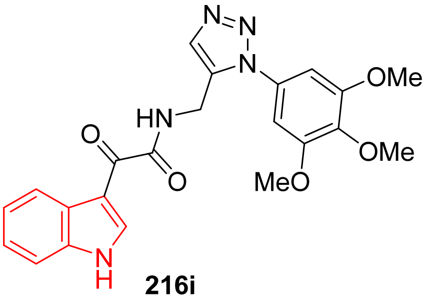

216i

IC_{50} = 11.91, 39.57, >100 μM against SKOV3, DU145, HELA, respectively; IC_{50} = 2.44, 1.76, 7.83 μM against COX-1. COX-2, 5-LOX, respectively

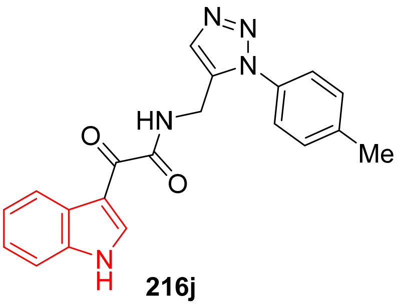

216j

IC_{50} = >100, >100, >100 μM against SKOV3, DU145, HELA, respectively; IC_{50} = 2.48, 0.33, 7.93 μM against COX-1. COX-2, 5-LOX, respectively

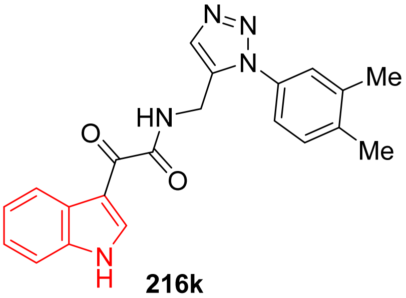

216k

IC_{50} = >100, >100, 6.29 μM against SKOV3, DU145, HELA, respectively; IC_{50} = 3.02, 1.68, 8.59 μM against COX-1. COX-2, 5-LOX, respectively

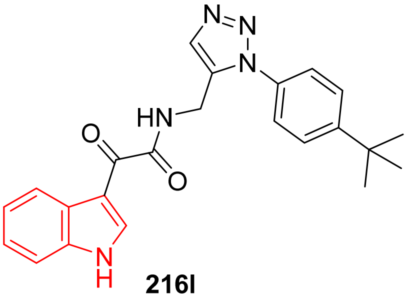

216l

IC_{50} = >100, >100, >100 μM against SKOV3, DU145, HELA, respectively; IC_{50} = 2.24, 0.19, 7.51 μM against COX-1. COX-2, 5-LOX, respectively

Fig. S40 (continued). Antiproliferation and enzymation inhibitory properties of indole-triazole conjugates **216**, Etoposide, Indomethacin, Celecoxib, and Norhihydroguaiaretic acid (ND = not done).

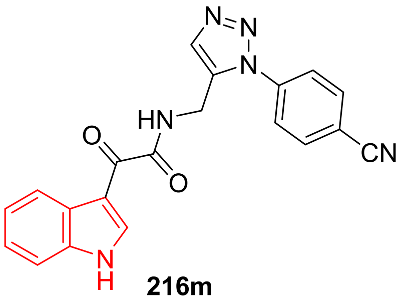

216m

IC_{50} = >100, >100, >100 μM against SKOV3, DU145, HELA, respectively; IC_{50} = 2.03, 0.159, 7.73 μM against COX-1. COX-2, 5-LOX, respectively

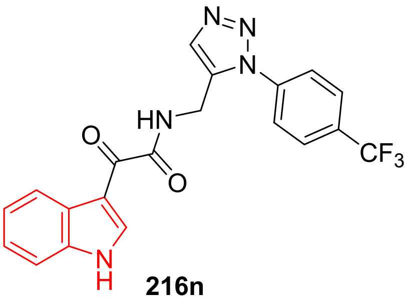

216n

IC_{50} = >100, 24.45, >100 μM against SKOV3, DU145, HELA, respectively; IC_{50} = 3.06, 2.95, 12.65 μM against COX-1. COX-2, 5-LOX, respectively

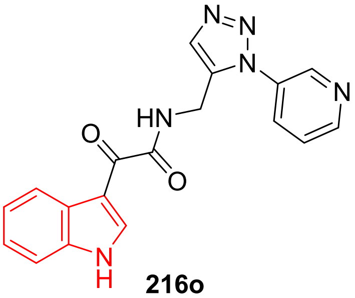

216o

IC_{50} = >100, 17.15, >100 μM against SKOV3, DU145, HELA, respectively; IC_{50} = 2.03, 0.48, 7.93 μM against COX-1. COX-2, 5-LOX, respectively

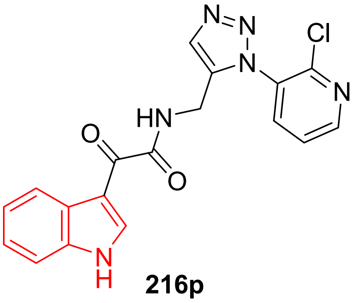

216p

IC_{50} = >100, >100, >300 μM against SKOV3, DU145, HELA, respectively; IC_{50} = 2.15, 0.85, 7.85 μM against COX-1. COX-2, 5-LOX, respectively

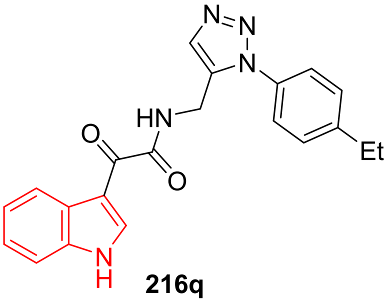

216q

IC_{50} = >100, 8.17, >100 μM against SKOV3, DU145, HELA, respectively; IC_{50} = 2.06, 0.12, 7.73 μM against COX-1. COX-2, 5-LOX, respectively

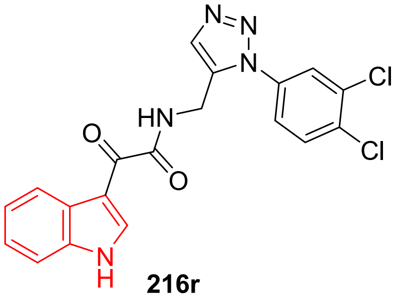

216r

IC_{50} = 12.63, 15.96, >100 μM against SKOV3, DU145, HELA, respectively; IC_{50} = 2.24, 0.16, 7.51 μM against COX-1. COX-2, 5-LOX, respectively

Fig. S40 (continued). Antiproliferation and enzymation inhibitory properties of indole-triazole conjugates **216**, Etoposide, Indomethacin, Celecoxib, and Norhihydroguaiaretic acid (ND =not done).

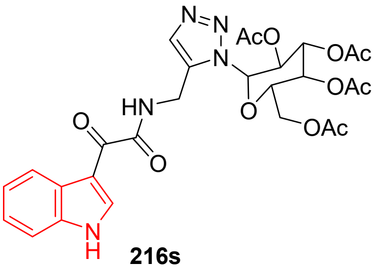

216s

IC_{50} = >100, >100, >100 μM against SKOV3, DU145, HELA, respectively; IC_{50} = 2.15, 0.90, 8.62 μM against COX-1. COX-2, 5-LOX, respectively

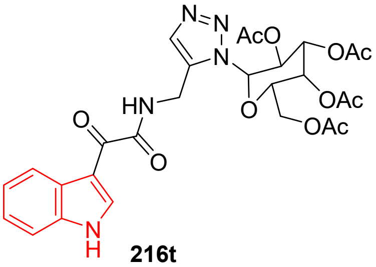

216t

IC_{50} = ND, ND, ND μM against SKOV3, DU145, HELA, respectively; IC_{50} = 2.14, 1.88, 11.58 μM against COX-1. COX-2, 5-LOX, respectively

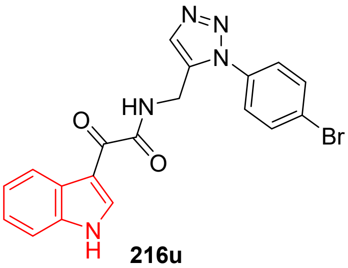

216u

IC_{50} = 63.42, >100, >100 μM against SKOV3, DU145, HELA, respectively; IC_{50} = 2.13, 0.489, 11.36 μM against COX-1. COX-2, 5-LOX, respectively

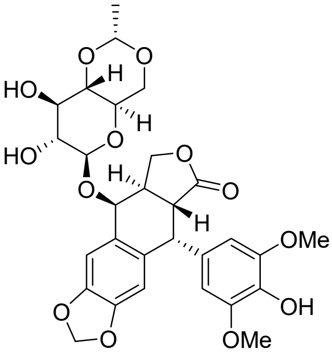

Etoposide (VP16)

IC_{50} = 2.39, 9.8, 7.43 μM against SKOV3, DU145, HELA, respectively

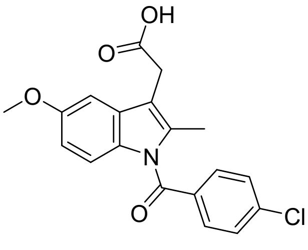

Indomethacin

IC_{50} = 0.0068, 0.049 μM against COX-1. COX-2, respectively

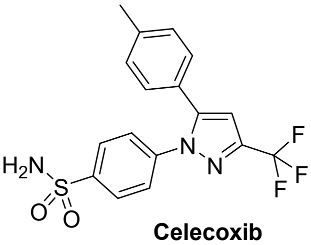

Celecoxib

IC_{50} = 14.8, 0.041 μM against COX-1. COX-2, respectively

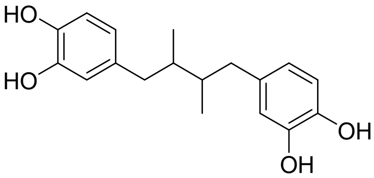

Norhihydroguaiaretic acid (NDGA)

IC_{50} = 7.31 μM against 5-LOX

Fig. S40 (continued). Antiproliferation and enzymation inhibitory properties of indole-triazole conjugates **216**, Etoposide, Indomethacin, Celecoxib, and Norhihydroguaiaretic acid (ND = not done).

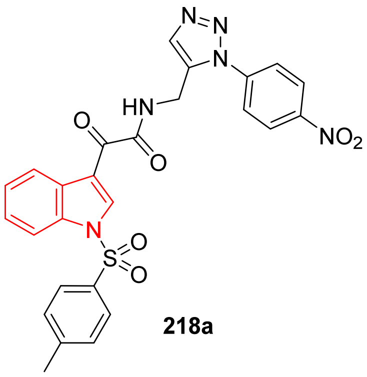

218a

IC_{50} = ND, ND, ND μM against SKOV3, DU145, HELA, respectively; IC_{50} = 3.12, 0.66, 9.75 μM against COX-1. COX-2, 5-LOX, respectively

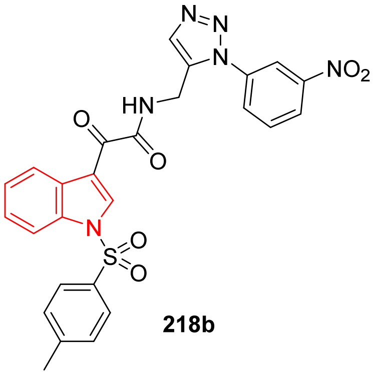

218b

IC_{50} = >100, 16.73, >100 μM against SKOV3, DU145, HELA, respectively; IC_{50} = 2.28, 0.75, 11.43 μM against COX-1. COX-2, 5-LOX, respectively

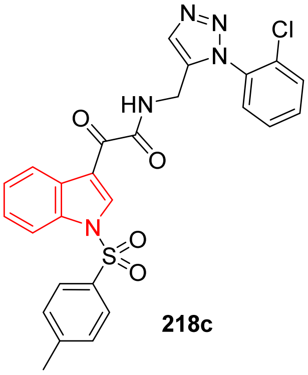

218c

IC_{50} = ND, ND, ND μM against SKOV3, DU145, HELA, respectively; IC_{50} = 4.15, 1.85, 10.85 μM against COX-1. COX-2, 5-LOX, respectively

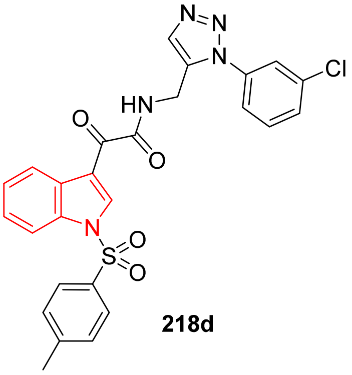

218d

IC_{50} = >100, >100, >100 μM against SKOV3, DU145, HELA, respectively; IC_{50} = 3.03, 0.85, 9.48 μM against COX-1. COX-2, 5-LOX, respectively

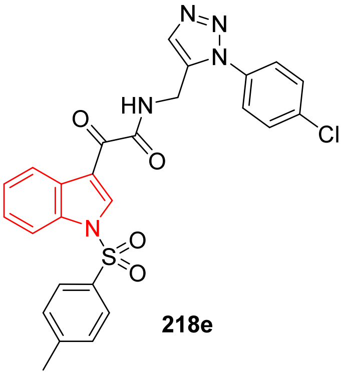

218e

IC_{50} = >100, >100, >100 μM against SKOV3, DU145, HELA, respectively; IC_{50} = 2.14, 1.86, 10.62 μM against COX-1. COX-2, 5-LOX, respectively

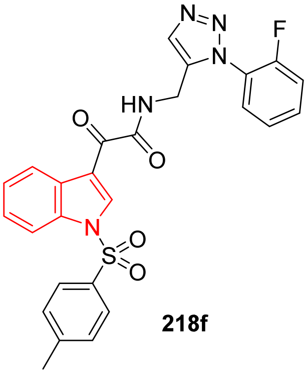

218f

IC_{50} = ND, ND, ND μM against SKOV3, DU145, HELA, respectively; IC_{50} = 2.38, 0.97, 8.62 μM against COX-1. COX-2, 5-LOX, respectively

Fig. S41. Antiproliferation and enzymation inhibitory properties of indole-triazole conjugates **218**, Etoposide, Indomethacin, Celecoxib, and Norhihydroguaiaretic acid (ND = not done).

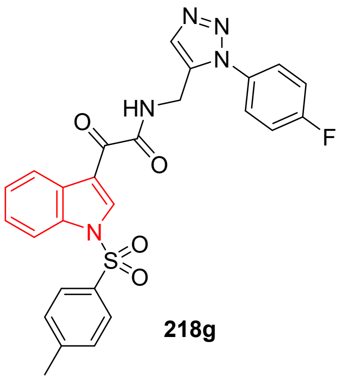

218g

IC_{50} = >100, >100, >100 μM against SKOV3, DU145, HELA, respectively; IC_{50} = 2.14, 1.88, 11.58 μM against COX-1. COX-2, 5-LOX, respectively

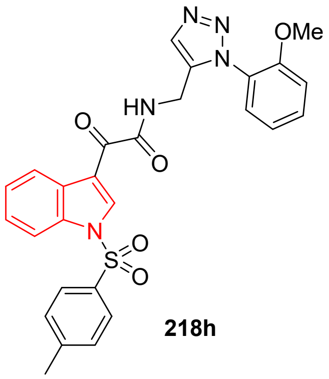

218h

IC_{50} = >100, >100, >100 μM against SKOV3, DU145, HELA, respectively; IC_{50} = 2.25, 0.44, 7.93 μM against COX-1. COX-2, 5-LOX, respectively

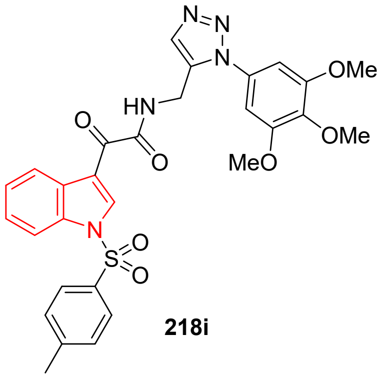

218i

IC_{50} = >100, >100, >100 μM against SKOV3, DU145, HELA, respectively; IC_{50} = 2.15, 0.85, 7.85 μM against COX-1. COX-2, 5-LOX, respectively

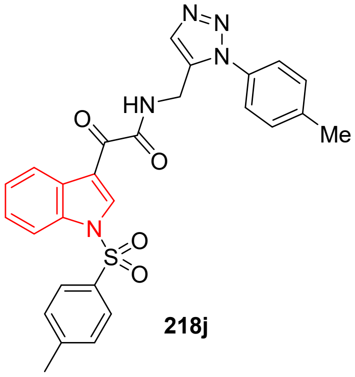

218j

IC_{50} = >100, >100, >100 μM against SKOV3, DU145, HELA, respectively; IC_{50} = 3.12, 1.43, 9.59 μM against COX-1. COX-2, 5-LOX, respectively

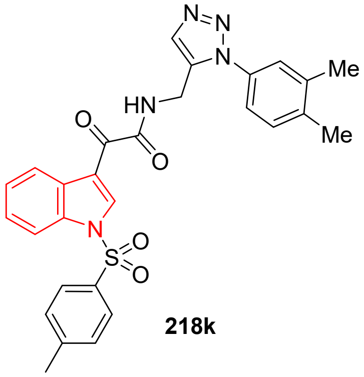

218k

IC_{50} = 20.71, >100, >100 μM against SKOV3, DU145, HELA, respectively; IC_{50} = 3.52, 1.66, 9.74 μM against COX-1. COX-2, 5-LOX, respectively

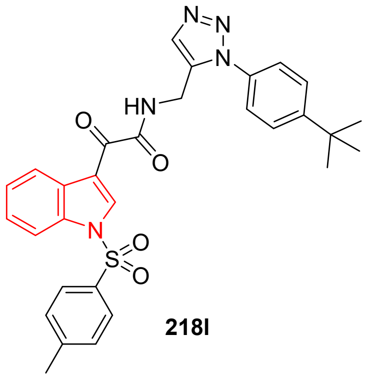

218l

IC_{50} = >100, 17.10, 50.24 μM against SKOV3, DU145, HELA, respectively; IC_{50} = 2.26, 1.25, 9.83 μM against COX-1. COX-2, 5-LOX, respectively

Fig. S41 (continued). Antiproliferation and enzymation inhibitory properties of indole-triazole conjugates **218**, Etoposide, Indomethacin, Celecoxib, and Norhihydroguaiaretic acid (ND = not done).

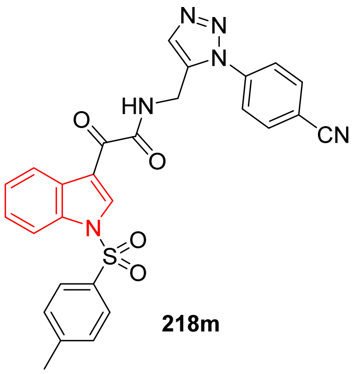

218m

IC_{50} = >100, 49.77, >100 μM against SKOV3, DU145, HELA, respectively; IC_{50} = 2.13, 0.48, 11.36 μM against COX-1. COX-2, 5-LOX, respectively

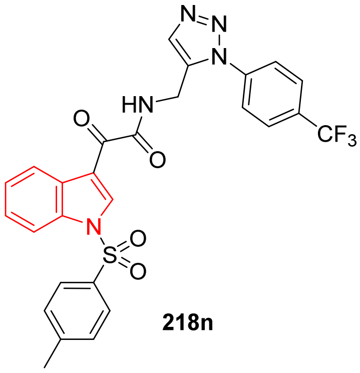

218n

IC_{50} = >100, >100, >100 μM against SKOV3, DU145, HELA, respectively; IC_{50} = 2.86, 1.21, 9.54 μM against COX-1. COX-2, 5-LOX, respectively

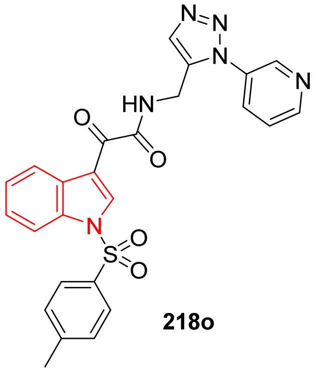

218o

IC_{50} = >100, >100, >100 μM against SKOV3, DU145, HELA, respectively; IC_{50} = 2.23, 0.35, 9.48 μM against COX-1. COX-2, 5-LOX, respectively

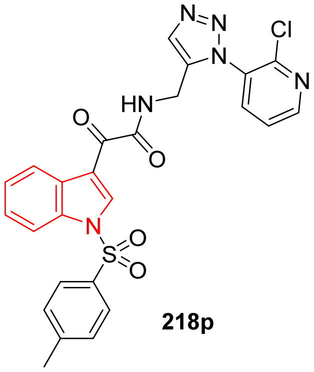

218p

IC_{50} = 29.32, >100, 21.64 μM against SKOV3, DU145, HELA, respectively; IC_{50} = 2.14, 1.99, 11.62 μM against COX-1. COX-2, 5-LOX, respectively

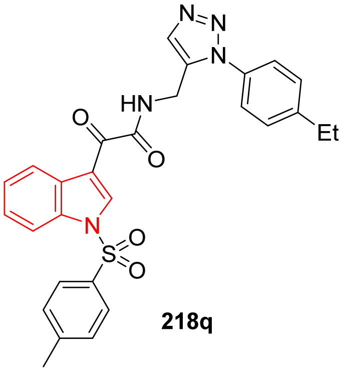

218q

IC_{50} = >100, >100, >100 μM against SKOV3, DU145, HELA, respectively; IC_{50} = 2.65, 0.22, 7.46 μM against COX-1. COX-2, 5-LOX, respectively

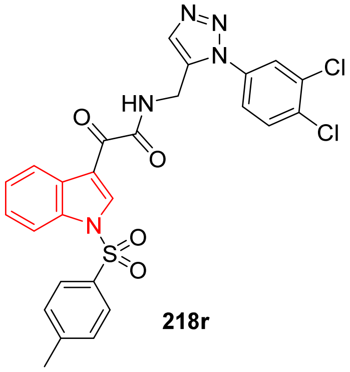

218r

IC_{50} = >100, 11.88, >100 μM against SKOV3, DU145, HELA, respectively; IC_{50} = 2.44, 0.32, 8.51 μM against COX-1. COX-2, 5-LOX, respectively

Fig. S41 (continued). Antiproliferation and enzymation inhibitory properties of indole-triazole conjugates **218**, Etoposide, Indomethacin, Celecoxib, and Norhihydroguaiaretic acid (ND = not done).

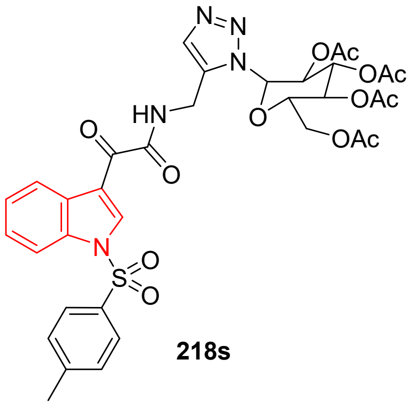

218s

IC_{50} = >100, 18.53, >100 μM against SKOV3, DU145, HELA, respectively; IC_{50} = 2.57, 0.12, 7.43 μM against COX-1. COX-2, 5-LOX, respectively

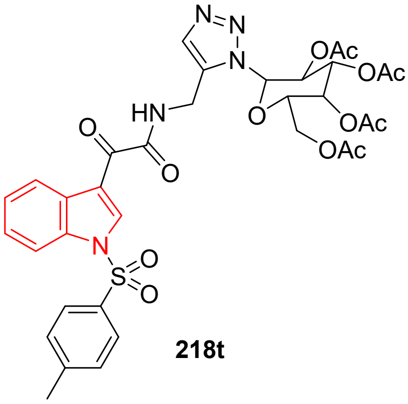

218t

IC_{50} = ND, ND, ND μM against SKOV3, DU145, HELA, respectively; IC_{50} = 3.06, 2.95, 12.65 μM against COX-1. COX-2, 5-LOX, respectively

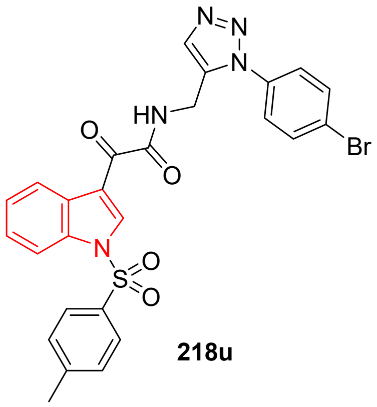

218u

IC_{50} = >100, >100, 7.79 μM against SKOV3, DU145, HELA, respectively; IC_{50} = 2.25, 1.75, 9.82 μM against COX-1. COX-2, 5-LOX, respectively

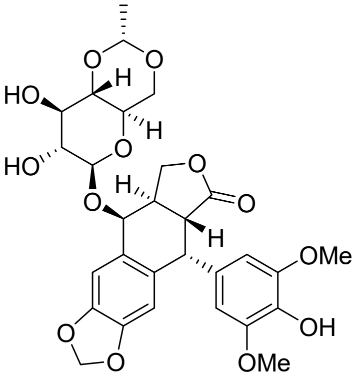

Etoposide (VP16)

IC_{50} = 2.39, 9.8, 7.43 μM against SKOV3, DU145, HELA, respectively

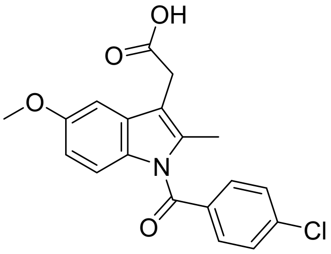

Indomethacin

IC_{50} = 0.0068, 0.049 μM against COX-1. COX-2, respectively

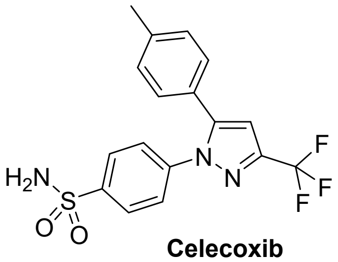

Celecoxib

IC_{50} = 14.8, 0.041 μM against COX-1. COX-2, respectively

Fig. S41 (continued). Antiproliferation and enzymation inhibitory properties of indole-triazole conjugates **218**, Etoposide, Indomethacin, Celecoxib, and Norhihydroguaiaretic acid (ND = not done).

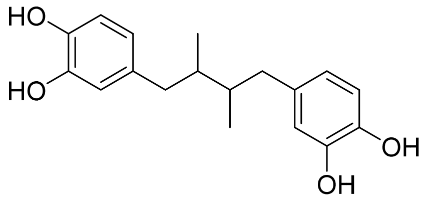

Norhihydroguaiaretic acid (NDGA)

IC_{50} = 7.31 μM against 5-LOX

Fig. S41 (continued). Antiproliferation and enzymation inhibitory properties of indole-triazole conjugates **218**, Etoposide, Indomethacin, Celecoxib, and Norhihydroguaiaretic acid (ND = not done).

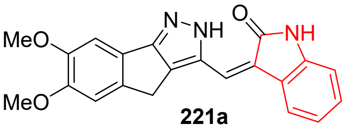

221a

IC_{50} = 2.16 ± 0.45, 2.92 ± 0.68, 3.45 ± 1.12, 4.36 ± 0.68 μM against HeLa, A549, MDA-MB-231, and HEK-293, respectively

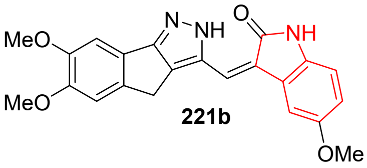

221b

IC_{50} = 2.58 ± 0.15, 3.43 ± 0.23, 4.14 ± 0.49, 5.1 ± 0.59 μM against HeLa, A549, MDA-MB-231, and HEK-293, respectively

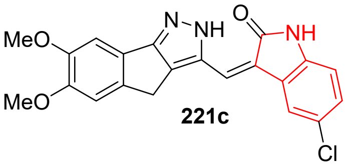

221c

IC_{50} = 8.96 ± 0.75, 13.54 ± 1.46, 12.29 ± 0.52, 9.21 ± 1.34 μM against HeLa, A549, MDA-MB-231, and HEK-293, respectively

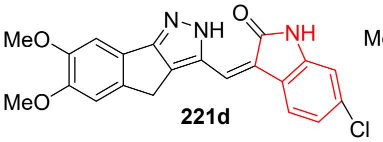

221d

IC_{50} = 1.33 ± 0.19, 2.44 ± 0.17, 2.08 ± 1.19, 2.97 ± 0.08 μM against HeLa, A549, MDA-MB-231, and HEK-293, respectively

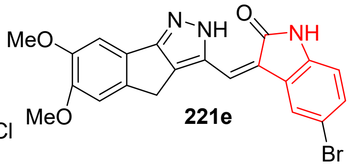

221e

IC_{50} = 11.52 ± 1.55, 17.12 ± 0.62, 18.68 ± 1.26, 19.56 ± 2.15 μM against HeLa, A549, MDA-MB-231, and HEK-293, respectively

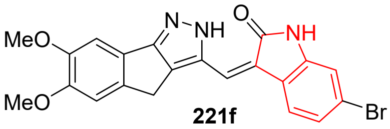

221f

IC_{50} = 20.52 ± 1.39, 22.52 ± 1.4, 20.35 ± 2.08, 24.63 ± 1.65 μM against HeLa, A549, MDA-MB-231, and HEK-293, respectively

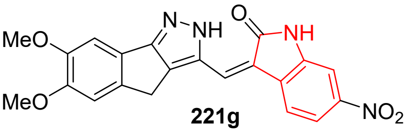

221g

IC_{50} = 30.74 ± 2.11, 34.43 ± 2.03, 29.86 ± 1.12, 36.73 ± 2.35 μM against HeLa, A549, MDA-MB-231, and HEK-293, respectively

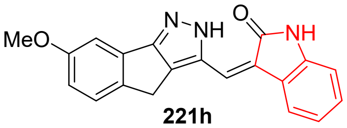

221h

IC_{50} = 15.97 ± 2.21, 20.56 ± 1.39, 19.28 ± 0.49, 22.48 ± 1.36 μM against HeLa, A549, MDA-MB-231, and HEK-293, respectively

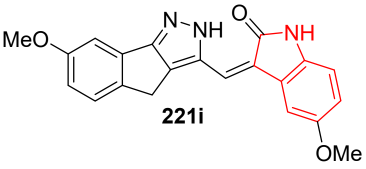

221i

IC_{50} = 24.53 ± 2.79, 30.05 ± 2.12, 30.63 ± 0.52, 33.37 ± 2.1 μM against HeLa, A549, MDA-MB-231, and HEK-293, respectively

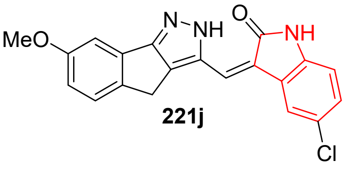

221j

IC_{50} = 17.52 ± 1.5, 17.6 ± 1.38, 20.75 ± 1.19, 27.41 ± 2.1 μM against HeLa, A549, MDA-MB-231, and HEK-293, respectively

Fig. S42. Antiproliferation properties (μM ± SD) of 3-[(indeno[1,2-*c*]-pyrazole-3-yl)methylene]indolin-2-ones **221** and Combretastatin A-4 (CA-4).

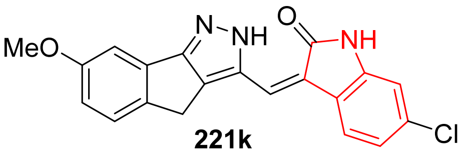

221k

IC_{50} = 16.99 ± 0.59, 20.95 ± 0.77, 19.04 ± 1.26, 21.87 ± 2.05 μM against HeLa, A549, MDA-MB-231, and HEK-293, respectively

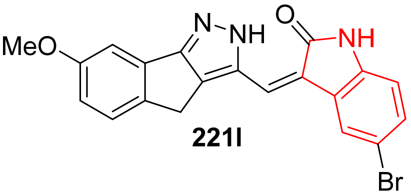

221l

IC_{50} = 30.94 ± 2.12, 29.79 ± 2.23, 34.15 ± 2.08, 35.75 ± 1.98 μM against HeLa, A549, MDA-MB-231, and HEK-293, respectively

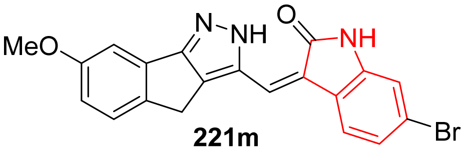

221m

IC_{50} = 25.38 ± 1.49, 26.14 ± 1.21, 23.4 ± 1.12, 28.57 ± 1.61 μM against HeLa, A549, MDA-MB-231, and HEK-293, respectively

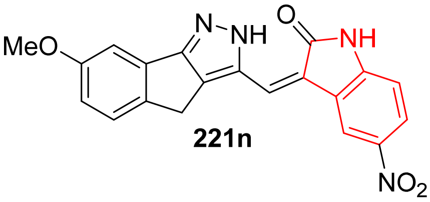

221n

IC_{50} = 22.09 ± 0.78, 26 ± 0.62, 29.17 ± 0.49, 30.79 ± 0.59 μM against HeLa, A549, MDA-MB-231, and HEK-293, respectively

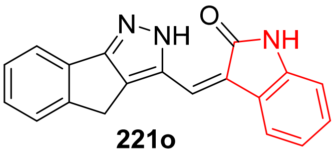

221o

IC_{50} = 11.65 ± 0.84, 16.57 ± 1.81, 18.96 ± 1.12, 24.26 ± 0.42 μM against HeLa, A549, MDA-MB-231, and HEK-293, respectively

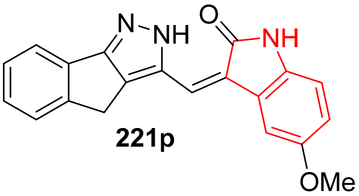

221p

IC_{50} = 30.11 ± 2.78, 29.55 ± 3.4, 26.73 ± 0.49, 33.86 ± 1.84 μM against HeLa, A549, MDA-MB-231, and HEK-293, respectively

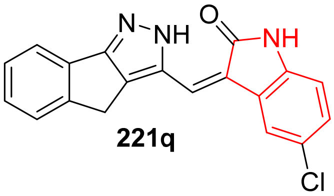

221q

IC_{50} = 15.16 ± 1.95, 20.48 ± 1.34, 16.81 ± 0.52, 24.29 ± 1.22 μM against HeLa, A549, MDA-MB-231, and HEK-293, respectively

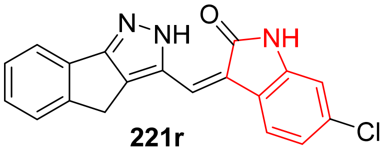

221r

IC_{50} = 14.6 ± 1.36, 20.53 ± 1.45, 24.34 ± 1.19, 19.37 ± 1.17 μM against HeLa, A549, MDA-MB-231, and HEK-293, respectively

Fig. S42 (continued). Antiproliferation properties (μM ± SD) of 3-[(indeno[1,2-*c*]-pyrazole-3-yl)methylene]indolin-2-ones **221** and Combretastatin A-4 (CA-4).

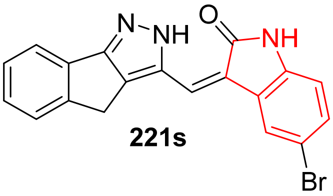

221s

IC_{50} = 16.85 ± 0.69, 21.09 ± 1.54, 20.34 ± 1.26, 22.48 ± 1.32 μM against HeLa, A549, MDA-MB-231, and HEK-293, respectively

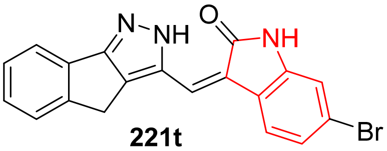

221t

IC_{50} = 30.69 ± 1.33, 35.55 ± 1.37, 29.65 ± 2.08, 34.23 ± 2.74 μM against HeLa, A549, MDA-MB-231, and HEK-293, respectively

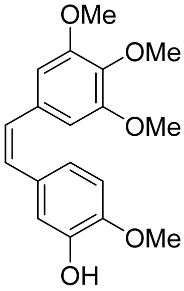

Combretastatin A-4 (CA-4)

IC_{50} = 1.43 ± 0.25, 1.15 ± 0.18, 0.92 ± 0.007, 1.73 ± 0.41 μM against HeLa, A549, MDA-MB-231, and HEK-293, respectively

Fig. S42 (continued). Antiproliferation properties (μM ± SD) of 3-[(indeno[1,2-*c*]-pyrazole-3-yl)methylene]indolin-2-ones **221** and Combretastatin A-4 (CA-4).

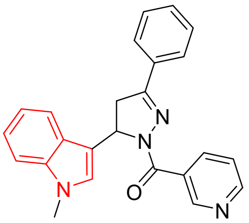

223a

GI_{50} = 7.20 ± 0.08, >10, 4.63 ± 0.02, >10, >300 against MCF-7, A549, HepG2, HeLa, 293T, respectively; IC_{50} = 26.7 ± 0.4 of tubulin polymerization inhibition

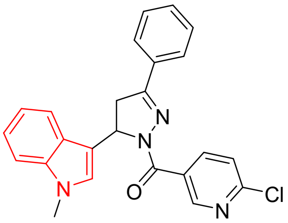

223b

GI_{50} = 9.25 ± 0.05, 8.46 ± 0.12, 7.91 ± 0.10, 8.31 ± 0.02, >300 against MCF-7, A549, HepG2, HeLa, 293T, respectively; IC_{50} = 31.7 ± 1.3 of tubulin polymerization inhibition

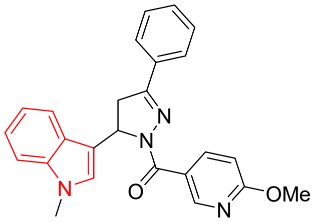

223c

GI_{50} = 4.31 ± 0.09, 3.04 ± 0.13, 3.13 ± 0.08, 3.98 ± 0.09, >300 against MCF-7, A549, HepG2, HeLa, 293T, respectively; IC_{50} = 9.7 ± 0.1 of tubulin polymerization inhibition

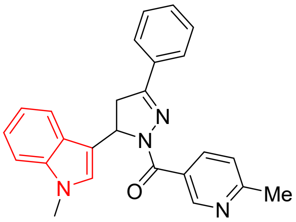

223d

GI_{50} = 4.01 ± 0.05, 1.19 ± 0.01, 1.22 ± 0.05, 2.39 ± 0.10, 154.36 ± 4.42 against MCF-7, A549, HepG2, HeLa, 293T, respectively; IC_{50} = 13.2 ± 0.4 of tubulin polymerization inhibition

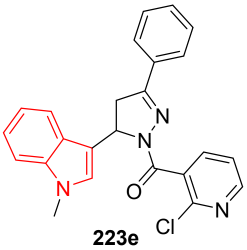

223e

GI_{50} = >10, >10, >10,>10, >300 against MCF-7, A549, HepG2, HeLa, 293T, respectively; IC_{50} = >40 of tubulin polymerization inhibition

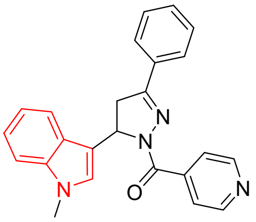

223f

GI_{50} = 1.11 ± 0.02, 0.99 ± 0.01. 0.63 ± 0.03, 0.59 ± 0.02, 97.29 ± 2.30 against MCF-7, A549, HepG2, HeLa, 293T, respectively; IC_{50} = >40 of tubulin polymerization inhibition

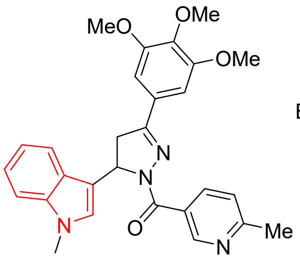

223g

GI_{50} = 0.18 ± 0.01, 0.23 ± 0.02, 0.31 ± 0.02, 0.21 ± 0.02, >300 against MCF-7, A549, HepG2, HeLa, 293T, respectively; IC_{50} = 2.3 ± 0.2 of tubulin polymerization inhibition

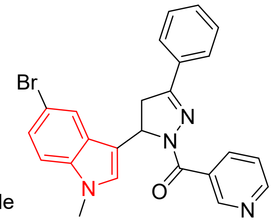

223h

GI_{50} = 1.52 ± 0.05, 1.60 ± 0.02, 2.52 ± 0.08, 1.73 ± 0.05, >300 against MCF-7, A549, HepG2, HeLa, 293T, respectively; IC_{50} = 3.4 ± 0.2 of tubulin polymerization inhibition

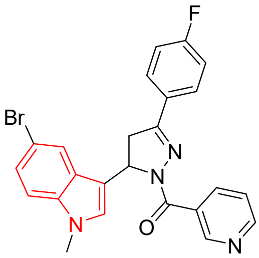

223i

GI_{50} = 0.48 ± 0.01, 1.38 ± 0.05. 0.48 ± 0.03, 0.63 ± 0.03, >300 against MCF-7, A549, HepG2, HeLa, 293T, respectively; IC_{50} = 5.2 ± 0.2 of tubulin polymerization inhibition

Fig. S43. Antiproliferation and tubulin polymerization inhibitory properties of the synthesized nicotinoyl/isonicotinyl pyrazolines featuring indolyl heterocycle **223** and Combretastatin A-4 (CA-4).

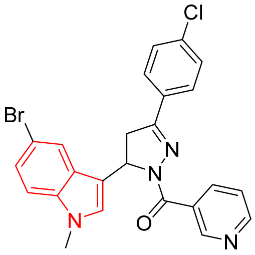

223j

GI_{50} = 8.10 ± 0.14, 7.65 ± 0.02, 5.80 ± 0.08, 5.2 ± 0.10, >300 against MCF-7, A549, HepG2, HeLa, 293T, respectively; IC_{50} = 19.4 ± 0.3 of tubulin polymerization inhibition

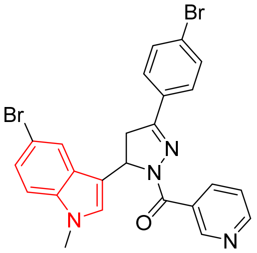

223k

GI_{50} = 11.8 ± 0.44, >10, 6.91 ± 0.04,>10, >300 against MCF-7, A549, HepG2, HeLa, 293T, respectively; IC_{50} = 18.4 ± 0.8 of tubulin polymerization inhibition

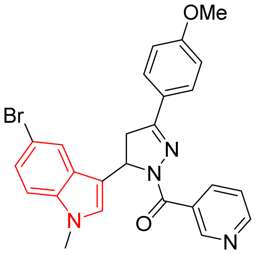

223l

GI_{50} = 1.56 ± 0.06, 1.19 ± 0.01, 0.54 ± 0.03, 0.89 ± 0.03, >300 against MCF-7, A549, HepG2, HeLa, 293T, respectively; IC_{50} = 4.5 ± 0.3 of tubulin polymerization inhibition

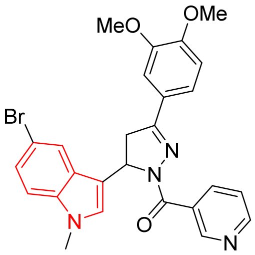

223m

GI_{50} = 1.17 ± 0.03, 2.38 ± 0.09, 2.45 ± 0.08, 2.09 ± 0.07, >300 against MCF-7, A549, HepG2, HeLa, 293T, respectively; IC_{50} = 5.6 ± 0.1 of tubulin polymerization inhibition

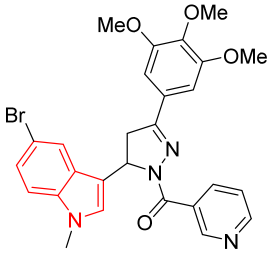

223n

GI_{50} = >10, >10, >10, >10, 130.78 ± 4.27 against MCF-7, A549, HepG2, HeLa, 293T, respectively; IC_{50} = >40 of tubulin polymerization inhibition

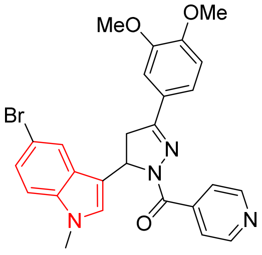

223o

GI_{50} = >10, 4.25 ± 0.13, >10, >10. >300 against MCF-7, A549, HepG2, HeLa, 293T, respectively; IC_{50} = 17.8 ± 0.4 of tubulin polymerization inhibition

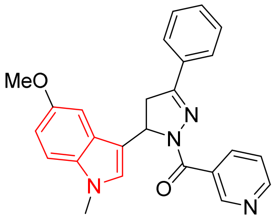

223p

GI_{50} = 1.16 ± 0.05, >10, 5.11 ± 0.10, 3.59 ± 0.06, >300 against MCF-7, A549, HepG2, HeLa, 293T, respectively; IC_{50} = 4.3 ± 0.2 of tubulin polymerization inhibition

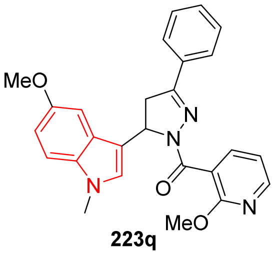

223q

GI_{50} = 0.99 ± 0.01, 1.11 ± 0.04, 1.33 ± 0.05, 1.1 ± 0.05, >300 against MCF-7, A549, HepG2, HeLa, 293T, respectively; IC_{50} = 2.9 ± 0.1 of tubulin polymerization inhibition

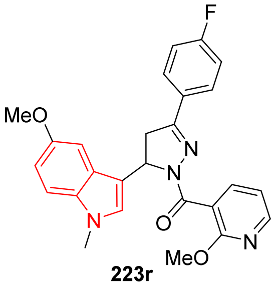

223r

GI_{50} = 0.48 ± 0.01, 0.71 ± 0.03, 0.22 ± 0.02, 0.41 ± 0.02, >300 against MCF-7, A549, HepG2, HeLa, 293T, respectively; IC_{50} = 2.6 ± 0.2 of tubulin polymerization inhibition

Fig. S43 (continued). Antiproliferation and tubulin polymerization inhibitory properties of the synthesized nicotinoyl/isonicotinyl pyrazolines featuring indolyl heterocycle **223** and Combretastatin A-4 (CA-4).

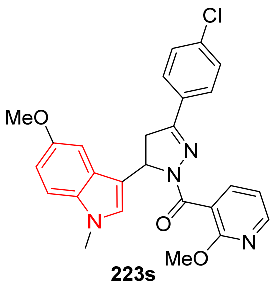

223s

GI_{50} = 3.71 ± 0.05, 3.39 ± 0.11, 2.08 ± 0.09, 2.26 ± 0.03, >300 against MCF-7, A549, HepG2, HeLa, 293T, respectively; IC_{50} = 15.6 ± 0.1 of tubulin polymerization inhibition

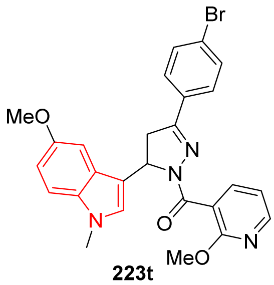

223t

GI_{50} = 6.96 ± 0.28, >10, >10, >10, >300 against MCF-7, A549, HepG2, HeLa, 293T, respectively; IC_{50} = 19.3 ± 0.1 of tubulin polymerization inhibition

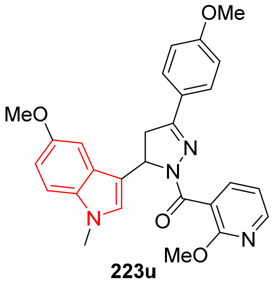

223u

GI_{50} = 0.56 ± 0.03, 0.45 ± 0.01, 0.23 ± 0.02, 0.18 ± 0.01, >300 against MCF-7, A549, HepG2, HeLa, 293T, respectively; IC_{50} = 2.3 ± 0.1 of tubulin polymerization inhibition

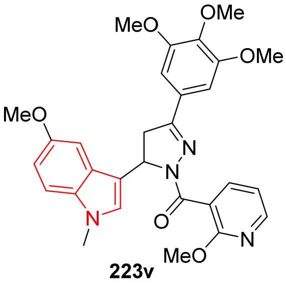

223v

GI_{50} = >10, >10, >10, >10, >300 against MCF-7, A549, HepG2, HeLa, 293T, respectively; IC_{50} = >40 of tubulin polymerization inhibition

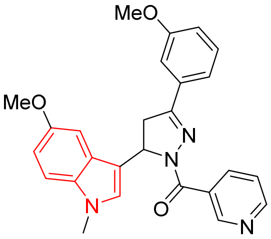

223w

GI_{50} = 0.81 ± 0.04, 1.02 ± 0.05, 0.91 ± 0.01, 1.27 ± 0.05, 125.40 ± 1.82 against MCF-7, A549, HepG2, HeLa, 293T, respectively; IC_{50} = 2.1 ± 0.2 of tubulin polymerization inhibition

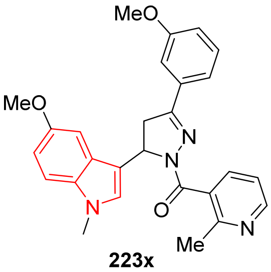

223x

GI_{50} = 0.09 ± 0.01, 0.59 ± 0.02, 0.029 ± 0.01, 0.034 ± 0.01, >300 against MCF-7, A549, HepG2, HeLa, 293T, respectively; IC_{50} = 1.6 ± 0.1 of tubulin polymerization inhibition

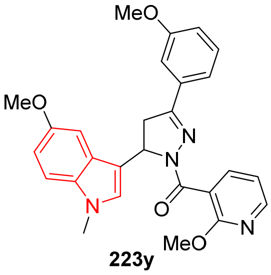

223y

GI_{50} = 0.13 ± 0.01, 0.47 ± 0.03, 0.19 ± 0.01, 0.34 ± 0.02, >300 against MCF-7, A549, HepG2, HeLa, 293T, respectively; IC_{50} = 1.9 ± 0.1 of tubulin polymerization inhibition

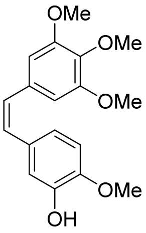

Combretastatin A-4 (CA-4)

GI_{50} = 0.14 ± 0.02, 0.31 ± 0.02, 0.17 ± 0.02, 0.092 ± 0.01, >300 against MCF-7, A549, HepG2, HeLa, 293T, respectively; IC_{50} = 2.1 ± 0.2 of tubulin polymerization inhibition

Fig. S43 (continued). Antiproliferation and tubulin polymerization inhibitory properties of the synthesized nicotinoyl/isonicotinyl pyrazolines featuring indolyl heterocycle **223** and Combretastatin A-4 (CA-4).

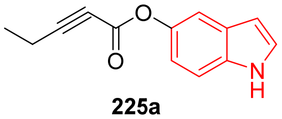

225a

IC_{50} = 13.1 ± 0.3, 10.7 ± 0.2, 9.8 ± 0.3, 12.1 ± 0.6, >200, >200, >200 μM against MCF-7, MDA-MB-231, HeLa, Ishikawa, MCF-10A, Hek-293, and 3T3-L1, respectively

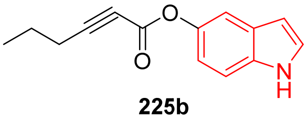

225b

IC_{50} = 30.2 ± 0.2, 15.9 ± 0.5, 14.5 ± 0.6, 30.5 ± 0.5, >200, >200, >200 μM against MCF-7, MDA-MB-231, HeLa, Ishikawa, MCF-10A, Hek-293, and 3T3-L1, respectively

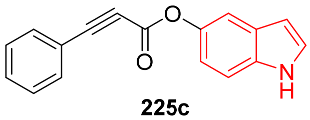

225c

IC_{50} = 3.8 ± 0.7, 9.9 ± 0.3, 3.6 ± 0.5, 10.1 ± 0.7, >200, >200, >200 μM against MCF-7, MDA-MB-231, HeLa, Ishikawa, MCF-10A, Hek-293, and 3T3-L1, respectively

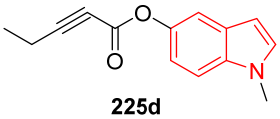

225d

IC_{50} = 23.0 ± 0.4, 23.5 ± 0.4, 14.0 ± 0.7, 13.1 ± 0.3, >200, >200, >200 μM against MCF-7, MDA-MB-231, HeLa, Ishikawa, MCF-10A, Hek-293, and 3T3-L1, respectively

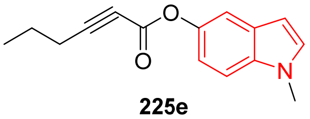

225e

IC_{50} = >200, >200, >200, >200, >200, >200, >200 μM against MCF-7, MDA-MB-231, HeLa, Ishikawa, MCF-10A, Hek-293, and 3T3-L1, respectively

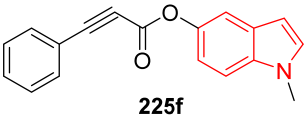

225f

IC_{50} = 37.6 ± 0.5, 40.7 ± 0.8, 49.5 ± 0.9, 77.7 ± 0.4, >200, >200, >200 μM against MCF-7, MDA-MB-231, HeLa, Ishikawa, MCF-10A, Hek-293, and 3T3-L1, respectively

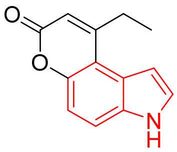

226a

IC_{50} = 86.0 ± 0.3, 44.4 ± 0.3, 21.1 ± 0.7, 84.0 ± 0.5, >200, >200, >200 μM against MCF-7, MDA-MB-231, HeLa, Ishikawa, MCF-10A, Hek-293, and 3T3-L1, respectively

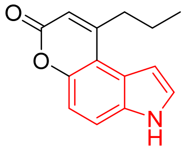

226b

IC_{50} = >200, 172.4 ± 0.6, 141.4 ± 0.7, >200, >200, >200 >200 μM against MCF-7, MDA-MB-231, HeLa, Ishikawa, MCF-10A, Hek-293, and 3T3-L1, respectively

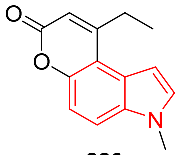

226c

IC_{50} = 131.5 ± 0.3, 80.2 ± 0.5, >200, >200, >200, >200, >200 μM against MCF-7, MDA-MB-231, HeLa, Ishikawa, MCF-10A, Hek-293, and 3T3-L1, respectively

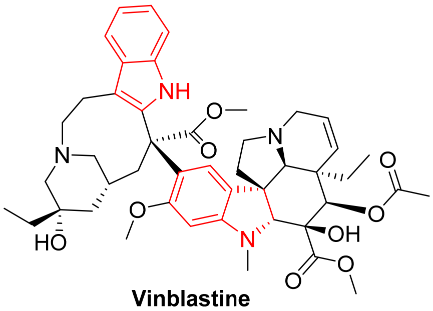

Vinblastine

IC_{50} = $4.5x10^{-2}$ ± 0.6, $1.6x10^{-1}$ ± 0.3, $6.7x10^{-2}$ ± 0.5, $2.5x10^{-2}$ ± 0,7, $2.3x10^{-2}$ ± 0.6, 80.8 ± 0.4, $1.9x10^{-4}$ ± 0.8 μM against MCF-7, MDA-MB-231, HeLa, Ishikawa, MCF-10A, Hek-293, and 3T3-L1, respectively

Fig. S44. Antiproliferation properties (μM ± SD) of the synthesized indoles **225**, pyranoindole **226** and Vinblastine.

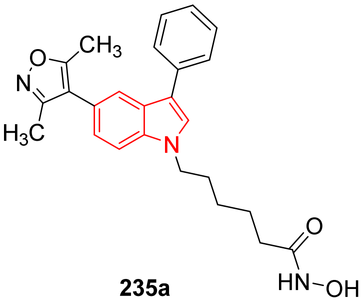

235a

GI_{50} = 11.29 ± 1.22 μM against THP-1; IC_{50} = 0.223 ± 0.031, 0.260 ± 0.022, 0.154 ± 0.023, 0.120 ± 0.010, >1 against HDACs, HDAC1, HDAC2, HDAC3, HDAC6, respectively; % inhibition rate = 86, 66, 21 at 100, 10 and 1 μM

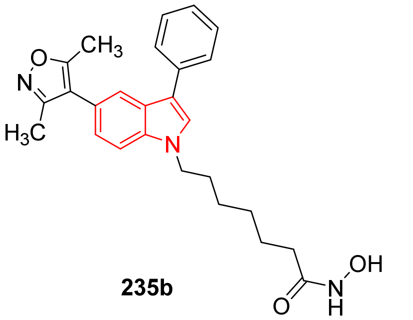

235b

GI_{50} = 12.43 ± 1.63 μM against THP-1; IC_{50} = 0.183 ± 0.013, 0.287 ± 0.016, 0.180 ± 0.009, 0.202 ± 0.012, >1 against HDACs, HDAC1, HDAC2, HDAC3, HDAC6, respectively; % inhibition rate = 91, 76, 34 at 100, 10 and 1 μM

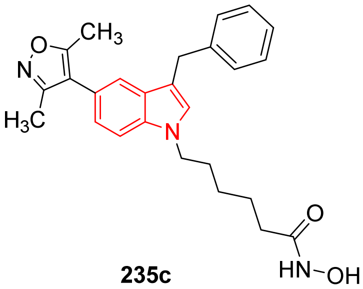

235c

GI_{50} = 12.45 ± 2.01 μM against THP-1; IC_{50} = 0.160 ± 0.021, 0.189 ± 0.011, 0.110 ± 0.012, 0.089 ± 0.007, >1 against HDACs, HDAC1, HDAC2, HDAC3, HDAC6, respectively; % inhibition rate = 96, 64, 16 at 100, 10 and 1 μM

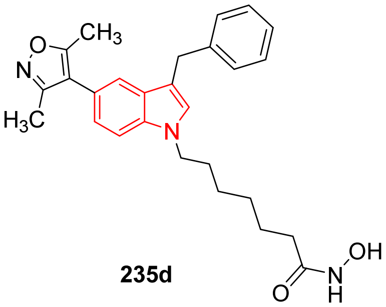

235d

GI_{50} = 12.13 ± 1.39 μM against THP-1; IC_{50} = 0.122 ± 0.013, 0.179 ± 0.011, 0.118 ± 0.019, 0.023 ± 0.009, >1 against HDACs, HDAC1, HDAC2, HDAC3, HDAC6, respectively; % inhibition rate = 96, 65, 31 at 100, 10 and 1 μM

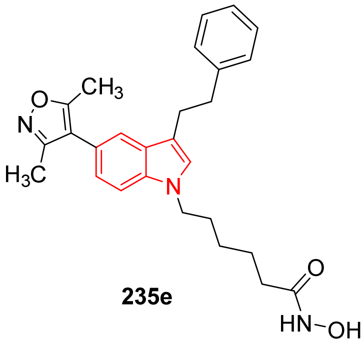

235e

GI_{50} = 10.06 ± 0.97 μM against THP-1; IC_{50} = 0.307 ± 0.019, 0.247 ± 0.021, 0.538 ± 0.064, 0.076 ± 0.010, >1 against HDACs, HDAC1, HDAC2, HDAC3, HDAC6, respectively; % inhibition rate = 98, 49, 39 at 100, 10 and 1 μM

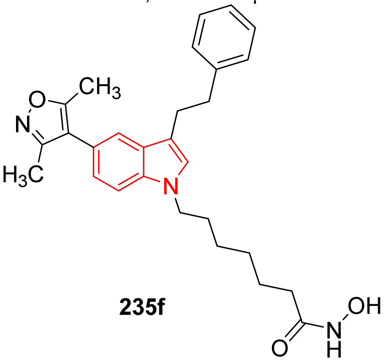

235f

GI_{50} = 8.79 ± 0.65 μM against THP-1; IC_{50} = 0.172 ± 0.021, 0.240 ± 0.017, 0.139 ± 0.016, 0.028 ± 0.005, >1 against HDACs, HDAC1, HDAC2, HDAC3, HDAC6, respectively; % inhibition rate = 94, 64, 24 at 100, 10 and 1 μM

Fig. S45. Antiproferation and inhibitory properties of HDAC and BRD4 of the synthesized indole-isoxazole conjugates **235**, Vorinostat and JQ1.

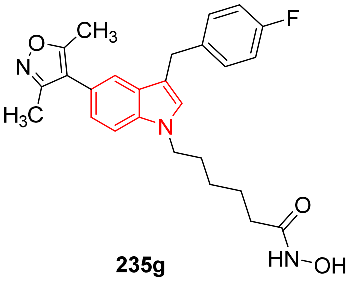

235g

GI_{50} = 21.26 ± 2.54 μM against THP-1;
IC_{50} = 0.286 ± 0.034, 0.341 ± 0.023, 0.517 ± 0.073, 0.039 ± 0.007, >1 against HDACs, HDAC1, HDAC2, HDAC3, HDAC6, respectively; % inhibition rate = 96, 76, 19 at 100, 10 and 1 μM

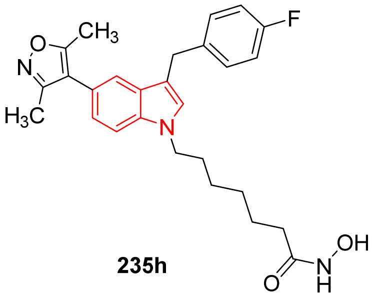

235h

GI_{50} = 15.5 ± 1.23 μM against THP-1;
IC_{50} = 0.291 ± 0.016, 0.181 ± 0.011, 0.298 ± 0.033, 0.005 ± 0.002, >1 against HDACs, HDAC1, HDAC2, HDAC3, HDAC6, respectively; % inhibition rate = 95, 88, 28 at 100, 10 and 1 μM

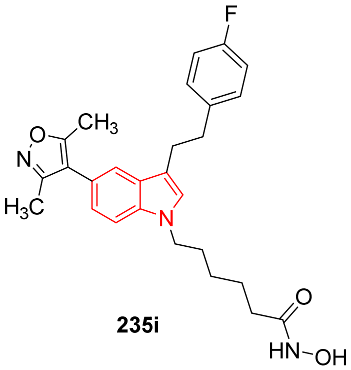

235i

GI_{50} = 12.98 ± 1.35 μM against THP-1;
IC_{50} =0.207 ± 0.018, 0.259 ± 0.031, 0.946 ± 0.086, 0.017 ± 0.004, >1 against HDACs, HDAC1, HDAC2, HDAC3, HDAC6, respectively; % inhibition rate = 97, 52, 9 at 100, 10 and 1 μM

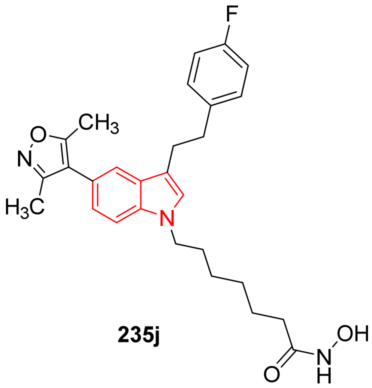

235j

GI_{50} = 14.11 ± 2.23 μM against THP-1;
IC_{50} = 0.030 ± 0.008, 0.079 ± 0.007, 0.487 ± 0.034, 0.064 ± 0.007, >1 against HDACs, HDAC1, HDAC2, HDAC3, HDAC6, respectively; % inhibition rate = 95, 50, 16 at 100, 10 and 1 μM

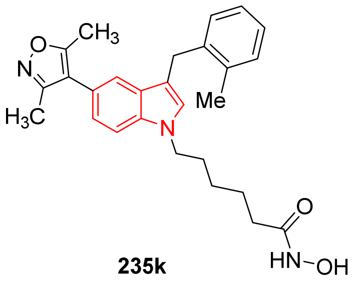

235k

GI_{50} = 14.82 ± 0.78 μM against THP-1;
IC_{50} = 0.369 ± 0.032, 0.314 ± 0.046, >1, 0.062 ± 0.012, >1 against HDACs, HDAC1, HDAC2, HDAC3, HDAC6, respectively; % inhibition rate = 95, 59, 18 at 100, 10 and 1 μM

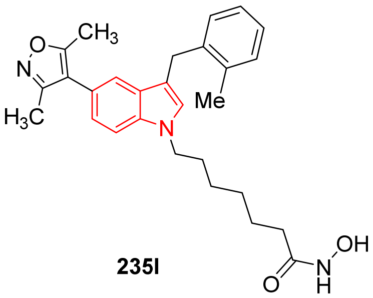

235l

GI_{50} = 13.93 ± 1.23 μM against THP-1;
IC_{50} = 0.149 ± 0.009, 0.220 ± 0.031, >1, 0.050 ± 0.009, >1 against HDACs, HDAC1, HDAC2, HDAC3, HDAC6, respectively; % inhibition rate = 97, 55, 14 at 100, 10 and 1 μM

Fig. S45 (continued). Antiproferation and inhibitory properties of HDAC and BRD4 of the synthesized indole-isoxazole conjugates **235**, Vorinostat and JQ1.

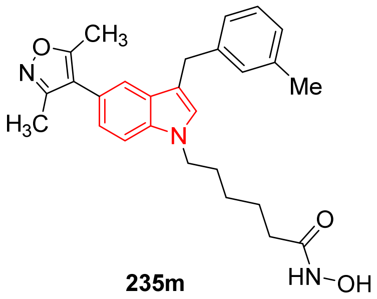

235m

GI_{50} = 24.21 ± 2.35 μM against THP-1; IC_{50} = 0.244 ± 0.033, 0.222 ± 0.019, 0.568 ± 0.067, 0.062 ± 0.013, >1 against HDACs, HDAC1, HDAC2, HDAC3, HDAC6, respectively; % inhibition rate = 94, 71, 15 at 100, 10 and 1 μM

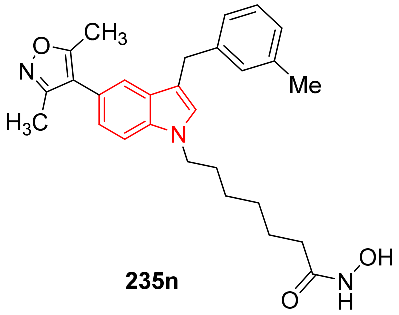

235n

GI_{50} = 23.34 ± 1.87 μM against THP-1; IC_{50} = 0.094 ± 0.006, 0.253 ± 0.012, 0.127 ± 0.013, 0.024 ± 0.006, >1 against HDACs, HDAC1, HDAC2, HDAC3, HDAC6, respectively; % inhibition rate = 96, 69, 14 at 100, 10 and 1 μM

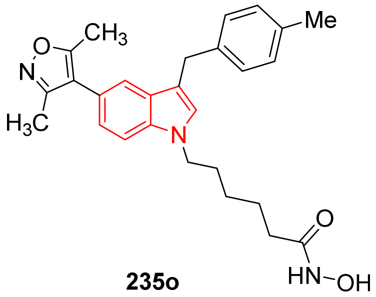

235o

GI_{50} = 16.00 ± 2.01 μM against THP-1; IC_{50} = 0.332 ± 0.041, 0.650 ± 0.053, 0.956 ± 0.071, 0.072 ± 0.011, >1 against HDACs, HDAC1, HDAC2, HDAC3, HDAC6, respectively; % inhibition rate = 97, 63, 18 at 100, 10 and 1 μM

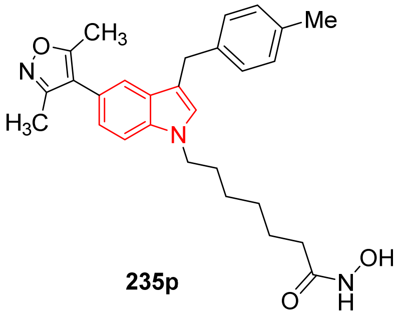

235p

GI_{50} = 12.96 ± 1.42 μM against THP-1; IC_{50} = 0.186 ± 0.023, 0.431 ± 0.033, 0.438 ± 0.038, 0.018 ± 0.007, >1 against HDACs, HDAC1, HDAC2, HDAC3, HDAC6, respectively; % inhibition rate = 95, 76, 13 at 100, 10 and 1 μM

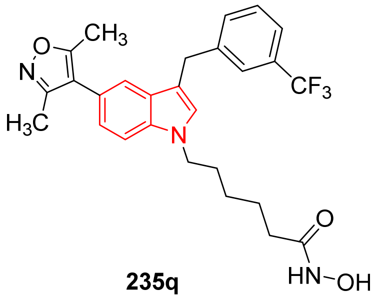

235q

GI_{50} = 7.82 ± 0.54 μM against THP-1; IC_{50} = 0.083 ± 0.011, 0.031 ± 0.005, 0.079 ± 0.010, 0.013 ± 0.003, >1 against HDACs, HDAC1, HDAC2, HDAC3, HDAC6, respectively; % inhibition rate = 92, 41, 12 at 100, 10 and 1 μM

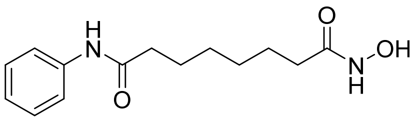

Vorinostat

GI_{50} = 0.51 ± 0.04 μM against THP-1; IC_{50} = 0.076 ± 0.002, 0.103 ± 0.008, 0.055 ± 0.007, 0.027 ± 0.006. 0.039 ± 0.002 against HDACs, HDAC1, HDAC2, HDAC3, HDAC6, respectively

Fig. S45 (continued). Antiproferation and inhibitory properties of HDAC and BRD4 of the synthesized indole-isoxazole conjugates **235**, Vorinostat and JQ1.

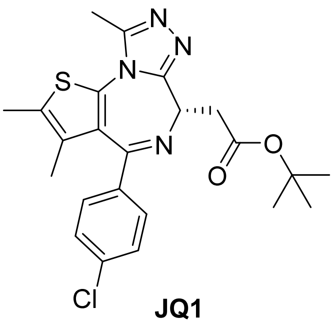

JQ1

GI_{50} = 0.13 ± 0.02 μM against THP-1;
% inhibition rate = 97, 96, 95
at 100, 10 and 1 μM

Fig. S45 (continued). Antiproferation and inhibitory properties of HDAC and BRD4 of the synthesized indole-isoxazole conjugates **235**, Vorinostat and JQ1.

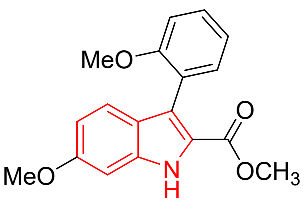

241a

EC_{50} = 1.90, 10.5 μM against CEM, RS4; 11, respectively

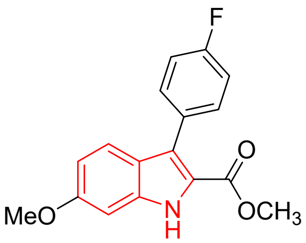

241b

EC_{50} = 10.0, 85.6 μM against CEM, RS4; 11, respectively

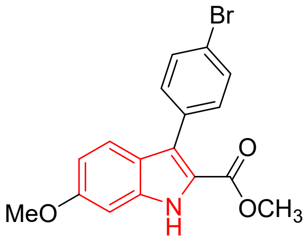

241c

EC_{50} = 0.33, 0.20 μM against CEM, RS4; 11, respectively

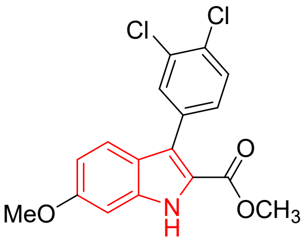

241d

EC_{50} = 0.58, 3.80 μM against CEM, RS4; 11, respectively

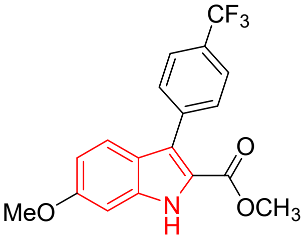

241d

EC_{50} = 0.20, 0.30 μM against CEM, RS4; 11, respectively

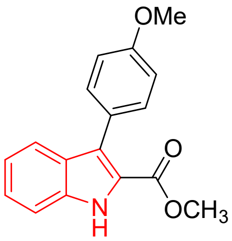

241e

EC_{50} = >100, >100 μM against CEM, RS4; 11, respectively

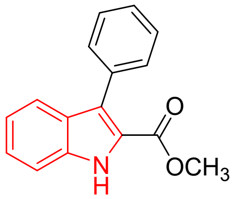

241f

EC_{50} = 35.8, 95.2 μM against CEM, RS4; 11, respectively

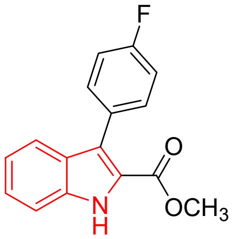

241g

EC_{50} = >100, >100 μM against CEM, RS4; 11, respectively

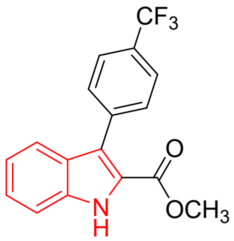

241h

EC_{50} = >100, >100 μM against CEM, RS4; 11, respectively

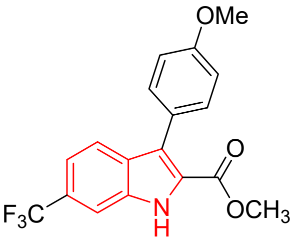

241i

EC_{50} = >100, >100 μM against CEM, RS4; 11, respectively

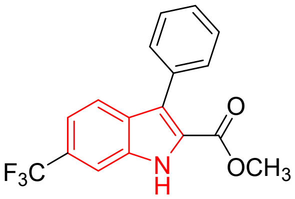

241j

EC_{50} = >100, >100 μM against CEM, RS4; 11, respectively

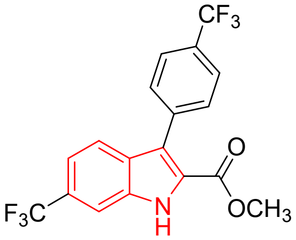

241j

EC_{50} = >100, >100 μM against CEM, RS4; 11, respectively

Fig. S46. Antiproliferation properties of 3,6-disubstituted-2-carboxyindoles **241**.
